# Jarisch-Herxheimer Reaction After Benzathine Penicillin G Treatment in Adults With Early Syphilis: Secondary Analysis of a Randomized Clinical Trial

**DOI:** 10.1001/jamanetworkopen.2024.59490

**Published:** 2025-02-13

**Authors:** Jodie A. Dionne, Chunming Zhu, Jorge Mejia-Galvis, Kimberly Workowski, Teresa A. Batteiger, Julia C. Dombrowski, Kenneth H. Mayer, Candice J. McNeil, Arlene C. Seña, Stephanie Taylor, Harold C. Wiesenfeld, Matthew M. Hamill, Charlotte Perlowski, Edward W. Hook

**Affiliations:** 1 Division of Infectious Diseases, Department of Medicine, University of Alabama at Birmingham; 2 Emmes Company LLC, Rockville, Maryland; 3 Enteric and Sexually Transmitted Infections Branch, National Institute of Allergy and Infectious Diseases, National Institutes of Health, Bethesda, Maryland; 4 Division of Infectious Diseases, Department of Medicine, Emory University, Atlanta, Georgia; 5 Division of Infectious Diseases, Department of Medicine, Indiana University School of Medicine, Indianapolis; 6 Division of Allergy and Infectious Diseases, University of Washington, Seattle; 7 The Fenway Institute, Fenway Health, Boston, Massachusetts; 8 Department of Medicine, Beth Israel Deaconess Medical Center/Harvard Medical School, Boston, Massachusetts; 9 Section on Infectious Diseases, Department of Medicine, Wake Forest University School of Medicine, Winston-Salem, North Carolina; 10 Division of Infectious Diseases, Department of Medicine, University of North Carolina at Chapel Hill; 11 Section on Infectious Diseases, Department of Medicine, Louisiana State University Health Sciences Center, New Orleans; 12 Department of Obstetrics, Gynecology and Reproductive Sciences, University of Pittsburgh, Pittsburgh, Pennsylvania; 13 Magee-Womens Research Institute, University of Pittsburgh, Pittsburgh, Pennsylvania; 14 Division of Infectious Diseases, Department of Medicine, Johns Hopkins University, Baltimore, Maryland; 15 FHI 360, Durham, North Carolina

## Abstract

**Importance:**

Syphilis rates have been increasing in the US for the past decade. The incidence of the Jarisch-Herxheimer reaction (JHR) after penicillin treatment for early syphilis is reported to range from 8% to 56%.

**Objectives:**

To prospectively assess the incidence of JHR signs and symptoms among adults with early syphilis treated with benzathine penicillin G and to document factors associated with JHR and benzathine penicillin G treatment response outcomes.

**Design, Setting, and Participants:**

The main study was designed as a phase 4 randomized clinical trial to compare the treatment efficacy of 1 vs 3 doses of benzathine penicillin G in adults with early syphilis, measured as serologic response at 6 months. A total of 249 adults with or without HIV were screened and enrolled between October 31, 2018, and March 3, 2020. Participants were screened and enrolled at 10 US study sites in the Sexually Transmitted Infections Clinical Trials Group. Statistical analysis for this secondary analysis took place between March 2023 and August 2024.

**Intervention:**

Participants received a first dose of benzathine penicillin G, 2.4 million units intramuscularly, at the enrollment visit. The JHR assessment window was day 1 to day 7 after the first dose of benzathine penicillin G.

**Main Outcomes and Measures:**

Primary outcomes in this study were the incidence of symptoms consistent with JHR within 7 days after benzathine penicillin G treatment. Unelicited and elicited symptoms were assessed by participant self-report using a standardized checklist during contact made by a study clinician. Factors associated with JHR were collected at baseline, and serologic treatment response was assessed at 6 months. Posttreatment incident JHR symptoms were captured as safety outcomes for this trial. Analysis was performed on an intention-to-treat basis.

**Results:**

Of 249 participants, the median age was 32 years (IQR, 27-41 years), 242 (97.2%) were men, and 153 (61.4%) were living with HIV. One or more JHR symptoms occurred in 59 participants (23.7%) treated for early syphilis, with a median symptom onset at 4.9 hours (IQR, 3.0-9.2 hours) and a median duration of 12.8 hours (IQR, 5.0-24.0 hours). Symptom onset was within 12 hours of treatment for 49 of 57 participants (86.0%). Among 59 symptomatic participants, myalgias (30 [50.8%]), chills (27 [45.8%]), weakness (23 [39.0%]), and feverishness (21 [35.6%]) were most common. In adjusted models, JHR was associated with secondary syphilis (adjusted odds ratio [AOR], 2.91 [95% CI, 1.51-5.61]) and the absence of HIV (AOR for living with HIV, 0.49 [95% CI, 0.26-0.94]). The proportion of participants with a serologic treatment response to benzathine penicillin G at 6 months was higher among participants with JHR (84.7% [50 of 59] vs 68.9% [131 of 190] without JHR).

**Conclusions and Relevance:**

In this prespecified secondary analysis of a randomized clinical trial of early syphilis treatment wtih benzathine penicillin G in adults, approximately 1 in 4 participants experienced short-lived JHR symptoms, which were associated with secondary syphilis stage, lack of HIV, and successful treatment outcomes at 6 months. These messages could be used in patient counseling.

**Trial Registration:**

ClinicalTrials.gov Identifier: NCT03637660

## Key Points

**Question:**

What proportion of patients with early syphilis have symptoms of the Jarisch-Herxheimer reaction (JHR) after treatment with benzathine penicillin G, and what are the associated characteristics?

**Findings:**

This prespecified secondary analysis of a multisite US randomized clinical trial found that, among 249 adults with early syphilis, JHR symptoms occurred after benzathine penicillin G treatment in 59 participants (23.7%). Onset of JHR was within 12 hours of treatment for 86.0% of participants, and JHR was associated with successful treatment response, secondary syphilis, and absence of HIV.

**Meaning:**

This study suggests that patients treated with benzathine penicillin G for early syphilis should be counseled about the potential of developing short-lived JHR symptoms within 24 hours that may be associated with successful treatment response.

## Teaser

This secondary analysis of a randomized clinical trial assesses the incidence of Jarisch-Herxheimer reaction among adults with early syphilis treated with benzathine penicillin G and the factors associated with Jarisch-Herxheimer reaction and benzathine penicillin G treatment response outcomes.

## Introduction

The Jarisch-Herxheimer reaction (JHR) describes a set of signs and symptoms that can occur after treatment of *Treponema pallidum* subsp *pallidum* (syphilis) and other infections caused by spirochetal bacteria. It was first described in the prepenicillin era by Adolf Jarisch in 1895, then by Herxheimer and Krause in 1902.^1,2,3^ In addition to occurring after syphilis, the JHR can occur after treatment of other systemic spirochetal infections, including leptospirosis (*Leptospira interrogans*), Lyme disease (*Borrelia burgdorferi*), and tick-borne relapsing fever (other *Borrelia* species).^4,5,6^ The incidence of JHR after penicillin treatment among adult and pediatric patients with early syphilis (primary, secondary, or early latent) ranges from 8% to 56%.^7,8^

The reported composition of JHR-associated signs and symptoms is variable and relatively nonspecific. Common elements include fever, chills, malaise, or worsening of syphilitic skin rash that occurs hours after treatment, although the prevalence of specific symptoms is not well described. Symptoms of JHR are often self-limited and mild to moderate, although hemodynamic changes and leukocytosis can occur, and JHR during pregnancy can lead to nonreassuring fetal status.^9^ The pathogenesis of JHR is not well understood. Symptoms have been attributed to cytokine release during treatment of *T pallidum*.^2,10,11,12,13^ Few studies have focused on identifying individual-level factors associated with JHR in syphilis (age, syphilis stage, history of syphilis, and HIV status) to provide insight into underlying immune mechanisms.^14,15^ Several studies have suggested a potential association between JHR and successful treatment response to penicillin therapy.^16,17,18^

This prespecified secondary analysis was designed to prospectively assess the incidence of JHR signs and symptoms among adults with a diagnosis of active, early syphilis who were treated with standard benzathine penicillin G dosing of 2.4 million units (1 vs 3 doses) as part of a phase 4 multicenter US randomized clinical trial (RCT). Unadjusted and adjusted models were created to assess participant-level factors that were independently associated with JHR after benzathine penicillin G treatment with stratification by HIV status. The association between JHR and benzathine penicillin G serologic treatment response at 6 months was also evaluated.

## Methods

### Study Design

The incidence of JHR symptoms, along with associated factors and treatment outcomes, were assessed as part of the safety analysis plan for a phase 4 RCT with a primary outcome of comparing the treatment efficacy of 1 vs 3 doses of benzathine penicillin G among adults with early syphilis, measured as serologic response at 6 months. The study was conducted as part of the National Institute of Allergy and Infectious Diseases Sexually Transmitted Infections (STIs) Clinical Trials Group and enrolled 249 adults at 10 US study sites between October 31, 2018, and March 3, 2020^19^ (NCT03637660; trial protocol and statistical analysis plan in Supplement 1). All study participants provided written informed consent, and the study was approved by the local institutional review board (IRB) at each study site (University of Alabama at Birmingham IRB, Emory University IRB, Indiana University IRB, University of Washington IRB, Beth Israel Deaconess Medical Center Committee on Clinical Investigations, Wake Forest University IRB, University of North Carolina at Chapel Hill IRB, Louisiana State University Health Sciences Center IRB, University of Pittsburgh IRB, and Johns Hopkins IRB). This study followed the Consolidated Standards of Reporting Trials (CONSORT) reporting guideline.

### Patients

Adult participants with or without HIV were eligible to participate if they had clinician-staged, untreated early syphilis with positive results of quantitative rapid plasma reagin (RPR) and treponemal antibody (*T pallidum* particle agglutination) serologic testing consistent with acute infection acquired in the previous 12 months. People who had received antibiotics with activity against *T pallidum* in the past 30 days were excluded from participation.

### Intervention

Participants were randomized to receive 1 vs 3 weekly doses of benzathine penicillin G, 2.4 million units intramuscularly, at baseline. The JHR assessment window was day 1 to day 7 after benzathine penicillin G dose 1, with information collected by telephone call, text message, email, or in person.

### Outcome Measures

Primary outcomes in this study were the incidence and assessment of symptoms consistent with JHR within 7 days after benzathine penicillin G treatment. Unelicited and elicited symptoms were assessed by participant self-report with a standardized checklist during contact made by a study clinician. Initial study procedures conducted 24 to 72 hours after treatment were designed to improve recall of specific symptom details.

Participants were queried about individual JHR symptoms, timing of symptom onset after benzathine penicillin G treatment, symptom severity (classified as mild, moderate, or severe), and duration. Severe symptoms were defined as preventing daily activity or requiring medical care. Specific elicited JHR symptoms were subjective feverishness or objective fever (including highest temperature, if available), chills, myalgias, weakness, flushing, worsening of rash, tachycardia, cardiac palpitations, generalized arthralgias, nausea, headache, dizziness, and “other” as described in free text. Participants were also asked if they took any medication to relieve symptoms or sought medical attention. Adverse event forms were reviewed for possible JHR symptoms that were not reported elsewhere. If a participant was not reached by telephone call, text message, or email, the JHR questionnaire was completed at the subsequent in-person follow-up study visit scheduled at day 7 after enrollment. For the syphilis treatment response outcome, the standard clinical definition was used: at least a 4-fold decrease in RPR titer or reversion to nonreactive at 6 months after treatment.

### Statistical Analysis

Statistical analysis for this secondary analysis took place between March 2023 and August 2024 using SAS, version 9.4 (SAS Institute). Analyses were performed for all study participants who were enrolled and defined as the intention-to-treat population who received at least 1 dose of benzathine penicillin G per protocol. The association of JHR with age, HIV status (according to testing reported at baseline or during follow-up), history of syphilis, syphilis stage, and baseline RPR titer was assessed by logistic models. Symptoms were grouped by frequency to look for patterns with multiple symptoms. Univariate models were fit for each independent variable, and a multivariable model was fit with all variables. All *P* values were from 2-sided tests, and results were deemed statistically significant at *P* < .05.

## Results

### Patients

Of 249 participants (median age, 32 years [IQR, 27-41 years]; 242 men [97.2%] and 7 women [2.8%]; 4 Asian participants [1.6%], 154 Black participants [61.8%], 12 Hispanic participants [4.8%], 63 White participants [25.3%], and 28 participants of other race or ethnicity [11.2%]), 223 (89.6%) reported recent sex with men (Table 1). Syphilis was staged as 19.4% primary (48 of 248), 47.2% secondary (117 of 248), and 33.5% early latent (83 of 248). Only 22 participants (8.8%) reported a known history of syphilis. The median quantitative RPR titer at baseline was 1:64 (IQR, 1:32-1:128), and 77.5% of participants (193 of 249) had an RPR titer of 1:32 or greater at enrollment. As anticipated based on case definitions and clinical presentation, significant overlap was noted between secondary syphilis staging, presence of a lesion or rash, and high RPR titer (≥1:32) among the 117 participants with secondary syphilis; 99 participants (84.6%) had all 3 at baseline. A total of 153 participants (61.4%) were persons living with HIV (PLWH). An additional 5 participants had unknown HIV status at baseline, 4 of whom tested negative for HIV during follow-up visits. Among PLWH with recent CD4 testing, 77.5% (110 of 142) had a CD4 count of 350 cells/mm^3^ or more (median, 539 cells/mm^3^ [IQR, 393-806 cells/mm^3^]), and 92.2% (141 of 153) reported receiving antiretroviral therapy. Recent HIV viral load data were available for a subset of participants; 54.2% (26 of 48) had a viral load less than 200 copies/mL (median viral load, 143 copies/mL [IQR, 42-13 850 copies/mL]).

**Table 1.** Characteristics of Participants With Early Syphilis by Symptomatic JHR After Benzathine Penicillin G Treatment

Characteristic	No. (%)		
	After treatment		Total (N = 249)
	JHR (n = 59)	No JHR (n = 190)	
Age, y			
18-29	26 (44.1)	67 (35.3)	93 (37.3)
30-39	16 (27.1)	67 (35.3)	83 (33.3)
40-49	8 (13.6)	30 (15.8)	38 (15.3)
≥50	9 (15.3)	26 (13.7)	35 (14.1)
Sex at birth			
Male	57 (96.6)	185 (97.4)	242 (97.2)
Female	2 (3.4)	5 (2.6)	7 (2.8)
Race and ethnicity by self-report			
Asian	0	4 (2.1)	4 (1.6)
Black	26 (44.1)	128 (67.4)	154 (61.8)
Hispanic	3 (5.1)	9 (4.7)	12 (4.8)
White	26 (44.1)	37 (19.5)	63 (25.3)
Other^a^	7 (11.9)	21 (11.1)	28 (11.2)
Recent sex partners			
Men only	46 (78.0)	144 (75.8)	190 (76.3)
Women only	4 (6.8)	17 (8.9)	21 (8.4)
Men and women	7 (11.9)	26 (13.7)	33 (13.3)
Other	2 (3.4)	3 (1.6)	5 (2.0)
Syphilis stage (n = 248)^b^			
Primary	9 (15.3)	39 (20.6)	48 (19.4)
Secondary	39 (66.1)	78 (41.3)	117 (47.2)
Early latent	11 (18.6)	72 (38.1)	83 (33.5)
RPR titer (n = 240)^c^			
1:1-1:4	2 (3.4)	13 (7.1)	15 (6.3)
1:8-1:16	8 (13.8)	24 (13.2)	32 (13.3)
1:32-1:64	21 (36.2)	79 (43.4)	100 (41.7)
1:128-1:512	27 (46.6)	66 (36.3)	93 (38.8)
Visible lesion or rash	47 (79.7)	118 (62.1)	165 (66.3)
Reported history of syphilis			
Ever	2 (3.4)	15 (7.9)	17 (6.8)
Past 12 mo	0	5 (2.6)	5 (2.0)
History of other STI (ever)			
* Chlamydia trachomatis*	2 (3.4)	12 (6.3)	14 (5.6)
* Neisseria gonorrhoeae*	1 (1.7)	9 (4.7)	10 (4.0)
Benzathine penicillin G response at 6 mo^d^	50 (84.7)	131 (68.9)	181 (72.7)
Persons living with HIV	30 (50.8)	123 (64.7)	153 (61.4)
CD4 count, cells/mm^3^, No./total No. (%)			
<200	4/30 (13.3)	8/123 (6.5)	12/153 (7.8)
200-349	4/30 (13.3)	16/123 (13.0)	20/153 (13.1)
≥350	22/30 (73.3)	88/123 (71.5)	110/153 (71.9)
Missing	0/30	11/123 (8.9)	11/153 (7.2)

Abbreviations: JHR, Jarisch-Herxheimer reaction; RPR, rapid plasma reagin; STI, sexually transmitted infection.

^a^ Includes American Indian or Alaska Native, Native Hawaiian or Other Pacific Islander, multiracial, and unknown.

^b^ Based on intention-to-treat population analyzed per protocol. One participant lacked documentation of syphilis stage. All participants were considered to be serologic nonresponders for the primary outcome.

^c^ Based on intention-to-treat population analyzed per protocol. Nine participants lacked a valid RPR titer at baseline. All participants were considered to be serologic nonresponders for the primary outcome.

^d^ Defined serologically as at least a 4-fold decrease in RPR titer or seroreversion to nonreactive.

### Outcomes

**Figure 1.**
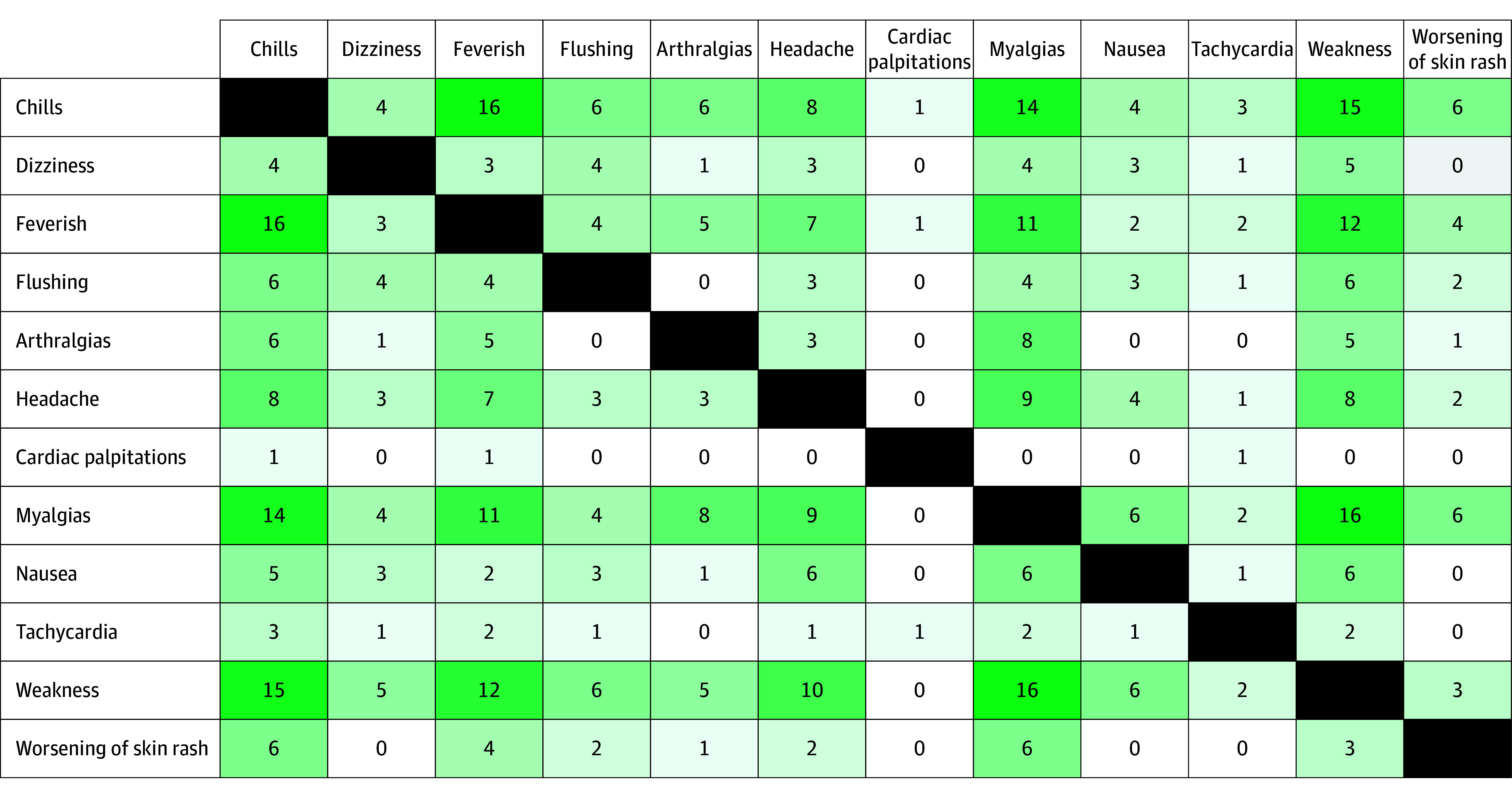
Constellation of Jarisch-Herxheimer Reaction Symptoms Among Symptomatic Participants (N = 59) The grading indicates the quantity of the event in relation to the others. The darker the green, the more likely the events were to have happened at the same time.

After benzathine penicillin G treatment, 23.7% of participants (59 of 249) experienced at least 1 symptom included in the JHR checklist (Table 2). Participants with JHR were disproportionately younger (aged 18-29 years), had secondary stage syphilis, had an RPR titer of 1:32 or more, were not living with HIV, and reported no history of syphilis. The most frequently reported symptoms were myalgias (30 of 59 [50.8%]), chills (27 of 59 [45.8%]), weakness (23 of 59 [39.0%]), feverishness (21 of 59 [35.6%]), and headache (17 of 59 [28.8%]). Most symptomatic participants (34 of 59 [57.6%]) reported 1 to 2 JHR symptoms, 69.5% (41 of 59) reported 2 or more symptoms, and 20.3% (12 of 59) reported 5 or more symptoms. Figure 1 demonstrates symptoms that co-occurred with others; chills were often accompanied by feverishness, weakness, and myalgias. The least frequently reported symptoms were palpitations (2 of 59 [3.4%]), tachycardia (4 of 59 [6.8%]), and dizziness (5 of 59 [8.5%]) (Table 2). Other related signs and symptoms documented as occurring shortly after treatment as part of adverse event assessment included chest pain, diarrhea, exfoliative generalized rash with swelling that was diagnosed as drug rash, facial weakness, and night sweats (1 case of each). Most JHR symptoms (52 of 59 [88.1%]) were mild to moderate, with a median time to onset of 4.9 hours (IQR, 3.0-9.2 hours) after receiving the benzathine penicillin G injection and a median symptom duration of 12.8 hours (IQR, 5.0-24.0 hours). Symptom onset was within 12 hours of treatment for 49 of 57 participants (86.0%). When stratified by HIV status, PLWH had a lower incidence of JHR symptoms (19.6% [30 of 153] vs 30.2% [29 of 96] of people without HIV) and a shorter median symptom duration (9.0 hours [IQR, 4.0-19.7 hours] among PLWH vs 18.0 hours [IQR, 7.0-26.0] among people without HIV). Among the symptoms elicited, only feverishness was slightly more common among PLWH; all other symptoms were more common among persons without HIV. There were no study terminations or hospitalizations attributed to JHR.

**Table 2.** Elicited Symptoms Among Participants With Early Syphilis After Benzathine Penicillin G

Symptom or characteristic	No. (%)		
	People with HIV (n = 153)	People without HIV (n = 96)	Total (N = 249)
Symptomatic	30 (19.6)	29 (30.2)	59 (23.7)
Symptom description^a^			
Feverish	14 (9.2)	7 (7.3)	21 (8.4)
Chills	13 (8.5)	14 (14.6)	27 (10.8)
Myalgias	15 (9.8)	15 (15.6)	30 (12.0)
Weakness	12 (7.8)	11 (11.5)	23 (9.2)
Flushing	3 (2.0)	6 (6.3)	9 (3.6)
Worsening of skin rash	4 (2.6)	7 (7.3)	11 (4.4)
Tachycardia	2 (1.3)	2 (2.1)	4 (1.6)
Cardiac palpitations	1 (0.7)	1 (1.0)	2 (0.8)
Arthralgias	4 (2.6)	4 (4.2)	8 (3.2)
Nausea	4 (2.6)	8 (8.3)	12 (4.8)
Headache	9 (5.9)	8 (8.3)	17 (6.8)
Dizziness	2 (1.3)	3 (3.1)	5 (2.0)
Characteristic			
No. of symptoms reported			
0	123 (80.4)	67 (69.8)	190 (76.3)
1	10 (6.5)	8 (8.3)	18 (7.2)
2	9 (5.9)	7 (7.3)	16 (6.4)
3-4	5 (3.3)	8 (8.3)	13 (5.2)
≥5	6 (3.9)	6 (6.3)	12 (4.8)
Symptom severity maximum (n = 59)			
Mild	8 (26.7)	18 (62.1)	26 (44.1)
Moderate	17 (56.7)	9 (31.0)	26 (44.1)
Severe	5 (16.7)	2 (6.9)	7 (11.9)
Time to symptom onset, median (IQR), h	4.6 (3.6-9.0)	5.0 (2.8-10.0)	4.9 (3.0-9.2)
Symptom onset after benzathine penicillin G (n = 57), h			
0-6	17 (43.6)	17 (60.7)	34 (59.6)
>6-12	7 (17.9)	8 (28.6)	15 (26.3)
>12-18	1 (2.6)	2 (7.1)	3 (5.3)
>18-24	3 (7.7)	1 (3.6)	4 (7.0)
>24-48	1 (2.6)	0	1 (1.8)
Duration of symptoms, median (IQR), h	9.0 (4.0-19.7)	18.0 (7.0-26.0)	12.8 (5.0-24.0)
Longest duration of symptoms (n = 58), h			
0-6	6 (20.0)	7 (25.0)	13 (22.4)
>6-12	6 (20.0)	4 (14.3)	10 (17.2)
>12-24	7 (23.3)	7 (25.0)	14 (24.1)
>24	11 (36.7)	10 (35.7)	21 (36.2)

^a^ Participants could have multiple symptoms.

**Figure 2.**
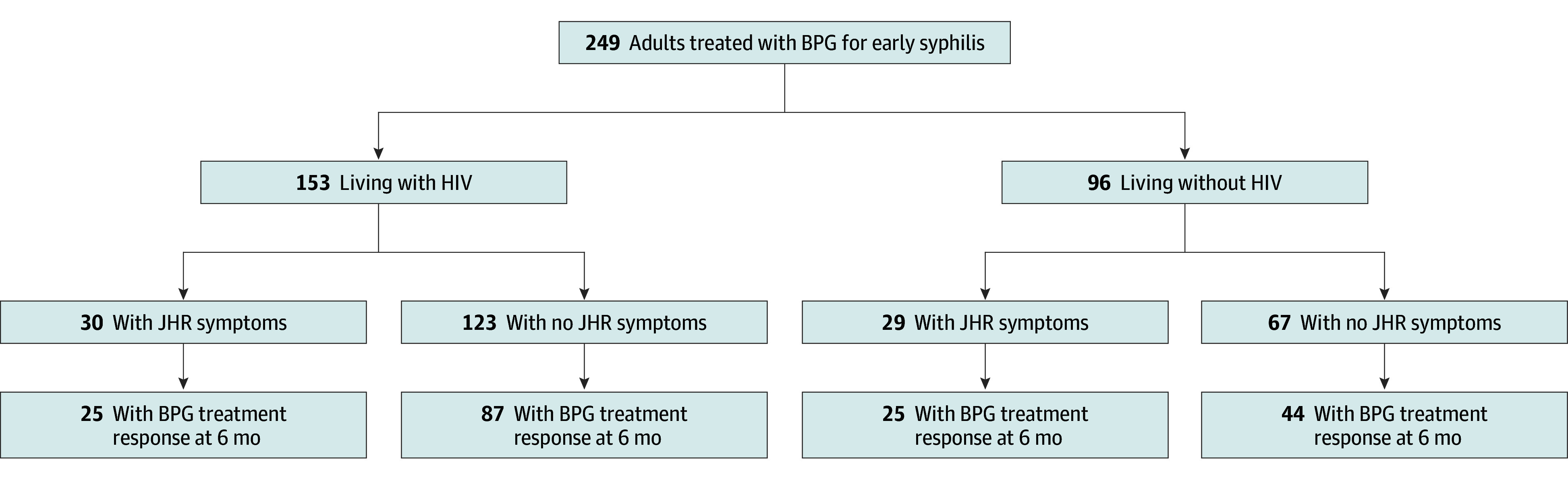
Flow Diagram by HIV Status, Jarisch-Herxheimer Reaction (JHR) Symptoms, and Treatment Response BPG indicates benzathine penicillin G.

In unadjusted and adjusted models for JHR after benzathine penicillin G treatment that included age, HIV status, history of syphilis, syphilis stage, and RPR titer of 1:32 or more, secondary stage syphilis was consistently associated with JHR (adjusted odds ratio [AOR], 2.91 [95% CI, 1.51-5.61]; *P* = .001) compared with primary or early latent stages. In the adjusted model, PLWH also had lower odds of JHR (AOR, 0.49 [95% CI, 0.26-0.94]; *P* = .03) compared with people without HIV (Table 3). A test for collinearity between variables was negative. In separate models stratified by HIV status, secondary syphilis was consistently associated with JHR. Lower CD4 count (<200 or <350 cells/mm^3^ vs ≥350 cells/mm^3^) was not associated with JHR among PLWH. A flow diagram (Figure 2) shows group characteristics stratified by HIV status, presence of JHR symptoms, and benzathine penicillin G treatment response at 6 months. The proportion of participants with serologic treatment response at 6 months was higher among participants with JHR (84.7% [50 of 59] vs 68.9% [131 of 190] without JHR; *P* = .01). This finding persisted among participants when compared by HIV status.

**Table 3.** Factors Associated With the Jarisch Herxheimer Reaction After Early Syphilis Treatment

Factor	Unadjusted OR (95% CI)	*P* value	Adjusted OR (95% CI)	*P* value
Age, y				
18-29	1.45 (0.80-2.62)	.22	1.02 (0.53-1.95)	.96
≥30	1 [Reference]		1 [Reference]	
Living with HIV	0.56 (0.31-1.02)	.06	0.49 (0.26-0.94)	.03
History of syphilis	0.30 (0.07-1.32)	.11	0.31 (0.07-1.40)	.13
Secondary stage of syphilis	2.78 (1.51-5.12)	.001	2.91 (1.51-5.61)	.001
RPR titer ≥1:32	1.23 (0.57-2.65)	.61	0.96 (0.42-2.21)	.93

Abbreviations: OR, odds ratio; RPR, rapid plasma reagin.

## Discussion

This secondary analysis conducted as part of a multicenter syphilis treatment trial allowed for the assessment of JHR symptoms and associated factors after benzathine penicillin G treatment in adults. In line with published data, typical JHR symptoms after treatment occurred in about 1 in 4 adults with early syphilis. In adjusted models, JHR was associated with secondary syphilis—a stage where high concentrations of *T pallidum* circulate 2 to 24 weeks after primary syphilis lesions heal. In this study, JHR was less common among PLWH compared with people without HIV. This finding confirms findings from Spain, where JHR incidence was 28% after early syphilis treatment, with lower rates observed among PLWH (29% vs 41% without HIV; *P* = .18).^20^ In contrast, Yang et al^14^ described higher JHR rates among PLWH (35% vs 25% without HIV; *P* = .09), and Tsai et al^15^ documented a JHR rate of 56% among PLWH in Taiwan. Differences in JHR by HIV status may reflect differential exposure to syphilis over time or unique host immunity in PLWH.

Most participants with JHR had 1 to 2 symptoms that started within 12 hours after treatment and lasted for 13 hours, on average. Among those with multiple symptoms, the most common constellation was feverishness, chills, weakness, and myalgias. Although a few participants reported severe JHR symptoms, there were no serious outcomes observed.^20^ A standardized JHR definition would be useful clinically and to compare rates and outcomes in future studies. Based on findings from this study and others, a standard JHR definition might include 2 or more generalized symptoms. Among symptomatic participants in this study, 69.5% would be included in this JHR definition.

In terms of participant characteristics, secondary syphilis was a key factor associated with JHR in this study and others. Secondary syphilis was the only factor associated with JHR in a retrospective study of 30 pregnant and nonpregnant women in Japan.^21^ In a univariate analysis of people with and without HIV, early-stage syphilis was also associated with JHR (82% vs 28%; *P* < .001).^14^ These observations vary from historical reports in which JHR was more common in later stages of syphilis.^22^ Contemporary study findings may contradict studies from the preantibiotic era because syphilis staging based on overlapping signs and symptoms is imprecise. Not only can syphilitic skin lesions occur on mucosal surfaces that are not readily observed, but the early latent stage can occur both before and after clinical manifestations of secondary syphilis appear,^23^ which makes it challenging to interpret JHR results stratified by syphilis stage. In principle, higher JHR incidence during early, active, disseminated infection compared with later stages with latent *T pallidum* infection is likely related to pathogenesis and the host immune response. Studies designed to characterize *T pallidum* pathogenesis and humoral and T-cell immunity during JHR are needed to elucidate underlying mechanisms.^7,13,20,24,25,26^

Although not documented in this study, syphilis treatment in the setting of high RPR titers (often defined as ≥1:32) has been associated with JHR among people with or without HIV.^14,15^ An RPR titer of 1:256 or higher was also associated with JHR among children treated for congenital syphilis.^27^ High RPR titers are common in secondary syphilis—a factor that was associated with JHR in our models. History of syphilis diagnosis was not associated with JHR in our study, yet it was negatively associated in Taiwan.^14,15^ Information for this variable in our study may have been limited by self-report among individuals with unrecognized prior syphilis infection (only 8.8% reported a known history of syphilis). History of syphilis is important to assess and include in future investigations because it may be associated with nonsterilizing immunity that could affect early immune responses.^28^

Study findings confirmed our hypothesis about a potential association between JHR and response to benzathine penicillin G treatment.^16,18^ In a prior study, Seña et al^18^ found that JHR was associated with treatment response (vs serofast treatment response) in some of the study population, although it was not statistically significant (odds ratio, 1.54 [95% CI, 0.95-2.52]). This association may be useful in terms of counseling patients about JHR symptoms as one factor associated with response. This finding warrants additional study to characterize underlying immune mechanisms associated with response to therapy.

### Strengths and Limitations

This study has some strengths, including the diversity of the population in terms of age, race and ethnicity, US region of residence, syphilis stage, HIV status, and RPR titers. The design of the multicenter RCT allowed for close follow-up and prospective data collection for a well-characterized study population with confirmed syphilis, disease staging by clinical experts, and directly observed therapy with follow-up shortly after treatment to reduce recall bias.

This study also has some limitations. It is not possible to comment on the factors associated with JHR in women or pregnancy due to the population enrolled in the trial. A strict definition of JHR is not currently available, symptoms are somewhat nonspecific, and there is no diagnostic test for JHR. In addition, the incidence of JHR among persons treated with antibiotics other than penicillin was not characterized. Other limitations of the dataset include the self-reported syphilis history and incomplete data on CD4 count, viral load, and antiretroviral therapy for PLWH.

In the future, the JHR clinical syndrome definition proposed in this study should be validated to allow for symptom assessment after syphilis treatment and to standardize comparison of JHR in research studies. As syphilis prevalence in the US continues to increase, clinicians may benefit from additional education about JHR after syphilis to manage patient expectations after treatment. New studies proposed to better define JHR and treatment outcomes should include women and gender-diverse individuals, pregnant persons, infants and children, participants with latent syphilis, global populations, and people taking doxycycline postexposure prophylaxis to prevent STIs. To better understand JHR pathogenesis, immune response studies are needed to characterize JHR at the cellular level in terms of bacterial burden and explore the association documented here between JHR and treatment response.

## Conclusions

In this prespecified secondary analysis of an RCT of JHR after intramuscular benzathine penicillin G treatment for early syphilis in adults, JHR symptoms occurred in 23.7% of participants within 24 to 48 hours. The median symptom onset was 4.9 hours, with a median duration of 12.8 hours. In adjusted models, JHR was associated with secondary syphilis stage and lack of HIV. Participants with JHR were more likely to have treatment response at 6 months. These messages could be used in patient counseling.

## Notes

Supplement 1.
Trial Protocol and Statistical Analysis Plan

Supplement 2.
Data Sharing Statement
