# Supplementary material for: Jarisch-Herxheimer Reaction After Benzathine Penicillin G Treatment in Adults With Early Syphilis: Secondary Analysis of a Randomized Clinical Trial
Source: JAMA Netw Open. 2025 Feb 13;8(2):e2459490. doi: 10.1001/jamanetworkopen.2024.59490 (PMC11826359; doi:10.1001/jamanetworkopen.2024.59490)
Supplement: Supplement 1. — Trial Protocol and Statistical Analysis Plan [file jamanetwopen-e2459490-s001.pdf]

# A Phase 4 Comparative Trial of Benzathine Penicillin G 2.4 Million Units Administered as a Single Dose versus Three Successive Weekly Doses for Treatment of Early Syphilis in Subjects with or without HIV Infection

**DMID Protocol Number:** 17-0101

**DMID Funding Mechanism:** HHSN272201300012I; HHSN27200014

**Principal Investigator:** Edward W. Hook, III, MD

**DMID Clinical Project Manager:** Delmyra Turpin, RN, MPH, CCRP

**DMID Medical Monitor:** Jorge Mejia-Galvis, MD

**DMID Medical Officer:** Lori Newman, MD

**DMID Scientific Lead:** Carolyn Deal, PhD

**Version Number:** 4.0

**22 June 2020**

# STATEMENT OF COMPLIANCE

The study will be carried out in accordance with Good Clinical Practice (GCP) as required by:

- United States (US) Code of Federal Regulations (CFR) applicable to clinical studies (45 CFR Part 46, 21 CFR Part 50, 21 CFR Part 56, and 21 CFR Part 312)
- International Conference on Harmonisation: Good Clinical Practice (ICH) E6; 62 Federal Register 25691 (1997); and future revisions
- National Institutes of Health (NIH) Clinical Terms of Award

All key personnel (all individuals responsible for the design and conduct of this study) have completed Human Subjects Protection Training.

# SIGNATURE PAGE

The signature below constitutes the approval of this protocol and the attachments and provides the necessary assurances that this trial will be conducted according to all stipulations of the protocol, including all statements regarding confidentiality, and according to local legal and regulatory requirements and applicable US federal regulations and ICH guidelines.

Principal Investigator:

Signed: ______________________________ Date: ____________

Edward W. Hook, III, MD
University of Alabama at Birmingham

Site Investigator:

Signed: ______________________________ Date: ____________

Teresa A. Batteiger, MD
Indiana University

Site Investigator:

Signed: ______________________________ Date: ____________

Jodie A. Dionne-Odom, MD
University of Alabama at Birmingham

Site Investigator:

Signed: ______________________________ Date: ____________

Julia C. Dombrowski, MD, MPH
University of Washington

Site Investigator:

Signed: ______________________________ Date: ____________

Kenneth H. Mayer, MD
Fenway Health

Site Investigator:

Signed: ______________________________ Date: ______________
Candice J. McNeil, MD, MPH
Wake Forest University Health Sciences

Site Investigator:

Signed: ______________________________ Date: ______________
Arlene C. Seña, MD, MPH
University of North Carolina at Chapel Hill

Site Investigator:

Signed: ______________________________ Date: ______________
Stephanie N. Taylor, MD
Louisiana State University Health Sciences Center

Site Investigator:

Signed: ______________________________ Date: ______________
Harold C. Wiesenfeld, MD, CM
University of Pittsburgh

Site Investigator:

Signed: ______________________________ Date: ______________
Kimberly A. Workowski, MD
Emory University

Site Investigator:

Signed: ______________________________ Date: ______________
Jonathan M. Zenilman, MD
Johns Hopkins University

## TABLE OF CONTENTS

Page
Statement of Compliance 2
Signature Page 3
Table of Contents 5
List of Abbreviations 8
Protocol Summary 10
1 Key Roles 13
2 Background Information and Scientific Rationale 16
2.1 Background Information ....16
2.2 Rationale ....17
2.3 Potential Risks and Benefits ....17
2.3.1 Potential Risks ....17
2.3.2 Known Potential Benefits....18
3 Objectives 19
3.1 Study Objectives....19
3.1.1 Primary Objective....19
3.1.2 Secondary Objectives ....19
3.2 Study Outcome Measures ....19
3.2.1 Primary Outcome Measure....19
3.2.2 Secondary Outcome Measures ....19
4 Study Design 20
5 Study Enrollment and Withdrawal 22
5.1 Subject Inclusion Criteria ....22
5.2 Subject Exclusion Criteria ....23
5.3 Treatment Assignment Procedures....24
5.3.1 Randomization Procedures ....24
5.3.2 Masking Procedures....24
5.3.3 Reasons for Withdrawal from Study Participation/Discontinuation of Study Treatment....24
5.3.4 Handling of Withdrawals from Study Participation/Discontinuations of Study Treatment....25
5.3.5 Termination of Study ....26
6 Study Intervention/Investigational Product 27
6.1 Study Product Description ....27
6.1.1 Acquisition....27
6.1.2 Formulation, Packaging, and Labeling....27
6.1.3 Product Storage and Stability ....27
6.2 Dosage, Preparation, and Administration of Study Intervention/Investigational Product ....27
6.3 Accountability Procedures for the Study Intervention/Investigational Product(s) ...28

6.4 Assessment of Subject Compliance with Study Intervention/Investigational Product ....28
6.5 Concomitant Medications/Treatments ....29
7 Study Schedule 30
7.1 Visit 1 – Screening and Enrollment/Baseline Visit (Day 1) ....30
7.2 Follow-up ....31
7.2.1 Visit 2 – Week 1 (Day 7 to 13) ....31
7.2.2 Visit 3 – Week 2 (6 to 12 days after Visit 2) ....32
7.2.3 Visit 4 – Month 1 (Day 30 ± 7 days) ....33
7.2.4 Visit 5 – Month 3 (Day 90 ± 21 days) ....33
7.2.5 Visit 6 – Month 6 (Day 180 ± 21 days) ....34
7.2.6 Visit 7 – Month 9 (Day 270 ± 28 days) ....35
7.2.7 Visit 8 – Month 12 (Day 360 ± 28 days) (Final Study Visit) ....35
7.3 Early Termination Visit ....36
7.4 Unscheduled Visit ....36
8 Study Procedures/Evaluations 38
8.1 Clinical Evaluations ....38
8.2 Laboratory Evaluations ....39
8.2.1 Clinical Laboratory Evaluations ....39
8.2.2 Special Assays or Procedures ....39
8.2.3 Specimen Preparation, Handling, and Shipping ....39
9 Assessment of Safety 40
9.1 Specification of Safety Parameters ....40
9.2 Methods and Timing for Assessing, Recording, and Analyzing Safety Parameters ....40
9.2.1 Adverse Events ....40
9.2.2 Reactogenicity ....41
9.2.3 Serious Adverse Events ....41
9.3 Reporting Procedures ....42
9.3.1 Serious Adverse Events ....42
9.3.2 Regulatory Reporting for Studies Not Conducted Under DMID-Sponsored IND ....43
9.3.3 Reporting of Pregnancy ....43
9.4 Type and Duration of Follow-up of Subjects after Adverse Events ....44
9.5 Halting Rules ....44
9.6 Safety Oversight (ISM plus DSMB) ....45
10 Clinical Monitoring 46
10.1 Site Monitoring Plan ....46
11 Statistical Considerations 47
11.1 Study Hypotheses ....47
11.2 Sample Size Considerations ....47
11.3 Planned Interim Analyses ....48
11.3.1 Safety Review ....48

11.3.2 Immunogenicity or Efficacy Review ....48
11.4 Final Analysis Plan ....49
11.4.1 Analysis Populations ....49
11.4.2 Baseline Characteristics ....49
11.4.3 Safety Analysis Plan ....50
11.4.4 Efficacy Analysis Plan ....50
12 Source Documents and Access to Source Data/Documents 53
13 Quality Control and Quality Assurance 54
14 Ethics/Protection of Human Subjects 55
14.1 Ethical Standard ....55
14.2 Institutional Review Board ....55
14.3 Informed Consent Process ....55
14.4 Exclusion of Women, Minorities, and Children (Special Populations) ....56
14.5 Subject Confidentiality ....56
14.6 Study Discontinuation ....57
14.7 Future Use of Stored Specimens ....58
15 Data Handling and Record Keeping 59
15.1 Data Management Responsibilities ....59
15.2 Data Capture Methods ....59
15.3 Types of Data ....60
15.4 Timing/Reports ....60
15.5 Study Records Retention ....60
15.6 Protocol Deviations ....60
16 Publication Policy 62
17 Literature References 63
Appendix A: Schedule of Events 64
Appendix B: Table for Grading the Severity of Adverse Events 66

## LIST OF ABBREVIATIONS

| | |
|---|---|
| AE | Adverse Event/Adverse Experience |
| BPG | Benzathine Penicillin G |
| CFR | Code of Federal Regulations |
| CMS | Clinical Material Services |
| CoC | Certificate of Confidentiality |
| CROMS | Clinical Research Operations and Management Support |
| CSR | Clinical Study Report |
| DFA-TP | Direct Fluorescence Antibody-*Treponema pallidum* |
| DHHS | Department of Health and Human Services |
| DMID | Division of Microbiology and Infectious Diseases |
| DSMB | Data and Safety Monitoring Board |
| eCRF | Electronic Case Report Form |
| EMR | Electronic Medical Record |
| FDA | Food and Drug Administration |
| FWA | Federal Wide Assurance |
| GCP | Good Clinical Practice |
| HIV | Human Immunodeficiency Virus |
| IATA | International Air Transport Association |
| ICF | Informed Consent Form |
| ICH | International Conference on Harmonisation |
| ICON | ICON Clinical Research, Inc. |
| IDES | Internet Data Entry System |
| IM | Intramuscular |
| IND | Investigational New Drug Application |
| IRB | Institutional Review Board |
| ISM | Independent Safety Monitor |
| ITT | Intent-to-Treat |
| IV | Intravenous |
| MedDRA® | Medical Dictionary for Regulatory Activities |
| MOP | Manual of Procedures |
| MSM | Men who have Sex with Men |
| MU | Million Units |
| N | Number (typically refers to subjects) |
| NAT | Nucleic Acid Test |
| NIAID | National Institute of Allergy and Infectious Diseases |
| NIH | National Institutes of Health |
| OER | Office of Extramural Research, NIH, DHHS |
| OHRP | Office for Human Research Protections |
| P&S | Primary and Secondary |
| PASS | Power Analysis and Sample Size Software |
| PCR | Polymerase Chain Reaction |

| | |
|---|---|
| PI | Principal Investigator |
| RCT | Randomized Controlled Trial |
| RPR | Rapid Plasma Reagin |
| QA | Quality Assurance |
| QC | Quality Control |
| SAE | Serious Adverse Event/Serious Adverse Experience |
| SAS | Statistical Analysis System |
| SDCC | Statistical and Data Coordinating Center |
| SOP | Standard Operating Procedure |
| STI | Sexually Transmitted Infection |
| STI CTG | Sexually Transmitted Infections Clinical Trials Group |
| STS | Serological Test for Syphilis |
| TRI | Technical Resources International, Inc. |
| US | United States |

# PROTOCOL SUMMARY

| | |
|---|---|
| **Title:** | A Phase 4 Comparative Trial of Benzathine Penicillin G 2.4 Million Units Administered as a Single Dose versus Three Successive Weekly Doses for Treatment of Early Syphilis in Subjects with or without HIV Infection |
| **Phase:** | 4 |
| **Population:** | Approximately 560 adults aged 18 years or older with untreated early (primary, secondary, or early latent) syphilis (to achieve 420 evaluable subjects) |
| **Number of Sites:** | Ten |
| **Study Duration:** | 48 months |
| **Subject Participation Duration:** | 12 months |
| **Description of Agent or Intervention:** | Benzathine penicillin G (BPG) 2.4 million units (MU) injected once (Arm 1) or weekly for three successive weeks (Arm 2) |

**Objectives:**

Primary:

- To compare the serological response to therapy in subjects with early (primary, secondary, or early latent) syphilis treated with BPG 2.4 MU once or weekly for three successive weeks

Secondary:

- To determine if the difference in response to therapy between treatment arms by Month 6 differs among subjects with or without HIV infection
- To determine the impact of multiple BPG injected doses on subject compliance with study product and adherence to the corresponding scheduled visits
- To determine the incidence and manifestations of the Jarisch-Herxheimer reaction among subjects treated for early syphilis with BPG
- To collect prospective data up to Month 12 on the serological response to therapy in subjects treated for early syphilis with either BPG regimen
- To compare epidemiological characteristics of early syphilis among subjects with or without HIV infection

| | |
|---|---|
| **Description of Study Design:** | BPG 2.4 MU injected either once (Arm 1) or weekly for three successive weeks (Arm 2) as determined by randomization, with follow-up visits at Months 1, 3, 6, 9, and 12 for safety, clinical, and laboratory assessments |
| **Estimated Time to Complete Enrollment:** | 36 months |

## Schematic of Study Design

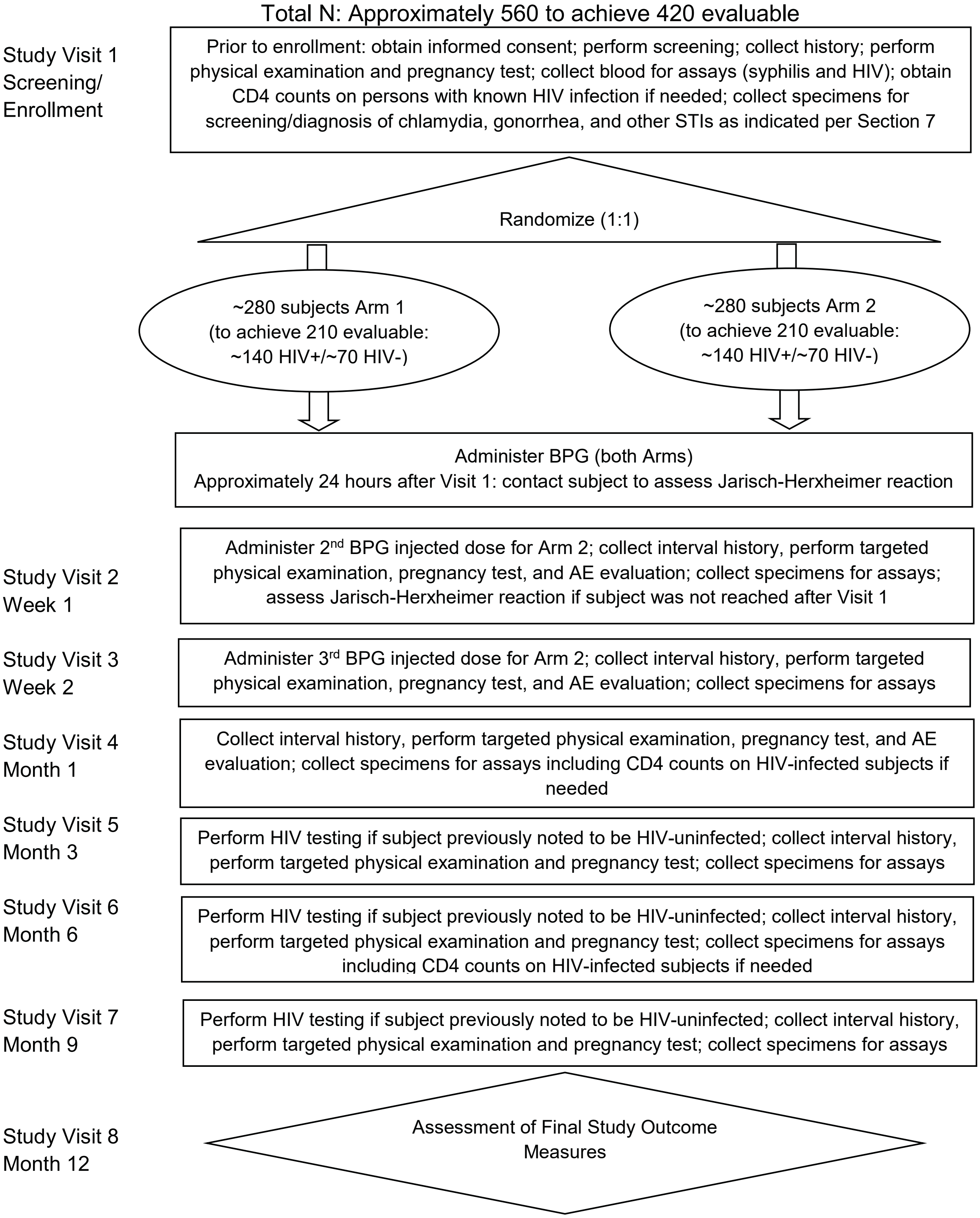

# 1 KEY ROLES

**Individuals:**

**Principal Investigator:**
Edward W. Hook, III, MD
The University of Alabama at Birmingham

**Site Investigator:**
Teresa A. Batteiger, MD
Indiana University

**Site Investigator:**
Jodie A. Dionne-Odom, MD
University of Alabama at Birmingham

**Site Investigator:**
Julia C. Dombrowski, MD, MPH
University of Washington

**Site Investigator:**
Kenneth H. Mayer, MD
Fenway Health

**Site Investigator:**
Candice J. McNeil, MD, MPH
Wake Forest University Health Sciences

**Site Investigator:**
Arlene C. Seña, MD, MPH
University of North Carolina at Chapel Hill

**Site Investigator:**
Stephanie N. Taylor, MD
Louisiana State University Health Sciences Center

**Site Investigator:**
Harold C. Wiesenfeld, MD
University of Pittsburgh, Magee-Women’s Hospital

**Site Investigator:**
Kimberly A. Workowski, MD
Emory University

**Site Investigator:**
Jonathan M. Zenilman, MD
Johns Hopkins University

**Institutions:**

**NIH – Division of Microbiology and Infectious Diseases**

**DMID/NIAID/NIH**
5601 Fishers Lane
Bethesda, MD 20892

**Scientific Lead**
Carolyn Deal, PhD

**Medical Officer**
Lori Newman, MD

**Medical Monitor**
Jorge Mejia-Galvis, MD

**Clinical Project Manager:**
Delmyra Turpin, RN, MPH, CCRP

**Statistical and Data Coordinating Center**

**The Emmes Company, LLC**
401 N. Washington St., Suite 700
Rockville, MD 20850
Phone: [redacted]
Fax: [redacted]
Email: [redacted]

**Project Manager**
Amy Price

**Statistician**
Nancy Hua, MS

| | |
|---|---|
| **Operations Coordinating Center** | **FHI 360**<br>359 Blackwell Street, Suite 200<br>Durham, NC 27701<br>Phone: [redacted]<br><br>**Protocol Specialist**<br>Charlotte Perlowski, MSPH<br><br>**Research Associate**<br>Ginger Pittman<br><br>**Research Assistant**<br>Shilysha Davis-Dublin |
| **UAMS STI CTG Statistical Consultant** | **University of Arkansas for Medical Sciences**<br>Jeannette Y. Lee, PhD |
| **Study Agent Repository** | **Fisher BioServices**<br>c/o DMID Clinical Materials Services<br>20439 Seneca Meadows Parkway<br>Germantown, MD 20876<br>Phone: [redacted]<br>Fax: [redacted]<br>Email: [redacted] |

# 2 BACKGROUND INFORMATION AND SCIENTIFIC RATIONALE

## 2.1 Background Information

Despite syphilis being a major public health problem for well over a century, and its treatment recommendations being unchanged for nearly 50 years, questions about syphilis and its management are amongst the most common questions to arise regarding sexually transmitted infections (STIs). The human immunodeficiency virus (HIV) epidemic has compounded these problems, as syphilis is now appreciated as both a risk marker and risk factor for HIV acquisition, and case reports and uncontrolled case series have led to recurring, unresolved questions regarding the optimal management of syphilis with respect to treatment regimens, serological follow-up, and risks for treatment failure [1]. Syphilis in the US is resurging, with rates of primary and secondary (P&S) syphilis increasing by 74% from 2012 to 2016 and disproportionately among HIV-infected persons [2]. Further rising P&S rates in women led to a 28% increase in congenital syphilis cases in the US between 2015 and 2016 [3]. No large prospective studies in the US have provided data on outcomes of recommended single-dose penicillin therapy versus widely-used but unstudied multiple-dose therapy for persons with early syphilis and HIV co-infection for over 30 years. In addition, there are no contemporary data describing serological responses to syphilis therapy at >6 months following therapy, raising questions as to how to best follow the ~20% of "serofast" patients who fail to serologically respond at >6 months following therapy. These questions represent a pressing public health need that is best addressed through a multicenter, randomized clinical trial (RCT).

Infections due to *Treponema pallidum* are the third most commonly reported infection in the US and are a global public health priority. Currently, P&S syphilis rates in the US are increasing faster than for any other reportable STI, and over half of P&S syphilis cases in men who have sex with men (MSM), the group that accounts for most new cases, are occurring among persons with HIV co-infection [3]. Untreated syphilis is a significant cause of morbidity in children born to mothers with untreated infection, may cause serious neurological or cardiovascular disease, and is both a proven risk factor and risk marker for HIV acquisition. Syphilis also remains a glaring example of STI health disparities, with P&S syphilis rates among African Americans in the US being more than six times those of non-Hispanic whites [4].

Penicillin is currently the only recommended treatment for syphilis (alternative therapies may be used in persons with penicillin allergy [5]). However, recent studies have shown that ~20% of persons treated for early syphilis with recommended, single-dose BPG 2.4 MU are "serofast" (do not show the desired 4-fold decline in serological test for syphilis titers 6 months following therapy) [5, 6], and concerns have been raised as to whether this regimen adequately treats early syphilis in HIV-infected persons. A well-conducted, multicenter RCT in the 1990s showed that higher doses of penicillin given in combination with the recommended BPG regimen did not

significantly change the serological response to therapy at 3, 6, or 12 months [7], but there are no RCTs evaluating whether a longer duration of BPG therapy improves the serological response to therapy among persons with or without HIV infection. While this earlier study addressed questions regarding daily penicillin doses for early syphilis treatment, the question of duration remains unresolved. As a result, there is substantial variation in the treatment of early syphilis, with many providers using multiple injected doses of BPG or treating serofast persons who have no evidence of active infection.

## 2.2 Rationale

Epidemiologically, syphilis infections tend to shift from one risk group to another. Past syphilis epidemics in the US have occurred in heterosexual men and women, or MSM. More recently, syphilis rates in the US have more than doubled over the past decade and now occur disproportionately among HIV-infected persons [4].

While penicillin is generally regarded as highly effective for syphilis treatment, whether or not currently recommended therapy is equally effective for persons with or without HIV infection remains unclear. In addition, recent appreciation of the fact that ~20% of persons treated for early syphilis are serofast has raised concerns regarding the significance of this observation [6]. Some clinicians believe that the serofast state signifies treatment failure, while others believe it represents failure to serologically respond to therapy in successfully treated patients. Each of these concerns may lead to inadequate therapy or over treatment in persons with early syphilis, as well as much confusion among clinicians about appropriate therapy.

The primary aims of this study are to determine whether subjects with or without HIV infection benefit from a longer duration of therapy (BPG 2.4 MU weekly for three successive weeks instead of as a single dose), and whether subjects with or without HIV infection differ in their response to BPG therapy for early syphilis.

## 2.3 Potential Risks and Benefits

### 2.3.1 Potential Risks

The risks associated with participation in the study are small. Penicillin is widely used to treat syphilis and other infections and is relatively well tolerated. All subjects in the trial will receive at least the currently recommended therapy. The most common adverse events (AEs) caused by penicillin include rash and hypersensitivity reactions. Therapeutic outcome is not a major concern, as all subjects will receive at least the Centers for Disease Control and Prevention-recommended dose of BPG for early syphilis. Subjects randomized to receive two additional injected doses of BPG will be at risk for additional discomfort and bruising at injection sites. Subjects may feel temporary pain or discomfort during blood draws, and there is a small risk of

infection at the site of the blood draw.

Information concerning subjects' sexual history is necessarily detailed given the study's objectives and may provoke some minor psychological or emotional stress when requested; subjects who receive a new diagnosis of an STI during the study may also experience minor psychological or emotional stress. Female subjects will be cautioned about the potential hazard of becoming pregnant and advised to use adequate birth control methods for the entire duration of their participation in the trial.

### 2.3.2 Known Potential Benefits

Subjects may benefit from this study by finding out more about the specific cause of symptoms they may be experiencing. Subjects may benefit from pre- and post-test counseling, treatment, and referrals, as necessary. It has previously been observed that subjects are empowered by knowing, for example, more about what syphilis is and how it can be prevented.

# 3 OBJECTIVES

## 3.1 Study Objectives

### 3.1.1 Primary Objective

- To compare the serological response to therapy in subjects with early (primary, secondary, or early latent) syphilis treated with BPG 2.4 MU once or weekly for three successive weeks

### 3.1.2 Secondary Objectives

- To determine if the difference in response to therapy between treatment arms by Month 6 differs among subjects with or without HIV infection
- To determine the impact of multiple BPG injected doses on subject compliance with study product and adherence to the corresponding scheduled visits
- To determine the incidence and manifestations of the Jarisch-Herxheimer reaction among subjects treated for early syphilis with BPG
- To collect prospective data up to Month 12 on the serological response to therapy in subjects treated for early syphilis with either BPG regimen
- To compare epidemiological characteristics of early syphilis among subjects with or without HIV infection

## 3.2 Study Outcome Measures

### 3.2.1 Primary Outcome Measure

- The proportion of subjects in each treatment group with a 4-fold decline in rapid plasma reagin (RPR) titers or seroreversion, simultaneously measured in sera collected throughout study participation at a single reference laboratory, by Month 6

### 3.2.2 Secondary Outcome Measures

- The proportion of subjects in each treatment group with a 4-fold decline in RPR titers or seroreversion by Month 6 among subjects with or without HIV infection
- The proportion of subjects in each treatment group with a 4-fold decline in RPR titers or seroreversion by Month 12
- The proportion of subjects in each treatment group with a 4-fold decline in RPR titers or seroreversion by Month 12 among subjects with or without HIV infection
- The proportion of subjects (overall and in each treatment group) who receive all assigned doses within the assigned visit windows
- The proportion of subjects in each treatment group who report Jarisch-Herxheimer reaction manifestations (fever, intensification of rash, myalgia, and other systemic symptoms) occurring within approximately 24 hours of initial BPG administration
- Demographics, sexual history, and socio-epidemiologic characteristics at baseline and sexual history through Months 6 and 12 among subjects with or without HIV infection and overall

# 4 STUDY DESIGN

This is a Phase 4, open-label, multicenter trial to evaluate the efficacy of a single injected dose of BPG 2.4 MU (Arm 1) compared to three successive weekly injected doses of BPG 2.4 MU (Arm 2) for treatment of early syphilis in HIV-infected and HIV-uninfected subjects. Subjects will be aged 18 years or older with untreated early syphilis (primary, secondary, or early latent as defined in prior syphilis treatment trials of the Sexually Transmitted Infections Clinical Trials Group (STI CTG)). The study will be conducted at 10 sites in the US and will enroll approximately 560 subjects to achieve 420 evaluable.

The study will involve a screening/enrollment visit and seven scheduled follow-up visits (Weeks 1 and 2, and Months 1, 3, 6, 9, and 12) over a 12-month period. At the enrollment visit, after providing informed consent, all subjects will undergo a brief sexual and medical history and a directed physical examination; all women of childbearing potential will have a urine or serum pregnancy test performed as part of qualification for study participation; all subjects will undergo phlebotomy for serological testing for syphilis and HIV according to the study and clinic protocols. All subjects who have not been tested for chlamydia and gonorrhea since last sexual activity and all subjects who have been sexually active in the past 14 days will have specimens collected for chlamydia and gonorrhea testing. Subjects will have specimens collected for other STI testing as indicated by local standard of care and subject history. All eligible subjects will be randomized to Arm 1 or Arm 2 and will receive an injected dose of BPG 2.4 MU.

Study personnel will attempt to contact subjects approximately 24 hours after Visit 1 to assess for symptoms of a Jarisch-Herxheimer reaction as described in Section 8.1. At the second and third visits (Weeks 1 and 2 of follow-up), subjects randomized to Arm 2 will receive injected doses of BPG 2.4 MU.

At all follow-up visits, subjects will have an interval history obtained, undergo a directed physical examination, and have repeat specimens collected for STI testing (based on the subject history and clinic protocols). At all follow-up visits, subjects will undergo phlebotomy for serological testing for syphilis and serum storage. At the Month 3, Month 6, Month 9, and Month 12 follow-up visits, consenting HIV-uninfected subjects will be tested for HIV infection using a 4$^{th}$ generation serological test for HIV. At Visit 1, study staff will swab consenting subjects' oral cavities and primary or secondary lesions (if lesions are present per Section 5.1); see Section 8.2.2.

Safety will be measured by subject report and physical examination (including vital signs: temperature, heart rate, respiration rate, and blood pressure; and genital, rectal, oral, skin, and lymph node examinations). All AEs (including solicited reactogenicity AEs and other unsolicited AEs) will be recorded through Month 1. Safety oversight will be provided by a

Data and Safety Monitoring Board (DSMB) and site Independent Safety Monitors (ISMs) as described in Section 9.6.

While sites will perform their own serological testing as needed for subject management, serological testing to determine study outcomes and serum banking will be performed at the Central Laboratory (University of Alabama at Birmingham).

The duration of the study for each subject will be 12 months. Enrollment is expected to be completed in 36 months. For additional details on study procedures/evaluations and study schedule by study visits, see Sections 7 and 8 and Appendix A.

# 5 STUDY ENROLLMENT AND WITHDRAWAL

To achieve 420 evaluable subjects, approximately 560 adults aged 18 years or older who meet all inclusion criteria and no exclusion criteria will be enrolled. No exemptions are granted on inclusion/exclusion criteria in trials sponsored by the Division of Microbiology and Infectious Diseases (DMID). Subjects may be recruited from STI clinics, HIV clinics, student health centers, and the public via advertising (e.g., flyers, radio, newspaper, social media). To enhance recruitment and retention, sites may seek institutional review board (IRB) permission to contact subjects by phone, text messaging, and/or email as appropriate. Any communication with subjects must be made using devices, methods, and services allowed by the local IRB and must be documented appropriately. As noted in Section 7.1, each subject may indicate his/her preferred method of contact to the study staff.

## 5.1 Subject Inclusion Criteria

Subjects eligible to enroll in this study must meet all inclusion criteria:

1. Subject is aged 18 years or older.
2. Subject has provided informed consent.
3. Subject has untreated primary*, secondary**, or early latent*** syphilis.
   *Primary syphilis is characterized by the presence of an ulcerative lesion at a potential site of inoculation (while classically solitary, shallow, painless and with an indurated, clean base, primary lesions may be multiple, may vary considerably in appearance, and/or may not be painless) or by darkfield, acceptable polymerase chain reaction (PCR), or direct fluorescence antibody-*T. pallidum* (DFA-TP) positive ulcers.
   **Secondary syphilis is characterized by classical palmar/plantar rash, condylomata lata, mucous patches, etc. or by darkfield, acceptable PCR, or DFA-TP positive lesions.
   ***Early latent syphilis is characterized by current reactive serologic tests for syphilis (STS) and a documented non-reactive STS, or documented sexual exposure to an individual known to have primary, secondary, or early latent syphilis diagnosed within the last 12 months.
4. Subject either has a newly reactive non-treponemal test (such as an RPR test) or a history of syphilis and a current increase in RPR titer of two or more dilutions (i.e., four-fold).
5. If subject is of childbearing potential, subject has a negative urine or serum pregnancy test.
6. Subject is willing to have an HIV test, participate in HIV counseling, and return to clinic for follow-up.
7. In the opinion of the investigator, subject is able and willing to comply with study procedures, including receipt of three BPG injected doses if randomized to Arm 2.

8. If female, subject must be of non-childbearing potential* or must be using an acceptable method of birth control** to avoid becoming pregnant.

   *Non-childbearing potential is defined as being post-menopausal for at least 1 year, status after bilateral tubal ligation, or status after bilateral oophorectomy, or status after hysterectomy.

   **Subject must agree to avoid becoming pregnant by using one of the following acceptable methods of birth control for the entire duration of participation in the trial:

   - Intrauterine contraceptive device; OR
   - Oral contraceptives; OR
   - Hormonal injections; OR
   - Hormonal implants; OR
   - Contraceptive patches; OR
   - Monogamous relationship with vasectomized partner; OR
   - Exclusively same-sex relationships; OR
   - Use of condoms by the male partner; OR
   - Abstinence

## 5.2 Subject Exclusion Criteria

Subjects eligible to enroll in this study must not meet any exclusion criteria:

1. Subject previously enrolled in this trial.
2. Subject has latent syphilis of unknown duration, late latent syphilis, or evidence of neurosyphilis, including ocular syphilis.*

   *e.g., eye pain/redness, recent ocular change, and/or changes in visual acuity
3. Subject has a known or suspected allergy or hypersensitivity to penicillin or other beta-lactam antibiotics.
4. Subject has a known or suspected STI other than syphilis requiring treatment with a drug active against *T. pallidum*.
5. Subject has used antibiotics* active against *T. pallidum* in the preceding 30 days.

   *Note: the use of antimicrobials known to NOT be effective against *T. pallidum* (e.g., quinolones, sulfonamides, trimethoprim, metronidazole, spectinomycin) will be allowed.
6. Subject has suspected or known ongoing drug use that might interfere with study participation and follow-up treatment.
7. Subject is breastfeeding.
8. Subject has used an investigational drug in the past 30 days that might interfere with safety or efficacy assessment.*

   *If the subject has used any investigational drugs in the past 30 days, contact the Principal Investigator, DMID Clinical Project Manager, DMID Medical Officer, and FHI 360 to confirm eligibility.
9. Subject has any other condition that, in the opinion of the investigator, would interfere with participation in the study.

## 5.3 Treatment Assignment Procedures

### 5.3.1 Randomization Procedures

Enrollment of subjects will be done online using the enrollment module of Emmes Advantage eClinical®. Subjects will be randomized at a 1:1 ratio to receive one of the two BPG treatments after informed consent is obtained and their eligibility is confirmed.

The study will use a stratified, permuted block-randomization scheme. Permuted block randomization is used to avoid the potential for serious imbalance in the number of subjects assigned to each group, an imbalance that can occur in the simple randomization procedures. Stratification will be by study site. While HIV status may not be known at the time of randomization, subjects will be classified as HIV-infected or HIV-uninfected to assess the secondary HIV status subgroup objective, and randomization will be limited as follows:

- If 280 subjects who were HIV-infected at baseline are confirmed evaluable, additional subjects who are known to be HIV-infected will not be allowed to enroll thereafter, while HIV-uninfected subjects and subjects of unknown HIV status may continue to enroll.

- If 140 subjects who were HIV-uninfected at baseline are confirmed evaluable, only subjects who are known to be HIV-infected will be allowed to enroll thereafter.

The list of randomized treatment assignments will be prepared by statisticians at Emmes and included in the enrollment module of its Internet Data Entry System (IDES). IDES will assign each subject a treatment code from the list after demographic and eligibility data have been entered.

Instructions for use of the enrollment module are included in the IDES User's Guide. Manual back-up randomization procedures are provided in the Manual of Procedures (MOP) for use in case a site temporarily loses access to the Internet or the online enrollment system is unavailable.

### 5.3.2 Masking Procedures

Neither subjects nor providers will be masked in this study. The outcome (serological response to therapy) is objective—furthermore, laboratory staff will be blinded to subject ID and treatment assignment—and it would not be ethical to give subjects placebo injections.

### 5.3.3 Reasons for Withdrawal from Study Participation/Discontinuation of Study Treatment

Subjects may voluntarily withdraw their consent for further study participation or may request to discontinue study treatment at any time and for any reason without penalty or prejudice to future

medical care.

In addition to voluntarily requesting to discontinue study treatment as noted above, subjects randomized to Arm 2 may also be discontinued from additional treatment after discussion with the investigator for the following reasons:

- AE(s) judged to be Grade 3 and related to study drug
- SAE(s) judged to be related to study drug
- Investigator's discretion
- Violation of eligibility criteria
- Significant deviation from the treatment plan specified in the protocol (e.g., incorrect administration of BPG, failure to attend study visits)
- Receipt of antibiotic therapy active against *T. pallidum* for treatment of syphilis infection

Subjects in either arm who require off-study retreatment for syphilis infection will be withdrawn from the study after completing an Early Termination Visit.

### 5.3.4 Handling of Withdrawals from Study Participation/Discontinuations of Study Treatment

Subjects who withdraw from study participation, or are excluded from any efficacy endpoint analysis, or are lost to follow-up after signing the informed consent form (ICF), randomization, and receipt of the first dose of BPG will not be replaced. Subjects who withdraw consent after signing the ICF and randomization but before receipt of the first dose of BPG may be replaced.

If the subject requests to discontinue study treatment at any time after the first dose of BPG, the subject will be asked to continue scheduled study procedures including safety and efficacy evaluations, if possible, and be given appropriate care under medical supervision if symptoms of any AE related to participation in the study are continuing. The subject will be followed until the AE is resolved or until the subject's condition becomes stable.

Subjects who request to discontinue study treatment at any time after the first dose of BPG will be reminded of the importance of continuing in the study for safety evaluations. Subjects will be encouraged to complete the Early Termination Visit if they choose not to complete the remaining study visits (i.e., if they withdraw from study participation). The Early Termination Visit procedures are listed in Section 7.3. Subjects who choose to withdraw from study participation will no longer be contacted for follow-up.

In the case of subjects who fail to appear for a follow-up safety assessment, extensive effort (e.g., three documented contact attempts via phone calls, e-mails, etc., made on separate occasions and followed by a certified letter) will be made to locate or recall them, or at least to determine their health status. Subjects who cannot be located after extensive effort will no longer be contacted for follow-up. These efforts will be documented in the subject's records.

If a subject is withdrawn from the study due to an AE that prohibits continued participation in the study, s/he will be given appropriate care and treatment under medical supervision until the condition has resolved or becomes stable.

Safety and efficacy data will be collected on any subject who is excluded from any efficacy endpoint analysis. Refer to Section 11 for details on how subjects who are withdrawn will be handled during analysis.

### 5.3.5 Termination of Study

Although the study sponsor has every intention of completing the study, the sponsor reserves the right to terminate the study at any time for clinical or administrative reasons. Reasons for termination include, but are not limited to, study closure due to DSMB review and recommendation or at the discretion of DMID.

# 6 STUDY INTERVENTION/INVESTIGATIONAL PRODUCT

## 6.1 Study Product Description

Penicillin G benzathine injectable suspension (BPG) is prepared by the reaction of dibenzylethylene diamine with two molecules of penicillin G. It occurs as a white crystalline powder and is very slightly soluble in water and sparingly soluble in alcohol. BPG (Bicillin® L-A) is available as a suspension for deep intramuscular (IM) injection.

### 6.1.1 Acquisition

BPG (Bicillin® L-A) is manufactured by Pfizer Laboratories and will be purchased from the study budget. Upon request by DMID, BPG will be transferred to:

DMID Clinical Materials Services (CMS)
Fisher BioServices
20439 Seneca Meadows Parkway
Germantown, MD 20876
Phone: [redacted]
Fax: [redacted]
Email: [redacted]

BPG will be shipped from the DMID Clinical Materials Services (CMS) to the investigational sites upon request and approval by DMID.

### 6.1.2 Formulation, Packaging, and Labeling

BPG contains penicillin G benzathine in aqueous suspension with sodium citrate buffer and, as weight/volume, approximately 0.5% lecithin, 0.6% carboxymethylcellulose, 0.6% povidone, 0.1% methylparaben, and 0.01% propylparaben. Bicillin® L-A suspension in the disposable-syringe formulation is viscous and opaque, and is available in a 2-mL size containing 1.2 MU BPG per syringe. It is labeled for deep **IM injection only**, not for intravenous (IV) use.

### 6.1.3 Product Storage and Stability

Refer to the protocol-specific MOP for guidance on BPG storage.

## 6.2 Dosage, Preparation, and Administration of Study Intervention/Investigational Product

At each site, a Research Pharmacist or clinician may be delegated the responsibility of BPG dispensation. The Research Pharmacist must be a licensed, registered pharmacist and is the

preferred healthcare practitioner to be delegated to perform this activity. If a Research Pharmacist is not available, a physician, nurse practitioner, physician assistant, registered nurse, or other authorized healthcare practitioner who is a member of the clinical study staff may be delegated to dispense BPG. These personnel must be licensed, trained, and qualified to prepare an investigational study product and must be authorized to dispense it under state and local rules and regulations.

BPG will be administered as a deep IM injection in the upper, outer quadrant of the buttock. Subjects will be randomized to one of two treatment assignments:

- Arm 1: BPG 2.4 MU injected once as two 2-mL injections (one in each buttock)
- Arm 2: BPG 2.4 MU injected weekly as two 2-mL injections (one in each buttock) for three successive weeks

## 6.3 Accountability Procedures for the Study Intervention/Investigational Product(s)

After receiving the BPG, the site principal investigator (PI) is responsible for its distribution and disposition and has ultimate responsibility for its accountability. The site PI may delegate to a site Research Pharmacist or an appropriately qualified staff member the responsibility for BPG accountability. The designee will be responsible for maintaining complete records and documentation of BPG receipt, accountability, dispensation, temperature and storage conditions, and final disposition. All BPG, whether administered or not, must be documented on the appropriate study drug accountability record or dispensing log. The sponsor's monitoring staff will verify the participating clinical sites’ study drug accountability records and dispensing logs per the study monitoring plan.

Upon completion or termination of the study and after the final monitoring visit, final disposition of the unused BPG will be determined by DMID and communicated to the sites by the DMID Clinical Project Manager.

## 6.4 Assessment of Subject Compliance with Study Intervention/Investigational Product

All BPG injections will be directly observed by the administering clinician. Compliance with study product for all subjects will be defined as whether subjects receive all assigned doses within the assigned visit windows.

## 6.5 Concomitant Medications/Treatments

Administration of any medications, therapies, or vaccines will be recorded on the appropriate data collection form. Concomitant medications will include all medications taken 30 days before initiating study treatment through Month 12 or early termination, whichever occurs first. Prescription and over-the-counter drugs will be included, as well as herbs, vitamins, and supplements. Previously recorded medications will be updated as appropriate.

Subjects who have received BPG and are subsequently diagnosed with a concomitant infection that requires systemic antibiotics will receive treatment according to the local clinic's standard protocols. Likewise, subjects who have received BPG through the study and subsequently require retreatment for syphilis infection will receive treatment according to the local clinic's standard protocols and will be withdrawn from the study per protocol Section 5.3.3.

At the discretion of the site PI, use of new medication should prompt evaluation for the presence of a new diagnosis of chronic medical disease or condition.

Medications that might interfere with the evaluation of BPG should not be used unless absolutely necessary.

Refer to Section 5 (Subject Inclusion and Exclusion Criteria) for medications that are prohibited for study eligibility and throughout study participation.

# 7 STUDY SCHEDULE

Study visit information is listed in this section, Section 8, and the Schedule of Events (Appendix A). Further instructions are described in the protocol-specific MOP.

## 7.1 Visit 1 – Screening and Enrollment/Baseline Visit (Day 1)

- The potential subject will be provided with a description of the study (purpose and study procedures) and asked to read the ICF or have it read to him/her. The ICF must be signed before any screening or study procedures are performed.
- Demographic information will be collected from the subject.
- Eligibility criteria will be reviewed with the subject.
  - Complete medical history will be obtained by interviewing subjects (and reviewing medical records for the past 14 days, if available) to assure eligibility. If available in the medical record, the most recent viral load result from the past six months prior to enrollment should be recorded.
  - Sexual history for the past 60 days will be collected.
  - A targeted physical examination will be performed by a qualified study clinician (including vital signs: temperature, heart rate, respiration rate, and blood pressure; and genital, rectal, oral, skin, and lymph node examinations).
  - Study staff will swab consenting subjects' oral cavities and primary or secondary lesions (if lesions are present per Section 5.1); see Section 8.2.2.
  - A urine or serum pregnancy test will be performed on all subjects of childbearing potential and must be negative prior to randomization.
  - All concomitant medications taken in the last 30 days prior to initiating BPG treatment will be recorded on the appropriate data collection form.
  - Blood will be collected for RPR test, storage, and HIV assays (including a CD4 count for persons with known HIV infection whose medical records do not include a CD4 count in the past 30 days, and an HIV test for persons who do not have a previously documented positive HIV test result). Specimens will be collected at sites of exposure for screening/diagnosis of gonorrhea and chlamydia for all subjects who have not been tested since last sexual activity and for all subjects who have been sexually active in the past 14 days and for other STIs as indicated by local standard of care and subject history. Clinicians will provide pre- and post-test counseling, treatment, and referrals per local standard of care.

At this juncture, if a potential subject is not eligible, the reason for screening failure will be recorded and the subject will not be enrolled.

- Subject will be enrolled in Advantage eClinical® and randomly assigned to Arm 1 or Arm 2.

- BPG will be administered, and the subject will be observed per clinic standard operating procedure (SOP).

- Study personnel will discuss with subjects and assess and record all AEs/SAEs (including solicited reactogenicity AEs and other unsolicited AEs).

- Protocol requirements will be reviewed with the subject.

- Contact information will be collected, the subject's preferred method of contact will be noted, and the Week 1 visit will be scheduled.

- The Jarisch-Herxheimer checklist will be distributed and the instructions reviewed with the subject. The subject will be asked to have the completed checklist available for reference during the non-visit contact approximately 24-48 hours after Visit 1 and to bring it to Visit 2. Study personnel will instruct the subject to use a thermometer to take temperature reading(s) at home, if possible, and note the highest temperature if s/he feels feverish any time before being contacted to assess for symptoms of a Jarisch-Herxheimer reaction as described in Section 8.1. Study personnel will attempt to contact subjects by phone call, text message, or email approximately 24 hours after Visit 1 to make this assessment. Note that data will be recorded on the appropriate electronic case report forms (eCRFs) based on subject interview; the Jarisch-Herxheimer checklist completed by the subject will not be retained.

## 7.2 Follow-up

### 7.2.1 Visit 2 – Week 1 (Day 7 to 13)

- Subject will be evaluated for resolution of any clinical signs of infection and will be asked specific questions on medication tolerance. If the subject was not contacted following Visit 1, s/he will be evaluated for symptoms of a Jarisch-Herxheimer reaction at Visit 2 as described in Section 8.1.

- Medical history will be reviewed (including review of interval medical records and local laboratory results, if available) and updated as appropriate. If available in the medical record, the most recent viral load result since enrollment should be recorded. If the local laboratory results indicate a need for retreatment for syphilis infection, the subject will receive treatment according to the local clinic's standard protocols and will be withdrawn from the study per protocol Section 5.3.3.

- Interim sexual history since the last study visit will be collected. Specimens will be collected at sites of exposure for STI screening/diagnosis as indicated by local standard of care and subject history.

- A urine pregnancy test will be performed on all subjects of childbearing potential.
- All concomitant medications taken since the last study visit will be recorded on the appropriate data collection form. Previously recorded medications will be updated as appropriate.
- A targeted physical examination will be performed by a qualified study clinician (including vital signs: temperature, heart rate, respiration rate, and blood pressure; and genital, rectal, oral, skin, and lymph node examinations).
- Blood will be collected for follow-up RPR tests and storage.
- Arm 2 only: second dose of BPG will be administered, and the subject will be observed per clinic SOP.
- Study personnel will discuss with subjects and assess and record all AEs/SAEs (including solicited reactogenicity AEs and other unsolicited AEs).
- Contact information will be reviewed, and the Week 2 visit will be scheduled.

### 7.2.2 Visit 3 – Week 2 (6 to 12 days after Visit 2)

- Subject will be evaluated for resolution of any clinical signs of infection.
- Medical history will be reviewed (including review of interval medical records and local laboratory results, if available) and updated as appropriate. If available in the medical record, the most recent viral load result since enrollment should be recorded. If the local laboratory results indicate a need for retreatment for syphilis infection, the subject will receive treatment according to the local clinic's standard protocols and will be withdrawn from the study per protocol Section 5.3.3.
- Interim sexual history since the last study visit will be collected. Specimens will be collected at sites of exposure for STI screening/diagnosis as indicated by local standard of care and subject history.
- A urine pregnancy test will be performed on all subjects of childbearing potential.
- All concomitant medications taken since the last study visit will be recorded on the appropriate data collection form. Previously recorded medications will be updated as appropriate.
- A targeted physical examination will be performed by a qualified study clinician (including vital signs: temperature, heart rate, respiration rate, and blood pressure; and genital, rectal, oral, skin, and lymph node examinations).
- Blood will be collected for follow-up RPR tests and storage.

- Arm 2 only: third dose of BPG will be administered, and the subject will be observed per clinic SOP.
- Study personnel will discuss with subjects and assess and record all AEs/SAEs (including solicited reactogenicity AEs and other unsolicited AEs).
- Contact information will be reviewed, and the Month 1 visit will be scheduled.

### 7.2.3 Visit 4 – Month 1 (Day 30 ± 7 days)

- Medical history will be reviewed (including review of interval medical records and local laboratory results, if available) and updated as appropriate. If available in the medical record, the most recent viral load result since enrollment should be recorded. If the local laboratory results indicate a need for retreatment for syphilis infection, the subject will receive treatment according to the local clinic's standard protocols and will be withdrawn from the study per protocol Section 5.3.3.
- Interim sexual history since the last study visit will be collected. Specimens will be collected at sites of exposure for STI screening/diagnosis as indicated by local standard of care and subject history.
- A urine pregnancy test will be performed on all subjects of childbearing potential.
- All concomitant medications taken since the last study visit will be recorded on the appropriate data collection form. Previously recorded medications will be updated as appropriate.
- Study personnel will discuss with subjects and assess and record all AEs/SAEs (including solicited reactogenicity AEs and other unsolicited AEs).
- A targeted physical examination will be performed by a qualified study clinician (including vital signs: temperature, heart rate, respiration rate, and blood pressure; and genital, rectal, oral, skin, and lymph node examinations).
- Blood will be collected for follow-up RPR tests, storage, and CD4 counts on persons newly found to have HIV infection from Visit 1 to Visit 4 (inclusive) whose medical records do not include a CD4 count in the past 30 days. Clinicians will provide pre- and post-test counseling, treatment, and referrals per local standard of care.
- Contact information will be reviewed, and the Month 3 visit will be scheduled.

### 7.2.4 Visit 5 – Month 3 (Day 90 ± 21 days)

- Medical history will be reviewed (including review of interval medical records and local laboratory results, if available) and updated as appropriate. If the local laboratory results indicate a need for retreatment for syphilis infection, the subject will receive treatment

according to the local clinic’s standard protocols and will be withdrawn from the study per protocol Section 5.3.3.

- Interim sexual history since the last study visit will be collected. Specimens will be collected at sites of exposure for STI screening/diagnosis as indicated by local standard of care and subject history.
- A urine pregnancy test will be performed on all subjects of childbearing potential.
- All concomitant medications taken since the last study visit will be recorded on the appropriate data collection form. Previously recorded medications will be updated as appropriate.
- A targeted physical examination will be performed by a qualified study clinician (including vital signs: temperature, heart rate, respiration rate, and blood pressure; and genital, rectal, oral, skin, and lymph node examinations).
- HIV testing will be performed if the subject was previously noted to be HIV-uninfected. Clinicians will provide pre- and post-test counseling, treatment, and referrals per local standard of care.
- Blood will be collected for follow-up RPR tests and storage.
- Contact information will be reviewed, and the Month 6 visit will be scheduled.

### 7.2.5 Visit 6 – Month 6 (Day 180 ± 21 days)

- Medical history will be reviewed (including review of interval medical records and local laboratory results, if available) and updated as appropriate. If the local laboratory results indicate a need for retreatment for syphilis infection, the subject will receive treatment according to the local clinic’s standard protocols and will be withdrawn from the study per protocol Section 5.3.3.
- Interim sexual history since the last study visit will be collected. Specimens will be collected at sites of exposure for STI screening/diagnosis as indicated by local standard of care and subject history.
- A urine pregnancy test will be performed on all subjects of childbearing potential.
- All concomitant medications taken since the last study visit will be recorded on the appropriate data collection form. Previously recorded medications will be updated as appropriate.
- A targeted physical examination will be performed by a qualified study clinician (including vital signs: temperature, heart rate, respiration rate, and blood pressure; and genital, rectal, oral, skin, and lymph node examinations).
- HIV testing will be performed if the subject was previously noted to be HIV-uninfected.

Clinicians will provide pre- and post-test counseling, treatment, and referrals per local standard of care.

- Blood will be collected for follow-up RPR tests, storage, and CD4 counts on persons found to have HIV infection whose medical records do not include a CD4 count in the past 30 days.
- Contact information will be reviewed, and the Month 9 visit will be scheduled.

### 7.2.6 Visit 7 – Month 9 (Day 270 ± 28 days)

- Medical history will be reviewed (including review of interval medical records and local laboratory results, if available) and updated as appropriate. If the local laboratory results indicate a need for retreatment for syphilis infection, the subject will receive treatment according to the local clinic's standard protocols and will be withdrawn from the study per protocol Section 5.3.3.
- Interim sexual history since the last study visit will be collected. Specimens will be collected at sites of exposure for STI screening/diagnosis as indicated by local standard of care and subject history.
- A urine pregnancy test will be performed on all subjects of childbearing potential.
- All concomitant medications taken since the last study visit will be recorded on the appropriate data collection form. Previously recorded medications will be updated as appropriate.
- A targeted physical examination will be performed by a qualified study clinician (including vital signs: temperature, heart rate, respiration rate, and blood pressure; and genital, rectal, oral, skin, and lymph node examinations).
- HIV testing will be performed if the subject was previously noted to be HIV-uninfected. Clinicians will provide pre- and post-test counseling, treatment, and referrals per local standard of care.
- Blood will be collected for follow-up RPR tests and storage.
- Contact information will be reviewed, and the Month 12 visit will be scheduled.

### 7.2.7 Visit 8 – Month 12 (Day 360 ± 28 days) (Final Study Visit)

- Medical history will be reviewed (including review of interval medical records and local laboratory results, if available) and updated as appropriate. If the local laboratory results indicate a need for retreatment for syphilis infection, the subject will receive treatment according to the local clinic's standard protocols.
- Interim sexual history since the last study visit will be collected. Specimens will be

collected at sites of exposure for STI screening/diagnosis as indicated by local standard of care and subject history.

- A urine pregnancy test will be performed on all subjects of childbearing potential.
- All concomitant medications taken since the last study visit will be recorded on the appropriate data collection form. Previously recorded medications will be updated as appropriate.
- A targeted physical examination will be performed by a qualified study clinician (including vital signs: temperature, heart rate, respiration rate, and blood pressure; and genital, rectal, oral, skin, and lymph node examinations).
- HIV testing will be performed if the subject was previously noted to be HIV-uninfected. Clinicians will provide pre- and post-test counseling, treatment, and referrals per local standard of care.
- Blood will be collected for follow-up RPR tests, storage, and CD4 counts on persons found to have HIV infection whose medical records do not include a CD4 count in the past 30 days.
- Contact information will be reviewed.

## 7.3 Early Termination Visit

The Visit 8 assessments and eCRFs (see Section 7.2.7: Visit 8 – Month 12 (Final Study Visit)) must be completed at the end of each subject's participation in the study if discontinued prior to Visit 8. If a subject withdraws between scheduled visits, the subject must come to the clinic to perform the Visit 8 assessments.

## 7.4 Unscheduled Visit

Subjects will be permitted to return for unscheduled visits between scheduled visits as needed to address issues related to AEs, continuing or new symptoms, or other problems as they arise. Any of the following activities may be performed at the discretion of the site PI (note that some items are only relevant to a subset of subjects):

- Medical history will be reviewed (including review of interval medical records and local laboratory results, if available) and updated as appropriate. If the local laboratory results indicate a need for retreatment for syphilis infection, the subject will receive treatment according to the local clinic's standard protocols and will be withdrawn from the study per protocol Section 5.3.3.
- Interim sexual history since the last study visit will be collected. Specimens will be collected at sites of exposure for STI screening/diagnosis as indicated by local standard of care and subject history.

- A urine pregnancy test will be performed on all subjects of childbearing potential.
- All concomitant medications taken since the last study visit will be recorded on the appropriate data collection form. Previously recorded medications will be updated as appropriate.
- Study personnel will discuss with subjects and assess and record all AEs/SAEs. Previously recorded AEs/SAEs will be updated as appropriate.
- A targeted physical examination will be performed by a qualified study clinician (including vital signs: temperature, heart rate, respiration rate, and blood pressure; and genital, rectal, oral, skin, and lymph node examinations).
- Contact information will be reviewed.

# 8 STUDY PROCEDURES/EVALUATIONS

## 8.1 Clinical Evaluations

Complete medical history will be obtained by interviewing the subjects at Visit 1 (and reviewing medical records for the past 14 days, if available) and will be updated at each clinic visit. At follow-up visits, an interim medical history will be obtained by interviewing the subjects (and reviewing interval medical records, if available), noting any changes since the previous visit.

Sexual history for the past 60 days will be obtained by interviewing the subjects at Visit 1. At follow-up visits, an interim sexual history will be obtained by interviewing the subjects, noting any changes since the previous visit.

Medications history (concomitant medications) will include a review of all current medications and medications taken 30 days before initiating study treatment. Prescription and over-the-counter drugs will be included as well as vitamins, herbs, and supplements. Assessment of eligibility will also include a review of all permitted and prohibited medications per the Subject Inclusion and Exclusion Criteria (see Section 5).

A targeted physical examination (vital signs (temperature, heart rate, respiration rate, and blood pressure); genital, rectal, oral, skin, and lymph node examinations) will be performed at each visit. All physical examinations will be performed by a qualified study clinician.

**Evaluation for symptoms of a Jarisch-Herxheimer reaction**

Approximately 24 hours after Visit 1, the subjects will be contacted as noted in Section 7.1 and evaluated for symptoms of a Jarisch-Herxheimer reaction. During this assessment, subjects should refer to the completed Jarisch-Herxheimer checklist distributed at Visit 1. Subjects will be asked about fever (confirmed by thermometer at home close to the time of fever onset, if possible), chills, myalgia, weakness, flushing, worsening of skin rash, tachycardia (i.e., fast heartbeat), heart palpitations, arthralgia, nausea, headache, and dizziness, including time of onset and time of resolution of each symptom reported. If subjects cannot be reached after Visit 1, the evaluation for symptoms of a Jarisch-Herxheimer reaction will be conducted at Visit 2 (per Section 7.1, all subjects will be asked to bring the completed checklist with them to Visit 2). Data will be recorded on the appropriate eCRF(s) based on subject interview during the non-visit contact or at Visit 2 if the contact is not successful; the Jarisch-Herxheimer checklist completed by the subject will not be retained.

**Evaluation for resolution of clinical signs of syphilis infection**

At Visit 2 and Visit 3, subjects will be evaluated for resolution of signs of syphilis documented at Visit 1, as noted in Section 7.

## 8.2 Laboratory Evaluations

### 8.2.1 Clinical Laboratory Evaluations

Diagnostic laboratory tests for HIV and other STIs as indicated will be performed locally according to local protocols. Phlebotomy for serological testing and serum storage to determine study outcomes will be performed locally with testing carried out at the Central Laboratory. Serological testing may also be conducted locally according to local protocols.

A pregnancy test will be performed at all visits on all subjects of childbearing potential; a urine or serum pregnancy test is permitted at Visit 1, while a urine pregnancy test is to be performed at all other visits. Results must be negative and known prior to randomization at Visit 1.

### 8.2.2 Special Assays or Procedures

At Visit 1, study staff will swab consenting subjects' oral cavities and primary or secondary lesions (if lesions are present per Section 5.1). Swabs will be stored in study-specific medium and shipped to the Giacani laboratory at the University of Washington for whole-genome sequencing of *T. pallidum* isolates. Collecting oral swabs and swabs of lesions (if present) is optional, and additional details can be found in the protocol-specific swab guidance manual.

### 8.2.3 Specimen Preparation, Handling, and Shipping

#### 8.2.3.1 Instructions for Specimen Preparation, Handling, and Storage

Specimen preparation, handling, and storage will be done according to local clinic SOPs. Additional details can be found in the protocol-specific MOP.

#### 8.2.3.2 Specimen Shipment

Specimen shipment to the Central Laboratory will occur according to all applicable International Air Transport Association (IATA) requirements. Additional details can be found in the protocol-specific MOP.

# 9 ASSESSMENT OF SAFETY

## 9.1 Specification of Safety Parameters

Safety will be monitored throughout the study by physical examination (including vital signs: temperature, heart rate, respiration rate, and blood pressure; and genital, rectal, oral, skin, and lymph node examinations) and subject reporting. Safety will be assessed by the frequency and severity of AEs/SAEs (including solicited reactogenicity AEs and other unsolicited AEs) occurring from the time of study product administration through the Month 1 visit.

## 9.2 Methods and Timing for Assessing, Recording, and Analyzing Safety Parameters

### 9.2.1 Adverse Events

**Adverse Event:**

ICH E6 GCP defines an AE as any untoward medical occurrence in a clinical research subject administered a study drug regardless of its causal relationship to the study drug. An AE can therefore be any unfavorable and unintended sign (including an abnormal laboratory finding), symptom, or disease temporally associated with the use of the study drug. The occurrence of an AE may come to the attention of study personnel during study visits and interviews of a study subject presenting for medical care or upon review by a study monitor. Since the safety profile of BPG (both 1 and 3 doses) is well established, and this study is not powered to detect new, unknown safety signals, non-serious AEs will not be reported to the DSMB.

All AEs not meeting the criteria for SAEs should be captured on the appropriate eCRF. Information to be collected includes event description, time of onset, clinician's assessment of severity, relationship to study drug (assessed only by those with the training and authority to make a diagnosis), and time of resolution/stabilization of the event. All AEs occurring while on study must be documented appropriately regardless of relationship. All AEs will be followed to adequate resolution.

Any medical condition that is present at the time that the subject is screened should be considered as a baseline finding and not reported as an AE. However, if it deteriorates at any time during the study, it should be recorded as an AE.

All AEs must be graded for severity and relationship to the study drug.

The US Food and Drug Administration (FDA) defines an AE as any untoward medical occurrence associated with the use of a drug in humans, whether or not considered drug-related.

**Severity of Event:** All AEs will be assessed by the clinician using a protocol-defined grading system (see Appendix B).

Changes in the severity of an AE should be documented to allow an assessment of its duration at each level of intensity to be performed. AEs characterized as intermittent require documentation of onset and duration of each episode.

**Relationship to Study Drug:** The clinician's assessment of an AE's relationship to the study drug is part of the documentation process, but it is not a factor in determining what is or is not reported in the study. If there is any doubt as to whether a clinical observation is an AE, it should be reported. All AEs must have their relationship to the study drug assessed using the terms "related" or "not related," as defined below. In a clinical trial, the study drug must always be suspect.

- Related – There is a reasonable possibility that the study drug caused the AE. Reasonable possibility means that there is evidence to suggest a causal relationship between the study drug and the AE.

- Not Related – There is not a reasonable possibility that administration of the study drug caused the AE.

### 9.2.2 Reactogenicity

Reactogenicity events are AEs that are common and known to occur for the study drug being studied. For this protocol, the following reactogenicity events are expected to occur among some subjects:

- Systemic reactions: Jarisch-Herxheimer reaction (as described in protocol Section 8.1)

- Local injection site reactions: pain or tenderness, erythema or redness, induration or swelling

Reactogenicity events (as solicited AEs), along with unsolicited AEs, will be assessed at the visits specified in protocol Section 7 and will be graded using a protocol-defined grading system (see Appendix B).

### 9.2.3 Serious Adverse Events

**Serious Adverse Event (SAE)**:

An AE or suspected adverse reaction is considered "serious" if, in the view of either the investigator or sponsor, it results in any of the following outcomes:

- death,

- a life-threatening AE*,
- inpatient hospitalization or prolongation of existing hospitalization,
- a persistent or significant incapacity or substantial disruption of the ability to conduct normal life functions, or
- a congenital anomaly/birth defect.
- Important medical events that may not result in death, be life-threatening, or require hospitalizations may be considered serious when, based upon appropriate medical judgment, they may jeopardize the subject and may require medical or surgical intervention to prevent one of the outcomes listed in this definition. Examples of such medical events include allergic bronchospasm requiring intensive treatment in an emergency room or at home, blood dyscrasias or convulsions that do not result in inpatient hospitalization, or the development of drug dependency or drug abuse.

* An AE is considered “life-threatening” if, in the view of either the investigator or sponsor, its occurrence places the subject at immediate risk of death. It does not include an AE that, had it occurred in a more severe form, might have caused death.

All SAEs will be:

- Assessed for severity and relationship to the study drug and alternate etiology (if not related to the study drug) by a licensed study physician.
- Recorded on the appropriate SAE form and eCRF.
- Followed through resolution by a licensed study physician.
- Reviewed and evaluated by the DSMB (periodic review unless related), DMID, and the IRB.

## 9.3 Reporting Procedures

### 9.3.1 Serious Adverse Events

SAEs will be followed until resolution even if this extends beyond the study reporting period. Resolution of an SAE is defined as the return to pretreatment status or stabilization of the condition with the expectation that it will remain chronic.

**Any AE that meets a protocol-defined serious criterion must be submitted immediately (within 24 hours of site awareness) on an SAE form to the DMID Pharmacovigilance Group, at the following address:**

**DMID Pharmacovigilance Group**
**Clinical Research Operations and Management Support (CROMS)**
**6500 Rock Spring Dr. Suite 650**
**Bethesda, MD 20817, USA**
**SAE Hot Line:** [redacted]
**SAE FAX Phone Number:** [redacted]
**SAE Email Address:** [redacted]

Other supporting documentation of the event may be requested by the DMID Pharmacovigilance Group and should be provided as soon as possible.

The DMID Medical Monitor and Clinical Protocol Manager will be notified of the SAE by the DMID Pharmacovigilance Group. The DMID Medical Monitor will review and assess the SAE for regulatory reporting and potential impact on study subject safety and protocol conduct.

At any time after completion of the study, if the investigator becomes aware of an SAE that is suspected to be related to the study drug, the investigator will report the event to the DMID Pharmacovigilance Group.

### 9.3.2 Regulatory Reporting for Studies Not Conducted Under DMID-Sponsored IND

As this study will not be conducted under an Investigational New Drug application (IND), MedWatch will be used only to report SAEs that are both unexpected and deemed related to the study drug. DMID should be copied simultaneously when an alternate method of reporting is utilized.

### 9.3.3 Reporting of Pregnancy

Subjects of child-bearing potential will be counseled to continue using acceptable forms of birth control for the entire duration of their participation in the trial. If a subject becomes pregnant during the study, dosing will be discontinued immediately, and early termination assessments will be performed. A pregnancy reporting form will be completed for any study subject who becomes pregnant during study participation. The site will maintain contact with pregnant study subjects to obtain pregnancy outcome information. The pregnant subject will be followed until delivery or until the end of pregnancy (in the case of miscarriage or pregnancy termination). Infants born to these study subjects will also be monitored for SAEs (congenital anomalies or other birth defects) and other complications for up to 2 months after birth. Pregnancy reporting forms will be limited to collecting data on the following information:

- prior maternal history including congenital abnormalities or pregnancy complications;

- estimated date of conception;
- estimated and actual date of delivery or end of pregnancy;
- pregnancy outcome (live birth, stillbirth, miscarriage, or elective termination);
- mode of delivery;
- maternal complications; and
- neonatal complications (i.e., lethal or nonlethal congenital abnormality).

Pregnancies occurring in subjects will be reported via Advantage eClinical® on the Pregnancy Report form.

## 9.4 Type and Duration of Follow-up of Subjects after Adverse Events

All AEs and SAEs will be followed from the time of study treatment through resolution even if this extends beyond the study reporting period (i.e., the Month 1 visit). Resolution of an AE/SAE is defined as the return to pretreatment status or stabilization of the condition with the expectation that it will remain chronic.

Follow-up procedures, evaluations, and outcomes will be recorded on the appropriate data collection form.

## 9.5 Halting Rules

Further enrollment will be halted for DSMB review if any of the following are reported:

- If one or more subjects experience an SAE judged by an investigator to be related to the study drug
- An overall pattern of clinical events that the DMID Medical Monitor or DSMB consider associated with the study drug and that may appear minor in terms of individual events, but that may collectively represent a serious potential concern for safety

If any of the halting rules are met, the study will not continue with the remaining enrollments or study treatments without a review by and recommendation from the DSMB to proceed.

DMID retains the authority to suspend additional enrollment and administration of the study drug during the entire study, as applicable.

## 9.6 Safety Oversight (ISM plus DSMB)

An ISM at each clinical research site will oversee the safety of research subjects at that site and will provide independent written evaluation of SAEs and related Grade 3 AEs to the PI, DMID Clinical Project Manager, and DMID Medical Monitor. The ISM will serve as an independent consultant for the site PI on subject-related issues. The ISM will communicate with the site PI and study PIs to resolve any issues.

Safety oversight will be conducted by a DSMB that is an independent group of experts that monitors subject safety and advises DMID. The DSMB members will be separate and independent of study personnel participating in this trial and should not have scientific, financial, or other conflict of interest related to the study. The DSMB will consist of members with appropriate expertise to contribute to the interpretation of the data from this trial.

The DSMB will review safety and enrollment data when half of the study subjects have been enrolled and observed for SAEs through the Month 1 Visit.

The DSMB will review study progress and subject clinical and safety data at the following time points:

- At least annually after the first subject is enrolled.
- Ad hoc when a halting rule is met, for immediate concerns regarding observations during the study, or as needed.
- During a final closeout meeting, held at the end of the study after the database is locked to review cumulative study data prior to finalizing the Clinical Study Report (CSR).

The DSMB will operate under the rules of a DMID-approved charter that will be written at the organizational meeting of the DSMB. As an outcome of each review/meeting, the DSMB will make a recommendation as to the advisability of proceeding with study administrations (as applicable), and to continue, modify, or terminate the study.

DMID or the DSMB chair may convene the DSMB on an ad hoc basis according to protocol criteria or if there are immediate concerns regarding observations during the study. The DMID Medical Monitor is empowered to stop enrollment and study treatment if SAEs that meet the halting criteria are reported. The DMID Medical Monitor will be responsible for reviewing SAEs in real time. The DSMB will review SAEs on a regular basis and *ad hoc* during the study.

# 10 CLINICAL MONITORING

## 10.1 Site Monitoring Plan

DMID, the sponsoring agency, or its designee will conduct site monitoring visits as detailed in the clinical monitoring plan and in accordance with DMID policies. Site monitoring is conducted to ensure that the human subject protections, study and laboratory procedures, study intervention administration, and data collection processes are of high quality and meet sponsor and ICH/GCP guidelines and applicable regulations and that the study is conducted in accordance with the protocol, protocol-specific manual of procedures, and applicable sponsor SOPs. Monitoring visits will include, but are not limited to, inspection of study facilities; review of regulatory files, accountability records, eCRFs, ICFs, printouts of medical and laboratory reports from the electronic medical records (EMR) system; and review of protocol and GCP compliance. Site monitors will have access to the study site, study personnel, and all study documentation according to the DMID-approved site monitoring plan. Study monitors will meet with PIs to discuss any problems and actions to be taken and document visit findings and discussions.

Site visits will be made at standard intervals as defined by DMID and may be made more frequently as directed by DMID.

# 11 STATISTICAL CONSIDERATIONS

## 11.1 Study Hypotheses

The primary objective of the study is to compare the serological response to therapy in subjects with early (primary, secondary, or early latent) syphilis treated with BPG 2.4 MU once or weekly for three successive weeks. In particular, the objective is to demonstrate noninferiority of the one-dose regimen compared to the three-dose regimen and to also assess noninferiority within the HIV status subgroups (HIV-infected and HIV-uninfected subjects). See Section 11.4.4 for the definition of serological response.

The null hypothesis for the primary objective is that the difference in serological response rate between the three-dose and one-dose groups is at least 10%, and the alternative hypothesis is that the difference in response rates is less than 10%. The null and alternative hypotheses for the secondary HIV status subgroup objective are defined similarly.

Note that tests will be performed and p-values will be reported for secondary analyses for informational purposes and not to make definitive claims. The study is intended and designed to formally test the primary hypothesis only, and so no corrections for multiplicity are planned.

## 11.2 Sample Size Considerations

Sample size calculations were performed using PASS 2008 and SAS 9.4. The following parameters were used for determining the required sample size for the primary objective:

- 1:1 allocation ratio between one-dose and three-dose arms
- Test the difference in response rate between the one-dose and three-dose arms (Farrington-Manning test, unadjusted for HIV status)
- Null hypothesis response rate in one-dose arm: 0.688
- Null hypothesis response rate in three-dose arm: 0.788
- Alternative hypothesis response rate in both arms: 0.788
- One-sided 0.05 alpha level

To achieve 80% power for the primary noninferiority comparison, 420 evaluable subjects (210 in each treatment arm) are needed. It is assumed that 25% of subjects will be ineligible for the primary analysis, and so 560 enrolled subjects are needed to reach the target of 420 evaluable subjects.

The study is not powered to assess the secondary noninferiority comparisons in the HIV status subgroups. As an illustration, power calculations were performed to assess the power available for these subgroup analyses using a sample size of 420. The following parameters were used for the calculations:

- Test the difference in response rate between the one-dose and three-dose arms within each HIV status stratum.
- Null hypothesis response rate in one-dose arm: 0.738 (HIV-infected); 0.638 (HIV-uninfected)
- Null hypothesis response rate in three-dose arm: 0.838 (HIV-infected); 0.738 (HIV-uninfected)
- Alternative hypothesis response rate in both arms: 0.838 (HIV-infected); 0.738 (HIV-uninfected)
- One-sided 0.05 alpha level
- 280 evaluable subjects in the HIV-infected stratum (140 in one-dose arm, 140 in three-dose arm).
- 140 evaluable subjects in the HIV-uninfected stratum (70 in one-dose arm, 70 in three-dose arm).

The HIV-infected noninferiority comparison has 72% power, and the HIV-uninfected noninferiority comparison has 38% power.

## 11.3 Planned Interim Analyses

### 11.3.1 Safety Review

The study will be monitored to determine if any of the safety halting rules described in Section 9.5 are met. The DSMB will also review study progress and subject safety data at specified times during the study and hold a study closeout meeting, as defined in the DSMB Charter.

### 11.3.2 Immunogenicity or Efficacy Review

There are no planned interim analyses of efficacy data.

## 11.4 Final Analysis Plan

A separate statistical analysis plan document will be generated that will contain the details of the analyses. This section outlines the major components of the analyses.

### 11.4.1 Analysis Populations

Intent-to-Treat (ITT) Population: This analysis population includes all randomized subjects.

Modified Intent-to-Treat (mITT) Population: This analysis population includes all randomized subjects who are eligible at the baseline visit and whose TPPA result is positive at the baseline visit and/or at Visit 5 (Month 3), if repeat testing was performed.

Safety Population: This analysis population includes all randomized subjects who received at least one dose of study treatment.

Evaluable Population: This analysis population includes all randomized subjects who are eligible at the baseline visit, have a known HIV status determined at or before the baseline visit, have positive TPPA results at the baseline visit and/or Visit 5 (Month 3) (if repeat testing was performed), received all assigned doses of BPG, have adequate RPR titer data available at baseline and applicable follow-up visit(s), and (for HIV-uninfected subjects only) HIV-uninfected status persists through applicable follow-up visit(s).

If a subject receives antibiotic(s) active against *T. pallidum* for reasons other than syphilis between enrollment and the applicable follow-up visit(s), the subject will be included in ITT analyses but excluded from Evaluable analyses. If a subject receives antibiotic(s) active against *T. pallidum* between enrollment and the applicable follow-up visit(s) due to local RPR testing results, the subject will be included in all analyses.

A blinded case review committee will review subjects' data to determine if treatment with drug(s) known to be active against *T. pallidum* for reasons other than syphilis infection excludes the subject from the Evaluable analysis population.

### 11.4.2 Baseline Characteristics

Baseline and demographic characteristics will be summarized overall and by formulation. For both continuous and categorical variables, appropriate summary statistics will be applied. For continuous variables, descriptive statistics will include the number of non-missing values, mean, standard deviation, median, minimum, and maximum. For categorical variables, descriptive statistics will include counts and percentages per category.

### 11.4.3 Safety Analysis Plan

Safety evaluations will be based on the incidence, severity, and type of AEs and SAEs, as detailed in Section 9. In addition, the incidence of systemic reactions and local injection site reactions will be evaluated. Safety variables will be tabulated and presented for all subjects in the safety population, grouped by treatment.

Reactogenicity events will be analyzed by taking the most severe response over the follow-up period, dichotomizing into a binary variable (none versus mild, moderate, or severe) and using exact confidence intervals to summarize the reactogenicity rates. Tabular and graphical summaries of events will be presented for each solicited event, by type (local/systemic), severity (none, mild, moderate, severe), and time point post-dose. The proportion of subjects who experience a Jarisch-Herxheimer reaction will be summarized overall and by treatment. Logistic regression analyses may be used to determine the association of treatment with Jarisch-Herxheimer reaction after adjusting for HIV co-infection status and time point.

Unsolicited AEs and SAEs will be coded by MedDRA® for preferred term and system organ class. The rate and exact 95% confidence intervals of AEs and SAEs in aggregate, as well as by MedDRA® categories, will be computed. The number of SAEs will be reported by a detailed listing showing the type, MedDRA® coding, relevant dates (treatment dosing date(s) and SAE onset and resolution dates), severity, relatedness, and outcome for each event.

### 11.4.4 Efficacy Analysis Plan

#### 11.4.4.1 Primary Efficacy Analysis

For the primary analysis, serological response to therapy by Month 6 will be defined as follows:

- 4-fold or greater decline in RPR titer by Month 6 compared to baseline, OR
- RPR-negative by Month 6 (i.e., seroreversion).

For the primary analysis, subjects who do not meet either of the above criteria by Month 6 will be classified as not experiencing serological response. If a subject has an observed serological response prior to meeting one of the Evaluable analysis population exclusion criteria, the subject will be included in the analyses as a responder. For subjects who have received BPG through the study and subsequently require retreatment for syphilis infection (i.e., per the local clinic's standard protocols), data collected following retreatment will not be used for analyses. Exploratory analyses of the primary outcome will consider alternative classifications of subjects (see Section 11.4.4.2).

For the comparison of efficacy of one dose versus three doses of BPG 2.4 MU, the number and proportion of subjects with serological response will be summarized overall and by treatment.

The point estimates for the treatment-specific proportions and difference in proportions as well as corresponding 95% confidence intervals will be reported. A hypothesis test will be conducted using the Farrington-Manning test at the 5% one-sided level of significance to formally compare the treatment arms. The primary analysis will be performed in the ITT analysis population and repeated as a secondary analysis in the mITT and the Evaluable analysis populations.

As the ITT analyses will include subjects who may not have adequate serological data to determine their response status for any reason, the response will need to be imputed. Multiple methods for imputation will be explored (e.g., Last Observation Carried Forward); details will be provided in the Statistical Analysis Plan.

As a secondary corroborative analysis, the Cochran-Mantel-Haenszel Chi-square test will be used to compare the two treatment arms with respect to response rate across HIV status subgroups (HIV-infected, HIV-uninfected). The Breslow-Day test for the homogeneity of odds ratios will be used to determine if the difference between the two treatment groups with respect to response rates varies by HIV status.

#### 11.4.4.2 Secondary Efficacy Analysis

The secondary efficacy analyses will be performed in the ITT and Evaluable analysis populations. For subjects who have received BPG through the study and subsequently require retreatment for syphilis infection (i.e., per the local clinic's standard protocols), data collected following retreatment will not be used for analyses.

For the comparison of efficacy of one dose versus three doses of BPG 2.4 MU by Month 6 in each of the HIV status subgroups, the number and proportion of subjects with serological response will be summarized overall and by treatment within each HIV status subgroup. The point estimates for the treatment-specific proportions and difference in proportions as well as corresponding 95% confidence intervals will be reported. For each HIV status subgroup, a hypothesis test will be conducted using the Farrington-Manning test at the 5% one-sided level of significance to formally compare the treatment arms.

The analyses comparing one dose versus three doses by Month 12, overall and within HIV status subgroups, will follow similarly to the above. In addition, geometric mean titers and fold declines from baseline will be summarized by time-point for each treatment group, overall and within HIV status subgroups.

For the comparison of compliance between the one dose and three dose groups, the number and proportion of subjects who receive all assigned doses within the assigned visit windows will be summarized overall and by treatment. The point estimates for the treatment-specific proportions and difference in proportions as well as corresponding 95% confidence intervals will be reported. A hypothesis test will be conducted using a Chi-square test at the 5% two-sided level of significance to formally compare the treatment arms.

Demographic, sexual history, and other characteristics of subjects will be presented overall and within HIV status subgroups at baseline and throughout follow-up. HIV status subgroups will be compared with respect to epidemiological characteristics using the Pearson Chi-square test for categorical measures and the t-test for independent samples or Wilcoxon rank sum test for continuous measures.

#### 11.4.4.3 Exploratory Efficacy Analysis

Any exploratory or sensitivity analyses to be performed to support any of the primary or secondary objectives will be specified in the Statistical Analysis Plan. As an example, the following alternative serological response classifications will be explored:

- Response: 4-fold decline in RPR titer compared to baseline, or RPR-negative (i.e., seroreversion).
- Non-Response: Less than 4-fold decline in RPR titer compared to baseline, and RPR-positive
- Failure: 4-fold or greater increase in RPR titer without a clear history of re-exposure

A blinded case review committee will review subjects' data to determine cases of re-exposure. If a subject has an observed serological response prior to meeting one of the Evaluable analysis population exclusion criteria, the subject will be included in the analyses as a responder. For subjects who have received BPG through the study and subsequently require retreatment for syphilis infection (i.e., per the local clinic's standard protocols), data collected following retreatment will not be used for analyses.

# 12 SOURCE DOCUMENTS AND ACCESS TO SOURCE DATA/DOCUMENTS

Each participating site will maintain appropriate medical and research records for this trial, in compliance with ICH E6, Section 4.9, and regulatory and institutional requirements for the protection of confidentiality of subjects. Each participating site will permit authorized representatives of DMID, its designees, and appropriate regulatory agencies to examine (and when required by applicable law, to copy) clinical study records for the purposes of quality assurance reviews, audits, monitoring, and evaluation of the study safety and progress. These representatives will be permitted access to all source data, which include, but are not limited to, hospital records, clinical and office charts, laboratory notes, memoranda, evaluation checklists, pharmacy dispensing records, recorded data from automated instruments, copies or transcriptions certified after verification as being accurate and complete, microfiches, photographic negatives, microfilm or magnetic media, x-rays, and subject files and records kept in the pharmacies, laboratories, and medico-technical departments involved in this clinical trial. Source data are all information, original records of clinical findings, observations, or other activities in a clinical trial necessary for the reconstruction and evaluation of this trial.

# 13 QUALITY CONTROL AND QUALITY ASSURANCE

Following a written DMID-accepted site quality management plan, the participating site is responsible for conducting routine quality assurance (QA) and quality control (QC) activities to internally monitor study progress and protocol compliance. The site PI will provide direct access to all trial-related sites, source data/data collection forms, and reports for the purpose of monitoring and auditing by the sponsor, and inspection by local and regulatory authorities. The site PI will ensure all study personnel are appropriately trained and applicable documentations are maintained on site.

The Statistical and Data Coordinating Center (SDCC) will implement QC procedures beginning with the data entry system and generate data QC checks that will be run on the database. Any missing data or data anomalies will be communicated to the site(s) for clarification and resolution.

# 14 ETHICS/PROTECTION OF HUMAN SUBJECTS

## 14.1 Ethical Standard

The site PI will ensure that this trial is conducted in full conformity with principles of the Belmont Report: Ethical Principles and Guidelines for the Protection of Human Subjects of Research of the National Commission for the Protection of Human Subjects of Biomedical and Behavioral Research (April 18, 1979) and codified in 45 CFR 46, 21 CFR 50 and 56, and ICH E6; 62 Federal Regulations 25691 (1997), if applicable. The site PI's institution will hold a current Federal Wide Assurance (FWA) issued by the Office of Human Research Protection (OHRP) for federally funded research.

## 14.2 Institutional Review Board

Prior to enrollment of subjects into this trial, the approved protocol and ICF will be reviewed and approved by the appropriate IRB listed on its FWA.

The responsible official for the IRB will sign the IRB letter of approval of the protocol before the start of this trial, and provide a copy to DMID. The IRB FWA number will be provided to DMID.

Should amendments to the protocol be required, the amendments will be written by the sponsor and provided to the site PI for submission to the IRB.

## 14.3 Informed Consent Process

The site PI, or designated study staff, will choose subjects in accordance with the eligibility criteria detailed in Section 5. Before any study procedures are performed, subjects must sign an ICF that complies with the requirements of 21 CFR Part 50, 45 CFR Part 46, and the local IRB.

Informed consent is a process that is initiated prior to an individual agreeing to participate in a trial and continuing throughout the individual's trial participation. Before any study procedures are performed, subjects will receive a comprehensive explanation of the proposed study procedures and study drug, including the nature and risks of the trial, alternate therapies, any known AEs, the investigational status of the components, and the other elements that are part of obtaining proper informed consent. Subjects will also receive a detailed explanation of the proposed use and disclosure of their protected health information, including specifically their specimens. Subjects will be allowed sufficient time to consider participation in the trial, after having the nature and risks of the trial explained to them, and will have the opportunity to discuss the trial with their family, friends, or legally authorized representative or think about it prior to agreeing to participate.

ICFs describing in detail the study drug, study procedures, risks, and possible benefits are given to subjects. The ICF must not include any exculpatory statements. ICFs will be IRB-approved, and subjects will be asked to read and review the appropriate document. Upon reviewing the appropriate document, the site PI (or designee) will explain the research study to subjects and answer any questions that may arise. Subjects must sign the ICF, and written documentation of the informed consent process is required prior to starting any study procedures/interventions being done specifically for the trial, including administering BPG.

DMID will provide the site PI, in writing, any new information that significantly affects the subjects' risk of receiving BPG. This new information will be communicated by the site PI to subjects who consent to participate in the trial in accordance with IRB requirements. The ICF will be updated, and subjects will be re-consented per IRB requirements, if necessary.

Study personnel may employ IRB-approved recruitment efforts before obtaining the subject’s consent; however, before any study procedures are performed to determine protocol eligibility, an ICF must be signed. Subjects will be given a copy of all ICFs that they sign.

By signing the ICF, subjects agree to complete all evaluations required by the trial, unless the subject withdraws voluntarily or is terminated from the trial for any reason.

The rights and welfare of subjects will be protected by emphasizing to subjects that the quality of their medical care will not be adversely affected if they decline to participate in or withdraw from this trial.

## 14.4 Exclusion of Women, Minorities, and Children (Special Populations)

This trial will be inclusive of female and male adults who meet the subject inclusion/exclusion criteria, regardless of religion or ethnic background. Should the outcome of this trial be deemed acceptable, additional trials may be initiated in other populations.

## 14.5 Subject Confidentiality

Subjects will have code numbers and will not be identified by name. Subject confidentiality is strictly held in trust by the participating site PIs, their study personnel, the sponsor(s), and their agents. This confidentiality is extended to cover testing of biological samples, in addition to the clinical information relating to subjects.

The study protocol, documentation, data, and all other information generated will be held in strict confidence. No information concerning the trial or the data will be released to any unauthorized third party without prior written approval of the sponsor.

All information provided by the sponsor and all data and information generated by the participating site as part of the trial (other than a subject's medical records) will be kept confidential by the site PI and other study personnel to the extent permitted by law. This information and data will not be used by the site PI or other study personnel for any purpose other than conducting the trial. These restrictions do not apply to: (1) information that becomes publicly available through no fault of the site PI or other study personnel; (2) information that is necessary to disclose in confidence to an IRB solely for the evaluation of the trial; (3) information that is necessary to disclose in order to provide appropriate medical care to a study subject; or (4) study results that may be published as described in Section 16. If a written contract for the conduct of the trial that includes confidentiality provisions inconsistent with this statement is executed, that contract's confidentiality provisions shall apply rather than this statement.

The study monitor, applicable regulatory authorities, such as the FDA, or other authorized representatives of the sponsor may inspect all documents and records required to be maintained by the site PI. This includes, but is not limited to, medical records (office, clinic, or hospital) and pharmacy records for the subjects in this trial. The participating sites will permit access to such records.

To protect privacy, a Certificate of Confidentiality (CoC) has been obtained. With this CoC, the researchers cannot be forced to release information that may identify the research subject, even by a court subpoena, in any federal, state, or local civil, criminal, administrative, legislative, or other proceedings. The researchers will use the CoC to resist any demands for information that would identify the subject, except as explained below.

The CoC cannot be used to resist a demand for information from personnel of the US Government that is used for auditing or evaluation of federally funded projects, like this trial, or for information that must be released in order to meet the requirements of the FDA.

A CoC does not prevent subjects from voluntarily releasing information about themselves or their involvement in this research. If any person or agency obtains a written consent to receive research information, then the researchers may not use the CoC to withhold that information.

The CoC does not prevent the researchers from reporting, without the subject's consent, information that would identify the subject as a participant in the research project regarding matters that must be legally reported, including: child and elder abuse, sexual abuse, or wanting to harm themselves or others.

## 14.6 Study Discontinuation

If the trial is discontinued, subjects who sign the ICF and are randomized and treated will continue to be followed for safety assessments. No further study drug will be administered

## 14.7 Future Use of Stored Specimens

Subjects will be consented to have leftover serum stored for future testing. Storage of samples for future testing is optional and is not a requirement for participation in the study. If the subject consents to storage and future use of specimens, specimens will be kept at the Central Laboratory (University of Alabama at Birmingham). Subjects will not be contacted with the results of these future research studies. Future testing on specimens will only occur to the extent authorized in each study site's ICF or as otherwise authorized under applicable law and after review and approval by DMID and the IRB of the researcher requesting the specimens.

Archived specimens will be identified only by the subject number and visit number, which will allow linkage of the specimens to study data but not to any personal identifiers.

There will be no direct benefit to subjects from allowing specimens to be stored and used for future purposes. However, the results may provide information that will help in the diagnosis or treatment of future patients.

Subjects' specimens will be kept until they are used up or destroyed. They may be used to develop new tests or products. In some instances, these may have potential commercial value. If a subject decides at any time that s/he does not want specimens stored for future research, s/he must contact the study staff who will then notify the laboratory/specimen archive staff, who will mark the specimens by adding a "destroy" label. Labeled specimens will be destroyed at the end of this study or will be removed from storage and destroyed as soon as possible.

# 15 DATA HANDLING AND RECORD KEEPING

The site PI is responsible for ensuring the accuracy, completeness, legibility, and timeliness of the data reported.

Data collection forms will be derived from the eCRFs and provided by the SDCC to record and maintain data for each subject enrolled in the study. All data collection forms should be completed in a neat, legible manner to ensure accurate interpretation of data. Black or blue ink is required to ensure clarity of reproduced copies. When making a change or correction, cross out the original entry with a single line and initial and date the change. Do not erase, overwrite, or use correction fluid or tape on the original.

Data reported in the eCRFs derived from the data collection forms should be consistent with the data collection forms, or the discrepancies should be explained.

The sponsor and/or its designee will provide guidance to site PIs and other study personnel on making corrections to the data collection forms and eCRFs.

## 15.1 Data Management Responsibilities

All data collection forms and laboratory reports must be reviewed by the clinical team and data entry personnel, who will ensure that they are accurate and complete. AEs must be recorded on the appropriate data collection form, assessed for severity and relationship, and reviewed by the site PI or appropriate sub-investigator.

Data collection is the responsibility of the study personnel at the participating sites under the supervision of the respective site PI. During the study, the site PI must maintain complete and accurate documentation for the study.

The SDCC for this study will be responsible for data management, quality review, analysis, and reporting of the study data.

## 15.2 Data Capture Methods

Clinical (including, but not limited to, AEs/SAEs, concomitant medications, medical history, physical assessments, and clinical laboratory values), and immunogenicity data will be entered into a 21 CFR 11-compliant Internet Data Entry System, Advantage eClinical®, provided by the SDCC. The data system includes password protection and internal quality checks, such as automatic range checks, to identify data that appear inconsistent, incomplete, or inaccurate. Clinical data will be entered directly from the data collection forms completed by the study personnel.

## 15.3 Types of Data

Data for this study will include clinical, safety, and outcome measures (e.g., clinical laboratory values and immunogenicity data).

## 15.4 Timing/Reports

A final report will be prepared following the availability of all the safety and laboratory data. Interim statistical reports may be generated as deemed necessary and appropriate by DMID. Safety and laboratory data summary reports may be generated for the DSMB.

After full analysis and final CSR reporting is complete, and upon request and DMID approval, the SDCC will provide the participating sites with a summary of results by treatment group. The participating sites requesting such information to share with study subjects must do so in compliance with their respective IRB guidelines.

## 15.5 Study Records Retention

Records and documents pertaining to the conduct of this study, including data collection forms, source documents, ICFs, laboratory test results, and medication inventory records, must be retained by the investigator for at least 2 years following the completion of this study. No records may be destroyed without written permission from the sponsor.

## 15.6 Protocol Deviations

A protocol deviation is any noncompliance with the clinical trial protocol, GCP, or protocol-specific MOP requirements. The noncompliance may be either on the part of the subject, the site PI, or the study site staff. As a result of deviations, corrective actions are to be developed by the site and implemented promptly.

These practices are consistent with ICH E6:

4.5 Compliance with Protocol, sections 4.5.1, 4.5.2, and 4.5.3

5.1 Quality Assurance and Quality Control, section 5.1.1

5.20 Noncompliance, sections 5.20.1, and 5.20.2.

It is the responsibility of the site PI to use continuous vigilance to identify and report deviations within 5 working days of identification of the protocol deviation, or within 5 working days of the scheduled protocol-required activity. All deviations must be promptly reported to DMID, via the SDCC's Advantage eClinical®.

Note: Those sites participating in trials with a designated 'central unit' will follow the reporting requirements specified in their protocols and MOPs. The 'central unit' will be responsible for submission of the protocol deviation information to TRI-ICON/DMID-CROMS.

All protocol deviations, as defined above, must be addressed in study subject data collection forms. A completed copy of the DMID Protocol Deviation Form must be maintained in the regulatory file, as well as in the subject's study chart. Protocol deviations must be sent to the local IRB per their guidelines. The site PI and other study personnel are responsible for knowing and adhering to their IRB requirements.

# 16 PUBLICATION POLICY

All investigators funded by the NIH must submit or have submitted for them to the National Library of Medicine's PubMed Central (http://www.ncbi.nlm.nih.gov/pmc/) an electronic version of their final, peer-reviewed manuscripts upon acceptance for publication, to be made publicly available no later than 12 months after the official date of publication. The NIH Public Access Policy ensures the public has access to the published results of NIH-funded research. It requires investigators to submit final peer-reviewed journal manuscripts that arise from NIH funds to the digital archive PubMed Central upon acceptance for publication. Further, the policy stipulates that these papers must be accessible to the public on PubMed Central no later than 12 months after publication.

Refer to:

- NIH Public Access Policy, http://publicaccess.nih.gov/
- NIH Office of Extramural Research (OER) Grants and Funding, http://grants.nih.gov/grants/oer.htm

As of January 2018, all clinical trials supported by the NIH must be registered on ClinicalTrials.gov no later than 21 days after the enrollment of the first subject. Results of all clinical trials supported by the NIH generally need to be submitted no later than 12 months following the primary completion date. A delay of up to 2 years is available for trials that meet certain criteria and have applied for certification of delayed posting.

As part of the result posting, a copy of this protocol (and its amendments) and a copy of the Statistical Analysis Plan will be posted on ClincialTrials.gov.

For this trial, the responsible party is DMID, which will register the trial and post results.

The responsible party does not plan to request certification of delayed posting.

Refer to:

- Public Law 110-85, Section 801, Clinical Trial Databases
- 42CFR11
- NIH NOT-OD-16-149

# 17 LITERATURE REFERENCES

1. Hook, E.W., *Syphilis.* Lancet, 2017. **389**(10078): p. 1550-1557.
2. *CDC Sexually transmitted disease surveillance 2016*. 2017, U.S. Department of Health and Human Services: Atlanta.
3. *CDC Sexually transmitted disease surveillance 2015*. 2016, U.S. Department of Health and Human Services: Atlanta.
4. *CDC Sexually transmitted disease surveillance 2012*. 2013, U.S. Department of Health and Human Services: Atlanta.
5. Workowski, K.A. and G.A. Bolan, *Sexually transmitted diseases treatment guidelines, 2015.* MMWR Recomm Rep, 2015. **64**(Rr-03): p. 1-137.
6. Sena, A.C., et al., *Predictors of serological cure and serofast state after treatment in HIV-negative persons with early syphilis.* Clin Infect Dis, 2011. **53**(11): p. 1092-9.
7. Rolfs, R.T., et al., *A randomized trial of enhanced therapy for early syphilis in patients with and without human immunodeficiency virus infection. The Syphilis and HIV Study Group.* N Engl J Med, 1997. **337**(5): p. 307-14.

# APPENDIX A: SCHEDULE OF EVENTS

| Procedures | | **Visit 1 – Screening/ Enrollment** (Day 1) | **Contact** (approx. 24 hours after Visit 1) | **Visit 2** (Week 1 – Day 7 to 13) | **Visit 3** (Week 2 – 6 to 12 days after Visit 2) | **Visit 4** (Month 1 – Day 30 ± 7 days) | **Visit 5** (Month 3 – Day 90 ± 21 days) | **Visit 6** (Month 6 – Day 180 ± 21 days) | **Visit 7** (Month 9 – Day 270 ± 28 days) | **Visit 8** (Month 12 – Day 360 ± 28 days) | **Early Termination** | **Unscheduled Visit**[15] |
|---|---|---|---|---|---|---|---|---|---|---|---|---|
| Informed consent | | X | | | | | | | | | | |
| Demographics | | X | | | | | | | | | | |
| Eligibility criteria | | X | | | | | | | | | | |
| Medical history[1] | | X | | X | X | X | X | X | X | X | X | X |
| Sexual history[2] | | X | | X | X | X | X | X | X | X | X | X |
| Targeted physical exam[3] | | X | | X | X | X | X | X | X | X | X | X |
| Pregnancy test[4] | | (X) | | (X) | (X) | (X) | (X) | (X) | (X) | (X) | (X) | (X) |
| Concomitant medications[5] | | X | | X | X | X | X | X | X | X | X | X |
| Specimen collection | RPR titer | X | | X | X | X | X | X | X | X | X | |
| | Storage | X | | X | X | X | X | X | X | X | X | |
| | CD4 count[6] | (X) | | | | (X) | | (X) | | (X) | (X) | |
| | HIV testing[7] | (X) | | | | | (X) | (X) | (X) | (X) | (X) | |
| | Other STI screening/ diagnosis[8] | X | | (X) | (X) | (X) | (X) | (X) | (X) | (X) | (X) | (X) |
| | Oral cavity and lesions (if present) swabbed[9] | (X) | | (X) | (X) | (X) | (X) | (X) | (X) | (X) | (X) | (X) |
| Randomization | | X | | | | | | | | | | |
| BPG administered[10] | | X | | (X) | (X) | | | | | | | |
| Contact information collected/reviewed[11] | | X | | X | X | X | X | X | X | X | X | X |
| Jarisch-Herxheimer checklist[12] | | X | X | (X) | | | | | | | | |
| Jarisch-Herxheimer reaction assessed[13] | | | X | (X) | | | | | | | | |
| Resolution of syphilis signs assessed | | | | X | X | | | | | | | |
| AEs/SAEs assessed[14] | | X | | X | X | X | | | | | | X |

(X) – As indicated/appropriate. Refer to footnotes below and protocol Section 7.

[1] At Visit 1, collect complete medical history (including review of medical records for the past 14 days, if available). At subsequent visits, review medical history (including interval medical records, if available) and update as appropriate.
[2] At Visit 1, collect sexual history for the past 60 days. At subsequent visits, collect interim sexual history since last visit.
[3] Targeted physical exam includes vital signs: temperature, heart rate, respiration rate, and blood pressure; and genital, rectal, oral, skin, and lymph node examinations.
[4] Perform on subjects of childbearing potential. At Visit 1, urine or serum pregnancy test is permitted. At subsequent visits, perform urine pregnancy test.
[5] At Visit 1, record concomitant medications taken in the last 30 days before initiating BPG. At subsequent visits, record all concomitant medications taken since the last visit and update previously recorded medications as appropriate.
[6] Collect for subjects with known HIV infection whose medical records do not include a CD4 count in the past 30 days; at Visit 4, this is only applicable to subjects newly found to have HIV infection from Visit 1 to Visit 4 (inclusive).
[7] Perform for subjects who do not have a previously documented positive HIV test result using locally available tests (e.g., nucleic acid tests (NATs), antibody/antigen tests).
[8] At Visit 1, collect specimens at sites of exposure for screening/diagnosis of chlamydia and gonorrhea for all subjects who have not been tested since last sexual activity and for all subjects who have been sexually active in the past 14 days and for other STIs as indicated by local standard of care and subject history. At subsequent visits, collect specimens at sites of

exposure for STI testing per local standard of care and subject history.
[9]It is optional to swab subject's oral cavity and lesions, if lesions are present. See Section 8.2.2.
[10] At Visit 1, administer BPG to all subjects. At Visits 2 and 3, administer BPG to subjects in Arm 2 only.
[11]At Visit 1, collect contact information. At subsequent visits, review contact information and update if needed.
[12] Distribute checklist at Visit 1. Subject refers to completed checklist during Jarisch-Herxheimer assessment; see Sections 7 and 8.1.
[13]Assess per protocol Section 8.1. If subject is not reached, assess at Visit 2.
[14] Assess and record all AEs/SAEs (including solicited reactogenicity AEs and other unsolicited AEs).
[15] At Unscheduled Visits, any of the specified evaluations that are relevant to the subject may be performed at the discretion of the site PI.

## APPENDIX B: TABLE FOR GRADING THE SEVERITY OF ADVERSE EVENTS

| PARAMETER | GRADE 1 - MILD | GRADE 2 - MODERATE | GRADE 3 - SEVERE |
|---|---|---|---|
| Clinical adverse event **NOT** identified below | Mild symptoms causing no or minimal interference with usual social and functional activities with intervention not indicated | Moderate symptoms causing greater than minimal interference with usual social and functional activities with intervention indicated | Severe symptoms causing inability to perform usual social and functional activities with intervention or hospitalization indicated |
| **Vital signs** | | | |
| Temperature | 38.0 to <38.6°C | 38.6 to <39.3°C | ≥39.3°C |
| Blood pressure | 140 – 159 mmHg systolic OR 90 – 99 mmHg diastolic | 160 – 179 mmHg systolic OR 100 – 109 mmHg diastolic | ≥180 mmHg systolic OR ≥110 mmHg diastolic OR hospitalization indicated |
| Pulse | 101-115 or 50-54 or 45-50 if baseline <60bpm | 116-130 or 45-49 or 40-44 if baseline <60bpm | >130 or ventricular dysrhythmias or <45 or <40 if baseline <60bpm |
| Respiration | 23-25 | 26-30 | ≥30 |
| **Local injection site** | | | |
| Pain or tenderness (report only one) | Symptoms causing no or minimal interference with usual social and functional activities | Symptoms causing greater than minimal interference with usual social and functional activities | Symptoms causing inability to perform usual social and functional activities |
| Erythema or redness (report only one) | 2.5 to <5 cm in greatest diameter AND Symptoms causing no or minimal interference with usual social and functional activities | ≥5 to <10 cm in greatest diameter OR Symptoms causing greater than minimal interference with usual social and functional activities | ≥10 cm in greatest diameter OR Ulceration OR Secondary infection OR Sterile abscess OR Symptoms causing inability to perform usual social and functional activities |
| Induration or swelling (report only one) | 2.5 to <5 cm in greatest diameter AND Symptoms causing no or minimal interference with usual social and functional activities | ≥5 to <10 cm in greatest diameter OR Symptoms causing greater than minimal interference with usual social and functional activities | ≥10 cm in greatest diameter OR Ulceration OR Secondary infection OR Sterile abscess OR Symptoms causing inability to perform usual social and functional activities |

| **Systemic** | | | |
|---|---|---|---|
| Anaphylaxis ** | -- | -- | Symptomatic bronchospasm, with or without urticaria; parenteral intervention indicated; allergy-related edema or angioedema; hypotension |
| ***Definition*: A disorder characterized by an acute inflammatory reaction resulting from the release of histamine and histamine-like substances from mast cells, causing a hypersensitivity immune response. Clinically, it presents with breathing difficulty, dizziness, hypotension, cyanosis, and loss of consciousness, and may lead to death.* | | | |
| Acute allergic reaction | Localized urticaria with no intervention indicated | Localized urticaria with intervention indicated OR Mild angioedema with no intervention indicated | Generalized urticaria OR Angioedema with intervention indicated OR Symptoms of mild bronchospasm |
| Hypersensitivity | Transient flushing or rash | Rash; flushing; urticaria; dyspnea | Symptomatic bronchospasm with or without urticaria; parenteral medication indicated; allergy-related edema or angioedema; hypotension |
| Fever | 38.0 to <38.6°C or 100.4 to <101.5°F | ≥38.6 to <39.3°C or ≥101.5 to <102.7°F | ≥39.3°C or ≥102.7°F |
| Chills | Symptoms causing no or minimal interference with usual social and functional activities | Symptoms causing greater than minimal interference with usual social and functional activities | Symptoms causing inability to perform usual social and functional activities |
| Headache | Symptoms causing no or minimal interference with usual social and functional activities | Symptoms causing greater than minimal interference with usual social and functional activities | Symptoms causing inability to perform usual social and functional activities |
| Myalgias (generalized) | Symptoms causing no or minimal interference with usual social and functional activities | Symptoms causing greater than minimal interference with usual social and functional activities | Symptoms causing inability to perform usual social and functional activities |
| Fatigue or Malaise (report only one) | Symptoms causing no or minimal interference with usual social and functional activities | Symptoms causing greater than minimal interference with usual social and functional activities | Symptoms causing inability to perform usual social and functional activities |

| | | | |
|---|---|---|---|
| Decreased appetite | Loss of appetite without decreased oral intake | Loss of appetite associated with decreased oral intake without significant weight loss | Loss of appetite associated with significant weight loss |
| **Dermatological** | | | |
| Skin rash | Localized rash | Diffuse rash OR Target lesions | Diffuse rash AND Vesicles or limited number of bullae or superficial ulcerations of mucous membrane limited to one site |
| **Gastrointestinal** | | | |
| Nausea | Transient (< 24 hours) or intermittent AND No or minimal interference with oral intake | Persistent nausea resulting in decreased oral intake for 24-48 hours | Persistent nausea resulting in minimal oral intake for > 48 hours OR Rehydration indicated (e.g., IV fluids) |
| Vomiting | Transient (< 24 hours) or intermittent AND No or minimal interference with oral intake | Frequent episodes with no or minimal dehydration | Persistent vomiting resulting in orthostatic hypotension OR Rehydration indicated (e.g., IV fluids) |
| Diarrhea | Transient or intermittent episodes of unformed stools OR Increase of ≤ 3 stools over baseline per 24-hour period | Persistent episodes of unformed to watery stools OR Increase of 4-6 stools over baseline per 24-hour period | Increase of ≥ 7 stools over baseline per 24-hour period OR IV fluid replacement indicated |
| Abdominal discomfort | Symptoms causing no or minimal interference with usual social and functional activities | Symptoms causing greater than minimal interference with usual social and functional activities | Symptoms causing inability to perform usual social and functional activities |

CLINICAL RESEARCH IN INFECTIOUS DISEASES

# STATISTICAL ANALYSIS PLAN
# for
# DMID Protocol: 17-0101

# Study Title:
# A Phase 4 Comparative Trial of Benzathine Penicillin G 2.4 Million Units Administered as a Single Dose versus Three Successive Weekly Doses for Treatment of Early Syphilis in Subjects with or without HIV Infection

# NCT03637660

**Version 1.0**

**DATE: 22-Dec-2022**

THIS COMMUNICATION IS PRIVILEGED AND CONFIDENTIAL

## STUDY TITLE

| | |
|---|---|
| **Protocol Number Code:** | **DMID Protocol: 17-0101** |
| **Protocol Version:** | **Version 4.0** |
| **Development Phase:** | Phase IV |
| **Products:** | Benzathine Penicillin G |
| **Form/Route:** | Intramuscular |
| **Indication Studied:** | Syphilis |
| **Sponsor:** | Division of Microbiology and Infectious Diseases<br>National Institute of Allergy and Infectious Diseases<br>National Institutes of Health |
| **Clinical Trial Initiation Date:** | 12 September 2018 |
| **Date of the Analysis Plan:** | 22 December 2022 |
| **Version Number:** | 1.0 |

This study was performed in compliance with Good Clinical Practice.

*Information contained in this publication is the property of Division of Microbiology and Infectious Diseases and is confidential. This information may not be disclosed to third parties without written authorization from Division of Microbiology and Infectious Diseases. This report may not be reproduced, stored in a retrieval system or transmitted in any form or by any means - electronic, mechanical, recording or otherwise - without the prior authorization from Division of Microbiology and Infectious Diseases. This document must be returned to Division of Microbiology and Infectious Diseases upon request.*

# TABLE OF CONTENTS

STUDY TITLE ..... II
TABLE OF CONTENTS ..... III
LIST OF ABBREVIATIONS ..... VI
1. PREFACE ..... 1
2. INTRODUCTION ..... 2
2.1. Study background ..... 2
2.2. Purpose of the Analyses ..... 2
3. STUDY OBJECTIVES AND ENDPOINTS ..... 3
3.1. Study Objectives ..... 3
3.1.1. Primary Objective ..... 3
3.1.2. Secondary Objectives ..... 3
3.2. Study Outcome Measures ..... 3
3.2.1. Primary Outcome Measure ..... 3
3.2.2. Secondary Outcome Measures ..... 3
3.3. Study Definitions and Derived Variables ..... 4
3.3.1. Serological Response to Therapy ..... 4
3.3.1.1. Primary Outcome Analysis ..... 4
3.3.1.2. Secondary Outcome Analysis ..... 4
3.3.1.3. Sensitivity Analysis for the Primary Outcome ..... 4
3.3.1.4. Exploratory Analysis for the Primary Outcome ..... 5
3.3.2. Treatment Adherence ..... 5
3.3.3. Jarisch-Herxheimer Reaction ..... 6
3.3.4. Baseline Value ..... 6
4. INVESTIGATIONAL PLAN ..... 7
4.1. Overall Study Design and Plan ..... 7
4.2. Discussion of Study Design, Including the Choice of Control Groups ..... 8
4.3. Selection of Study Population ..... 8
4.4. Treatments ..... 9
4.4.1. Treatments Administered ..... 9
4.4.2. Identity of Investigational Product(s) ..... 10
4.4.3. Method of Assigning Subjects to Treatment Groups (Randomization) ..... 10

**Table of Contents** *(continued)*


4.4.4. Selection of Doses in the Study ....10
4.4.5. Selection and Timing of Dose for Each Subject ....11
4.4.6. Blinding ....11
4.4.7. Prior and Concomitant Therapy ....11
4.5. Efficacy (Immunogenicity) and Safety Variables ....11
5. SAMPLE SIZE CONSIDERATIONS ....14
5.1. Study with Planned Sample Size ....14
5.2. Study with Smaller Sample Size ....14
6. GENERAL STATISTICAL CONSIDERATIONS ....17
6.1. General Principles ....17
6.1.1. Pseudo Code ....17
6.2. Timing of Analyses ....18
6.3. Analysis Populations ....18
6.3.1. Safety Analyses ....18
6.3.2. Efficacy Analyses ....19
6.3.2.1. Intent-to-Treat Population ....19
6.3.2.2. Modified Intent-to-Treat (mITT) Population ....19
6.3.2.3. Evaluable Population ....19
6.3.2.4. Analysis Population Summaries ....20
6.4. Covariates and Subgroups ....20
6.5. Missing Data ....20
6.6. Interim Analyses and Data Monitoring ....21
6.7. Multicenter Studies ....22
6.8. Multiple Comparisons/Multiplicity ....22
7. STUDY SUBJECTS ....23
7.1. Disposition of Subjects ....23
7.2. Protocol Deviations ....23
8. EFFICACY EVALUATION ....24
8.1. Primary Efficacy Analysis ....24
8.1.1. Sensitivity Analyses for Primary Efficacy Outcome ....24
8.2. Secondary Efficacy Analyses ....24




PRIVILEGED AND CONFIDENTIAL COMMUNICATION

**Table of Contents** *(continued)*

8.2.1. Efficacy by 12 Months ....24
8.2.2. Efficacy by 6 Months Among Subjects with and without HIV Infection ....25
8.2.3. Efficacy by 12 Months Among Subjects with and without HIV Infection ....25
8.2.4. Treatment Compliance by Treatment Group ....25
8.2.5. Demographics and Baseline Characteristics by HIV status ....26
8.3. Exploratory Efficacy Analyses ....26
8.4. Clinical Laboratory Evaluations ....26
9. SAFETY EVALUATION ....27
9.1. Prior Conditions ....27
9.1.1. Prior and Concurrent Medical Conditions ....27
9.1.2. Prior and Concomitant Medications ....27
9.2. Measurements of Treatment Compliance ....27
9.3. Adverse Events ....27
9.3.1. Solicited Events and Symptoms ....28
9.3.2. Unsolicited Adverse Events ....28
9.4. Deaths, Serious Adverse Events and other Significant Adverse Events ....29
9.5. Pregnancies ....29
9.6. Vital Signs and Physical Evaluations ....29
9.7. Other Safety Measures ....29
10. PHARMACOKINETICS ....30
11. IMMUNOGENICITY ....31
12. OTHER ANALYSES ....32
13. REPORTING CONVENTIONS ....33
14. TECHNICAL DETAILS ....34
15. SUMMARY OF CHANGES IN THE CONDUCT OF THE STUDY OR PLANNED ANALYSES ....35
16. REFERENCES ....36
17. LISTING OF TABLES, FIGURES, AND LISTINGS ....37
APPENDICES ....38
APPENDIX 1. TABLE MOCK-UPS ....39
APPENDIX 2. FIGURE MOCK-UPS ....96
APPENDIX 3. LISTINGS MOCK-UPS ....106



PRIVILEGED AND CONFIDENTIAL COMMUNICATION

# LIST OF ABBREVIATIONS

| | |
|---|---|
| AE | Adverse Event |
| ALT | Alanine Aminotransferase |
| AST | Aspartate Aminotransferase |
| BP | Blood Pressure |
| BPG | Benzathine Penicillin G |
| BUN | Blood Urea Nitrogen |
| C | Celsius |
| CI | Confidence Interval |
| CRF | Case Report Form |
| DMID | Division of Microbiology and Infectious Diseases |
| DSMB | Data and Safety Monitoring Board |
| EDC | Electronic Data Capture |
| ELISA | Enzyme-linked Immunosorbent Assay |
| ER | Emergency Room |
| F | Fahrenheit |
| GGT | Gamma Glutamyl Transferase |
| GMT | Geometric Mean Titer |
| GMFR | Geometric Mean Fold Rise |
| ICH | International Conference on Harmonisation |
| IRB | Institutional Review Board |
| ITT | Intention to Treat |
| L | Liter |
| LLN | Lower Limit of Normal |
| mcg | Microgram |
| MedDRA | Medical Dictionary for Regulatory Activities |
| mEq | Milliequivalent |
| mg | Milligram |
| mITT | Modified Intention to Treat |
| mL | Milliliter |
| MAR | Missing at Random |
| MCAR | Missing Completely at Random |

**List of Abbreviations** *(continued)*

| | |
|---|---|
| MNAR | Missing Not at Random |
| N | Number (typically refers to subjects) |
| NIH | National Institutes of Health |
| PI | Principal Investigator |
| PP | Per Protocol |
| PT | Preferred Term |
| RBC | Red Blood Cell |
| RCD | Reverse Cumulative Distribution |
| RPR | Rapid Plasma Reagin |
| SAE | Serious Adverse Event |
| SD | Standard Deviation |
| SDCC | Statistical and Data Coordinating Center |
| SMC | Safety Monitoring Committee |
| SOC | System Organ Class |
| SOP | Standard Operating Procedures |
| TFL | Tables, figures, and listings |
| TPPA | Treponema pallidum Particle Agglutination |
| U | Units |
| ULN | Upper Limit of Normal |
| WBC | White Blood Cell |
| WHO | World Health Organization |


PRIVILEGED AND CONFIDENTIAL COMMUNICATION

# 1. PREFACE

The Statistical Analysis Plan (SAP) for "A Phase 4 Comparative Trial of Benzathine Penicillin G 2.4 Million Units Administered as a Single Dose versus Three Successive Weekly Doses for Treatment of Early Syphilis in Subjects with or without HIV Infection" (DMID Protocol 17-0101) describes and expands upon the statistical information presented in the protocol.

This document describes all planned analyses and provides reasons and justifications for these analyses. It also includes sample tables, figures, and listings (TFL) planned for the final analyses. Regarding the final analyses and Clinical Study Report (CSR), this SAP follows the International Conference on Harmonisation of Technical Requirements for Registration of Pharmaceuticals for Human Use (ICH) Guidelines, as indicated in Topic E3 (Structure and Content of Clinical Study Reports), and more generally is consistent with Topic E8 (General Considerations for Clinical Trials) and Topic E9 (Statistical Principles for Clinical Trials). This SAP also follows the FDA guidance regarding the COVID-19 public health emergency. 1. Contingency measures implemented to manage study conduct during disruption of the study as a result of COVID-19 control measures. 2. All participants affected by the COVID-19 will be highlighted. 3. Analyses and corresponding discussions that address the impact of implemented contingency measures. 4. Any protocol deviation due to COVID-19 will be documented.

The structure and content of the SAP provides sufficient detail to meet the requirements identified by the FDA and ICH, while all work planned and reported for this SAP will follow internationally accepted guidelines published by the American Statistical Association and the Royal Statistical Society for statistical practice.

This document contains four sections: (1) a review of the study design, (2) general statistical considerations, (3) comprehensive statistical analysis methods for efficacy and safety outcomes, and (4) a list of proposed tables and figures. Within the table, figure, and listing mock-ups (Appendices Appendix 1, Appendix 2, and Appendix 3), references to CSR sections are included. Any deviation from this SAP will be described and justified in protocol amendments and/or in the CSR, as appropriate. The reader of this SAP is encouraged to also review the study protocol for details on conduct of the study and the operational aspects of clinical assessments.

# 2. INTRODUCTION

## 2.1. Study background

Despite syphilis being a major public health problem for well over a century, and its treatment recommendations being unchanged for nearly 50 years, questions about syphilis and its management are amongst the most common questions to arise regarding sexually transmitted infections (STIs). The human immunodeficiency virus (HIV) epidemic has compounded these problems, as syphilis is now appreciated as both a risk marker and risk factor for HIV acquisition, and case reports and uncontrolled case series have led to recurring, unresolved questions regarding the optimal management of syphilis with respect to treatment regimens, serological follow-up, and risks for treatment failure[2]. Syphilis in the US is resurging, with rates of primary and secondary (P&S) syphilis increasing by 74% from 2012 to 2016 and disproportionately among HIV-infected persons[3]. Further rising P&S rates in women led to a 28% increase in congenital syphilis cases in the US between 2015 and 2016[4]. No large prospective studies in the US have provided data on outcomes of recommended single-dose penicillin therapy versus widely used but unstudied multiple-dose therapy for persons with early syphilis and HIV co-infection for over 30 years. In addition, there are no contemporary data describing serological responses to syphilis therapy at >6 months following therapy, raising questions as to how to best follow the ~20% of "serofast" patients who fail to serologically respond at >6 months following therapy. These questions represent a pressing public health need that is best addressed through a multicenter, randomized clinical trial (RCT).

Infections due to *Treponema pallidum* are the third most commonly reported infection in the US and are a global public health priority. Currently, P&S syphilis rates in the US are increasing faster than for any other reportable STI, and over half of P&S syphilis cases in men who have sex with men (MSM), the group that accounts for most new cases, are occurring among persons with HIV co-infection[4] Untreated syphilis is a significant cause of morbidity in children born to mothers with untreated infection, may cause serious neurological or cardiovascular disease, and is both a proven risk factor and risk marker for HIV acquisition. Syphilis also remains a glaring example of STI health disparities, with P&S syphilis rates among African Americans in the US being more than six times those of non-Hispanic whites [5].

Penicillin is currently the only recommended treatment for syphilis (alternative therapies may be used in persons with penicillin allergy[6]. However, recent studies have shown that ~20% of persons treated for early syphilis with recommended, single-dose BPG 2.4 MU are "serofast" (do not show the desired 4-fold decline in serological test for syphilis titers 6 months following therapy)[6, 7], and concerns have been raised as to whether this regimen adequately treats early syphilis in HIV-infected persons. A well-conducted, multicenter RCT in the 1990s showed that higher doses of penicillin given in combination with the recommended BPG regimen did not significantly change the serological response to therapy at 3, 6, or 12 months[7], but there are no RCTs evaluating whether a longer duration of BPG therapy improves the serological response to therapy among persons with or without HIV infection. While this earlier study addressed questions regarding daily penicillin doses for early syphilis treatment, the question of duration remains unresolved. As a result, there is substantial variation in the treatment of early syphilis, with many providers using multiple injected doses of BPG or treating serofast persons who have no evidence of active infection.

## 2.2. Purpose of the Analyses

These analyses will assess the efficacy and safety of a single dose or three successive weekly doses regimen of Benzathine Penicillin G 2.4 Million units for the treatment of early Syphilis in subjects with or without HIV infection and will be included in the clinical study report.

# 3. STUDY OBJECTIVES AND ENDPOINTS

## 3.1. Study Objectives

### 3.1.1. Primary Objective

To compare the serological response to therapy in subjects with early (primary, secondary, or early latent) syphilis treated with BPG 2.4 MU once or weekly for three successive weeks

### 3.1.2. Secondary Objectives

1. To determine if the difference in response to therapy between treatment arms by Month 6 differs among subjects with or without HIV infection
2. To determine the impact of multiple BPG injected doses on subject compliance with study product and adherence to scheduled visits
3. To determine the incidence and manifestations of the Jarisch-Herxheimer reaction among subjects treated for early syphilis with BPG
4. To collect prospective data up to Month 12 on the serological response to therapy in subjects treated for early syphilis with either BPG regimen
5. To compare epidemiological characteristics of early syphilis among subjects with or without HIV infection

## 3.2. Study Outcome Measures

### 3.2.1. Primary Outcome Measure

The proportion of subjects in each treatment group with a 4-fold decline in rapid plasma reagin (RPR) titers or seroreversion, simultaneously measured in sera collected throughout study participation at a single reference laboratory, by Month 6

### 3.2.2. Secondary Outcome Measures

1. The proportion of subjects in each treatment group with a 4-fold decline in RPR titers or seroreversion by Month 6 among subjects with or without HIV infection
2. The proportion of subjects in each treatment group with a 4-fold decline in RPR titers or seroreversion by Month 12
3. The proportion of subjects in each treatment group with a 4-fold decline in RPR titers or seroreversion by Month 12 among subjects with or without HIV infection
4. The proportion of subjects (overall and in each treatment group) who receive all assigned doses within the assigned visit windows
5. The proportion of subjects in each treatment group who report Jarisch-Herxheimer reaction manifestations (fever, intensification of rash, myalgia, and other systemic symptoms) occurring within approximately 24 hours of initial BPG administration
6. Demographics, sexual history, and socio-epidemiologic characteristics at baseline and sexual history through Months 6 and 12 among subjects with or without HIV infection and overall


PRIVILEGED AND CONFIDENTIAL COMMUNICATION

## 3.3. Study Definitions and Derived Variables

### 3.3.1. Serological Response to Therapy

#### 3.3.1.1. Primary Outcome Analysis

For the primary analysis, serological response to therapy by Month 6 will be defined as follows, where available RPR results from all visits prior to the end of month 6 visit window (i.e. scheduled visits up to and including the Month 6 visit, early termination visit, or any unscheduled visit that occurred prior to the end of month 6 visit window), will be evaluated:

- 4-fold or greater decline in RPR titer at any visit prior to the end of month 6 visit window compared to baseline, OR
- RPR-negative at any visit prior to the end of month 6 visit window (i.e., seroreversion).

It is possible that a subject reaches 4-fold decline in RPR titer at an earlier visit but increases again later to less than 4-fold decline. In this case, it will be classified as a serological response.

For the primary analysis, subjects who do not meet either of the above criteria will be classified as not experiencing serological response by Month 6. For subjects who have received BPG through the study and subsequently require retreatment for syphilis infection (i.e., per the local clinic's standard protocols), data collected after retreatment will not be used for analyses. See Section 6.5 for appropriate handling of visits with missing RPR test and titer results.

#### 3.3.1.2. Secondary Outcome Analysis

For the secondary analysis, serological response to therapy by Month 12 will be defined as follows, where available RPR results from all visits prior to the end of Month 12 visit window (i.e. scheduled visits and early termination visit that occurred between scheduled visits up to and including the Month 12 visit), will be evaluated:

- 4-fold or greater decline in RPR titer at any visit prior to or including the Month 12 compared to baseline, OR
- RPR-negative at any visit prior to or including the Month 12 (i.e., seroreversion).

It is possible that a subject reaches 4-fold decline in RPR titer at an earlier visit but increases again later to less than 4-fold decline. In this case, it will be classified as a serological response.

For the secondary analysis, subjects who do not meet either of the above criteria will be classified as not experiencing serological response by Month 12. For subjects who have received BPG through the study and subsequently require retreatment for syphilis infection (i.e., per the local clinic's standard protocols), data collected following retreatment will not be used for analyses. See Section 6.5 for appropriate handling of visits with missing RPR test and titer results.

#### 3.3.1.3. Sensitivity Analysis for the Primary Outcome

We are going to do two sensitivity analyses for the primary outcome.

1. Due to the COVID-19 pandemic, a certain number of subjects discontinued the study treatment or early terminated from the study. If these subjects do not have enough data to demonstrate serological response, they will be classified as failure. By removing these subjects from the analysis, we will

reduce cases of false failure. This analysis will be performed with ITT, mITT and evaluable populations

2. For the second sensitivity analysis of the primary outcome, serological response to therapy by Month 6 will be defined as follows, where available RPR results from the last available visit prior to the end of month 6 visit window (i.e. scheduled visits up to and including the Month 6 visit and early termination visit that occurred prior to the end of month 6 visit window), will be evaluated:

- 4-fold or greater decline in RPR titer at the last available visits prior to the end of month 6 visit window compared to baseline, OR
- RPR-negative at the last available visit prior to the end of month 6 visit window (i.e., seroreversion).

In this definition, if a subject reaches 4-fold decline in RPR titer at an earlier visit but increases again later to less than 4-fold decline, they will be classified as a serological non-response.

#### 3.3.1.4. Exploratory Analysis for the Primary Outcome

Exploratory analyses of the primary outcome will consider the following alternative serological response classifications:

- Response:
  - 4-fold or more decline in RPR titer at any visit prior to the end of month 6 visit window compared to baseline,

    or
  - RPR-negative (i.e., seroreversion) at any visit prior to the end of month 6 visit window.
- Non-Response:
  - RPR-positive at all visits,

    and
  - Less than 4-fold decline in RPR titer at all visits prior to the end of month 6 visit window compared to baseline,

    and
  - Less than 4-fold increase in RPR titer at all visits prior to the end of month 6 visit window
- Failure:
  - 4-fold or greater increase in RPR titer without a clear history of re-exposure at any visit prior to the end of month 6 visit window,

    and
  - Less than 4-fold decline in RPR titer at all visits compared to baseline.

### 3.3.2. Treatment Adherence

All BPG injections will be directly observed by the administering clinician. Compliance with study product will be defined as the subject received all assigned doses within the assigned visit windows. A subject is not adherent to study product if they miss at least one dose or receive at least one dose out of the visit window. An exception to this rule is that a subject is considered as compliant to the study product if they received all

doses according to the schedule they were given, even if the schedule itself might be out of the normal visit window.

### 3.3.3. Jarisch-Herxheimer Reaction

Approximately 24 - 48 hours after Visit 1, subjects are contacted and evaluated for symptoms of a Jarisch-Herxheimer reaction. The symptoms of a Jarisch-Herxheimer reaction include feverishness, chills, myalgia, weakness, flushing, worsening of skin rash, tachycardia (i.e., fast heartbeat), heart palpitations, arthralgia, nausea, headache, and dizziness. The incidence and severity of each symptom will be reported.

### 3.3.4. Baseline Value

The baseline value will be defined as the last value obtained prior to the first dose of study product. If a subject did not receive any dose, the enrollment date will be used as the first dose date for any determinations. For efficacy outcomes, the baseline value which is simultaneously measured in sera collected throughout study participation at a single reference laboratory will be used as baseline when calculating the serological response. The HIV status at baseline was determined by a two-step process. Firstly, the participant will be asked if she/he has a known HIV positive status, if the answer is yes, then baseline HIV status will be classed as positive. If the participant indicates that their status is not known to them, usually an HIV test is performed to determine the HIV status at baseline. But if a HIV test wasn't performed at baseline, the HIV status at baseline will be unknown.

# 4. INVESTIGATIONAL PLAN

## 4.1. Overall Study Design and Plan

This is a Phase 4, open-label, multicenter trial to evaluate the efficacy of a single injected dose of BPG 2.4 MU (Arm 1) compared to three successive weekly injected doses of BPG 2.4 MU (Arm 2) for treatment of early syphilis in HIV-infected and HIV-uninfected subjects. Subjects are aged 18 years or older with untreated early syphilis (primary, secondary, or early latent as defined in prior syphilis treatment trials of the Sexually Transmitted Infections Clinical Trials Group (STI CTG)). The study is conducted at 10 sites in the US and will enroll approximately 560 subjects to achieve 420 evaluable.

The study involves a screening/enrollment visit and seven scheduled follow-up visits (Weeks 1 and 2, and Months 1, 3, 6, 9, and 12) over a 12-month period. At the enrollment visit, after providing informed consent, all subjects undergo a brief sexual and medical history and a directed physical examination; all women of childbearing potential have a urine or serum pregnancy test performed as part of qualification for study participation; all subjects undergo phlebotomy for serological testing for syphilis and HIV according to the study and clinic protocols. All subjects who have not been tested for chlamydia and gonorrhea since last sexual activity and all subjects who have been sexually active in the past 14 days have specimens collected for chlamydia and gonorrhea testing. Subjects have specimens collected for other STI testing as indicated by local standard of care and subject history. All eligible subjects are randomized to Arm 1 or Arm 2 and will receive an injected dose of BPG 2.4 MU.

Study personnel will attempt to contact subjects approximately 24-48 hours after Visit 1 to assess for symptoms of a Jarisch-Herxheimer reaction as described in protocol **Section 8.1**. At the second and third visits (Weeks 1 and 2 of follow-up), subjects randomized to Arm 2 will receive injected doses of BPG 2.4 MU.

At all follow-up visits, subjects have an interval history obtained, undergo a directed physical examination, and have repeat specimens collected for STI testing (based on the subject history and clinic protocols). At all follow-up visits, subjects undergo phlebotomy for serological testing for syphilis and serum storage. At the Month 3, Month 6, Month 9, and Month 12 follow-up visits, consenting HIV-uninfected subjects are tested for HIV infection using a 4th generation serological test for HIV. At Visit 1, study staff will swab consenting subjects' oral cavities and primary or secondary lesions (if lesions are present per protocol **Section 5.1**); see protocol **Section 8.2.2**.

Safety is measured by subject report and physical examination (including vital signs: temperature, heart rate, respiration rate, and blood pressure; and genital, rectal, oral, skin, and lymph node examinations). All AEs (including solicited reactogenicity AEs and other unsolicited AEs) are recorded through Month 1. Safety oversight is provided by a Data and Safety Monitoring Board (DSMB) and site Independent Safety Monitors (ISMs) as described in protocol **Section 9.6**.

While sites perform their own serological testing as needed for subject management, serological testing to determine study outcomes and serum banking is performed at the Central Laboratory (University of Alabama at Birmingham).

The duration of the study for each subject is 12 months. Enrollment is expected to be completed in 36 months. Table 1 presents the schematic of the study design and Table 2 presents the schedule of events.

## 4.2. Discussion of Study Design, Including the Choice of Control Groups

While penicillin is generally regarded as highly effective for syphilis treatment, whether or not currently recommended therapy is equally effective for persons with or without HIV infection remains unclear. In addition, recent appreciation of the fact that ~20% of persons treated for early syphilis are serofast has raised concerns regarding the significance of this observation[6]. Some clinicians believe that the serofast state signifies treatment failure, while others believe it represents failure to serologically respond to therapy in successfully treated patients. Each of these concerns may lead to inadequate therapy or over treatment in persons with early syphilis, as well as much confusion among clinicians about appropriate therapy. Hence, the longer duration of therapy (BPG 2.4 MU weekly for three successive weeks) was to be compared with a single dose regimen by the serological response to therapy among persons with or without HIV infection. Subjects are randomized at a 1:1 ratio to receive one of the two BPG treatments.

## 4.3. Selection of Study Population

Subjects eligible to enroll in this study must meet all inclusion criteria:

1. Subject is aged 18 years or older.
2. Subject has provided informed consent.
3. Subject has untreated primary*, secondary**, or early latent*** syphilis.

   *Primary syphilis is characterized by the presence of an ulcerative lesion at a potential site of inoculation (while classically solitary, shallow, painless and with an indurated, clean base, primary lesions may be multiple, may vary considerably in appearance, and/or may not be painless) or by darkfield, acceptable polymerase chain reaction (PCR), or direct fluorescence antibody-T. pallidum (DFA-TP) positive ulcers.

   **Secondary syphilis is characterized by classical palmar/plantar rash, condylomata lata, mucous patches, etc. or by darkfield, acceptable PCR, or DFA-TP positive lesions.

   ***Early latent syphilis is characterized by current reactive serologic tests for syphilis (STS) and a documented non-reactive STS, or documented sexual exposure to an individual known to have primary, secondary, or early latent syphilis diagnosed within the last 12 months.
4. Subject either has a newly reactive non-treponemal test (such as an RPR test) or a history of syphilis and a current increase in RPR titer of two or more dilutions (i.e., four-fold).
5. If subject is of childbearing potential, subject has a negative urine or serum pregnancy test.
6. Subject is willing to have an HIV test, participate in HIV counseling, and return to clinic for follow-up.
7. In the opinion of the investigator, subject is able and willing to comply with study procedures, including receipt of three BPG injected doses if randomized to Arm 2.
8. If female, subject must be of non-childbearing potential* or must be using an acceptable method of birth control** to avoid becoming pregnant.

   *Non-childbearing potential is defined as being post-menopausal for at least 1 year, status after bilateral tubal ligation, or status after bilateral oophorectomy, or status after hysterectomy.

**Subject must agree to avoid becoming pregnant by using one of the following acceptable methods of birth control for the entire duration of participation in the trial:

- Intrauterine contraceptive device; OR
- Oral contraceptives; OR
- Hormonal injections; OR
- Hormonal implants; OR
- Contraceptive patches; OR
- Monogamous relationship with vasectomized partner; OR
- Exclusively same-sex relationships; OR
- Use of condoms by the male partner; OR
- Abstinence

Subjects eligible to enroll in this study must not meet any exclusion criteria:

1. Subject previously enrolled in this trial.
2. Subject has latent syphilis of unknown duration, late latent syphilis, or evidence of neurosyphilis, including ocular syphilis.*

   *e.g., eye pain/redness, recent ocular change, and/or changes in visual acuity
3. Subject has a known or suspected allergy or hypersensitivity to penicillin or other beta-lactam antibiotics.
4. Subject has a known or suspected STI other than syphilis requiring treatment with a drug active against T. pallidum.
5. Subject has used antibiotics* active against T. pallidum in the preceding 30 days.

   *Note: the use of antimicrobials known to NOT be effective against T. pallidum (e.g., quinolones, sulfonamides, trimethoprim, metronidazole, spectinomycin) will be allowed.
6. Subject has suspected or known ongoing drug use that might interfere with study participation and follow-up treatment.
7. Subject is breastfeeding.
8. Subject has used any investigational drugs in the past 30 days that might interfere with safety or efficacy assessment.*

   *If the subject has used any investigational drugs in the past 30 days, contact the Principal Investigator, DMID Clinical Project Manager, DMID Medical Officer, and FHI 360 to confirm eligibility.
9. Subject has any other condition that, in the opinion of the investigator, would interfere with participation in the study.

## 4.4. Treatments

### 4.4.1. Treatments Administered

BPG is administered as a deep IM injection in the upper, outer quadrant of the buttock. Participants are randomized using a 1:1 ratio to receive one or the two BPG treatments:

- Arm 1: BPG 2.4 MU injected once as two 2-mL injections (one in each buttock)
- Arm 2: BPG 2.4 MU injected weekly as two 2-mL injections (one in each buttock) for three successive weeks

The dates of first treatment are presented for all subjects who received at least one dose of study product (i.e. the Safety Population, defined in Section 6.3.1) by site in Table 3 and treatment group in Table 4.

### 4.4.2. Identity of Investigational Product(s)

BPG contains penicillin G benzathine in aqueous suspension with sodium citrate buffer and, as weight/volume, approximately 0.5% lecithin, 0.6% carboxymethylcellulose, 0.6% povidone, 0.1% methylparaben, and 0.01% propylparaben. Bicillin® L-A suspension in the disposable-syringe formulation is viscous and opaque and is available in a 2-mL size containing 1.2 MU BPG per syringe. It is labeled for deep IM injection only, not for intravenous (IV) use.

### 4.4.3. Method of Assigning Subjects to Treatment Groups (Randomization)

Enrollment of subjects is done online using the enrollment module of Emmes Advantage eClinical®. Subjects are randomized at a 1:1 ratio to receive one of the two BPG treatments after informed consent is obtained and their eligibility is confirmed.

The study uses a stratified, permuted block-randomization scheme. Permuted block randomization is used to avoid the potential for serious imbalance in the number of subjects assigned to each group, an imbalance that can occur in the simple randomization procedures. Stratification is by study site. While HIV status may not be known at the time of randomization, subjects are classified as HIV-infected or HIV-uninfected to assess the secondary HIV status subgroup objective, and randomization is limited as follows:

- If 280 subjects who were HIV-infected at baseline are confirmed evaluable, additional subjects who are known to be HIV-infected are not allowed to enroll thereafter, while HIV-uninfected subjects and subjects of unknown HIV status may continue to enroll.
- If 140 subjects who were HIV-uninfected at baseline are confirmed evaluable, only subjects who are known to be HIV-infected are allowed to enroll thereafter.

The list of randomized treatment assignments is prepared by statisticians at Emmes and included in the enrollment module of its Internet Data Entry System (IDES). IDES assigns each subject a treatment code from the list after demographic and eligibility data have been entered.

Instructions for use of the enrollment module are included in the IDES User's Guide. Manual back-up randomization procedures are provided in the Manual of Procedures (MOP) for use in case a site temporarily loses access to the Internet, or the online enrollment system is unavailable.

### 4.4.4. Selection of Doses in the Study

Subjects will be randomized to one of two treatment assignments:

- Arm 1: BPG 2.4 MU injected once as two 2-mL injections (one in each buttock)
- Arm 2: BPG 2.4 MU injected weekly as two 2-mL injections (one in each buttock) for three successive weeks

### 4.4.5. Selection and Timing of Dose for Each Subject

At the time of enrollment, subjects are randomized to receive either one dose of BPG (Arm 1) or three doses of BPG over 3 weeks (Arm 2). All eligible subjects will receive a dose of BPG on day 1. Subjects enrolled in Arm 2 will also receive a dose of BPG at Visit 2 (Week 1) and a dose of BPG at Visit 3 (Week 2). Subjects in Arm 2 should receive the three doses at a minimum of 7-day intervals per the BPG package insert.

The following describes the dose assigned for each treatment group:

| Study Groups | Visit 1, Day 1 | Visit 2, Week 1 (Day 7 to 13) | Visit 3, Week 2 (6 to 12 days after Visit 2) |
|---|---|---|---|
| Arm 1 | BPG 2.4 MU injected as two IM 2-mL injections | None | None |
| Arm 2 | BPG 2.4 MU injected as two IM 2-mL injections | BPG 2.4 MU injected as two IM 2-mL injections | BPG 2.4 MU injected as two IM 2-mL injections |

### 4.4.6. Blinding

Neither subjects nor providers will be masked in this study. The outcome (serological response to therapy) is objective—furthermore, laboratory staff will be blinded to subject ID and treatment assignment—and it would not be ethical to give subjects placebo injections.

### 4.4.7. Prior and Concomitant Therapy

Administration of any medications, therapies, or vaccines will be recorded on the appropriate data collection form. Concomitant medications will include all medications taken 30 days before initiating study treatment through Month 12 or early termination, whichever occurs first. Prescription and over-the-counter drugs will be included, as well as herbs, vitamins, and supplements. Previously recorded medications will be updated as appropriate.

Subjects who have received BPG and are subsequently diagnosed with a concomitant infection that requires systemic antibiotics will receive treatment according to the local clinic's standard protocols. Likewise, subjects who have received BPG through the study and subsequently require retreatment for syphilis infection will receive treatment according to the local clinic's standard protocols and will be withdrawn from the study per protocol **Section 5.3.3**.

At the discretion of the site PI, use of new medication should prompt evaluation for the presence of a new diagnosis of chronic medical disease or condition.

Medications that might interfere with the evaluation of BPG should not be used unless absolutely necessary.

Refer to protocol **Section 5** (Subject Inclusion and Exclusion Criteria) for medications that are prohibited for study eligibility and throughout study participation.

## 4.5. Efficacy (Immunogenicity) and Safety Variables

For safety and efficacy analyses, multiple observations within specific visit period are accepted. In the case of multiple observations within a specific window, the assessment value that is closest to the scheduled visit window will be used in the analyses for the post-baseline records, except the RPR data collected from early termination at an unscheduled visit could be used for serological response determination in addition to the data from the scheduled visits. For screening and baseline visits, the last assessment value will be used. All

the recorded data will be listed. If observations have the same distance to the scheduled assessment, the latest one will be used.

**Efficacy Variables**

See Section 3.3 for efficacy variable definitions. Serological response is the primary efficacy variable, which is defined in Section 3.3.1; The treatment schedule adherence is defined in Section 3.3.2.

**Safety Variables**

Safety will be monitored throughout the study by physical examination (including vital signs: temperature, heart rate, respiration rate, and blood pressure; and genital, rectal, oral, skin, and lymph node examinations) and subject reporting. Safety will be assessed by the frequency and severity of AEs/SAEs (including solicited reactogenicity AEs and other unsolicited AEs) occurring from the time of study product administration through the Month 1 visit.

**Adverse Event**

ICH E6 GCP defines an AE as any untoward medical occurrence in a clinical research subject administered a study drug regardless of its causal relationship to the study drug. An AE can therefore be any unfavorable and unintended sign (including an abnormal laboratory finding), symptom, or disease temporally associated with the use of the study drug. The occurrence of an AE may come to the attention of study personnel during study visits and interviews of a study subject presenting for medical care or upon review by a study monitor. Since the safety profile of BPG (both 1 and 3 doses) is well established, and this study is not powered to detect new, unknown safety signals. Non-serious AEs will not be reported to the DSMB.

All AEs not meeting the criteria for SAEs should be captured on the appropriate eCRF. Information to be collected includes event description, time of onset, clinician's assessment of severity, relationship to study drug (assessed only by those with the training and authority to make a diagnosis), and time of resolution/stabilization of the event. All AEs occurring while on study must be documented appropriately regardless of relationship. All AEs will be followed to adequate resolution. Any medical condition that is present at the time that the subject is screened should be considered as a baseline finding and not reported as an AE. However, if it deteriorates at any time during the study, it should be recorded as an AE.

All AEs must be graded for severity and relationship to the study drug. The US Food and Drug Administration (FDA) defines an AE as any untoward medical occurrence associated with the use of a drug in humans, whether or not considered drug-related.

**Severity of Event**: All AEs will be assessed by the clinician using a protocol-defined grading system (see protocol Appendix B). Changes in the severity of an AE should be documented to allow an assessment of its duration at each level of intensity to be performed. AEs characterized as intermittent require documentation of onset and duration of each episode.

**Relationship to Study Drug**: The clinician's assessment of an AE's relationship to the study drug is part of the documentation process, but it is not a factor in determining what is or is not reported in the study. If there is any doubt as to whether a clinical observation is an AE, it should be reported. All AEs must have their relationship to the study drug assessed using the terms "related" or "not related," as defined below. In a clinical trial, the study drug must always be suspect.

Related – There is a reasonable possibility that the study drug caused the AE. Reasonable possibility means that there is evidence to suggest a causal relationship between the study drug and the AE.

Not Related – There is not a reasonable possibility that administration of the study drug caused the AE. See Section 3.3 for efficacy variable definitions.

**Reactogenicity**

Reactogenicity events are AEs that are common and known to occur for the study drug being studied. For this protocol, the following reactogenicity events are expected to occur among some subjects:

Systemic reactions: Jarisch-Herxheimer reaction as defined in Section 3.3.3 and in protocol Section 8.1

Local injection site reactions: pain or tenderness, erythema or redness, induration or swelling

Reactogenicity events (as solicited AEs), along with unsolicited AEs, will be assessed at the visits specified in protocol Section 7 and will be graded using a protocol-defined grading system (see protocol Appendix B).

**Serious Adverse Event (SAE)**

An AE or suspected adverse reaction is considered "serious" if, in the view of either the investigator or sponsor, it results in any of the following outcomes:

- death,
- a life-threatening AE*,
- inpatient hospitalization or prolongation of existing hospitalization,
- a persistent or significant incapacity or substantial disruption of the ability to conduct normal life functions, or
- a congenital anomaly/birth defect.
- Important medical events that may not result in death, be life-threatening, or require hospitalizations may be considered serious when, based upon appropriate medical judgment, they may jeopardize the subject and may require medical or surgical intervention to prevent one of the outcomes listed in this definition. Examples of such medical events include allergic bronchospasm requiring intensive treatment in an emergency room or at home, blood dyscrasias or convulsions that do not result in inpatient hospitalization, or the development of drug dependency or drug abuse.

* An AE is considered "life-threatening" if, in the view of either the investigator or sponsor, its occurrence places the subject at immediate risk of death. It does not include an AE that, had it occurred in a more severe form, might have caused death.

All SAEs will be

- Assessed for severity and relationship to the study drug and alternate etiology (if not related to the study drug) by a licensed study physician.
- Recorded on the appropriate SAE form and eCRF.
- Followed through resolution by a licensed study physician.
- Reviewed and evaluated by the DSMB (periodic review unless related), DMID, and the IRB.

# 5. SAMPLE SIZE CONSIDERATIONS

## 5.1. Study with Planned Sample Size

Sample size calculations were performed using PASS 2008 and SAS 9.4. The following parameters were used for determining the required sample size for the primary objective:

- 1:1 allocation ratio between one-dose and three-dose arms
- Test the difference in response rate between the one-dose and three-dose arms (Farrington-Manning test, unadjusted for HIV status)
- Null hypothesis response rate in one-dose arm: 0.688
- Null hypothesis response rate in three-dose arm: 0.788
- Alternative hypothesis response rate in both arms: 0.788
- One-sided 0.05 alpha level

To achieve 80% power for the primary noninferiority comparison, 420 evaluable subjects (210 in each treatment arm) are needed. It is assumed that 25% of subjects will be ineligible for the primary analysis, and so 560 enrolled subjects are needed to reach the target of 420 evaluable subjects.

The study is not powered to assess the secondary noninferiority comparisons in the HIV status subgroups. As an illustration, power calculations were performed to assess the power available for these subgroup analyses using a sample size of 420. The following parameters were used for the calculations:

- Test the difference in response rate between the one-dose and three-dose arms within each HIV status stratum.
- Null hypothesis response rate in one-dose arm: 0.738 (HIV-infected); 0.638 (HIV-uninfected)
- Null hypothesis response rate in three-dose arm: 0.838 (HIV-infected); 0.738 (HIV-uninfected)
- Alternative hypothesis response rate in both arms: 0.838 (HIV-infected); 0.738 (HIV-uninfected)
- One-sided 0.05 alpha level
- 280 evaluable subjects in the HIV-infected stratum (140 in one-dose arm, 140 in three-dose arm).
- 140 evaluable subjects in the HIV-uninfected stratum (70 in one-dose arm, 70 in three-dose arm).

The HIV-infected noninferiority comparison has 72% power, and the HIV-uninfected noninferiority comparison has 38% power.

## 5.2. Study with Smaller Sample Size

When sample size is small, the statistical power will decrease. A simulation study was performed to explore the proposed method for the analysis across a small sample sizes. In particular, data was simulated under the following three conditions:

- Outcome data in both the 1-dose and 3-dose arms sampled from a binomial distribution with rate = 0.788.
- Outcome data in the 1-dose arm sampled from a binomial distribution with rate = 0.738, while the 3-dose arm data was sampled from a distribution with rate = 0.788.


PRIVILEGED AND CONFIDENTIAL COMMUNICATION

- Outcome data in the 1-dose arm sampled from a binomial distribution with rate = 0.688, while the 3-dose arm data was sampled from a distribution with rate = 0.788.
- Outcome data in the 1-dose arm sampled from a binomial distribution with rate = 0.588, while the 3-dose arm data was sampled from a distribution with rate = 0.788.

For each of the above scenarios, we simulated performing the analysis at the 40% and 50% time point (i.e. once we achieve 40% or 50% of the evaluable target). There are multiple ways to construct the futility bounds; two alpha-spending methods were explored here: O'Brien-Fleming Analog (OBF), and alpha-time (α-Time).

The table below provides three summaries from the simulation study:

- The probability of meeting the futility criterion at the analysis with smaller sample size (i.e. the probability of demonstrating 3-dose is superior to 1-dose).
- The probability of making a non-inferiority (NI) claim at the analysis with target sample size.
- The mean and standard deviation of the conditional power (CP) estimates across all simulated scenarios.

| Scenario | Percent of Target Sample size | Futility Bound Method | Prob. of Meeting Futility Criterion (%) | Prob. of NI Claim (%) | Mean (SD) CP at Interim (%) |
|---|---|---|---|---|---|
| $P_1 = 0.788$<br>$P_3 = 0.788$ | 40% | OBF | <1 | 80 | 80 (19) |
| | | α-Time | 1 | 78 | 78 (20) |
| | 50% | OBF | <1 | 79 | 79 (22) |
| | | α-Time | 1 | 77 | 77 (23) |
| $P_1 = 0.738$<br>$P_3 = 0.788$ | 40% | OBF | 2 | 32 | 63 (25) |
| | | α-Time | 5 | 30 | 60 (25) |
| | 50% | OBF | 4 | 33 | 57 (29) |
| | | α-Time | 8 | 28 | 54 (29) |
| $P_1 = 0.688$<br>$P_3 = 0.788$ | 40% | OBF | 8 | 5.0 | 44 (27) |
| | | α-Time | 18 | 4.5 | 42 (26) |
| | 50% | OBF | 18 | 4.4 | 33 (28) |
| | | α-Time | 27 | 4.2 | 31 (27) |
| $P_1 = 0.588$<br>$P_3 = 0.788$ | 40% | OBF | 48 | <1 | 13 (16) |
| | | α-Time | 66 | <1 | 12 (16) |
| | 50% | OBF | 73 | <1 | 5 (11) |
| | | α-Time | 82 | <1 | 5 (10) |

The potential outcomes from the above scenarios are: When null hypothesis response rate in one-dose arm is 0.688 and null hypothesis response rate in three-dose arm: 0.788, as indicated in Section 5.1, the probability of claim non-inferiority is less than 5%, and the statistical power is less than 45%. The issue is less that the

non-inferiority design is causing issues but more so that the analysis with small sample size is an under-powered.

# 6. GENERAL STATISTICAL CONSIDERATIONS

## 6.1. General Principles

Most of the continuous variables will be summarized using the following descriptive statistics: N (non-missing sample size), mean, standard deviation, median, maximum and minimum. RPR titer will be summarized using N (non-missing sample size), Geometric Mean Titer (GMT), and 95% confidence interval. The frequency and percentages (based on the non-missing sample size) of observed levels will be reported for all categorical measures. Wilson confidence intervals for binomial proportions and differences in binomial proportions will be computed for efficacy variables and safety variables. For the hypothesis tests comparing treatment groups with respect to the compliance endpoint, the two-sided Pearson Chi-Square test with the Yates continuity correction will be used. For the above tests, a 5% two-sided significance level will be used. For the hypothesis tests comparing treatment groups with respect to efficacy outcomes, the Farrington-Manning test at the 5% one-sided level of significance will be used. A noninferiority hypothesis test will be conducted using the Farrington-Manning test at the 5% one-sided level of significance to formally compare the treatment arms, that is, whether the one-dose group is non-inferior to the three-dose group.

For all efficacy outcome measures, the Intent-to-Treat (ITT) population (see Section 6.3) will be used as the primary analysis population and the primary analysis will be repeated as a secondary analysis in the mITT and the Evaluable analysis populations. The secondary efficacy analyses will be performed in the ITT and Evaluable analysis populations. For all safety analyses, the Safety population will be used as the analysis population.

In general, all data will be listed, sorted by site, treatment and subject, and when appropriate by visit number within subject. All summary tables will be structured with a column/sub-table for each treatment group in the order (One dose BPG 2.4 MU, Three doses BPG 2.4 MU, and All Subjects) and will be annotated with the total population size relevant to that table/treatment, including any missing observations.

Note that in the data listings, Subject ID is the unique subject identifier, not the Study ID used on study and dates will not be included, only Study Day. Study Day 1 will be the day of the first dose of treatment.

### 6.1.1. Pseudo Code

The following SAS® pseudo code will be used to calculate the following:

Farrington-Manning test at 5% one-sided significance level and risk difference (and 95% asymptotic CI) from 2x2 table:

```
proc freq;
      table treatment*analysis_variable /riskdiff (noninf margin=.1 method=fm);
      ods output PdiffNoninf=outdn;
run;
```

Cochran-Mantel-Haenszel Chi-square test and Breslow-Day test for h x 2 x 2 table:

```
proc freq;
      Table strata*treatment*analysis_variable / relrisk plots(only)=relriskplot(stats) CMH;
      ods output CMH=outputdsn1;
      ods output BreslowDayTest=outputdsn2;
run;
```

Chi-Square test at 5% two-sided significance level and odds ratio (and 95% asymptotic CI) from 2x2 table:

```
proc freq;
      Table treatment*analysis_variable / chisq;
      ods output ChiSq=outputdsn1;
      ods output RelativeRisks=outputdsn2;
run;
```

95% Wilson CI for proportions/percentages:

```
proc freq;
      Table treatment*analysisvariable / binomial(wilson);
      ods output binomialcls=outputdsn;
run;
```

95% Wilson CI for difference in proportions (produces Newcombe CI):

```
proc freq;
      Table treatment*analysisvariable / riskdiff(cl=Wilson);
      Exact Riskdiff;
      ods output pdiffcls=outputdsn;
run;
```

t-test for independent samples

```
proc ttest sides=2 alpha=0.05;
      class HIV_status;
      var analysis_variable;
run;
```

Logistic Regression

```
proc logistic;
      class var_1 var_2 var_3/ param=glm;
      model y = var_1 var_2 var_3;
run;
```

## 6.2. Timing of Analyses

There is no planned interim analysis of efficacy data. The final analysis for primary, secondary efficacy outcomes by Month 6 and safety data will be performed after an interim database lock when all subjects have been followed through Visit 6, at month 6 (Window: 180±21 days). The final analysis for all other outcomes will be performed after the final database lock when all subjects have been followed through Visit 8, the final study visit, at Month 12 (Window: 360±28 days).

## 6.3. Analysis Populations

### 6.3.1. Safety Analyses

All safety analyses will be performed in the safety analysis population. The safety population includes all randomized subjects who received at least one dose of study product.


PRIVILEGED AND CONFIDENTIAL COMMUNICATION

### 6.3.2. Efficacy Analyses

All efficacy analyses will be performed in the Intent-to-Treat population and Evaluable population. The primary analysis will be performed in the ITT population and repeated as a secondary analysis in the mITT and the Evaluable analysis populations. The secondary efficacy analyses will be performed in the ITT and Evaluable populations.

#### 6.3.2.1. Intent-to-Treat Population

The ITT population includes all randomized subjects, regardless of whether they received study treatment or were compliant with the administration procedures or schedule. In the unlikely event of an error in randomization or study product administration (such as, incorrect dosing schedule), subjects will be grouped by their intended randomized assignment.

#### 6.3.2.2. Modified Intent-to-Treat (mITT) Population

The mITT population includes all randomized subjects who are eligible at the baseline visit and whose TPPA (Treponema pallidum Particle Agglutination) result is positive at the baseline visit and/or at Visit 5 (Month 3), if repeat testing was performed.

#### 6.3.2.3. Evaluable Population

Two Evaluable populations (by Month 6 and by Month 12) are of interest per the primary and secondary study endpoints.

Both Evaluable populations include all randomized subjects who are eligible at the baseline visit, have a known HIV status determined at or before the baseline visit, have positive TPPA results at the baseline visit and/or Visit 5 (Month 3) (if repeat testing was performed), received all assigned doses of BPG, have RPR titer data available at baseline and applicable follow-up visit(s) (have RPR test data available at baseline and at least one follow-up visit at or before Month 6 visit for the Evaluable population by Month 6; have RPR titer data available at baseline and at least one follow-up visits between month 6 and Month 12 visit and (for HIV-uninfected subjects only) HIV-uninfected status persists through applicable follow-up visit(s). According to CDC guideline, an interval of 10-14 days between injections is acceptable, so subjects who receive all doses within this interval will be considered as 'received all assigned doses of BPG'.

If a subject receives antibiotic(s) active against T. pallidum for reasons other than syphilis between enrollment and the applicable follow-up visit(s), following situations will be considered:

a. if a subject has valid baseline and at least one follow-up RPR value before receiving antibiotic(s) active against T. pallidum for reasons other than syphilis, those RPR values (or seronegative) prior to receiving antibiotic(s) will be kept and used to evaluate the efficacy outcome, and the subject will be included in the evaluable population.
b. if a subject has valid baseline but no follow-up RPR value before (s)he receives antibiotic(s) active against T. pallidum for reasons other than syphilis, the subject will be excluded from evaluable population.
c. Receipt of antibiotic(s) active against T. pallidum for reasons other than syphilis will not exclude subjects or their data post-antibiotic from the ITT or mITT populations.

If a subject receives antibiotic(s) active against T. pallidum between enrollment and the applicable follow-up visit(s) for retreatment of syphilis, the subject may be included in all analyses if they are otherwise eligible for


PRIVILEGED AND CONFIDENTIAL COMMUNICATION

the analysis. Those RPR values (or seronegative) prior to and/or on the data of receiving retreatment will be kept and used to evaluate the efficacy outcome, those RPR values after receiving retreatment will not be used for analysis, and the subject will be included in both ITT and evaluable population.

A blinded case review committee will review subjects' data to determine if treatment with drug(s) known to be active against T. pallidum for reasons other than syphilis infection excludes the subject from the Evaluable analysis population. A separated listing of subjects, their concurrent medications, and their sexual history data will be provided to the committee for review.

In the unlikely event of an error in randomization or study product administration (i.e., incorrect dosing schedule), subjects will be grouped by the product they actual received.

#### 6.3.2.4. Analysis Population Summaries

Table 5 summarizes the ITT and mITT population eligibilities by randomized treatment group and reasons for exclusion. Table 6 summarizes the Safety and Evaluable population eligibilities by actual treatment group and reasons for exclusion. Subjects will be included in the count for a particular reason for exclusion if they met that criterion. As subjects may meet more than one criterion for exclusion, the "Excluded from…" counts may be less than the sum of the individual reason counts. A listing of the subjects excluded from each of the analysis populations and the reasons for exclusion will be provided (Listing 1).

## 6.4. Covariates and Subgroups

The protocol defines formal subgroup analyses. Subgroups are defined by the baseline HIV status (HIV-infected and HIV-uninfected). Demographics, baseline characteristics and sexual history will be reported by baseline HIV status. The point estimates of the treatment-specific proportions within each HIV status subgroup, difference in proportions and corresponding 95% confidence intervals will be reported. For each HIV status subgroup, a hypothesis test will be conducted to compare the treatment arms with respect to response rate across HIV status subgroups. The homogeneity of odds ratios will be tested to determine if the difference between the treatment groups with respect to response rates varies by HIV status. Logistic regression analyses will be used to assess the association of treatment with Jarisch-Herxheimer reaction after adjusting for HIV co-infection status and time point post-dose.

## 6.5. Missing Data

For the primary analysis, serological response is defined by 4-fold or more decline compared to baseline or seronegative any visit prior to the end of Month 6 visit window. Though the ITT and mITT populations include subjects who may not have adequate serological data to determine their response status for any reason, the above definition is equivalent to the Best Case Imputation, which means the lowest RPR value or seronegative from the available visits will be used impute the missing values and to evaluate the serological response. If there were no follow-up RPR values by the end of month 6 visit window, the missing value will be imputed by the baseline value, which is also the best case in the situation.

As one of the two sensitivity analyses, the primary outcome will be defined based on the last available visit by month 6. The Last Observation Carried Forward (LOCF) will be used to impute the missing visit. When a subject did not have any follow-up RPR values from the collected data by month 6, we will use the last available visit before the end of month 6 visit window to impute the serological response.

If a subject missed visits, discontinued treatment or early terminated due to Covid-19, the subject will be recorded and reported. Also, another sensitivity analysis will be performed to evaluate the impact by Covid-19 by removing those subjects who discontinued treatment or early terminated.

## 6.6. Interim Analyses and Data Monitoring

There are no planned interim analyses of efficacy data. However, when all enrolled subjects have completed the Month 6 visit (or are past the visit window), the efficacy data through Month 6 and all available safety data will be locked and all planned analyses for this data will be performed. If a table or figure includes summary for both month 6 and post month 6 data, then the table or figure will be generated for locked data at month 6 and will then be updated once the final data is available.

The remainder of the data through Month 12 will be analyzed once all the study data is entered, cleaned, monitored and locked. Individual subject listings, which reveal the individual treatment assignments, will only be generated at the final analysis after all Month 12 data have been locked.

The study is monitored to determine if any of the safety halting rules are met. The halting rules are:

- If one or more subjects experience an SAE judged by an investigator to be related to the study drug
- An overall pattern of clinical events that the DMID Medical Monitor or DSMB consider associated with the study drug and that may appear minor in terms of individual events, but that may collectively represent a serious potential concern for safety

If any of the halting rules are met, the study will not continue with the remaining enrollments or study treatments without a review by and recommendation from the DSMB to proceed. A summary of halting rules is provided in Table 7**.** DMID retains the authority to suspend additional enrollment and administration of the study drug during the entire study, as applicable.

An ISM at each clinical research site oversees the safety of research subjects at that site and provides independent written evaluation of SAEs and related Grade 3 AEs to the PI, DMID Clinical Project Manager, and DMID Medical Monitor. The ISM will serve as an independent consultant for the site PI on subject-related issues. The ISM will communicate with the site PI and study PIs to resolve any issues.

Safety oversight is conducted by a DSMB that is an independent group of experts that monitors subject safety and advises DMID. The DSMB members are separate and independent of study personnel participating in this trial and should not have scientific, financial, or other conflict of interest related to the study. The DSMB consists of members with appropriate expertise to contribute to the interpretation of the data from this trial.

The DSMB reviews safety and enrollment data when half of the study subjects have been enrolled and observed for SAEs through the Month 1 Visit.

The DSMB reviews study progress and subject clinical and safety data at the following time points:

- At least annually after the first subject is enrolled.
- Ad hoc when a halting rule is met, for immediate concerns regarding observations during the study, or as needed.
- During a final closeout meeting, held at the end of the study after the database is locked to review cumulative study data prior to finalizing the Clinical Study Report (CSR).

The DSMB operates under the rules of a DMID-approved charter that are written at the organizational meeting of the DSMB. As an outcome of each review/meeting, the DSMB makes a recommendation as to the

advisability of proceeding with study administrations (as applicable), and to continue, modify, or terminate the study.

DMID or the DSMB chair may convene the DSMB on an ad hoc basis according to protocol criteria or if there are immediate concerns regarding observations during the study. The DMID Medical Monitor is empowered to stop enrollment and study treatment if SAEs that meet the halting criteria are reported. The DMID Medical Monitor is responsible for reviewing SAEs in real time. The DSMB reviews SAEs on a regular basis and ad hoc during the study.

## 6.7. Multicenter Studies

Safety and efficacy data will be pooled across all clinical sites. Center effects are not anticipated because the sites are using standardized procedures for administration of study product and assessment of solicited and unsolicited adverse events, and the study relies on the central laboratory for the assessment of serological data and clinical efficacy endpoints.

## 6.8. Multiple Comparisons/Multiplicity

No adjustments for multiplicity are planned.

# 7. STUDY SUBJECTS

## 7.1. Disposition of Subjects

The disposition of subjects in the study will be tabulated by randomized treatment group and all subjects. Table 8 will present a summary of the reasons that subjects were screened but not enrolled. Table 9 will show the number of subjects enrolled, randomized, treated, complied with treatment, completed Month 6 RPR blood draw, completed Month 12 RPR blood draw, and completed the study. A listing of subjects who terminated early from study or discontinued treatment and the reason for early termination or treatment discontinuation will be included in Listing 2. The subjects who terminated early or discontinued treatment due to Covid-19 pandemic will be flagged in the listing.

A flowchart showing the disposition of study subjects, adapted from the CONSORT statement will be included (Figure 1). This figure will present the number of subjects screened, enrolled, lost to follow-up, and analyzed, by treatment arm.

## 7.2. Protocol Deviations

A summary of subject-specific protocol deviations will be presented by the deviation category, the type of deviation, and randomized treatment group for all enrolled subjects (Table 10). All subject-specific protocol deviations and non-subject-specific protocol deviations will be included in Appendix 3 as data listings (Listing 3 and Listing 4, respectively).


PRIVILEGED AND CONFIDENTIAL COMMUNICATION

# 8. EFFICACY EVALUATION

## 8.1. Primary Efficacy Analysis

For the primary analysis of the comparison of efficacy of one dose versus three doses of BPG 2.4 MU, the number and proportion of subjects with serological response and 95% confidence interval will be summarized overall and by treatment. The point estimates for the treatment-specific proportion with a one-sided 95% confidence interval (CI) will be reported. The difference in proportions and corresponding one-sided 95% CI will also be reported. Serological response for the primary analysis is defined in Section 3.3.1**.** The primary analysis will be performed in the ITT population. The primary analysis will be repeated as a secondary analysis in the mITT and Evaluable populations.

The null hypothesis for the primary objective is that the difference in serological response rate between the three-dose and one-dose groups is at least 10%, and the alternative hypothesis is that the difference in response rates is less than 10%.

$$H_0: P_3 - P_1 \geq 0.10$$
$$H_1: P_3 - P_1 < 0.10$$

The null and alternative hypotheses for the secondary HIV status subgroup objective are defined similarly. For the hypothesis tests comparing treatment groups with respect to efficacy outcomes, the Farrington-Manning test at the 5% one-sided level of significance will be used.

Table 11 will summarize the primary outcome for ITT, mITT and Evaluable populations. Figure 2**,** Figure 3, and Figure 4 will present the individual efficacy response data at each visit for ITT, mITT and Evaluable populations by treatment group by Month 6. Figure 11 will present the Non inferiority test result for the primary outcome between two treatment groups by Month 6. Listing 5 will present the individual efficacy response data for all randomized subjects. Individual subject listings of TPPA test results (Listing 24) will be provided.

### 8.1.1. Sensitivity Analyses for Primary Efficacy Outcome

One sensitivity analysis is do the same analysis with primary outcome except removing subjects who early terminated from study due to Covid-19 pandemic. The second sensitivity analysis of the Primary outcome will be performed with a different definition of the outcome, the last available value prior to Month 6 for each subject. The outcome is defined in Section 6.5. Table 31 and Table 32 will present sensitivity analyses, the Serological response to Therapy at Month 6 by treatment Group. The analyses will be performed in the ITT, mITT, and Evaluable populations.

## 8.2. Secondary Efficacy Analyses

The secondary efficacy analyses will be performed in the ITT and Evaluable populations.

### 8.2.1. Efficacy by 12 Months

Summaries of the number and proportion of subjects with serological response and difference in proportion with 95% Wilson confidence intervals by Month 12 by treatment group in the ITT population and the Evaluable population by Month 12 will be presented in Table 12. A hypothesis test will be conducted using the Farrington-Manning test at the 5% one-sided level of significance to formally compare the treatment arms. The setup of the noninferiority test will be analogous to that of the primary analysis. Figure 5, and Figure 6 will present the individual efficacy response data at each visit for ITT, and Evaluable populations by

treatment group by Month 12. Figure 12 will present the Non inferiority test result for the primary outcome between two treatment groups by Month 12.

### 8.2.2. Efficacy by 6 Months Among Subjects with and without HIV Infection

The response rate of the two treatment arms across HIV status subgroups (HIV-infected, HIV-uninfected) will be summarized in Table 13 for the ITT and Evaluable populations. The Breslow-Day test for the homogeneity of odds ratios and Cochran-Mantel-Haenszel Chi-square test will be used to determine if the difference between the two treatment groups with respect to response rates varies by HIV status.

Table 14 will present the summaries of the number and proportion of subjects with serological response and by each timepoint by treatment group in the ITT population.

Table 15 will present the summaries of the number and proportion of subjects with serological response and difference in proportions with 95% Wilson confidence intervals by Month 6 by treatment group and baseline HIV status in the ITT population and the Evaluable population by Month 6, respectively. For each HIV status subgroup, a hypothesis test will be conducted using the Farrington-Manning test at the 5% one-sided level of significance to formally compare the treatment arms. The setup of the noninferiority test will be analogous to that of the primary analysis, performed within each subgroup.

Figure 7 and Figure 8 will present the individual efficacy response data at each visit for all randomized subjects by treatment group and baseline HIV Status by Month 6 in the ITT, and Evaluable populations.

Individual subject listings of HIV testing outcomes (Listing 23) will be provided.

### 8.2.3. Efficacy by 12 Months Among Subjects with and without HIV Infection

Table 16 will present the summaries of the number and proportion of subjects with serological response and difference in proportions with 95% Wilson confidence intervals by Month 12 by treatment group and baseline HIV status in the ITT population and the Evaluable population by Month 12, respectively. For each HIV status subgroup, a hypothesis test will be conducted using the Farrington-Manning test at the 5% one-sided level of significance to formally compare the treatment arms.

The geometric mean titers and geometric mean fold declines from baseline will be summarized by each visit for each treatment group, overall and within HIV status subgroups in the ITT and Evaluable populations (Table 17, Table 18, and Table 19). Figure 9 and Figure 10 will present the individual efficacy response data at each visit for all randomized subjects by treatment group and baseline HIV Status by Month 12 in the ITT, and Evaluable populations.

### 8.2.4. Treatment Compliance by Treatment Group

For the comparison of compliance between the one dose and three dose groups, the number and proportion of subjects who receive all assigned doses within the assigned visit windows will be summarized overall and by treatment group in ITT and Evaluable populations. The point estimates for the treatment-specific proportions and difference in proportions as well as corresponding 95% confidence intervals will be presented. A hypothesis test will be conducted using a Chi-square test at the 5% two-sided level of significance to formally compare the treatment arms (Table 20). Listing 6 will present the individual compliance data for all enrolled subjects.


PRIVILEGED AND CONFIDENTIAL COMMUNICATION

### 8.2.5. Demographics and Baseline Characteristics by HIV status

Categorical and continuous demographics, and baseline socio-epidemiologic characteristics will be summarized overall and by baseline HIV status subgroup in the ITT population (Table 21 and Table 22). Baseline sexual history will be summarized overall and by baseline HIV status subgroup in the ITT populations (Table 23**,** Table 24). Categorical and continuous sexual history questions through Month 6 and Month 12 will be summarized overall and by baseline HIV status subgroup in the ITT and corresponding Evaluable populations (Table 25**,** Table 26**,** Table 27**,** Table 28**,** Table 29**,** and Table 30**)**. For continuous variables, descriptive statistics will include the number of non-missing values, mean, standard deviation, median, minimum, and maximum. For categorical variables, descriptive statistics will include counts and percentages per category.

Individual subject listing will be presented for all demographics and other baseline socio-epidemiologic characteristics (Listing 7). Individual subject listing of baseline and follow-up sexual behavior history for all enrolled subjects will be presented in Listing 8 and Listing 9. Sexually Transmitted Disease history at baseline for all enrolled subjects will be presented in Listing 10. Sexually Transmitted Infections testing result will be present in Listing 11.

## 8.3. Exploratory Efficacy Analyses

The exploratory efficacy analysis will be performed to support the primary efficacy analysis by applying the alternative serological response classification stated in Section 3.3.1**.** The serological response will be classified into three levels which are Response, Non-Response and Failure. Similar summary as primary efficacy analysis will be presented in Table 33 for ITT, mITT and Evaluable populations.

## 8.4. Clinical Laboratory Evaluations

RPR test evaluations will be presented in Listing 5**.** Individual subject listings of HIV testing outcomes (Listing 23) and TPPA test results (Listing 24) will be provided. Descriptive statistics for CD4 (Table 49) and Viral Load (Table 50), including mean, standard deviation, median, minimum and maximum values by time point and actual treatment group will be summarized.

# 9. SAFETY EVALUATION

## 9.1. Prior Conditions

All current illnesses and past pre-existing medical conditions will be MedDRA® coded using MedDRA dictionary version 23.1 or higher. Concomitant medications will be coded to the Anatomical Therapeutic Classification using the WHO Drug Dictionary.

### 9.1.1. Prior and Concurrent Medical Conditions

Summaries of subjects' pre-existing and concurrent medical conditions will be presented by randomized treatment group for the Safety Population (Table 34). An individual subject listing for all enrolled subjects will be presented for all medical conditions (Listing 12).

### 9.1.2. Prior and Concomitant Medications

The use of prior and concomitant medications taken during the study will be recorded on the CRFs. Summaries of medications that were started prior to dosing and continuing at the time of dosing as well as medications that were started during dosing or during follow up will be presented by WHO Drug Anatomical Codes (ATC) Level 1 and Level 2 and actual treatment group for subjects in the Safety population (Table 35).

An individual subject listing for all enrolled subjects will be presented for all concomitant medications (Listing 13).

## 9.2. Measurements of Treatment Compliance

Subjects are randomized to receive either one dose of BPG (Arm 1) or three doses of BPG over 3 weeks (Arm 2). Subjects are compliant to the treatment if all doses per randomization are received, and the corresponding visit days are within the assigned visit window. See Section 3.3.2 for the definition of adherence to scheduled visits. See compliance tables for secondary efficacy analyses in Section 8.2.

The number of subjects not compliant with study treatment will be presented by treatment group as part of the subject disposition table (Table 9). An individual subject listing of treatment compliance for all enrolled subjects will be provided in Listing 6.

## 9.3. Adverse Events

When calculating the incidence of adverse events (i.e., on a per subject basis), each subject will only be counted once and any repetitions of adverse events within a subject will be ignored; the denominator will be the total safety population size and population within the actual treatment group. AEs/SAEs (including solicited reactogenicity AEs and other unsolicited AEs) occurring from the time of study product administration through the Month 1 visit are collected. All adverse events reported will be included in the summaries and analyses. All analyses in Section 9.3 will be performed in the safety analysis population using the actual treatment received. An overall summary of adverse events will be presented in Table 36.

### 9.3.1. Solicited Events and Symptoms

Reactogenicity events are AEs that are common and known to occur for the study drug being studied. For this study, the following reactogenicity events are expected to occur among some subjects:

- Systemic reactions: Jarisch-Herxheimer reaction (as described in protocol **Section 8.1**)
- Local injection site reactions: pain or tenderness, erythema or redness, induration or swelling

Reactogenicity events (as solicited AEs) will be assessed at the visits specified in protocol **Section 7** and will be graded on a scale of 1 (mild), 2 (moderate) and 3 (severe) by using a protocol-defined grading system (see protocol Appendix B). Reactogenicity events will be analyzed by taking the most severe response over the follow-up period, dichotomizing into a binary variable (none versus mild, moderate, or severe) and using exact confidence intervals to summarize the reactogenicity rates.

The number and percent of subjects reporting at least one solicited adverse event will be summarized for each solicited adverse event along with the 95% Wilson CI and presented in Table 37.

The number and percentage of subjects reporting each solicited systemic adverse event will be summarized by the maximum severity, and actual treatment group along with the 95% Wilson CIs (Table 38). The number and percentage of subjects reporting each solicited local adverse event will be summarized by the maximum severity, dose, and actual treatment group along with the 95% Wilson CIs (Table 39).

The percentage of subjects reporting each solicited systemic adverse event (i.e. Jarisch-Herxheimer reaction) by maximum severity per subject and Time Post First Dose will be presented in Figure 13**.** The percentage of subjects reporting each solicited systemic adverse event by maximum severity of solicited local symptoms per Subject and Dose will be presented in Figure 14.

Logistic regression analyses will be used to assess the association of treatment with Jarisch-Herxheimer reaction after adjusting for HIV co-infection status and onset time post-dose (Table 40). The binary outcome is whether the subject experienced any symptoms of the Jarisch-Herxheimer Reaction in the 24-hours following Visit 1. The onset time of Jarisch-Herxheimer Reaction is categorized into 0-12 hours, >12-24 hours, >24-48 hours. Crude and adjusted odds ratios and 95% Wald CIs will be presented.

Solicited systemic and local reactions by subject will be presented in Listing 14 and Listing 15 **.**

### 9.3.2. Unsolicited Adverse Events

The number of subjects, the proportion of subjects who experienced unsolicited AEs and SAEs following the first dose of the study product through Visit 4 (Month 1, Day 30 ± 7 days), and the 95% Wilson CIs for the proportion of subjects who experienced unsolicited AEs and SAEs related to study product through Visit 4 will be presented for the safety population and actual treatment group. Denominators for percentages are the number of subjects who received the study product being summarized.

Unsolicited adverse events by subject will be presented in Listing 16.

The following summaries for unsolicited adverse events will be presented by MedDRA® system organ class, preferred term, and treatment group:

- Subject incidence and total frequency of adverse events over time by dose with 95% CI (Table 41);
- Subject incidence and total frequency of related adverse events over time by dose with 95% CI (Table 42);
- Summary of severity and relationship to study product for the unsolicited adverse events (Table 43);

- Subject incidence and percentage by maximum severity and maximum relationship to study product for all subjects (Table 44);
- The number of adverse events occurring in ≥ 5% of subjects in any treatment group (Table 45, Table 46);
- Subject listing of serious adverse events (Table 47);
- Subject listing of non-serious adverse events of moderate or greater severity (Table 48);
- Bar chart of total frequency of adverse events by severity and MedDRA® system organ class (Figure 15);
- Bar chart of subject incidence of adverse events by severity and MedDRA® system organ class (Figure 16);
- Bar chart of total frequency of adverse events by relationship to study product and MedDRA® system organ class (Figure 17);
- Bar chart of subject incidence of adverse events by relationship to study product and MedDRA® system organ class (Figure 18);

## 9.4. Deaths, Serious Adverse Events and other Significant Adverse Events

A listing of deaths and serious adverse events will be presented including Subject ID, treatment group, Adverse Event Description, Dose Number Associated with, SAE onset day and duration post associated dose, Reason Reported as an SAE, Relationship to Treatment, Alternate Etiology if Not Related, Outcome, and Duration of Event in days (Table 47).

## 9.5. Pregnancies

For any subjects in the Safety population who became pregnant during the study, every attempt will be made to follow these subjects to completion of pregnancy to document the outcome, including information regarding any complications with pregnancy and/or delivery. Listings of pregnancies and outcomes, including the total pregnancies, number of live births, and number of spontaneous abortions, elective abortions or still births will be presented (Listing 17, Listing 18, Listing 19, Listing 20, and Listing 21).

## 9.6. Vital Signs and Physical Evaluations

At Visit 2 and Visit 3, subjects will be evaluated for resolution of signs of syphilis documented at Visit 1. An individual subject listing of syphilis signs by visits 1, 2 and 3 will be presented (Listing 22). A targeted physical examination (vital signs (temperature, heart rate, respiration rate, and blood pressure); genital, rectal, oral, skin, and lymph node examinations) will be performed at each visit. Each vital sign for subjects in the Safety Population will be summarized by visit, treatment group and grading of severity (Table 51, Table 52, Table 53, Table 54, and Table 55). Subject listings will be provided for HIV test result (Listing 23), TPPA test result (Listing 24), vital signs (Listing 25) and physical exam findings (Listing 26).

## 9.7. Other Safety Measures

N/A

# 10. PHARMACOKINETICS

N/A

# 11. IMMUNOGENICITY

N/A

# 12. OTHER ANALYSES

N/A

# 13. REPORTING CONVENTIONS

P-values ≥0.001 and ≤0.999 will be reported to three decimal places; p-values less than 0.001 will be reported as "<0.001"; p-values greater than 0.999 will be reported as "> 0.999". The mean, median, standard deviation, and any other statistics other than quantiles, will be reported to one decimal place greater than the original data. Quantiles other than the median will use the same number of decimal places as the original data. Proportions will be presented to two decimal places; values <0.01 will be presented as "<0.01". Percentages will be reported to the nearest whole number; values < 1% will be presented as "<1". Estimated parameters, not on the same scale as raw observations (e.g. regression coefficients) will be reported to three significant figures.


PRIVILEGED AND CONFIDENTIAL COMMUNICATION

# 14. TECHNICAL DETAILS

SAS version 9.4 or above will be used to generate all tables, figures and listings.

# 15. SUMMARY OF CHANGES IN THE CONDUCT OF THE STUDY OR PLANNED ANALYSES

There are no changes in the conduct of the study or planned analyses.

# 16. REFERENCES

1. Drummond R. CONSORT Revised: Improving the Reporting of Randomized Clinical Trials. JAMA. 2001; 285(15):2006-2007.
2. Hook, E.W., *Syphilis.* Lancet, 2017. **389**(10078): p. 1550-1557.
3. *CDC Sexually transmitted disease surveillance 2016*. 2017, U.S. Department of Health and Human Services: Atlanta.
4. *CDC Sexually transmitted disease surveillance 2015*. 2016, U.S. Department of Health and Human Services: Atlanta.
5. *CDC Sexually transmitted disease surveillance 2012*. 2013, U.S. Department of Health and Human Services: Atlanta.
6. Workowski, K.A. and G.A. Bolan, *Sexually transmitted diseases treatment guidelines, 2015.* MMWR Recomm Rep, 2015. **64**(Rr-03): p. 1-137.
7. Sena, A.C., et al., *Predictors of serological cure and serofast state after treatment in HIV-negative persons with early syphilis.* Clin Infect Dis, 2011. **53**(11): p. 1092-9.
8. Rolfs, R.T., et al., *A randomized trial of enhanced therapy for early syphilis in patients with and without human immunodeficiency virus infection. The Syphilis and HIV Study Group.* N Engl J Med, 1997. **337**(5): p. 307-14.


PRIVILEGED AND CONFIDENTIAL COMMUNICATION

# 17. LISTING OF TABLES, FIGURES, AND LISTINGS

Table, figure, and listing shells are presented in Appendices 1, 2, and 3.


PRIVILEGED AND CONFIDENTIAL COMMUNICATION

# APPENDICES

# APPENDIX 1. TABLE MOCK-UPS

## LIST OF TABLES

Table 1: Study Design....43
Table 2: Schedule of Study Procedures ....44
Table 3: Dates of First Treatment by Site – Safety Population ....46
Table 4: Dates of First Treatment by Treatment Group - Safety Population....47
Table 5: ITT and mITT Analysis Population Eligibilities by Treatment Group - All Enrolled subjects....48
Table 6: Safety and Evaluable Population Eligibilities by Treatment Group....49
Table 7: Summary of Halting Rules - Safety Population ....51
Table 8: Summary of Screen Failures....52
Table 9: Subject Disposition by Treatment Group- All Enrolled Subjects ....53
Table 10: Distribution of Protocol Deviations by Category, Type, and Treatment Group – All Enrolled subjects ....54
Table 11: Serological Response to Therapy by Month 6 by Treatment Group ....55
Table 12: Serological Response to Therapy by Month 12 by Treatment Group ....56
Table 13: Summary Statistics for Comparison of Treatment Groups with Respect to Response Rate across Baseline HIV Status by Month 6 ....57
Table 14: Serological Response to Therapy by Each Timepoint by Treatment Group - ITT Population....58
Table 15: Serological Response to Therapy by Month 6 by Treatment Group and Baseline HIV Status....59
Table 16: Serological Response to Therapy at Month 12 by Treatment Group and Baseline HIV Status....59
Table 17: RPR Geometric Mean Titer (GMT) and RPR Geometric Mean Fold Decline (GMFD) by Visit, Treatment Group and Baseline HIV Status — ITT Population....60
Table 18: RPR Geometric Mean Titer (GMT) and RPR Geometric Mean Fold Decline (GMFD) by Visit, Treatment Group and Baseline HIV Status— Evaluable Population by Month 6 ....61
Table 19: RPR Geometric Mean Titer (GMT) and RPR Geometric Mean Fold Decline (GMFD) by Visit, Treatment Group and Baseline HIV Status— Evaluable Population by Month 12 ....61
Table 20: Subjects Compliant with Study Product by Treatment Group ....62

Table 21: Summary of Categorical Demographics and Socio-epidemiologic Characteristics at Baseline by HIV Status and Treatment Group — ITT Population ....63

Table 22: Summary of Continuous Demographics and Socio-epidemiologic Characteristics at Baseline by HIV Status and Treatment Group — ITT Population ....64

Table 23: Summary of Categorical Sexual History at Baseline by HIV Status and Treatment Group — ITT Population ....65

Table 24: Summary of Continuous Sexual History at Baseline by HIV Status and Treatment Group — ITT Population ....68

Table 25: Summary of Categorical Sexual History through Month 6 by Baseline HIV Status and Treatment Group— ITT Population....70

Table 26: Summary of Categorical Sexual History through Month 12 by Baseline HIV Status and Treatment Group — ITT Population....71

Table 27: Summary of Categorical Sexual History through Month 6 by Baseline HIV Status and Treatment Group — Evaluable Population by Month 6 ....71

Table 28: Summary of Categorical Sexual History through Month 12 by Baseline HIV Status and Treatment Group — Evaluable Population by Month 12 ....71

Table 29: Summary of Continuous Sexual History through Month 6 by Baseline HIV Status and Treatment Group ....72

Table 30: Summary of Continuous Sexual History through Month 12 by Baseline HIV Status and Treatment Group ....72

Table 31: Sensitivity analysis 1 - Serological Response to Therapy at Month 6 by Treatment Group – Removing Early Termination Due to COVID ....73

Table 32: Sensitivity analysis 2 - Serological Response to Therapy at Month 6 by Treatment Group – Re-defined Serological Response ....74

Table 33: Exploratory Analysis---Serological Response to Therapy by Month 6 by Treatment Group....75

Table 34: Summary of Subjects with Pre-Existing Medical Conditions by MedDRA System Organ Class and Treatment Group - Safety Population....76

Table 35: Number and Percentage of Subjects with Prior and Concomitant Medications by WHO Drug Classification and Treatment Group - Safety Population ....77

Table 36: Overall Summary of Adverse Events - Safety Population ....78

Table 37: Number and Percentage of Subjects Experiencing Solicited Events with 95% Confidence Intervals by Symptom and Treatment Group — Safety Population ....79


PRIVILEGED AND CONFIDENTIAL COMMUNICATION

Table 38: Number and Percentage of Subjects Experiencing Solicited Systemic Events by Symptom, Maximum Severity and Treatment Group — Safety Population.........80

Table 39: Number and Percentage of Subjects Experiencing Solicited Local Events with 95% Confidence Intervals by Symptom, Maximum Severity, Dose, and Treatment Group — Safety Population .....................................................................83

Table 40: The Association of Treatment with Jarisch-Herxheimer Reaction after Adjusting for HIV Co-infection status and Jarisch-Herxheimer Reaction (JHR) Onset Time Post-dose by Logistic Regression - Safety Population ................85

Table 41: Number and Percentage of Subjects Experiencing Unsolicited Adverse Events with 95% Confidence Intervals by MedDRA System Organ Class and Preferred Term, Dose Number, and Treatment Group — Safety Population .............................................................................................................86

Table 42: Number and Percentage of Subjects Experiencing Related Unsolicited Adverse Events with 95% Confidence Intervals by MedDRA System Organ Class and Preferred Term, Dose Number, and Treatment Group — Safety Population .............................................................................................................86

Table 43: Number of Unsolicited Adverse Events by MedDRA System Organ Class and Preferred Term, Maximum Severity and Relationship, and Treatment Group — Safety Population.........................................................................................87

Table 44: Number and Percentage of Subjects Experiencing Unsolicited Adverse Events by MedDRA System Organ Class and Preferred Term, Maximum Severity and Relationship, and Treatment Group — Safety Population....................88

Table 45: Unsolicited Adverse Events Occurring in ≥5% of Subjects in Any Treatment Group by MedDRA System Organ Class, Preferred Term, and Treatment Group - Safety Population ..........................................................................................89

Table 46: Solicited Adverse Events Occurring in ≥5% of Subjects in Any Treatment Group by MedDRA System Organ Class, Preferred Term, and Treatment Group - Safety Population ..........................................................................................89

Table 47: Listing of Serious Adverse Events – All Enrolled Subjects......................................90

Table 48: Listing of Non-Serious, Unsolicited Adverse Events – All Enrolled Subjects ..........91

Table 49: Laboratory Summary Statistics by Time Point and Treatment Group – CD4 Count in HIV-infected Subjects.........................................................................93

Table 50: Laboratory Summary Statistics by Time Point and Treatment Group – Viral Load in HIV-infected Subjects .........................................................................94

Table 51: Vital Signs by Maximum Severity, Time Point, and Treatment Group – Any Assessment – Safety Population.........................................................................95

Table 52: Vital Signs by Maximum Severity, Time Point, and Treatment Group – Temperature – Safety Population .........................................................................95


PRIVILEGED AND CONFIDENTIAL COMMUNICATION

Table 53: Vital Signs by Maximum Severity, Time Point, and Treatment Group – Blood Pressure – Safety Population ....95

Table 54: Vital Signs by Maximum Severity, Time Point, and Treatment Group – Pulse – Safety Population ....95

Table 55: Vital Signs by Maximum Severity, Time Point, and Treatment Group – Respiration – Safety Population ....95



PRIVILEGED AND CONFIDENTIAL COMMUNICATION

**Table 1: Study Design**

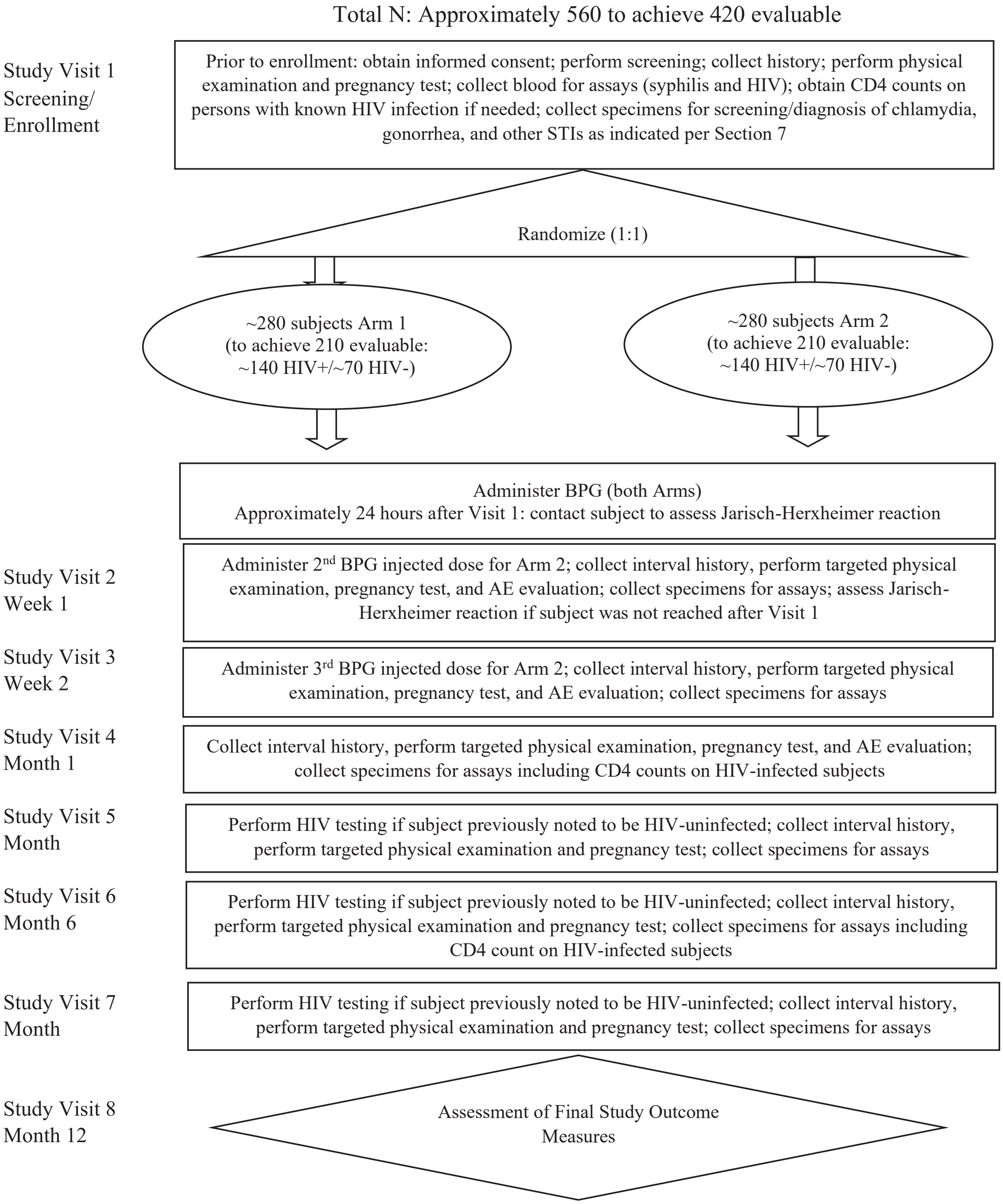

## Table 2: Schedule of Study Procedures

| Procedures | Visit 1 – Screening/ Enrollment (Day 1) | Contact (approx. 24 hours after Visit 1) | Visit 2 (Week 1 – Day 7 to 13) | Visit 3 (Week 2 – 6 to 12 days after Visit 2) | Visit 4 (Month 1 – Day 30 ± 7 days) | Visit 5 (Month 3 – Day 90 ± 21 days) | Visit 6 (Month 6 – Day 180 ± 21 days) | Visit 7 (Month 9 – Day 270 ± 28 days) | Visit 8 (Month 12 – Day 360 ± 28 days) | Early Termination | Unscheduled Visit[15] |
|---|---|---|---|---|---|---|---|---|---|---|---|
| Informed consent | X | | | | | | | | | | |
| Demographics | X | | | | | | | | | | |
| Eligibility criteria | X | | | | | | | | | | |
| Medical history[1] | X | | X | X | X | X | X | X | X | X | X |
| Sexual history[2] | X | | X | X | X | X | X | X | X | X | X |
| Targeted physical exam[3] | X | | X | X | X | X | X | X | X | X | X |
| Pregnancy test[4] | (X) | | (X) | (X) | (X) | (X) | (X) | (X) | (X) | (X) | (X) |
| Concomitant medications[5] | X | | X | X | X | X | X | X | X | X | X |
| Specimen collection: RPR titer | X | | X | X | X | X | X | X | X | X | |
| Specimen collection: Storage | X | | X | X | X | X | X | X | X | X | |
| Specimen collection: CD4 count[6] | (X) | | | | (X) | | (X) | | (X) | (X) | |
| Specimen collection: HIV testing[7] | (X) | | | | | (X) | (X) | (X) | (X) | (X) | |
| Specimen collection: Other STI screening/diagnosis[8] | X | | (X) | (X) | (X) | (X) | (X) | (X) | (X) | (X) | (X) |
| Specimen collection: Oral cavity and lesions (if present) swabbed[9] | (X) | | (X) | (X) | (X) | (X) | (X) | (X) | (X) | (X) | (X) |
| Randomization | X | | | | | | | | | | |
| BPG administered[10] | X | | (X) | (X) | | | | | | | |
| Contact information collected/reviewed[11] | X | | X | X | X | X | X | X | X | X | X |
| Jarisch-Herxheimer checklist[12] | X | X | (X) | | | | | | | | |
| Jarisch-Herxheimer reaction assessed[13] | | X | (X) | | | | | | | | |
| Resolution of syphilis signs assessed | | | X | X | | | | | | | |
| AEs/SAEs assessed[14] | X | | X | X | X | | | | | | X |

| Procedures | Visit 1 – Screening/ Enrollment (Day 1) | Contact (approx. 24 hours after Visit 1) | Visit 2 (Week 1 – Day 7 to 13) | Visit 3 (Week 2 – 6 to 12 days after Visit 2) | Visit 4 (Month 1 – Day 30 ± 7 days) | Visit 5 (Month 3 – Day 90 ± 21 days) | Visit 6 (Month 6 – Day 180 ± 21 days) | Visit 7 (Month 9 – Day 270 ± 28 days) | Visit 8 (Month 12 – Day 360 ± 28 days) | Early Termination | Unscheduled Visit[15] |
|---|---|---|---|---|---|---|---|---|---|---|---|

(X) – As indicated/appropriate. Refer to footnotes below and protocol Section 7.
[1] At Visit 1, collect complete medical history (including review of medical records for the past 14 days, if available). At subsequent visits, review medical history (including interval medical records, if available) and update as appropriate.
[2] At Visit 1, collect sexual history for the past 60 days. At subsequent visits, collect interim sexual history since last visit.
[3] Targeted physical exam includes vital signs: temperature, heart rate, respiration rate, and blood pressure; and genital, rectal, oral, skin, and lymph node examinations.
[4] Perform on subjects of childbearing potential. At Visit 1, urine or serum pregnancy test is permitted. At subsequent visits, perform urine pregnancy test.
[5] At Visit 1, record concomitant medications taken in the last 30 days before initiating BPG. At subsequent visits, record all concomitant medications taken since the last visit and update previously recorded medications as appropriate.
[6] Collect for subjects with known HIV infection whose medical records do not include a CD4 count in the past 30 days; at Visit 4, this is only applicable to subjects newly found to have HIV infection from Visit 1 to Visit 4 (inclusive).
[7] Perform for subjects who do not have a previously documented positive HIV test result using locally available tests (e.g., nucleic acid tests (NATs), antibody/antigen tests).
[8] At Visit 1, collect specimens at sites of exposure for screening/diagnosis of chlamydia and gonorrhea for all subjects who have not been tested and have been sexually active in the past 14 days and for other STIs as indicated by local standard of care and subject history. At subsequent visits, collect specimens at sites of exposure for STI testing per local standard of care and subject history.
[9] It is optional to swab subject's oral cavity and lesions, if lesions are present. See protocol Section 8.2.2.
[10] At Visit 1, administer BPG to all subjects. At Visits 2 and 3, administer BPG to subjects in Arm 2 only.
[11] At Visit 1, collect contact information. At subsequent visits, review contact information and update if needed.
[12] Distribute checklist at Visit 1. Subject refers to completed checklist during Jarisch-Herxheimer assessment; see protocol Sections 7 and 8.1.
[13] Assess per protocol Section 8.1. If subject is not reached, assess at Visit 2.
[14] Assess and record all AEs/SAEs (including solicited reactogenicity AEs and other unsolicited AEs).
[15] At Unscheduled Visits, any of the specified evaluations that are relevant to the subject may be performed at the discretion of the site PI.

**Table 3: Dates of First Treatment by Site – Safety Population**

[Implementation Note: Dates will be categorized based on length of enrollment period; try breaking the calendar year into halves first, if too many rows, will discuss and adjust the length.]

| Dates of Dosing | Site 1 (N = X) | | Site 2 (N = X) | | … | All Subjects (N = X) | |
|---|---|---|---|---|---|---|---|
| | n | % | n | % | … | n | % |
| DDMMMYYYY-DDMMMYYYY | x | x | x | x | … | x | x |
| DDMMMYYYY-DDMMMYYYY | x | x | x | x | … | x | x |
| DDMMMYYYY-DDMMMYYYY | x | x | x | x | … | x | x |
| DDMMMYYYY-DDMMMYYYY | x | x | x | x | … | x | x |

Note: N=Number of subjects in the safety population


PRIVILEGED AND CONFIDENTIAL COMMUNICATION

**Table 4: Dates of First Treatment by Treatment Group - Safety Population**

[Implementation Note: Dates will be categorized based on length of enrollment period; try breaking the calendar year into halves first, if too many rows, will discuss and adjust the length.]

| Dates of Dosing | One Dose BPG 2.4 MU (N = X) | | Three Doses BPG 2.4 MU (N = X) | | All Subjects (N = X) | |
|---|---|---|---|---|---|---|
| | n | % | n | % | n | % |
| DDMMMYYYY-DDMMMYYYY | x | x | x | x | x | x |
| DDMMMYYYY-DDMMMYYYY | x | x | x | x | x | x |
| DDMMMYYYY-DDMMMYYYY | x | x | x | x | x | x |
| DDMMMYYYY-DDMMMYYYY | x | x | x | x | x | x |

Note: N=Number of subjects in the safety population

**Table 5: ITT and mITT Analysis Population Eligibilities by Treatment Group - All Enrolled subjects**

| | | | One Dose BPG 2.4 MU (N = X) | | Three Doses BPG 2.4 MU (N = X) | | All Subjects (N=X) | |
|---|---|---|---|---|---|---|---|---|
| **Analysis Population** | **Eligibility Category** | **Reason Subjects Excluded** | **n** | **%** | **n** | **%** | **n** | **%** |
| Intent-to-Treat Analysis Population | Eligible for ITT Population | N/A | x | 100 | x | 100 | x | 100 |
| | Excluded from ITT Population | Any Reason | x | - | x | - | x | - |
| | | Failed screening | x | - | x | - | x | - |
| Modified Intent-to-Treat Analysis Population | Eligible for mITT Population | N/A | x | xx | x | xx | x | xx |
| | Excluded from mITT Population | Any Reason | x | xx | x | xx | x | xx |
| | | not eligible at the baseline visit | x | xx | x | xx | x | xx |
| | | Did not have a positive TPPA result at the baseline visit and/or at Visit 5 (Month 3), if repeat testing was performed | x | xx | x | xx | x | xx |

Notes: N, the denominator of percentages is the number of enrolled subjects in the specified treatment.
Treatment group is the intended randomized group.
Refer to Listing 1 for subjects who are excluded from the Analysis populations.

**Table 6: Safety and Evaluable Population Eligibilities by Treatment Group**

| | | | One Dose BPG 2.4 MU (N = X) | | Three Doses BPG 2.4 MU (N = X) | | All Subjects (N=X) | |
|---|---|---|---|---|---|---|---|---|
| **Analysis Population** | **Eligibility Category** | **Reason Subjects Excluded** | **n** | **%** | **n** | **%** | **n** | **%** |
| Safety Analysis Population | Eligible for Safety Population | N/A | x | x | x | x | x | x |
| | Excluded from Safety Population | Any Reason | x | x | x | x | x | x |
| | | Did not receive at least one dose of study product | x | x | x | x | x | x |
| Evaluable Analysis Population by Month 6 | Eligible for Evaluable Population by Month 6 | N/A | x | x | x | x | x | x |
| | Excluded from Evaluable Population by Month 6 | Any Reason | x | x | x | x | x | x |
| | | HIV status unknown at the baseline visit | x | x | x | x | x | x |
| | | Did not have a positive TPPA result at the baseline visit and/or at Visit 5 (Month 3), if repeat testing was performed | x | x | x | x | x | x |
| | | Did not receive all assigned doses of BPG | x | x | x | x | x | x |
| | | Did not have adequate RPR titer data available at baseline and applicable follow-up visit(s) by Month 6 | x | x | x | x | x | x |
| | | Received antibiotic(s) active against *T. pallidum* between enrollment and the applicable follow-up visit(s) by Month 6 for reasons other than syphilis infection[a] | x | x | x | x | x | x |
| | | HIV-uninfected status did not persist through applicable follow-up visit(s) by Month 6 (for HIV-uninfected subjects only) | x | x | x | x | x | x |
| Evaluable Analysis Population by Month 12 | Eligible for Evaluable Population by Month 12 | N/A | x | x | x | x | x | x |
| | Excluded from Evaluable Population by Month 12 | Any Reason | x | x | x | x | x | x |
| | | HIV status unknown at or before the baseline visit | x | x | x | x | x | x |
| | | Did not have a positive TPPA result at the baseline visit and/or at Visit 5 (Month 3), if repeat testing was performed | x | x | x | x | x | x |
| | | Did not receive all assigned doses of BPG | x | x | x | x | x | x |
| | | Did not have adequate RPR titer data available at baseline and applicable follow-up visit(s) by Month 12 | x | x | x | x | x | x |

| | | | One Dose BPG 2.4 MU (N = X) | | Three Doses BPG 2.4 MU (N = X) | | All Subjects (N=X) | |
|---|---|---|---|---|---|---|---|---|
| **Analysis Population** | **Eligibility Category** | **Reason Subjects Excluded** | **n** | **%** | **n** | **%** | **n** | **%** |
| | | Received antibiotic(s) active against *T. pallidum* between enrollment and the applicable follow-up visit(s) by Month 12 [a] | x | x | x | x | x | x |
| | | HIV-uninfected status did not persist through applicable follow-up visit(s) by Month 12 (for HIV-uninfected subjects only) | x | x | x | x | x | x |

Notes: Denominator of percentages is the number of enrolled subjects in the specified treatment group. N=number of enrolled subjects in the specified treatment group
Treatment group is the actual treatment a subject received.
Refer to Listing 1 for subjects who are excluded from the Analysis populations.

[a] A blinded case review committee reviewed subjects' data and determined if the antibiotics use effect the subjects' inclusion eligibility in evaluable populations.

### Table 7: Summary of Halting Rules - Safety Population

| Halting Rules | Halting Rule Triggered (Yes/No) | # Contributing to Halting Rule/ Total Needed to Halt |
|---|---|---|
| If one or more subjects experience an SAE judged by an investigator to be related to the study drug | Yes/No | x/1 |
| An overall pattern of clinical events that the DMID Medical Monitor or DSMB consider associated with the study drug and that may appear minor in terms of individual events, but that may collectively represent a serious potential concern for safety | Yes/No | x/1 |

## Table 8: Summary of Screen Failures

| Inclusion/Exclusion Category | Inclusion/Exclusion Criterion | n[a] | %[b] |
|---|---|---|---|
| Inclusion and Exclusion | Number of subjects failing any eligibility criterion | x | 100 |
| Inclusion | Any inclusion criterion | x | xx |
| | [inclusion criterion 1] | x | xx |
| | [inclusion criterion 2] | x | xx |
| | [inclusion criterion 3] | x | xx |
| Exclusion | Any exclusion criterion | x | xx |
| | [exclusion criterion 1] | x | xx |
| | [exclusion criterion 2] | x | xx |
| | [exclusion criterion 3] | x | xx |

[a] More than one criterion may be marked per subject.
[b] Denominator for percentages is the total number of screen failures.

**Table 9: Subject Disposition by Treatment Group- All Enrolled Subjects**

| Subject Disposition | One Dose BPG 2.4 MU (N = X) | | Three Doses BPG 2.4 MU (N = X) | | All Subjects (N=X) | |
|---|---|---|---|---|---|---|
| | n | % | n | % | n | % |
| Screened | N/A | N/A | N/A | N/A | x | N/A |
| Enrolled/Randomized | x | 100 | x | 100 | x | 100 |
| Received the First Dose of Treatment | x | xx | x | xx | x | xx |
| Received All Scheduled Treatment[a] | x | xx | x | xx | x | xx |
| Complied with Treatment[b] | x | xx | x | xx | x | xx |
| Completed Month 6 RPR Blood Draw | x | xx | x | xx | x | xx |
| Completed Month 12 RPR Blood Draw | x | xx | x | xx | x | xx |
| Completed Follow-up Visit 8 – Month 12 (Day 360±28 days)[a] | x | xx | x | xx | x | xx |

Notes: N=Number of enrolled subjects in the specified treatment group.
[a] Refer to Listing 2 for reasons subjects discontinued or terminated early.
[b] Subjects are considered compliant to the treatment if subjects receive all assigned doses within the assigned visit windows.

**Table 10: Distribution of Protocol Deviations by Category, Type, and Treatment Group – All Enrolled subjects**

| Category | Deviation Type | One Dose BPG 2.4 MU (N = X) | | Three Doses BPG 2.4 MU (N = X) | | All Subjects (N=X) | |
|---|---|---|---|---|---|---|---|
| | | No. of Subj. | No. of Dev. | No. of Subj. | No. of Dev. | No. of Subj. | No. of Dev. |
| Treatment administration schedule | Any type | x | x | x | x | x | x |
| | Out of window visit | x | x | x | x | x | x |
| | Missed visit/visit not conducted | x | x | x | x | x | x |
| | Missed treatment administration | x | x | x | x | x | x |
| | Delayed treatment administration | x | x | x | x | x | x |
| | Other | x | x | x | x | x | x |
| Follow-up visit schedule | Any type | x | x | x | x | x | x |
| | Out of window visit | x | x | x | x | x | x |
| | Missed visit/visit not conducted | x | x | x | x | x | x |
| | Other | x | x | x | x | x | x |
| Protocol procedure/assessment | Any type | x | x | x | x | x | x |
| | Incorrect version of ICF signed | x | x | x | x | x | x |
| | Blood not collected | x | x | x | x | x | x |
| | Other specimen not collected | x | x | x | x | x | x |
| | Too few aliquots obtained | x | x | x | x | x | x |
| | Specimen result not obtained | x | x | x | x | x | x |
| | Required procedure not conducted | x | x | x | x | x | x |
| | Required procedure done incorrectly | x | x | x | x | x | x |
| | Study product temperature excursion | x | x | x | x | x | x |
| | Specimen temperature excursion | x | x | x | x | x | x |
| | Other | x | x | x | x | x | x |
| Treatment administration | Any type | x | x | x | x | x | x |
| | Required procedure done incorrectly | x | x | x | x | x | x |
| | Study product temperature excursion | x | x | x | x | x | x |
| | Other | x | x | x | x | x | x |

Note: N=Number of enrolled subjects randomized to the specified treatment group.


PRIVILEGED AND CONFIDENTIAL COMMUNICATION

### Table 11: Serological Response to Therapy by Month 6 by Treatment Group

| Population | Treatment Group* | Number of Subjects with Serological Response, n | Number of Subjects, N | Proportion of Subjects with Serological Response (95% CI)[a] | Difference in Proportion of Subjects with Serological Response Between Two Treatment Groups (95% CI)[a] | P-Value[b] |
|---|---|---|---|---|---|---|
| **ITT Population** | **One Dose BPG 2.4 MU** | xxx | xxx | 0.xx(0.xx, 0.xx) | 0.xx(0.xx, 0.xx) | 0.xxx |
| | **Three Doses BPG 2.4 MU** | xxx | xxx | 0.xx(0.xx, 0.xx) | | |
| **mITT Population** | **One Dose BPG 2.4 MU** | xxx | xxx | 0.xx(0.xx, 0.xx) | 0.xx(0.xx, 0.xx) | 0.xxx |
| | **Three Doses BPG 2.4 MU** | xxx | xxx | 0.xx(0.xx, 0.xx) | | |
| **Evaluable Population** | **One Dose BPG 2.4 MU** | xxx | xxx | 0.xx(0.xx, 0.xx) | 0.xx(0.xx, 0.xx) | 0.xxx |
| | **Three Doses BPG 2.4 MU** | xxx | xxx | 0.xx(0.xx, 0.xx) | | |

[a]The denominator for proportion is based on the number of subjects enrolled in the specified treatment group and analysis population. 95% CI = 95% Wilson Confidence Interval
[b] P-value is calculated based on the noninferiority analysis for the proportion difference (one-dose group is non-inferior to three-dose group) by Farrington-Manning method with a 10% margin.
*Treatment group is the intended randomized group for ITT and mITT populations. Treatment group is the actual treatment a subject received for the Evaluable population.

**Table 12: Serological Response to Therapy by Month 12 by Treatment Group**

| Population | Treatment Group | Number of Subjects with Serological Response, n | Number of Subjects, N | Proportion of Subjects with Serological Response (95% CI)[a] | Difference in Proportion of Subjects with Serological Response Between Two Treatment Groups (95% CI) | P-Value[b] |
|---|---|---|---|---|---|---|
| ITT Population | One Dose BPG 2.4 MU | xxx | xxx | 0.xx(0.xx, 0.xx) | 0.xx(0.xx, 0.xx) | 0.xxx |
| | Three Doses BPG 2.4 MU | xxx | xxx | 0.xx(0.xx, 0.xx) | | |
| Evaluable Population | One Dose BPG 2.4 MU | xxx | xxx | 0.xx(0.xx, 0.xx) | 0.xx(0.xx, 0.xx) | 0.xxx |
| | Three Doses BPG 2.4 MU | xxx | xxx | 0.xx(0.xx, 0.xx) | | |

[a]The denominator for proportion is based on the number of subjects enrolled in the specified treatment group and analysis population. 95% CI = 95% Wilson Confidence Interval
[b] P-value is calculated based on the noninferiority analysis for the proportion difference (one-dose group is non-inferior to three-dose group) by Farrington-Manning method with a 10% margin.
*Treatment group is the intended randomized group for ITT population. Treatment group is the actual treatment a subject received for the Evaluable population.

**Table 13: Summary Statistics for Comparison of Treatment Groups with Respect to Response Rate across Baseline HIV Status by Month 6**

| Population | Test Method | Statistic | Result |
|---|---|---|---|
| ITT Population | Cochran-Mantel-Haenszel Test | Relative Risk - HIV positive (95% CI) | x.xxx(x.xxx, x.xxx) |
| | | Relative Risk - HIV negative (95% CI) | x.xxx(x.xxx, x.xxx) |
| | | Common Relative Risk[a] (95% CI) | x.xxx(x.xxx, x.xxx) |
| | | P-value | 0.xxx |
| | Breslow-Day Test for Homogeneity of the Odds Ratios | Common Odds Ratio | x.xxx(x.xxx, x.xxx) |
| | | P-value | 0.xxx |
| Evaluable Population | Cochran-Mantel-Haenszel Test | Relative Risk - HIV positive (95% CI) | x.xxx(x.xxx, x.xxx) |
| | | Relative Risk - HIV negative (95% CI) | x.xxx(x.xxx, x.xxx) |
| | | Common Relative Risk[a] (95% CI) | x.xxx(x.xxx, x.xxx) |
| | | P-value | 0.xxx |
| | Breslow-Day Test for Homogeneity of the Odds Ratios | Common Odds Ratio | x.xxx(x.xxx, x.xxx) |
| | | P-value | 0.xxx |

[a] The denominator for proportion is based on the number of subjects enrolled in the specified treatment group, HIV status and analysis population. The numerator is the number of subjects who were responders in in the specified treatment group, HIV status and analysis population.

## Table 14: Serological Response to Therapy by Each Timepoint by Treatment Group - ITT Population

| Time | Treatment Group | Number of Subjects with Serological Response, n | Number of Subjects, N | Proportion of Subjects with Serological Response (95% CI)[a] |
|---|---|---|---|---|
| **Week 1** | **One Dose BPG 2.4 MU** | xxx | xxx | 0.xx(0.xx, 0.xx) |
| | **Three Doses BPG 2.4 MU** | xxx | xxx | 0.xx(0.xx, 0.xx) |
| **Week 2** | **One Dose BPG 2.4 MU** | xxx | xxx | 0.xx(0.xx, 0.xx) |
| | **Three Doses BPG 2.4 MU** | xxx | xxx | 0.xx(0.xx, 0.xx) |
| **Week 3** | **One Dose BPG 2.4 MU** | xxx | xxx | 0.xx(0.xx, 0.xx) |
| | **Three Doses BPG 2.4 MU** | xxx | xxx | 0.xx(0.xx, 0.xx) |
| **Month 1** | **One Dose BPG 2.4 MU** | xxx | xxx | 0.xx(0.xx, 0.xx) |
| | **Three Doses BPG 2.4 MU** | xxx | xxx | 0.xx(0.xx, 0.xx) |
| **Month 3** | **One Dose BPG 2.4 MU** | xxx | xxx | 0.xx(0.xx, 0.xx) |
| | **Three Doses BPG 2.4 MU** | xxx | xxx | 0.xx(0.xx, 0.xx) |
| **Month 6** | **One Dose BPG 2.4 MU** | xxx | xxx | 0.xx(0.xx, 0.xx) |
| | **Three Doses BPG 2.4 MU** | xxx | xxx | 0.xx(0.xx, 0.xx) |
| **Month 9** | **One Dose BPG 2.4 MU** | xxx | xxx | 0.xx(0.xx, 0.xx) |
| | **Three Doses BPG 2.4 MU** | xxx | xxx | 0.xx(0.xx, 0.xx) |
| **Month 12** | **One Dose BPG 2.4 MU** | xxx | xxx | 0.xx(0.xx, 0.xx) |
| | **Three Doses BPG 2.4 MU** | xxx | xxx | 0.xx(0.xx, 0.xx) |

[a]The denominator for proportion is based on the number of subjects enrolled in the specified treatment group and analysis population. 95% CI = 95% Wilson Confidence Interval

**Table 15: Serological Response to Therapy by Month 6 by Treatment Group and Baseline HIV Status**

| Population | Baseline HIV Status | Treatment Group | Number of Subjects with Serological Response, n | Number of Subjects, N | Proportion of Subject with Serological Response (95% CI) | Difference in Proportion of Subjects with Serological Response Between Two Treatment Groups |
|---|---|---|---|---|---|---|
| ITT Population | HIV-infected | One Dose BPG 2.4 MU | xxx | xxx | 0.xx (0.xx, 0.xx) | 0.xx (0.xx, 0.xx) |
| | | Three Doses BPG 2.4 MU | xxx | xxx | 0.xx (0.xx, 0.xx) | |
| | | All Subjects | xxx | xxx | 0.xx (0.xx, 0.xx) | N/A |
| | HIV-uninfected | One Dose BPG 2.4 MU | xxx | xxx | 0.xx (0.xx, 0.xx) | 0.xx (0.xx, 0.xx) |
| | | Three Doses BPG 2.4 MU | xxx | xxx | 0.xx (0.xx, 0.xx) | |
| | | All Subjects | xxx | xxx | 0.xx (0.xx, 0.xx) | N/A |
| Evaluable Population | HIV-infected | One Dose BPG 2.4 MU | xxx | xxx | 0.xx (0.xx, 0.xx) | 0.xx (0.xx, 0.xx) |
| | | Three Doses BPG 2.4 MU | xxx | xxx | 0.xx (0.xx, 0.xx) | |
| | | All Subjects | xxx | xxx | 0.xx (0.xx, 0.xx) | N/A |
| | HIV-uninfected | One Dose BPG 2.4 MU | xxx | xxx | 0.xx (0.xx, 0.xx) | 0.xx (0.xx, 0.xx) |
| | | Three Doses BPG 2.4 MU | xxx | xxx | 0.xx (0.xx, 0.xx) | |
| | | All Subjects | xxx | xxx | 0.xx (0.xx, 0.xx) | N/A |

Notes: The denominator for proportion is based on the number of subjects enrolled in the respective treatment group and analysis population.
95% CI= 95% Wilson Confidence Interval

**Tables with similar format:**

**Table 16: Serological Response to Therapy at Month 12 by Treatment Group and Baseline HIV Status**


PRIVILEGED AND CONFIDENTIAL COMMUNICATION

**Table 17: RPR Geometric Mean Titer (GMT) and RPR Geometric Mean Fold Decline (GMFD) by Visit, Treatment Group and Baseline HIV Status — ITT Population**

[Implementation Note: Visit 1 will not have GMFD or GMFD 95% CI rows.]

| Time Point | Statistic | One Dose BPG 2.4 MU (N = X) | | | Three Doses BPG 2.4 MU (N = X) | | | All Subjects (N=X) | | |
|---|---|---|---|---|---|---|---|---|---|---|
| | | HIV-infected | HIV-uninfected | All | HIV-infected | HIV-uninfected | All | HIV-infected | HIV-uninfected | All |
| Baseline | n | x | x | x | x | x | x | x | x | x |
| | GMT | x.x | x.x | x.x | x.x | x.x | x.x | x.x | x.x | x.x |
| | 95% CI | x.xx, x.xx | x.xx, x.xx | x.xx, x.xx | x.xx, x.xx | x.xx, x.xx | x.xx, x.xx | x.xx, x.xx | x.xx, x.xx | x.xx, x.xx |
| Week 1 | n | x | x | x | x | x | x | x | x | x |
| | GMT | x.x | x.x | x.x | x.x | x.x | x.x | x.x | x.x | x.x |
| | GMT 95% CI | x.xx, x.xx | x.xx, x.xx | x.xx, x.xx | x.xx, x.xx | x.xx, x.xx | x.xx, x.xx | x.xx, x.xx | x.xx, x.xx | x.xx, x.xx |
| | GMFD | x.x | x.x | x.x | x.x | x.x | x.x | x.x | x.x | x.x |
| | GMFD 95% CI | x.xx, x.xx | x.xx, x.xx | x.xx, x.xx | x.xx, x.xx | x.xx, x.xx | x.xx, x.xx | x.xx, x.xx | x.xx, x.xx | x.xx, x.xx |
| Week 2 | n | x | x | x | x | x | x | x | x | x |
| | GMT | x.x | x.x | x.x | x.x | x.x | x.x | x.x | x.x | x.x |
| | GMT 95% CI | x.xx, x.xx | x.xx, x.xx | x.xx, x.xx | x.xx, x.xx | x.xx, x.xx | x.xx, x.xx | x.xx, x.xx | x.xx, x.xx | x.xx, x.xx |
| | GMFD | x.x | x.x | x.x | x.x | x.x | x.x | x.x | x.x | x.x |
| | GMFD 95% CI | x.xx, x.xx | x.xx, x.xx | x.xx, x.xx | x.xx, x.xx | x.xx, x.xx | x.xx, x.xx | x.xx, x.xx | x.xx, x.xx | x.xx, x.xx |
| Month 1 | n | x | x | x | x | x | x | x | x | x |
| | GMT | x.x | x.x | x.x | x.x | x.x | x.x | x.x | x.x | x.x |
| | GMT 95% CI | x.xx, x.xx | x.xx, x.xx | x.xx, x.xx | x.xx, x.xx | x.xx, x.xx | x.xx, x.xx | x.xx, x.xx | x.xx, x.xx | x.xx, x.xx |
| | GMFD | x.x | x.x | x.x | x.x | x.x | x.x | x.x | x.x | x.x |
| | GMFD 95% CI | x.xx, x.xx | x.xx, x.xx | x.xx, x.xx | x.xx, x.xx | x.xx, x.xx | x.xx, x.xx | x.xx, x.xx | x.xx, x.xx | x.xx, x.xx |
| Month 2 | n | x | x | x | x | x | x | x | x | x |
| | GMT | x.xx, x.xx | x.xx, x.xx | x.xx, x.xx | x.xx, x.xx | x.xx, x.xx | x.xx, x.xx | x.xx, x.xx | x.xx, x.xx | x.xx, x.xx |
| | GMT 95% CI | x.xx, x.xx | x.xx, x.xx | x.xx, x.xx | x.xx, x.xx | x.xx, x.xx | x.xx, x.xx | x.xx, x.xx | x.xx, x.xx | x.xx, x.xx |
| | GMFD | x.x | x.x | x.x | x.x | x.x | x.x | x.x | x.x | x.x |
| | GMFD 95% CI | x.xx, x.xx | x.xx, x.xx | x.xx, x.xx | x.xx, x.xx | x.xx, x.xx | x.xx, x.xx | x.xx, x.xx | x.xx, x.xx | x.xx, x.xx |

| Time Point | Statistic | One Dose BPG 2.4 MU (N = X) | | | Three Doses BPG 2.4 MU (N = X) | | | All Subjects (N=X) | | |
|---|---|---|---|---|---|---|---|---|---|---|
| | | HIV-infected | HIV-uninfected | All | HIV-infected | HIV-uninfected | All | HIV-infected | HIV-uninfected | All |
| **Month 6** | n | x | x | x | x | x | x | x | x | x |
| | GMT | x.x | x.x | x.x | x.x | x.x | x.x | x.x | x.x | x.x |
| | GMT 95% CI | x.xx, x.xx | x.xx, x.xx | x.xx, x.xx | x.xx, x.xx | x.xx, x.xx | x.xx, x.xx | x.xx, x.xx | x.xx, x.xx | x.xx, x.xx |
| | GMFD | x.x | x.x | x.x | x.x | x.x | x.x | x.x | x.x | x.x |
| | GMFD 95% CI | x.xx, x.xx | x.xx, x.xx | x.xx, x.xx | x.xx, x.xx | x.xx, x.xx | x.xx, x.xx | x.xx, x.xx | x.xx, x.xx | x.xx, x.xx |
| **Month 9** | n | x | x | x | x | x | x | x | x | x |
| | GMT | x.x | x.x | x.x | x.x | x.x | x.x | x.x | x.x | x.x |
| | GMT 95% CI | x.xx, x.xx | x.xx, x.xx | x.xx, x.xx | x.xx, x.xx | x.xx, x.xx | x.xx, x.xx | x.xx, x.xx | x.xx, x.xx | x.xx, x.xx |
| | GMFD | x.x | x.x | x.x | x.x | x.x | x.x | x.x | x.x | x.x |
| | GMFD 95% CI | x.xx, x.xx | x.xx, x.xx | x.xx, x.xx | x.xx, x.xx | x.xx, x.xx | x.xx, x.xx | x.xx, x.xx | x.xx, x.xx | x.xx, x.xx |
| **Month 12** | n | x | x | x | x | x | x | x | x | x |
| | GMT | x.x | x.x | x.x | x.x | x.x | x.x | x.x | x.x | x.x |
| | GMT 95% CI | x.xx, x.xx | x.xx, x.xx | x.xx, x.xx | x.xx, x.xx | x.xx, x.xx | x.xx, x.xx | x.xx, x.xx | x.xx, x.xx | x.xx, x.xx |
| | GMFD | x.x | x.x | x.x | x.x | x.x | x.x | x.x | x.x | x.x |
| | GMFD 95% CI | x.xx, x.xx | x.xx, x.xx | x.xx, x.xx | x.xx, x.xx | x.xx, x.xx | x.xx, x.xx | x.xx, x.xx | x.xx, x.xx | x.xx, x.xx |

Notes: N=Number of subjects who had RPR titer results available in the specified analysis population in the specified treatment group at the specified visit. RPR titer results from the early termination visit that occurred between scheduled visits are excluded.
GMFD represents the geometric mean fold decline in antibody counts compared to baseline

Tables with similar format:

**Table 18: RPR Geometric Mean Titer (GMT) and RPR Geometric Mean Fold Decline (GMFD) by Visit, Treatment Group and Baseline HIV Status— Evaluable Population by Month 6**

**Table 19: RPR Geometric Mean Titer (GMT) and RPR Geometric Mean Fold Decline (GMFD) by Visit, Treatment Group and Baseline HIV Status— Evaluable Population by Month 12**

## Table 20: Subjects Compliant with Study Product by Treatment Group

| Population | Treatment Group | Number of Compliant[a] Subjects, n | Number of Subjects, N | Proportion (95% CI) | Difference in Proportion Between Two Treatment Groups (95% CI) | P-Value[b] |
|---|---|---|---|---|---|---|
| **ITT Population** | **One Dose BPG 2.4 MU** | xxx | xxx | 0.xx (0.xx, 0.xx) | 0.xx (0.xx, 0.xx) | 0.xxx |
| | **Three Doses BPG 2.4 MU** | xxx | xxx | 0.xx (0.xx, 0.xx) | | |
| | **All Subjects** | xxx | xxx | 0.xx (0.xx, 0.xx) | N/A | N/A |
| **Evaluable Population by Month 6** | **One Dose BPG 2.4 MU** | xxx | xxx | 0.xx (0.xx, 0.xx) | 0.xx (0.xx, 0.xx) | 0.xxx |
| | **Three Doses BPG 2.4 MU** | xxx | xxx | 0.xx (0.xx, 0.xx) | | |
| | **All Subjects** | xxx | xxx | 0.xx (0.xx, 0.xx) | N/A | N/A |
| **Evaluable Population by Month 12** | **One Dose BPG 2.4 MU** | xxx | xxx | 0.xx (0.xx, 0.xx) | 0.xx (0.xx, 0.xx) | 0.xxx |
| | **Three Doses BPG 2.4 MU** | xxx | xxx | 0.xx (0.xx, 0.xx) | | |
| | **All Subjects** | xxx | xxx | 0.xx (0.xx, 0.xx) | N/A | N/A |

Notes: The denominator for proportion is based on the number of subjects enrolled in the specified treatment group and analysis population.
95% CI= 95% Wilson Confidence Interval
[a] Subjects are compliant if they receive all assigned doses within the assigned visit windows.
[b] P-value is calculated based on the two-sided Pearson Chi-Square test at the .05 level of significance.

**Table 21: Summary of Categorical Demographics and Socio-epidemiologic Characteristics at Baseline by HIV Status and Treatment Group — ITT Population**

| Variable | Characteristic | One Dose BPG 2.4 MU | | | | | | Three Doses BPG 2.4 MU | | | | | | All Subjects | | | | | |
|---|---|---|---|---|---|---|---|---|---|---|---|---|---|---|---|---|---|---|---|
| | | HIV-infected (N=X) | | HIV-uninfected (N=X) | | All Subjects (N=X) | | HIV-infected (N=X) | | HIV-uninfected (N=X) | | All Subjects (N=X) | | HIV-infected (N=X) | | HIV-uninfected (N=X) | | All Subjects (N=X) | |
| | | n | % | n | % | n | % | n | % | n | % | n | % | n | % | n | % | n | % |
| Sex | Male | x | xx | x | xx | x | xx | x | xx | x | xx | x | xx | x | xx | x | xx | x | xx |
| | Female | x | xx | x | xx | x | xx | x | xx | x | xx | x | xx | x | xx | x | xx | x | xx |
| Ethnicity | Not Hispanic or Latino | x | xx | x | xx | x | xx | x | xx | x | xx | x | xx | x | xx | x | xx | x | xx |
| | Hispanic or Latino | x | xx | x | xx | x | xx | x | xx | x | xx | x | xx | x | xx | x | xx | x | xx |
| | Not Reported | x | xx | x | xx | x | xx | x | xx | x | xx | x | xx | x | xx | x | xx | x | xx |
| | Unknown | x | xx | x | xx | x | xx | x | xx | x | xx | x | xx | x | xx | x | xx | x | xx |
| Race | American Indian or Alaskan Native | x | xx | x | xx | x | xx | x | xx | x | xx | x | xx | x | xx | x | xx | x | xx |
| | Asian | x | xx | x | xx | x | xx | x | xx | x | xx | x | xx | x | xx | x | xx | x | xx |
| | Native Hawaiian or Other Pacific Islander | x | xx | x | xx | x | xx | x | xx | x | xx | x | xx | x | xx | x | xx | x | xx |
| | Black or African American | x | xx | x | xx | x | xx | x | xx | x | xx | x | xx | x | xx | x | xx | x | xx |
| | White | x | xx | x | xx | x | xx | x | xx | x | xx | x | xx | x | xx | x | xx | x | xx |
| | Multi-Racial | x | xx | x | xx | x | xx | x | xx | x | xx | x | xx | x | xx | x | xx | x | xx |
| | Unknown | x | xx | x | xx | x | xx | x | xx | x | xx | x | xx | x | xx | x | xx | x | xx |
| Highest level of education completed? | Did not complete high school | x | x | x | x | x | x | x | x | x | x | x | x | x | x | x | x | x | x |
| | Completed high school | x | x | x | x | x | x | x | x | x | x | x | x | x | x | x | x | x | x |
| | Completed junior college | x | x | x | x | x | x | x | x | x | x | x | x | x | x | x | x | x | x |
| | undergraduate degree | x | x | x | x | x | x | x | x | x | x | x | x | x | x | x | x | x | x |
| | Completed graduate degree | x | x | x | x | x | x | x | x | x | x | x | x | x | x | x | x | x | x |

**Table 22: Summary of Continuous Demographics and Socio-epidemiologic Characteristics at Baseline by HIV Status and Treatment Group — ITT Population**

| Variable | Statistic | One Dose BPG 2.4 MU | | | Three Doses BPG 2.4 MU | | | All Subjects | | |
|---|---|---|---|---|---|---|---|---|---|---|
| | | HIV-infected (N=X) | HIV-uninfected (N=X) | All Subjects (N=X) | HIV-infected (N=X) | HIV-uninfected (N=X) | All Subjects (N=X) | HIV-infected (N=X) | HIV-uninfected (N=X) | All Subjects (N=X) |
| Age | Mean (SD) | x.x (x.x) | x.x (x.x) | x.x (x.x) | x.x (x.x) | x.x (x.x) | x.x (x.x) | x.x (x.x) | x.x (x.x) | x.x (x.x) |
| | Median | x.x | x.x | x.x | x.x | x.x | x.x | x.x | x.x | x.x |
| | Min, Max | x, x | x, x | x, x | x, x | x, x | x, x | x, x | x, x | x, x |
| Years of formal education | Mean (SD) | x.x (x.x) | x.x (x.x) | x.x (x.x) | x.x (x.x) | x.x (x.x) | x.x (x.x) | x.x (x.x) | x.x (x.x) | x.x (x.x) |
| | Median | x.x | x.x | x.x | x.x | x.x | x.x | x.x | x.x | x.x |
| | Min, Max | x, x | x, x | x, x | x, x | x, x | x, x | x, x | x, x | x, x |

**Table 23: Summary of Categorical Sexual History at Baseline by HIV Status and Treatment Group — ITT Population**

| | | One Dose BPG 2.4 MU | | | | | | Three Doses BPG 2.4 MU | | | | | | All Subjects | | | | | |
|---|---|---|---|---|---|---|---|---|---|---|---|---|---|---|---|---|---|---|---|
| | | HIV-infected (N=X) | | HIV-uninfected (N=X) | | All Subjects (N=X) | | HIV-infected (N=X) | | HIV-uninfected (N=X) | | All Subjects (N=X) | | HIV-infected (N=X) | | HIV-uninfected (N=X) | | All Subjects (N=X) | |
| **Variable** | **Characteristic** | **n** | **%** | **n** | **%** | **n** | **%** | **n** | **%** | **n** | **%** | **n** | **%** | **n** | **%** | **n** | **%** | **n** | **%** |
| Gender identity | Man | x | x | x | x | x | x | x | x | x | x | x | x | x | x | x | x | x | x |
| | Woman | x | x | x | x | x | x | x | x | x | x | x | x | x | x | x | x | x | x |
| | Trans male | x | x | x | x | x | x | x | x | x | x | x | x | x | x | x | x | x | x |
| | Trans female | x | x | x | x | x | x | x | x | x | x | x | x | x | x | x | x | x | x |
| | Non-conforming | x | x | x | x | x | x | x | x | x | x | x | x | x | x | x | x | x | x |
| | Multiple | x | x | x | x | x | x | x | x | x | x | x | x | x | x | x | x | x | x |
| | Non-binary | x | x | x | x | x | x | x | x | x | x | x | x | x | x | x | x | x | x |
| | Other | x | x | x | x | x | x | x | x | x | x | x | x | x | x | x | x | x | x |
| | Declined to respond | x | x | x | x | x | x | x | x | x | x | x | x | x | x | x | x | x | x |
| Sexual orientation | Heterosexual | x | x | x | x | x | x | x | x | x | x | x | x | x | x | x | x | x | x |
| | Homosexual | x | x | x | x | x | x | x | x | x | x | x | x | x | x | x | x | x | x |
| | Bisexual | x | x | x | x | x | x | x | x | x | x | x | x | x | x | x | x | x | x |
| | Other | x | x | x | x | x | x | x | x | x | x | x | x | x | x | x | x | x | x |
| | Declined to respond | x | x | x | x | x | x | x | x | x | x | x | x | x | x | x | x | x | x |
| Sexual partner | Men | x | x | x | x | x | x | x | x | x | x | x | x | x | x | x | x | x | x |
| | Women | x | x | x | x | x | x | x | x | x | x | x | x | x | x | x | x | x | x |
| | Trans males | x | x | x | x | x | x | x | x | x | x | x | x | x | x | x | x | x | x |
| | Transferals | x | x | x | x | x | x | x | x | x | x | x | x | x | x | x | x | x | x |
| | Multiple | x | x | x | x | x | x | x | x | x | x | x | x | x | x | x | x | x | x |
| | Other | x | x | x | x | x | x | x | x | x | x | x | x | x | x | x | x | x | x |
| | Declined to respond | x | x | x | x | x | x | x | x | x | x | x | x | x | x | x | x | x | x |

| Variable | | Characteristic | One Dose BPG 2.4 MU | | | | | | Three Doses BPG 2.4 MU | | | | | | All Subjects | | | | | |
|---|---|---|---|---|---|---|---|---|---|---|---|---|---|---|---|---|---|---|---|---|
| | | | HIV-infected (N=X) | | HIV-uninfected (N=X) | | All Subjects (N=X) | | HIV-infected (N=X) | | HIV-uninfected (N=X) | | All Subjects (N=X) | | HIV-infected (N=X) | | HIV-uninfected (N=X) | | All Subjects (N=X) | |
| | | | n | % | n | % | n | % | n | % | n | % | n | % | n | % | n | % | n | % |
| Sexual intercourse in the past 60 days? | | Yes | x | x | x | x | x | x | x | x | x | x | x | x | x | x | x | x | x | x |
| | | No | x | x | x | x | x | x | x | x | x | x | x | x | x | x | x | x | x | x |
| | | Unknown | x | x | x | x | x | x | x | x | x | x | x | x | x | x | x | x | x | x |
| If yes to above question, | Sites of exposure | Oral receptive | x | x | x | x | x | x | x | x | x | x | x | x | x | x | x | x | x | x |
| | | Oral active | x | x | x | x | x | x | x | x | x | x | x | x | x | x | x | x | x | x |
| | | Rectal receptive | x | x | x | x | x | x | x | x | x | x | x | x | x | x | x | x | x | x |
| | | Rectal insertive | x | x | x | x | x | x | x | x | x | x | x | x | x | x | x | x | x | x |
| | | Genital/vaginal | x | x | x | x | x | x | x | x | x | x | x | x | x | x | x | x | x | x |
| | Condom or barrier for protection? | Always | x | x | x | x | x | x | x | x | x | x | x | x | x | x | x | x | x | x |
| | | Sometimes | x | x | x | x | x | x | x | x | x | x | x | x | x | x | x | x | x | x |
| | | Never | x | x | x | x | x | x | x | x | x | x | x | x | x | x | x | x | x | x |
| Any known exposures to an STD in the past 60 days? | | Yes | x | x | x | x | x | x | x | x | x | x | x | x | x | x | x | x | x | x |
| | | No | x | x | x | x | x | x | x | x | x | x | x | x | x | x | x | x | x | x |
| | | Unknown | x | x | x | x | x | x | x | x | x | x | x | x | x | x | x | x | x | x |
| Diagnosed with gonorrhea in the past 60 days? | | Yes | x | x | x | x | x | x | x | x | x | x | x | x | x | x | x | x | x | x |
| | | No | x | x | x | x | x | x | x | x | x | x | x | x | x | x | x | x | x | x |
| Tested for gonorrhea in the past 14 days? | | Yes | x | x | x | x | x | x | x | x | x | x | x | x | x | x | x | x | x | x |
| | | No | x | x | x | x | x | x | x | x | x | x | x | x | x | x | x | x | x | x |
| If yes to above question, | Test results? | Positive | x | x | x | x | x | x | x | x | x | x | x | x | x | x | x | x | x | x |
| | | Negative | x | x | x | x | x | x | x | x | x | x | x | x | x | x | x | x | x | x |

| | | One Dose BPG 2.4 MU | | | | | | Three Doses BPG 2.4 MU | | | | | | All Subjects | | | | | |
|---|---|---|---|---|---|---|---|---|---|---|---|---|---|---|---|---|---|---|---|
| | | HIV-infected (N=X) | | HIV-uninfected (N=X) | | All Subjects (N=X) | | HIV-infected (N=X) | | HIV-uninfected (N=X) | | All Subjects (N=X) | | HIV-infected (N=X) | | HIV-uninfected (N=X) | | All Subjects (N=X) | |
| **Variable** | **Characteristic** | **n** | **%** | **n** | **%** | **n** | **%** | **n** | **%** | **n** | **%** | **n** | **%** | **n** | **%** | **n** | **%** | **n** | **%** |
| Diagnosed with chlamydia in the past 60 days? | Yes | x | x | x | x | x | x | x | x | x | x | x | x | x | x | x | x | x | x |
| | No | x | x | x | x | x | x | x | x | x | x | x | x | x | x | x | x | x | x |
| Tested for chlamydia in the past 14 days? | Yes | x | x | x | x | x | x | x | x | x | x | x | x | x | x | x | x | x | x |
| | No | x | x | x | x | x | x | x | x | x | x | x | x | x | x | x | x | x | x |
| If yes to above question, Test results? | Positive | x | x | x | x | x | x | x | x | x | x | x | x | x | x | x | x | x | x |
| | Negative | x | x | x | x | x | x | x | x | x | x | x | x | x | x | x | x | x | x |
| Diagnosed with any other STI in the past 60 days? | Yes | x | x | x | x | x | x | x | x | x | x | x | x | x | x | x | x | x | x |
| | No | x | x | x | x | x | x | x | x | x | x | x | x | x | x | x | x | x | x |

## Table 24: Summary of Continuous Sexual History at Baseline by HIV Status and Treatment Group — ITT Population

| Variable | Statistic | One Dose BPG 2.4 MU | | | Three Doses BPG 2.4 MU | | | All Subjects | | |
|---|---|---|---|---|---|---|---|---|---|---|
| | | HIV-infected (N=X) | HIV-uninfected (N=X) | All Subjects (N=X) | HIV-infected (N=X) | HIV-uninfected (N=X) | All Subjects (N=X) | HIV-infected (N=X) | HIV-uninfected (N=X) | All Subjects (N=X) |
| Days since last had sexual intercourse in the past 60 days | Mean (SD) | x.x (x.x) | x.x (x.x) | x.x (x.x) | x.x (x.x) | x.x (x.x) | x.x (x.x) | x.x (x.x) | x.x (x.x) | x.x (x.x) |
| | Median | x.x | x.x | x.x | x.x | x.x | x.x | x.x | x.x | x.x |
| | Min, Max | x, x | x, x | x, x | x, x | x, x | x, x | x, x | x, x | x, x |
| Total number of partners in the past 60 days | Mean (SD) | x.x (x.x) | x.x (x.x) | x.x (x.x) | x.x (x.x) | x.x (x.x) | x.x (x.x) | x.x (x.x) | x.x (x.x) | x.x (x.x) |
| | Median | x.x | x.x | x.x | x.x | x.x | x.x | x.x | x.x | x.x |
| | Min, Max | x, x | x, x | x, x | x, x | x, x | x, x | x, x | x, x | x, x |
| Number of new partners | Mean (SD) | x.x (x.x) | x.x (x.x) | x.x (x.x) | x.x (x.x) | x.x (x.x) | x.x (x.x) | x.x (x.x) | x.x (x.x) | x.x (x.x) |
| | Median | x.x | x.x | x.x | x.x | x.x | x.x | x.x | x.x | x.x |
| | Min, Max | x, x | x, x | x, x | x, x | x, x | x, x | x, x | x, x | x, x |
| Number of regular partners | Mean (SD) | x.x (x.x) | x.x (x.x) | x.x (x.x) | x.x (x.x) | x.x (x.x) | x.x (x.x) | x.x (x.x) | x.x (x.x) | x.x (x.x) |
| | Median | x.x | x.x | x.x | x.x | x.x | x.x | x.x | x.x | x.x |
| | Min, Max | x, x | x, x | x, x | x, x | x, x | x, x | x, x | x, x | x, x |
| Number of occasional partners | Mean (SD) | x.x (x.x) | x.x (x.x) | x.x (x.x) | x.x (x.x) | x.x (x.x) | x.x (x.x) | x.x (x.x) | x.x (x.x) | x.x (x.x) |
| | Median | x.x | x.x | x.x | x.x | x.x | x.x | x.x | x.x | x.x |
| | Min, Max | x, x | x, x | x, x | x, x | x, x | x, x | x, x | x, x | x, x |
| Number of male partners | Mean (SD) | x.x (x.x) | x.x (x.x) | x.x (x.x) | x.x (x.x) | x.x (x.x) | x.x (x.x) | x.x (x.x) | x.x (x.x) | x.x (x.x) |
| | Median | x.x | x.x | x.x | x.x | x.x | x.x | x.x | x.x | x.x |
| | Min, Max | x, x | x, x | x, x | x, x | x, x | x, x | x, x | x, x | x, x |
| Number of female partners | Mean (SD) | x.x (x.x) | x.x (x.x) | x.x (x.x) | x.x (x.x) | x.x (x.x) | x.x (x.x) | x.x (x.x) | x.x (x.x) | x.x (x.x) |
| | Median | x.x | x.x | x.x | x.x | x.x | x.x | x.x | x.x | x.x |
| | Min, Max | x, x | x, x | x, x | x, x | x, x | x, x | x, x | x, x | x, x |

| Variable | Statistic | One Dose BPG 2.4 MU | | | Three Doses BPG 2.4 MU | | | All Subjects | | |
|---|---|---|---|---|---|---|---|---|---|---|
| | | **HIV-infected (N=X)** | **HIV-uninfected (N=X)** | **All Subjects (N=X)** | **HIV-infected (N=X)** | **HIV-uninfected (N=X)** | **All Subjects (N=X)** | **HIV-infected (N=X)** | **HIV-uninfected (N=X)** | **All Subjects (N=X)** |
| Days since last diagnosed with gonorrhea in the past 60 days | Mean (SD) | x.x (x.x) | x.x (x.x) | x.x (x.x) | x.x (x.x) | x.x (x.x) | x.x (x.x) | x.x (x.x) | x.x (x.x) | x.x (x.x) |
| | Median | x.x | x.x | x.x | x.x | x.x | x.x | x.x | x.x | x.x |
| | Min, Max | x, x | x, x | x, x | x, x | x, x | x, x | x, x | x, x | x, x |
| Days since last diagnosed with chlamydia in the past 60 days | Mean (SD) | x.x (x.x) | x.x (x.x) | x.x (x.x) | x.x (x.x) | x.x (x.x) | x.x (x.x) | x.x (x.x) | x.x (x.x) | x.x (x.x) |
| | Median | x.x | x.x | x.x | x.x | x.x | x.x | x.x | x.x | x.x |
| | Min, Max | x, x | x, x | x, x | x, x | x, x | x, x | x, x | x, x | x, x |
| Days since last diagnosed with any other STI in the past 60 days | Mean (SD) | x.x (x.x) | x.x (x.x) | x.x (x.x) | x.x (x.x) | x.x (x.x) | x.x (x.x) | x.x (x.x) | x.x (x.x) | x.x (x.x) |
| | Median | x.x | x.x | x.x | x.x | x.x | x.x | x.x | x.x | x.x |
| | Min, Max | x, x | x, x | x, x | x, x | x, x | x, x | x, x | x, x | x, x |

**Table 25: Summary of Categorical Sexual History through Month 6 by Baseline HIV Status and Treatment Group— ITT Population**

[Implementation notes for table 25, 27, and 29, the Time will be Week1 Week2 month1. Month 3 and Month 6; and for table 26, 28 and 30, the Time will be Month 9 and Month 12.]

| Time | Variable | Characteristic | One Dose BPG 2.4 MU | | | | | | Three Doses BPG 2.4 MU | | | | | | All Subjects | | | | | |
|---|---|---|---|---|---|---|---|---|---|---|---|---|---|---|---|---|---|---|---|---|
| | | | Baseline HIV-infected (N=X) | | Baseline HIV-uninfected (N=X) | | All Subjects (N=X) | | Baseline HIV-infected (N=X) | | Baseline HIV-uninfected (N=X) | | All Subjects (N=X) | | Baseline HIV-infected (N=X) | | Baseline HIV-uninfected (N=X) | | All Subjects (N=X) | |
| | | | n | % | n | % | n | % | n | % | n | % | n | % | n | % | n | % | n | % |
| Week 1 | Sexual intercourse since last visit? | Yes | x | x | x | x | x | x | x | x | x | x | x | x | x | x | x | x | x | x |
| | | No | x | x | x | x | x | x | x | x | x | x | x | x | x | x | x | x | x | x |
| | | Unknown | x | x | x | x | x | x | x | x | x | x | x | x | x | x | x | x | x | x |
| | Since last visit, if the subject had sexual intercourse, indicate sites of exposure | Oral receptive | x | x | x | x | x | x | x | x | x | x | x | x | x | x | x | x | x | x |
| | | Oral active | x | x | x | x | x | x | x | x | x | x | x | x | x | x | x | x | x | x |
| | | Rectal receptive | x | x | x | x | x | x | x | x | x | x | x | x | x | x | x | x | x | x |
| | | Rectal insertive | x | x | x | x | x | x | x | x | x | x | x | x | x | x | x | x | x | x |
| | | Genital/vaginal | x | x | x | x | x | x | x | x | x | x | x | x | x | x | x | x | x | x |
| | Since last visit, if the subject had sexual intercourse, how often did the subject use a condom or barrier for protection? | Always | x | x | x | x | x | x | x | x | x | x | x | x | x | x | x | x | x | x |
| | | Sometimes | x | x | x | x | x | x | x | x | x | x | x | x | x | x | x | x | x | x |
| | | Never | x | x | x | x | x | x | x | x | x | x | x | x | x | x | x | x | x | x |
| | Any known exposures to syphilis since last visit? | Yes | x | x | x | x | x | x | x | x | x | x | x | x | x | x | x | x | x | x |
| | | No | x | x | x | x | x | x | x | x | x | x | x | x | x | x | x | x | x | x |
| | | Unknown | x | x | x | x | x | x | x | x | x | x | x | x | x | x | x | x | x | x |
| | Any known exposures to another STD since last visit? | Yes | x | x | x | x | x | x | x | x | x | x | x | x | x | x | x | x | x | x |
| | | No | x | x | x | x | x | x | x | x | x | x | x | x | x | x | x | x | x | x |
| | | Unknown | x | x | x | x | x | x | x | x | x | x | x | x | x | x | x | x | x | x |

| Time | Variable | Characteristic | One Dose BPG 2.4 MU | | | | | | Three Doses BPG 2.4 MU | | | | | | All Subjects | | | | | |
|---|---|---|---|---|---|---|---|---|---|---|---|---|---|---|---|---|---|---|---|---|
| | | | Baseline HIV-infected (N=X) | | Baseline HIV-uninfected (N=X) | | All Subjects (N=X) | | Baseline HIV-infected (N=X) | | Baseline HIV-uninfected (N=X) | | All Subjects (N=X) | | Baseline HIV-infected (N=X) | | Baseline HIV-uninfected (N=X) | | All Subjects (N=X) | |
| | | | **n** | **%** | **n** | **%** | **n** | **%** | **n** | **%** | **n** | **%** | **n** | **%** | **n** | **%** | **n** | **%** | **n** | **%** |
| | Since last visit, was the subject treated or does the subject require retreatment for syphilis infection apart from study treatment received according to their randomization assignment? | Yes | x | x | x | x | x | x | x | x | x | x | x | x | x | x | x | x | x | x |
| | | No | x | x | x | x | x | x | x | x | x | x | x | x | x | x | x | x | x | x |
| | | Unknown | x | x | x | x | x | x | x | x | x | x | x | x | x | x | x | x | x | x |
| | Since last visit, if the subject was treated or require retreatment for syphilis infection apart from study treatment received according to their randomization, how was retreatment need determined? | Local RPR results | x | x | x | x | x | x | x | x | x | x | x | x | x | x | x | x | x | x |
| | | Known re-exposure | x | x | x | x | x | x | x | x | x | x | x | x | x | x | x | x | x | x |
| | | Return of or worsening symptoms | x | x | x | x | x | x | x | x | x | x | x | x | x | x | x | x | x | x |
| | | Other | x | x | x | x | x | x | x | x | x | x | x | x | x | x | x | x | x | x |
| Week 2 … | | | | | | | | | | | | | | | | | | | | |

Tables with similar format:

**Table 26: Summary of Categorical Sexual History through Month 12 by Baseline HIV Status and Treatment Group — ITT Population**

**Table 27: Summary of Categorical Sexual History through Month 6 by Baseline HIV Status and Treatment Group — Evaluable Population by Month 6**

**Table 28: Summary of Categorical Sexual History through Month 12 by Baseline HIV Status and Treatment Group — Evaluable Population by Month 12**

**Table 29: Summary of Continuous Sexual History through Month 6 by Baseline HIV Status and Treatment Group**

| Population | Time | Variable | Statistic | One Dose BPG 2.4 MU | | | Three Doses BPG 2.4 MU | | | All Subjects | | |
|---|---|---|---|---|---|---|---|---|---|---|---|---|
| | | | | HIV-infected (N=X) | HIV-uninfected (N=X) | All Subjects (N=X) | HIV-infected (N=X) | HIV-uninfected (N=X) | All Subjects (N=X) | HIV-infected (N=X) | HIV-uninfected (N=X) | All Subjects (N=X) |
| **ITT Population** | Week 1 | Total number of partners since last visit | Mean (SD) | x.x (x.x) | x.x (x.x) | x.x (x.x) | x.x (x.x) | x.x (x.x) | x.x (x.x) | x.x (x.x) | x.x (x.x) | x.x (x.x) |
| | | | Median | x.x | x.x | x.x | x.x | x.x | x.x | x.x | x.x | x.x |
| | | | Min, Max | x, x | x, x | x, x | x, x | x, x | x, x | x, x | x, x | x, x |
| | | Number of new partners since last visit | Mean (SD) | x.x (x.x) | x.x (x.x) | x.x (x.x) | x.x (x.x) | x.x (x.x) | x.x (x.x) | x.x (x.x) | x.x (x.x) | x.x (x.x) |
| | | | Median | x.x | x.x | x.x | x.x | x.x | x.x | x.x | x.x | x.x |
| | | | Min, Max | x, x | x, x | x, x | x, x | x, x | x, x | x, x | x, x | x, x |
| | | Number of continuing partners since last visit | Mean (SD) | x.x (x.x) | x.x (x.x) | x.x (x.x) | x.x (x.x) | x.x (x.x) | x.x (x.x) | x.x (x.x) | x.x (x.x) | x.x (x.x) |
| | | | Median | x.x | x.x | x.x | x.x | x.x | x.x | x.x | x.x | x.x |
| | | | Min, Max | x, x | x, x | x, x | x, x | x, x | x, x | x, x | x, x | x, x |
| | Week 2… | | | | | | | | | | | |
| **Evaluable Population by Month 6** | Week 1 | Total number of partners | Mean (SD) | x.x (x.x) | x.x (x.x) | x.x (x.x) | x.x (x.x) | x.x (x.x) | x.x (x.x) | x.x (x.x) | x.x (x.x) | x.x (x.x) |
| | | | Median | x.x | x.x | x.x | x.x | x.x | x.x | x.x | x.x | x.x |
| | | | Min, Max | x, x | x, x | x, x | x, x | x, x | x, x | x, x | x, x | x, x |
| | | Number of new partners | Mean (SD) | x.x (x.x) | x.x (x.x) | x.x (x.x) | x.x (x.x) | x.x (x.x) | x.x (x.x) | x.x (x.x) | x.x (x.x) | x.x (x.x) |
| | | | Median | x.x | x.x | x.x | x.x | x.x | x.x | x.x | x.x | x.x |
| | | | Min, Max | x, x | x, x | x, x | x, x | x, x | x, x | x, x | x, x | x, x |
| | | Number of continuing partners | Mean (SD) | x.x (x.x) | x.x (x.x) | x.x (x.x) | x.x (x.x) | x.x (x.x) | x.x (x.x) | x.x (x.x) | x.x (x.x) | x.x (x.x) |
| | | | Median | x.x | x.x | x.x | x.x | x.x | x.x | x.x | x.x | x.x |
| | | | Min, Max | x, x | x, x | x, x | x, x | x, x | x, x | x, x | x, x | x, x |
| | Week 2…. | | | | | | | | | | | |

Notes: N= Number of enrolled subjects in the specified baseline HIV status subgroup and analysis population.
Baseline is the Screening/Enrollment - Visit 1.
The numbers at each visit within the table specified period were totaled before deriving the summary statistics.

Tables with similar format:

**Table 30: Summary of Continuous Sexual History through Month 12 by Baseline HIV Status and Treatment Group**

**Table 31: Sensitivity analysis 1 - Serological Response to Therapy at Month 6 by Treatment Group – Removing Early Termination Due to COVID**

| Population | Treatment Group* | Number of Subjects with Serological Response, n | Number of Subjects, N | Proportion of Subjects with Serological Response (95% CI)[a] | Difference in Proportion of Subjects with Serological Response Between Two Treatment Groups (95% CI)[a] | P-Value |
|---|---|---|---|---|---|---|
| ITT Population | One Dose BPG 2.4 MU | xxx | xxx | 0.xx(0.xx, 0.xx) | 0.xx(0.xx, 0.xx) | 0.xxx |
| | Three Doses BPG 2.4 MU | xxx | xxx | 0.xx(0.xx, 0.xx) | | |
| | All Subjects | xxx | xxx | 0.xx(0.xx, 0.xx) | N/A | N/A |
| mITT Population | One Dose BPG 2.4 MU | xxx | xxx | 0.xx(0.xx, 0.xx) | 0.xx(0.xx, 0.xx) | 0.xxx |
| | Three Doses BPG 2.4 MU | xxx | xxx | 0.xx(0.xx, 0.xx) | | |
| | All Subjects | xxx | xxx | 0.xx(0.xx, 0.xx) | N/A | N/A |
| Evaluable Population | One Dose BPG 2.4 MU | xxx | xxx | 0.xx(0.xx, 0.xx) | 0.xx(0.xx, 0.xx) | 0.xxx |
| | Three Doses BPG 2.4 MU | xxx | xxx | 0.xx(0.xx, 0.xx) | | |
| | All Subjects | xxx | xxx | 0.xx(0.xx, 0.xx) | N/A | N/A |

Notes: The denominator for proportion is based on the number of subjects enrolled in the specified treatment group and analysis population.
[a] 95% CI= 95% Wilson Confidence Interval

**Table 32: Sensitivity analysis 2 - Serological Response to Therapy at Month 6 by Treatment Group – Re-defined Serological Response**

| Population | Treatment Group* | Number of Subjects with Serological Response, n | Number of Subjects, N | Proportion of Subjects with Serological Response (95% CI)[a] | Difference in Proportion of Subjects with Serological Response Between Two Treatment Groups (95% CI)[a] | P-Value |
|---|---|---|---|---|---|---|
| ITT Population | One Dose BPG 2.4 MU | xxx | xxx | 0.xx(0.xx, 0.xx) | 0.xx(0.xx, 0.xx) | 0.xxx |
| | Three Doses BPG 2.4 MU | xxx | xxx | 0.xx(0.xx, 0.xx) | | |
| | All Subjects | xxx | xxx | 0.xx(0.xx, 0.xx) | N/A | N/A |
| mITT Population | One Dose BPG 2.4 MU | xxx | xxx | 0.xx(0.xx, 0.xx) | 0.xx(0.xx, 0.xx) | 0.xxx |
| | Three Doses BPG 2.4 MU | xxx | xxx | 0.xx(0.xx, 0.xx) | | |
| | All Subjects | xxx | xxx | 0.xx(0.xx, 0.xx) | N/A | N/A |
| Evaluable Population | One Dose BPG 2.4 MU | xxx | xxx | 0.xx(0.xx, 0.xx) | 0.xx(0.xx, 0.xx) | 0.xxx |
| | Three Doses BPG 2.4 MU | xxx | xxx | 0.xx(0.xx, 0.xx) | | |
| | All Subjects | xxx | xxx | 0.xx(0.xx, 0.xx) | N/A | N/A |

Notes: The denominator for proportion is based on the number of subjects enrolled in the specified treatment group and analysis population.
[a] 95% CI= 95% Wilson Confidence Interval

**Table 33: Exploratory Analysis---Serological Response to Therapy by Month 6 by Treatment Group**

[Implementation Notes: Treatment group is the intended randomized group for ITT and mITT populations. Treatment group is the actual treatment a subject received for the Evaluable populations.]

| Population | Treatment Group | Number (Proportion) of Subjects with Serological Response[a] | Number (Proportion) of Subjects with Serological Non-Response[a] | Number (Proportion) of Subjects with Failure[a] Serological Response | Number of Subjects, N |
|---|---|---|---|---|---|
| ITT Population | One Dose BPG 2.4 MU | xxx (0.xx) | xxx (0.xx) | xxx (0.xx) | xxx |
| | Three Doses BPG 2.4 MU | xxx (0.xx) | xxx (0.xx) | xxx (0.xx) | xxx |
| | All Subjects | xxx (0.xx) | xxx (0.xx) | xxx (0.xx) | xxx |
| mITT Population | One Dose BPG 2.4 MU | xxx (0.xx) | xxx (0.xx) | xxx (0.xx) | xxx |
| | Three Doses BPG 2.4 MU | xxx (0.xx) | xxx (0.xx) | xxx (0.xx) | xxx |
| | All Subjects | xxx (0.xx) | xxx (0.xx) | xxx (0.xx) | xxx |
| Evaluable Population | One Dose BPG 2.4 MU | xxx (0.xx) | xxx (0.xx) | xxx (0.xx) | xxx |
| | Three Doses BPG 2.4 MU | xxx (0.xx) | xxx (0.xx) | xxx (0.xx) | xxx |
| | All Subjects | xxx (0.xx) | xxx (0.xx) | xxx (0.xx) | xxx |

Notes: The denominator for proportion is based on the number of subjects enrolled in the specified treatment group and analysis population.
95% CI= 95% Wilson Confidence Interval
[a] Alternative serological response classifications – Response: 4-fold decline in RPR titer compared to baseline, or RPR-negative (i.e., seroreversion); Non-Response: Less than 4-fold decline in RPR titer compared to baseline, and RPR-positive; Failure: 4-fold or greater increase in RPR titer without a clear history of re-exposure, and less than 4-fold decline in RPR titer at all visit compared to baseline.

**Table 34: Summary of Subjects with Pre-Existing Medical Conditions by MedDRA System Organ Class and Treatment Group - Safety Population**

| | One Dose BPG 2.4 MU (N = X) | | Three Doses BPG 2.4 MU (N = X) | | All Subjects (N=X) | |
|---|---|---|---|---|---|---|
| **MedDRA System Organ Class** | **n** | **%** | **n** | **%** | **n** | **%** |
| Any SOC | x | x | x | x | x | x |
| [SOC 1] | x | x | x | x | x | x |
| [SOC 2] | x | x | x | x | x | x |

Notes: N=number of subjects in the specified treatment group in the Safety Population.
n = Number of subjects reporting medical history within the specified SOC.
A subject is only counted once per SOC.

**Table 35: Number and Percentage of Subjects with Prior and Concomitant Medications by WHO Drug Classification and Treatment Group - Safety Population**

| WHO Drug Code Level 1, Anatomic Group | WHO Drug Code Level 2, Therapeutic Subgroup | One Dose BPG 2.4 MU (N = X) | | Three Doses BPG 2.4 MU (N = X) | | All Subjects (N=X) | |
|---|---|---|---|---|---|---|---|
| | | n | % | n | % | n | % |
| Any Level 1 Codes | Any Level 2 Codes | x | x | x | x | x | x |
| [ATC Level 1 - 1] | Any | x | x | x | x | x | x |
| | [ATC 2 - 1] | x | x | x | x | x | x |
| | [ATC 2 - 2] | x | x | x | x | x | x |
| | [ATC 2 - 3] | x | x | x | x | x | x |
| [ATC Level 1 – 2] | Any | x | x | x | x | x | x |
| | [ATC 2 - 1] | x | x | x | x | x | x |
| | [ATC 2 - 2] | x | x | x | x | x | x |
| | [ATC 2 - 3] | x | x | x | x | x | x |

Notes: N= Number of subjects in the specified treatment group and the Safety population
n=Number of subjects reporting taking at least one medication in the specific WHO Drug Class.

## Table 36: Overall Summary of Adverse Events - Safety Population

| | One Dose BPG 2.4 MU (N = X) | | Three Doses BPG 2.4 MU (N = X) | | All Subjects (N=X) | |
|---|---|---|---|---|---|---|
| Subjects[a] with | n | % | n | % | N | % |
| At least one local solicited adverse event | x | x | x | x | x | x |
| At least one systemic solicited adverse event | x | x | x | x | x | x |
| At least one unsolicited adverse event | x | x | x | x | x | x |
| At least one related unsolicited adverse event | x | x | x | x | x | x |
| Mild (Grade 1) | x | x | x | x | x | x |
| Moderate (Grade 2) | x | x | x | x | x | x |
| Severe (Grade 3) | x | x | x | x | x | x |
| Not yet assessed | | | | | | |
| At least one severe (Grade 3) unsolicited adverse event | x | x | x | x | x | x |
| Related | x | x | x | x | x | x |
| Unrelated | x | x | x | x | x | x |
| At least one serious adverse event[b] | x | x | x | x | x | x |
| At least one related, serious adverse event | x | x | x | x | x | x |
| At least one adverse event leading to early termination[c] | x | x | x | x | x | x |

Notes: N = Number of subjects in the Safety Population
[a] Subjects are counted once for each category regardless of the number of events.
[b] A listing of Serious Adverse Events is included in Table 47
[c] As reported on the Adverse Event eCRF.

**Table 37: Number and Percentage of Subjects Experiencing Solicited Events with 95% Confidence Intervals by Symptom and Treatment Group — Safety Population**

| | | One Dose BPG 2.4 MU (N = X) | | | Three Doses BPG 2.4 MU (N = X) | | | All Subjects (N=X) | | |
|---|---|---|---|---|---|---|---|---|---|---|
| **Category** | **Solicited Adverse Event** | **n** | **%** | **95% CI** | **n** | **%** | **95% CI** | **n** | **%** | **95% CI** |
| Solicited Adverse Events | Any Solicited Adverse Event | x | x | x.x, x.x | x | x | x.x, x.x | x | x | x.x, x.x |
| Solicited Local Adverse Events | Any Local Adverse Event | x | x | x.x, x.x | x | x | x.x, x.x | x | x | x.x, x.x |
| | Pain or tenderness | x | x | x.x, x.x | x | x | x.x, x.x | x | x | x.x, x.x |
| | Erythema or redness | x | x | x.x, x.x | x | x | x.x, x.x | x | x | x.x, x.x |
| | Induration or swelling | x | x | x.x, x.x | x | x | x.x, x.x | x | x | x.x, x.x |
| Solicited Systemic Adverse Events | Any Systemic Adverse Event (Jarisch-Herxheimer reaction) | x | x | x.x, x.x | x | x | x.x, x.x | x | x | x.x, x.x |
| | Feverishness | x | x | x.x, x.x | x | x | x.x, x.x | x | x | x.x, x.x |
| | Chills | x | x | x.x, x.x | x | x | x.x, x.x | x | x | x.x, x.x |
| | Myalgia | x | x | x.x, x.x | x | x | x.x, x.x | x | x | x.x, x.x |
| | Weakness | x | x | x.x, x.x | x | x | x.x, x.x | x | x | x.x, x.x |
| | Flushing | x | x | x.x, x.x | x | x | x.x, x.x | x | x | x.x, x.x |
| | Worsening of skin rash | x | x | x.x, x.x | x | x | x.x, x.x | x | x | x.x, x.x |
| | Tachycardia | x | x | x.x, x.x | x | x | x.x, x.x | x | x | x.x, x.x |
| | Heart palpitations | x | x | x.x, x.x | x | x | x.x, x.x | x | x | x.x, x.x |
| | Generalized arthralgia | x | x | x.x, x.x | x | x | x.x, x.x | x | x | x.x, x.x |
| | Nausea | x | x | x.x, x.x | x | x | x.x, x.x | x | x | x.x, x.x |
| | Headache | x | x | x.x, x.x | x | x | x.x, x.x | x | x | x.x, x.x |
| | Dizziness | x | x | x.x, x.x | x | x | x.x, x.x | x | x | x.x, x.x |

Notes: N = number of subjects in the specified treatment group in the Safety Population. 95% CI= 95% Wilson Confidence Interval
A subject is only counted once per solicited adverse event.

**Table 38: Number and Percentage of Subjects Experiencing Solicited Systemic Events by Symptom, Maximum Severity and Treatment Group — Safety Population**

| | | Severity | One Dose BPG 2.4 MU (N = X) n | One Dose BPG 2.4 MU (N = X) % | One Dose BPG 2.4 MU (N = X) 95% CI | Three Doses BPG 2.4 MU (N = X) n | Three Doses BPG 2.4 MU (N = X) % | Three Doses BPG 2.4 MU (N = X) 95% CI | All Subjects (N=X) n | All Subjects (N=X) % | All Subjects (N=X) 95% CI |
|---|---|---|---|---|---|---|---|---|---|---|---|
| Any Solicited Systemic Event | | Any severity | x | x | x,x | x | x | x,x | x | x | x,x |
| | | None | x | x | x,x | x | x | x,x | x | x | x,x |
| | | Mild | x | x | x,x | x | x | x,x | x | x | x,x |
| | | Moderate | x | x | x,x | x | x | x,x | x | x | x,x |
| | | Severe | x | x | x,x | x | x | x,x | x | x | x,x |
| Jarisch-Herxheimer Reaction | Feverishness | Any severity | x | x | x,x | x | x | x,x | x | x | x,x |
| | | None | x | x | x,x | x | x | x,x | x | x | x,x |
| | | Mild | x | x | x,x | x | x | x,x | x | x | x,x |
| | | Moderate | x | x | x,x | x | x | x,x | x | x | x,x |
| | | Severe | x | x | x,x | x | x | x,x | x | x | x,x |
| | Chills | Any severity | x | x | x,x | x | x | x,x | x | x | x,x |
| | | None | x | x | x,x | x | x | x,x | x | x | x,x |
| | | Mild | x | x | x,x | x | x | x,x | x | x | x,x |
| | | Moderate | x | x | x,x | x | x | x,x | x | x | x,x |
| | | Severe | x | x | x,x | x | x | x,x | x | x | x,x |
| | Myalgia | Any severity | x | x | x,x | x | x | x,x | x | x | x,x |
| | | None | x | x | x,x | x | x | x,x | x | x | x,x |
| | | Mild | x | x | x,x | x | x | x,x | x | x | x,x |
| | | Moderate | x | x | x,x | x | x | x,x | x | x | x,x |
| | | Severe | x | x | x,x | x | x | x,x | x | x | x,x |

| | | Severity | One Dose BPG 2.4 MU (N = X) n | % | 95% CI | Three Doses BPG 2.4 MU (N = X) n | % | 95% CI | All Subjects (N=X) n | % | 95% CI |
|---|---|---|---|---|---|---|---|---|---|---|---|
| | Weakness | Any severity | x | x | x,x | x | x | x,x | x | x | x,x |
| | | None | x | x | x,x | x | x | x,x | x | x | x,x |
| | | Mild | x | x | x,x | x | x | x,x | x | x | x,x |
| | | Moderate | x | x | x,x | x | x | x,x | x | x | x,x |
| | | Severe | x | x | x,x | x | x | x,x | x | x | x,x |
| | Flushing | Any severity | x | x | x,x | x | x | x,x | x | x | x,x |
| | | None | x | x | x,x | x | x | x,x | x | x | x,x |
| | | Mild | x | x | x,x | x | x | x,x | x | x | x,x |
| | | Moderate | x | x | x,x | x | x | x,x | x | x | x,x |
| | | Severe | x | x | x,x | x | x | x,x | x | x | x,x |
| | Worsening of skin rash | Any severity | x | x | x,x | x | x | x,x | x | x | x,x |
| | | None | x | x | x,x | x | x | x,x | x | x | x,x |
| | | Mild | x | x | x,x | x | x | x,x | x | x | x,x |
| | | Moderate | x | x | x,x | x | x | x,x | x | x | x,x |
| | | Severe | x | x | x,x | x | x | x,x | x | x | x,x |
| | Tachycardia | Any severity | x | x | x,x | x | x | x,x | x | x | x,x |
| | | None | x | x | x,x | x | x | x,x | x | x | x,x |
| | | Mild | x | x | x,x | x | x | x,x | x | x | x,x |
| | | Moderate | x | x | x,x | x | x | x,x | x | x | x,x |
| | | Severe | x | x | x,x | x | x | x,x | x | x | x,x |
| | Heart palpitations | Any severity | x | x | x,x | x | x | x,x | x | x | x,x |
| | | None | x | x | x,x | x | x | x,x | x | x | x,x |
| | | Mild | x | x | x,x | x | x | x,x | x | x | x,x |
| | | Moderate | x | x | x,x | x | x | x,x | x | x | x,x |
| | | Severe | x | x | x,x | x | x | x,x | x | x | x,x |

| | | Severity | One Dose BPG 2.4 MU (N = X) | | | Three Doses BPG 2.4 MU (N = X) | | | All Subjects (N=X) | | |
|---|---|---|---|---|---|---|---|---|---|---|---|
| | | | n | % | 95% CI | n | % | 95% CI | n | % | 95% CI |
| | Generalized arthralgia | Any severity | x | x | x,x | x | x | x,x | x | x | x,x |
| | | None | x | x | x,x | x | x | x,x | x | x | x,x |
| | | Mild | x | x | x,x | x | x | x,x | x | x | x,x |
| | | Moderate | x | x | x,x | x | x | x,x | x | x | x,x |
| | | Severe | x | x | x,x | x | x | x,x | x | x | x,x |
| | Nausea | Any severity | x | x | x,x | x | x | x,x | x | x | x,x |
| | | None | x | x | x,x | x | x | x,x | x | x | x,x |
| | | Mild | x | x | x,x | x | x | x,x | x | x | x,x |
| | | Moderate | x | x | x,x | x | x | x,x | x | x | x,x |
| | | Severe | x | x | x,x | x | x | x,x | x | x | x,x |
| | Headache | Any severity | x | x | x,x | x | x | x,x | x | x | x,x |
| | | None | x | x | x,x | x | x | x,x | x | x | x,x |
| | | Mild | x | x | x,x | x | x | x,x | x | x | x,x |
| | | Moderate | x | x | x,x | x | x | x,x | x | x | x,x |
| | | Severe | x | x | x,x | x | x | x,x | x | x | x,x |
| | Dizziness | Any severity | x | x | x,x | x | x | x,x | x | x | x,x |
| | | None | x | x | x,x | x | x | x,x | x | x | x,x |
| | | Mild | x | x | x,x | x | x | x,x | x | x | x,x |
| | | Moderate | x | x | x,x | x | x | x,x | x | x | x,x |
| | | Severe | x | x | x,x | x | x | x,x | x | x | x,x |

Note: Denominator for percentages is the number of subjects in the specified treatment group in the Safety Population with solicited adverse event data available after the first dose of study product. 95% CI= 95% Wilson Confidence Interval

**Table 39: Number and Percentage of Subjects Experiencing Solicited Local Events with 95% Confidence Intervals by Symptom, Maximum Severity, Dose, and Treatment Group — Safety Population**

[Implementation note applies to all tables: If the table will be multi-page, move the footnote/explanation to the footer so that it repeats for each page of the table.]

[Implementation note: Post each dose, use whichever is more severe between most severe response among days 1-6 and the maximum severity/measurement after day 6 from ZRL.]

| | | Post Dose 1 [One Dose BPG 2.4 MU Group] (N=X) | | | Post Dose 1 [Three Doses BPG 2.4 MU Group] (N=X) | | | Post Dose 2 [Three Doses BPG 2.4 MU Group] (N=X) | | | Post Dose 3 [Three Doses BPG 2.4 MU Group] (N=X) | | | Post Any Dose [Three Doses BPG 2.4 MU Group] (N=X) | | | Overall (N=X) | | |
|---|---|---|---|---|---|---|---|---|---|---|---|---|---|---|---|---|---|---|---|
| **Solicited Adverse Event** | **Severity[a]** | **n** | **%** | **95% CI** | **n** | **%** | **95% CI** | **n** | **%** | **95% CI** | **n** | **%** | **95% CI** | **n** | **%** | **95% CI** | **n** | **%** | **95% CI** |
| Any Solicited Local Event | Any Severity | x | x | x,x | x | x | x,x | x | x | x,x | x | x | x,x | x | x | x,x | x | x | x,x |
| | None | x | x | x,x | x | x | x,x | x | x | x,x | x | x | x,x | x | x | x,x | x | x | x,x |
| | Mild | x | x | x,x | x | x | x,x | x | x | x,x | x | x | x,x | x | x | x,x | x | x | x,x |
| | Moderate | x | x | x,x | x | x | x,x | x | x | x,x | x | x | x,x | x | x | x,x | x | x | x,x |
| | Severe | x | x | x,x | x | x | x,x | x | x | x,x | x | x | x,x | x | x | x,x | x | x | x,x |
| Pain or tenderness | Any Severity | x | x | x,x | x | x | x,x | x | x | x,x | x | x | x,x | x | x | x,x | x | x | x,x |
| | None | x | x | x,x | x | x | x,x | x | x | x,x | x | x | x,x | x | x | x,x | x | x | x,x |
| | Mild | x | x | x,x | x | x | x,x | x | x | x,x | x | x | x,x | x | x | x,x | x | x | x,x |
| | Moderate | x | x | x,x | x | x | x,x | x | x | x,x | x | x | x,x | x | x | x,x | x | x | x,x |
| | Severe | x | x | x,x | x | x | x,x | x | x | x,x | x | x | x,x | x | x | x,x | x | x | x,x |
| Erythema or redness | Any Severity | x | x | x,x | x | x | x,x | x | x | x,x | x | x | x,x | x | x | x,x | x | x | x,x |
| | None | x | x | x,x | x | x | x,x | x | x | x,x | x | x | x,x | x | x | x,x | x | x | x,x |
| | Mild | x | x | x,x | x | x | x,x | x | x | x,x | x | x | x,x | x | x | x,x | x | x | x,x |
| | Moderate | x | x | x,x | x | x | x,x | x | x | x,x | x | x | x,x | x | x | x,x | x | x | x,x |
| | Severe | x | x | x,x | x | x | x,x | x | x | x,x | x | x | x,x | x | x | x,x | x | x | x,x |

| Solicited Adverse Event | Severity[a] | Post Dose 1 [One Dose BPG 2.4 MU Group] (N=X) | | | Post Dose 1 [Three Doses BPG 2.4 MU Group] (N=X) | | | Post Dose 2 [Three Doses BPG 2.4 MU Group] (N=X) | | | Post Dose 3 [Three Doses BPG 2.4 MU Group] (N=X) | | | Post Any Dose [Three Doses BPG 2.4 MU Group] (N=X) | | | Overall (N=X) | | |
|---|---|---|---|---|---|---|---|---|---|---|---|---|---|---|---|---|---|---|---|
| | | n | % | 95% CI | n | % | 95% CI | n | % | 95% CI | n | % | 95% CI | n | % | 95% CI | n | % | 95% CI |
| Induration or swelling | Any Severity | x | x | x,x | x | x | x,x | x | x | x,x | x | x | x,x | x | x | x,x | x | x | x,x |
| | None | x | x | x,x | x | x | x,x | x | x | x,x | x | x | x,x | x | x | x,x | x | x | x,x |
| | Mild | x | x | x,x | x | x | x,x | x | x | x,x | x | x | x,x | x | x | x,x | x | x | x,x |
| | Moderate | x | x | x,x | x | x | x,x | x | x | x,x | x | x | x,x | x | x | x,x | x | x | x,x |
| | Severe | x | x | x,x | x | x | x,x | x | x | x,x | x | x | x,x | x | x | x,x | x | x | x,x |

Notes: Denominator for percentages is the number of subjects in the specified treatment group and the Safety Population with solicited adverse event data available after the first dose of study product
95% CI= 95% Wilson Confidence Interval
[a] Each subject's maximum severity is reported for each solicited adverse event across all doses.

**Table 40: The Association of Treatment with Jarisch-Herxheimer Reaction after Adjusting for HIV Co-infection status and Jarisch-Herxheimer Reaction (JHR) Onset Time Post-dose by Logistic Regression - Safety Population**

| Risk Factors | Odds Ratio [a] | P-Value | Adjusted Odds Ratio[b] | 95% Wald CIs | P-Value |
|---|---|---|---|---|---|
| Three Doses BPG 2.4 MU Group vs One Dose BPG 2.4 MU Group (ref) | x.x | x.xxx | x.x | x,x | x.xxx |
| Baseline HIV-infected vs Baseline HIV-uninfected (ref) | x.x | x.xxx | x.x | x,x | x.xxx |
| JHR Onset Time Post-dose: 0-12 hours vs >24-48 hours (ref) | x.x | x.xxx | x.x | x,x | x.xxx |
| JHR Onset Time Post-dose: >12-24 hours vs >24-48 hours (ref) | x.x | x.xxx | x.x | x,x | x.xxx |

Note: P-value is from corresponding the logistic regression model for the variable.
[a] Odds ratio is from univariate models;
[b] Odds ratio is from the adjusted logistic model


PRIVILEGED AND CONFIDENTIAL COMMUNICATION

**Table 41: Number and Percentage of Subjects Experiencing Unsolicited Adverse Events with 95% Confidence Intervals by MedDRA System Organ Class and Preferred Term, Dose Number, and Treatment Group — Safety Population**

| | | Post Dose 1 [One Dose BPG 2.4 MU Group] (N = X) | | | Post Dose 1 [Three Doses BPG 2.4 MU Group] (N = X) | | | Post Dose 2 [Three Doses BPG 2.4 MU Group] (N = X) | | | Post Dose 3 [Three Doses BPG 2.4 MU Group] (N = X) | | | Post Any Dose [Three Doses BPG 2.4 MU Group] (N = X) | | | Overall (N = X) | | |
|---|---|---|---|---|---|---|---|---|---|---|---|---|---|---|---|---|---|---|---|
| **MedDRA System Organ Class** | **MedDRA Preferred Term** | **n** | **%** | **95% CI** | **n** | **%** | **95% CI** | **n** | **%** | **95% CI** | **n** | **%** | **95% CI** | **n** | **%** | **95% CI** | **n** | **%** | **95% CI** |
| Any SOC | Any PT | x | x | x, x | x | x | x, x | x | x | x, x | x | x | x, x | x | x | x, x | x | X | x, x |
| [SOC 1] | Any PT | x | x | x, x | x | x | x, x | x | x | x, x | x | x | x, x | x | x | x, x | x | X | x, x |
| | [PT 1] | x | x | x, x | x | x | x, x | x | x | x, x | x | x | x, x | x | x | x, x | x | X | x, x |
| | [PT 2] | x | x | x, x | x | x | x, x | x | x | x, x | x | x | x, x | x | x | x, x | x | X | x, x |

Notes: N = number of subjects in the specified treatment group and the Safety Population
This table presents number and percentage of subjects. 95% CI= 95% Wilson Confidence Interval
For each timepoint, a subject is only counted once per PT. For a subject who had an AE ongoing through multiple doses, do we count the subject once at the earliest dose when the AE started? That is, AE ongoing from post dose 1 to post dose 2, we'll count the subject once in the post dose 1 column, and not in the post dose 2 column.

Table with similar format:

**Table 42: Number and Percentage of Subjects Experiencing Related Unsolicited Adverse Events with 95% Confidence Intervals by MedDRA System Organ Class and Preferred Term, Dose Number, and Treatment Group — Safety Population**

**Table 43: Number of Unsolicited Adverse Events by MedDRA System Organ Class and Preferred Term, Maximum Severity and Relationship, and Treatment Group — Safety Population**

[Implementation note: check if there were any Not Yet Determined events, report if yes.]

| MedDRA SOC | Preferred Term | Severity | One Dose BPG 2.4 MU (n = X) | | | Three Doses BPG 2.4 MU (n = X) | | | All Subjects (n = X) | | |
|---|---|---|---|---|---|---|---|---|---|---|---|
| | | | Related | Not Related | Total | Related | Not Related | Total | Related | Not Related | Total |
| Any SOC | Any PT | Any Severity | xx | xx | xx | xx | xx | xx | xx | xx | xx |
| | | Mild | xx | xx | xx | xx | xx | xx | xx | xx | xx |
| | | Moderate | xx | xx | xx | xx | xx | xx | xx | xx | xx |
| | | Severe | xx | xx | xx | xx | xx | xx | xx | xx | xx |
| SOC 1 | PT 1 | Any Severity | xx | xx | xx | xx | xx | xx | xx | xx | xx |
| | | Mild | xx | xx | xx | xx | xx | xx | xx | xx | xx |
| | | Moderate | xx | xx | xx | xx | xx | xx | xx | xx | xx |
| | | Severe | xx | xx | xx | xx | xx | xx | xx | xx | xx |
| | PT 2 | Any Severity | xx | xx | xx | xx | xx | xx | xx | xx | xx |
| | | Mild | xx | xx | xx | xx | xx | xx | xx | xx | xx |
| | | Moderate | xx | xx | xx | xx | xx | xx | xx | xx | xx |
| | | Severe | xx | xx | xx | xx | xx | xx | xx | xx | xx |

Note: n = Number of events for subjects in the Safety Population.

**Table 44: Number and Percentage of Subjects Experiencing Unsolicited Adverse Events by MedDRA System Organ Class and Preferred Term, Maximum Severity and Relationship, and Treatment Group — Safety Population**

| MedDRA System Organ Class | Preferred Term | Severity | One Dose BPG 2.4 MU (N = X) | | | | | | Three Doses BPG 2.4 MU (N = X) | | | | | | All Subjects (N = X) | | | | | |
|---|---|---|---|---|---|---|---|---|---|---|---|---|---|---|---|---|---|---|---|---|
| | | | Related | | Not Related | | Total | | Related | | Not Related | | Total | | Related | | Not Related | | Total | |
| | | | n | % | n | % | n | % | n | % | n | % | n | % | n | % | n | % | n | % |
| Any SOC | Any PT | Any Severity | x | xx | x | xx | x | xx | x | xx | x | xx | x | xx | x | xx | x | xx | x | xx |
| | | Mild | x | xx | x | xx | x | xx | x | xx | x | xx | x | xx | x | xx | x | xx | x | xx |
| | | Moderate | x | xx | x | xx | x | xx | x | xx | x | xx | x | xx | x | xx | x | xx | x | xx |
| | | Severe | x | xx | x | xx | x | xx | x | xx | x | xx | x | xx | x | xx | x | xx | x | xx |
| SOC 1 | PT 1 | Any Severity | x | xx | x | xx | x | xx | x | xx | x | xx | x | xx | x | xx | x | xx | x | xx |
| | | Mild | x | xx | x | xx | x | xx | x | xx | x | xx | x | xx | x | xx | x | xx | x | xx |
| | | Moderate | x | xx | x | xx | x | xx | x | xx | x | xx | x | xx | x | xx | x | xx | x | xx |
| | | Severe | x | xx | x | xx | x | xx | x | xx | x | xx | x | xx | x | xx | x | xx | x | xx |
| | PT 2 | Any Severity | x | xx | x | xx | x | xx | x | xx | x | xx | x | xx | x | xx | x | xx | x | xx |
| | | Mild | x | xx | x | xx | x | xx | x | xx | x | xx | x | xx | x | xx | x | xx | x | xx |
| | | Moderate | x | xx | x | xx | x | xx | x | xx | x | xx | x | xx | x | xx | x | xx | x | xx |
| | | Severe | x | xx | x | xx | x | xx | x | xx | x | xx | x | xx | x | xx | x | xx | x | xx |

Notes: N = Number of subjects in the Safety Population.
For severity, a subject is counted once per preferred term and is summarized according to their highest severity.


PRIVILEGED AND CONFIDENTIAL COMMUNICATION

**Table 45: Unsolicited Adverse Events Occurring in ≥5% of Subjects in Any Treatment Group by MedDRA System Organ Class, Preferred Term, and Treatment Group - Safety Population**

| Preferred Term | MedDRA System Organ Class | One Dose BPG 2.4 MU (N = X) | | | Three Doses BPG 2.4 MU (N = X) | | | All Subjects (N=X) | | |
|---|---|---|---|---|---|---|---|---|---|---|
| | | n | % | Events | n | % | Events | n | % | Events |
| Serious Adverse Events | | | | | | | | | | |
| All | All | x | x | x | x | x | x | x | X | x |
| PT1 | SOC1 | x | x | x | x | x | x | x | X | x |
| Etc. | Etc. | x | x | x | x | x | x | x | X | x |
| Non-serious Adverse Events | | | | | | | | | | |
| All | All | x | x | x | x | x | x | x | X | x |
| PT1 | SOC1 | x | x | x | x | x | x | x | X | x |
| Etc | Etc | x | x | x | x | x | x | x | X | x |

N = number of subjects in the specified treatment group and the Safety Population
n= number of subjects reporting event.
Events= total frequency of events reported.

Table with similar format:

**Table 46: Solicited Adverse Events Occurring in ≥5% of Subjects in Any Treatment Group by MedDRA System Organ Class, Preferred Term, and Treatment Group - Safety Population**

**Table 47: Listing of Serious Adverse Events – All Enrolled Subjects**

[Implementation Notes: If the event is ongoing (no stop date), indicate "ongoing" in the "Duration" column. In the "If Not Related, Alternate Etiology" column, merge the 2 data fields for collecting alternate etiology, separate by a colon. If there are no comments for an event, populate 'Comments' row with 'None'. In the CSR, Subject ID should be USUBJID (not PATID) for purposes of de-identification. Listing should be sorted by treatment group, Subject ID, AE Number.

Show footnote if the following scenario exist: Notes: Subjects are excluded from Safety Population if it is unknown whether they received at least one dose, but the adverse events of such subjects are included in the AE listings]

| Adverse Event | Associated with Dose No. | No. of Days Post Associated Dose | Duration (Days) | Severity | SAE? | Relationship to Study Treatment | In Not Related, Alternative Etiology | Action Taken with Study Treatment | Subject Discontinued Due to AE | Outcome | MedDRA System Organ Class | MedDRA Preferred Term |
|---|---|---|---|---|---|---|---|---|---|---|---|---|
| Treatment Group: , Subject ID: , AE Number: | | | | | | | | | | | | |
| xxxxxxx | xx | x | x | xxxxxx | xxxxxx | xxxxxx | xxxxx | xxxxxx | Y/N | xxxxx | xxxxx | xxxxx |
| Comments: xxxxxxxxxxxxx | | | | | | | | | | | | |
| | | | | | | | | | | | | |
| Treatment Group: , Subject ID: , AE Number: | | | | | | | | | | | | |
| xxxxxxx | xx | x | x | xxxxxx | xxxxxx | xxxxxx | xxxxx | xxxxxx | Y/N | xxxxx | Xxxxx | xxxxx |
| Comments: xxxxxxxxxxxxx | | | | | | | | | | | | |

**Table 48: Listing of Non-Serious, Unsolicited Adverse Events – All Enrolled Subjects**

[Implementation Notes: If the event is ongoing (no stop date), indicate "ongoing" in the "Duration" column. In the "If Not Related, Alternate Etiology" column, merge the 2 data fields for collecting alternate etiology, separate by a colon. If there are no comments for an event, populate 'Comments' row with 'None'. In the CSR, Subject ID should be USUBJID (not PATID) for purposes of de-identification. Listing should be sorted by Treatment Group, Subject ID, AE Number.

Show footnote if the following scenario exist: Notes: Subjects are excluded from Safety Population if it is unknown whether they received at least one dose, but the adverse events of such subjects are included in the AE listings.]

| Adverse Event | Associated with Dose No. | No. of Days Post Associated Dose | Duration (Days) | Severity | SAE? | Relationship to Study Treatment | In Not Related, Alternative Etiology | Action Taken with Study Treatment | Subject Discontinued Due to AE | Outcome | MedDRA System Organ Class | MedDRA Preferred Term |
|---|---|---|---|---|---|---|---|---|---|---|---|---|
| Treatment Group: , Subject ID: , AE Number: | | | | | | | | | | | | |
| xxxxxxx | xx | x | x | xxxxxx | xxxxxx | xxxxxx | xxxxx | xxxxxx | Y/N | xxxxx | xxxxx | xxxxx |
| Comments: xxxxxxxxxxxxx | | | | | | | | | | | | |
| | | | | | | | | | | | | |
| Treatment Group: , Subject ID: , AE Number: | | | | | | | | | | | | |
| xxxxxxx | xx | x | x | xxxxxx | xxxxxx | xxxxxx | xxxxx | xxxxxx | Y/N | xxxxx | Xxxxx | xxxxx |
| Comments: xxxxxxxxxxxxx | | | | | | | | | | | | |

**Narratives of Deaths, Other Serious and Significant Adverse Events**

(not included in SAP, but this is a placeholder for the CSR)

**Table 49: Laboratory Summary Statistics by Time Point and Treatment Group – CD4 Count in HIV-infected Subjects**

[Implementation notes:

1. For calculated fields (Mean, SD, Median), decimal place should be the format in which the data were collected + 1 extra place. For Min, Max, decimal place should be in the same format in which the data were collected.

2. If there are test results at interim visits, e.g., 06S, 06T, include them in the corresponding scheduled visit under "Time Point" column, e.g., Visit 6.]

| Time Point | Variable | Treatment Group | N | Mean | Standard Deviation | Median | Min, Max |
|---|---|---|---|---|---|---|---|
| Visit 1 (Day 1) | CD4 count | One Dose BPG 2.4 MU | x | xx.x | xx.x | xx.x | xx.x, xx.x |
| | | Three Doses BPG 2.4 MU | | | | | |
| Visit 4 (Day 30±7 days) | CD4 count | One Dose BPG 2.4 MU | | | | | |
| | | Three Doses BPG 2.4 MU | | | | | |
| | Change from Baseline | One Dose BPG 2.4 MU | | | | | |
| | | Three Doses BPG 2.4 MU | | | | | |
| Visit 6 (Day 180±21 days) | CD4 count | One Dose BPG 2.4 MU | | | | | |
| | | Three Doses BPG 2.4 MU | | | | | |
| | Change from Baseline | One Dose BPG 2.4 MU | | | | | |
| | | Three Doses BPG 2.4 MU | | | | | |
| Visit 8 (Day 360±28 days) | CD4 count | One Dose BPG 2.4 MU | | | | | |
| | | Three Doses BPG 2.4 MU | | | | | |
| | Change from Baseline | One Dose BPG 2.4 MU | | | | | |
| | | Three Doses BPG 2.4 MU | | | | | |

Note: N = Number of subjects in the Safety Population who had CD4 Counts at the specified time point. Test results between scheduled visits are included in the most recent visits.

**Table 50: Laboratory Summary Statistics by Time Point and Treatment Group – Viral Load in HIV-infected Subjects**

[Implementation notes:

1. For calculated fields (Mean, SD, Median), decimal place should be the format in which the data were collected + 1 extra place. For Min, Max, decimal place should be in the same format in which the data were collected.

2. If there are test results at interim visits, e.g., 06S, 06T, include them in the corresponding scheduled visit under "Time Point" column, e.g., Visit 6.]

| Time Point | Treatment Group | N | Mean | Standard Deviation | Median | Min, Max |
|---|---|---|---|---|---|---|
| **Detectable Viral Load** | | | | | | |
| Visit 1<br>(Day 1) | One Dose BPG 2.4 MU | x | xx.x | xx.x | xx.x | xx.x, xx.x |
| | Three Doses BPG 2.4 MU | | | | | |
| Visit 2<br>(Day 7-13days) | One Dose BPG 2.4 MU | | | | | |
| | Three Doses BPG 2.4 MU | | | | | |
| Visit 3<br>(Day 13±23 days) | One Dose BPG 2.4 MU | | | | | |
| | Three Doses BPG 2.4 MU | | | | | |
| Visit 4<br>(Day 30±7 days) | One Dose BPG 2.4 MU | | | | | |
| | Three Doses BPG 2.4 MU | | | | | |

Notes: N = Number of subjects in the Safety Population who had CD4 Counts at the specified time point.

**Table 51: Vital Signs by Maximum Severity, Time Point, and Treatment Group – Any Assessment – Safety Population**

[Implementation Note: Generate one table for “Any Assessment” and one table for each assessment as listed below.]

| Time Point | Treatment Group | N | None | | Mild | | Moderate | | Severe | | Missing | |
|---|---|---|---|---|---|---|---|---|---|---|---|---|
| | | | n | % | n | % | n | % | n | % | N | % |
| Visit 1 | One Dose BPG 2.4 MU | x | x | xx | x | xx | x | xx | x | xx | X | xx |
| | Three Doses BPG 2.4 MU | | | | | | | | | | | |
| … | | | | | | | | | | | | |
| Visit 8 | One Dose BPG 2.4 MU | | | | | | | | | | | |
| | Three Doses BPG 2.4 MU | | | | | | | | | | | |
| Max Severity Post Baseline | One Dose BPG 2.4 MU | | | | | | | | | | | |
| | Three Doses BPG 2.4 MU | | | | | | | | | | | |

Notes: The “Max Severity Post Baseline” row indicate the maximum severity experienced by each subject at any time point post baseline, including unscheduled assessments. N = Number of subjects in the Safety Population.
Grading scales:
Temperature: Grade 1: 38.0 - <38.6°C; Grade 2: 38.6 - <39.3°C; Grade 3: ≥39.3°C
Blood Pressure: Grade 1: 140 – 159 mmHg systolic OR 90 – 99 mmHg diastolic; Grade 2: 160 – 179 mmHg systolic OR 100 – 109 mmHg diastolic; Grade 3: ≥180 mmHg systolic OR ≥110 mmHg diastolic OR hospitalization indicated
Pulse: Grade 1: 101-115 or 50-54 or 45-50 if baseline <60bpm; Grade 2: 116-130 or 45-49 or 40-44 if baseline <60bpm; Grade 3: >130 or ventricular dysrhythmias or <45 or <40 if baseline <60bpm
Respiration: Grade 1: 23-25; Grade 2: 26-30; Grade 3: >30

Tables with similar format:

**Table 52: Vital Signs by Maximum Severity, Time Point, and Treatment Group – Temperature – Safety Population**

**Table 53: Vital Signs by Maximum Severity, Time Point, and Treatment Group – Blood Pressure – Safety Population**

**Table 54: Vital Signs by Maximum Severity, Time Point, and Treatment Group – Pulse – Safety Population**

**Table 55: Vital Signs by Maximum Severity, Time Point, and Treatment Group – Respiration – Safety Population**

# APPENDIX 2. FIGURE MOCK-UPS

## LIST OF FIGURES

Figure 1: CONSORT Flow Diagram ....97
Figure 2: Rapid Plasma Reagin Titer per Subject by Month 6 -ITT population....98
Figure 3: Rapid Plasma Reagin Titer per Subject by Month 6 -mITT population....98
Figure 4: Rapid Plasma Reagin Titer per Subject by Month 6 -Evaluable population....98
Figure 5: Rapid Plasma Reagin Titer per Subject by Month 12 -ITT population....98
Figure 6: Rapid Plasma Reagin Titer per Subject by Month 12 -Evaluable population....98
Figure 7: Rapid Plasma Reagin Titer per Subject by Treatment and Baseline HIV Status by Month 6 -ITT population....98
Figure 8: Rapid Plasma Reagin Titer per Subject by Treatment and Baseline HIV Status by Month 6 -Evaluable population....98
Figure 9: Rapid Plasma Reagin Titer per Subject by Treatment and Baseline HIV Status by Month 12 -ITT population....98
Figure 10: Rapid Plasma Reagin Titer per Subject by Treatment and Baseline HIV Status by Month 12 -Evaluable population....98
Figure 11: Difference in Proportion of Subjects with Serological Response Between Two Treatment Groups by Month 6....99
Figure 12: Difference in Proportion of Subjects with Serological Response Between Two Treatment Groups by Month 12....99
Figure 13: Maximum Severity of Jarisch-Herxheimer reaction per Subject by Time Post First Dose - Safety Population....100
Figure 14: Maximum Severity of Solicited Local Symptoms per Subject by Dose - Safety Population....101
Figure 15: Number of Related Unsolicited Adverse Events by MedDRA System Organ Class and Severity - Safety Population....102
Figure 16: Number and Percentage of Subjects Experiencing Related Unsolicited Adverse Events by MedDRA® System Organ Class and Maximum Severity - Safety Population....103
Figure 17: Number of Unsolicited Adverse Events by MedDRA System Organ Class and Relationship to Treatment - Safety Population....104
Figure 18: Number and Percentage of Subjects Experiencing Unsolicited Adverse Events by MedDRA System Organ Class and Relationship to Treatment - Safety Population....105

**Figure 1: CONSORT Flow Diagram**

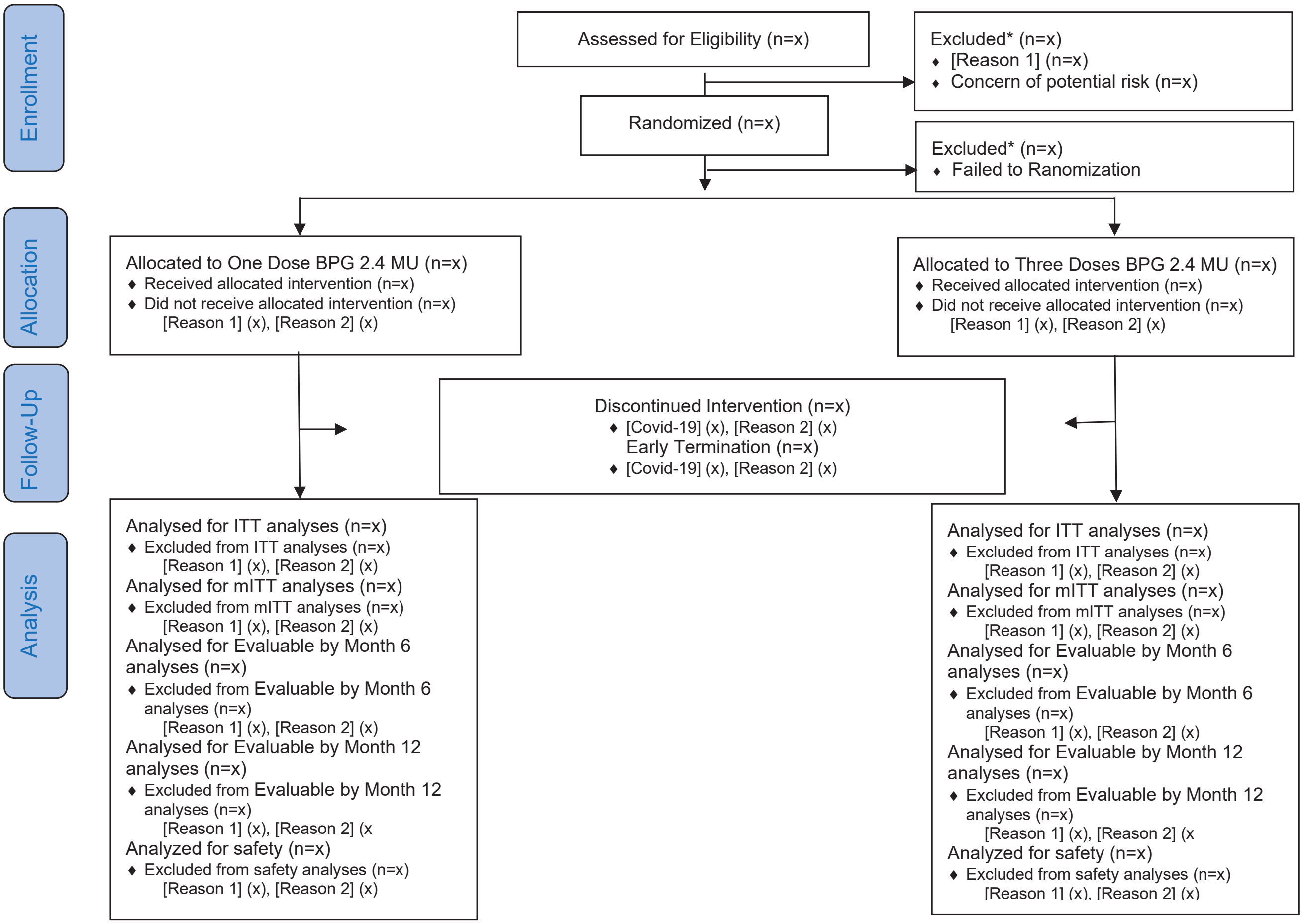

**Figure 2: Rapid Plasma Reagin Titer per Subject by Month 6 -ITT population**

[Implementation Notes: this is a generic plot

- Change the "Visits" (X axis) into "Time", then label the X-axis by "Baseline", "Week 1" "Week 2", "Month 1", "Month 3", and "Month 6".
- The dash line is the RPR titer for each subject at each timepoint;
- The black line is the Geometric Mean Titer at each time point
- Change the Group names to the treatment arms.
- Color can be changed]

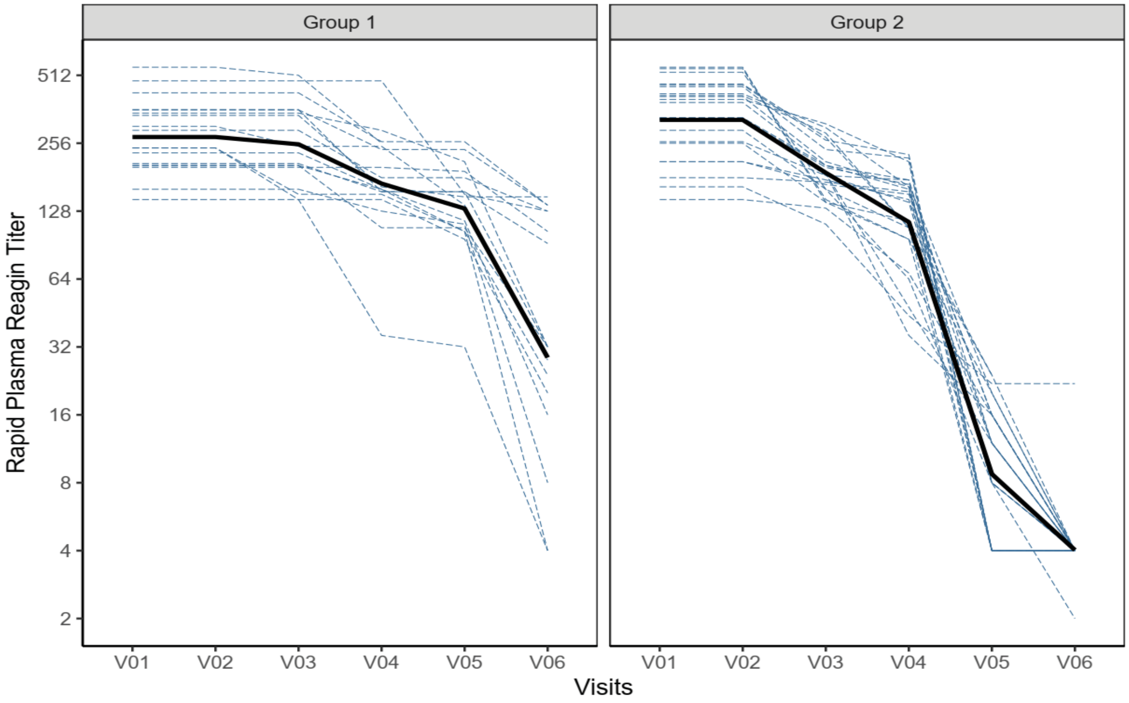


Figures with a similar format:

**Figure 3: Rapid Plasma Reagin Titer per Subject by Month 6 -mITT population**

**Figure 4: Rapid Plasma Reagin Titer per Subject by Month 6 -Evaluable population**

**Figure 5: Rapid Plasma Reagin Titer per Subject by Month 12 -ITT population**

**Figure 6: Rapid Plasma Reagin Titer per Subject by Month 12 -Evaluable population**

**Figure 7: Rapid Plasma Reagin Titer per Subject by Treatment and Baseline HIV Status by Month 6 -ITT population**

**Figure 8: Rapid Plasma Reagin Titer per Subject by Treatment and Baseline HIV Status by Month 6 -Evaluable population**

**Figure 9: Rapid Plasma Reagin Titer per Subject by Treatment and Baseline HIV Status by Month 12 -ITT population**

**Figure 10: Rapid Plasma Reagin Titer per Subject by Treatment and Baseline HIV Status by Month 12 -Evaluable population**


PRIVILEGED AND CONFIDENTIAL COMMUNICATION

**Figure 11: Difference in Proportion of Subjects with Serological Response Between Two Treatment Groups by Month 6**

[Implementation Notes: this is a generic plot,

The dark block is the estimates of difference of proportion of serological response between three dose vs one dose; the horizontal line and two bars are the one-sided 95% confidence interval;

The dash vertical line is the non-inferiority margin 0.10. ]

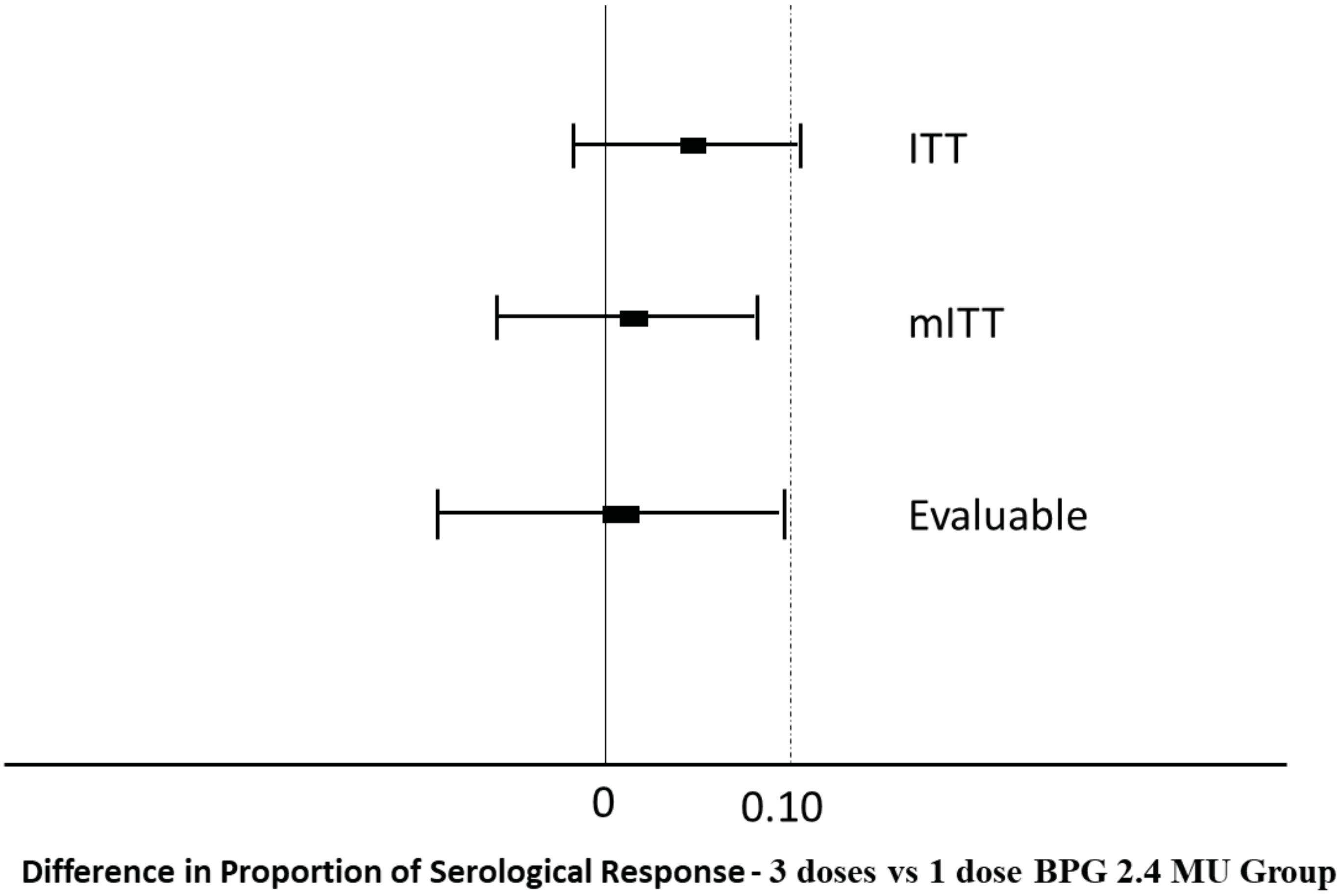


Figures with a similar format:

**Figure 12: Difference in Proportion of Subjects with Serological Response Between Two Treatment Groups by Month 12**

**Figure 13: Maximum Severity of Jarisch-Herxheimer reaction per Subject by Time Post First Dose - Safety Population**

[Implementation Notes:

- Include a panel on the right for "All Subjects"
- Change the time points (vertical axis) into "Time Post First Dose (Hours)" 0-12 hours, >12-24 hours, >24-48 hours]

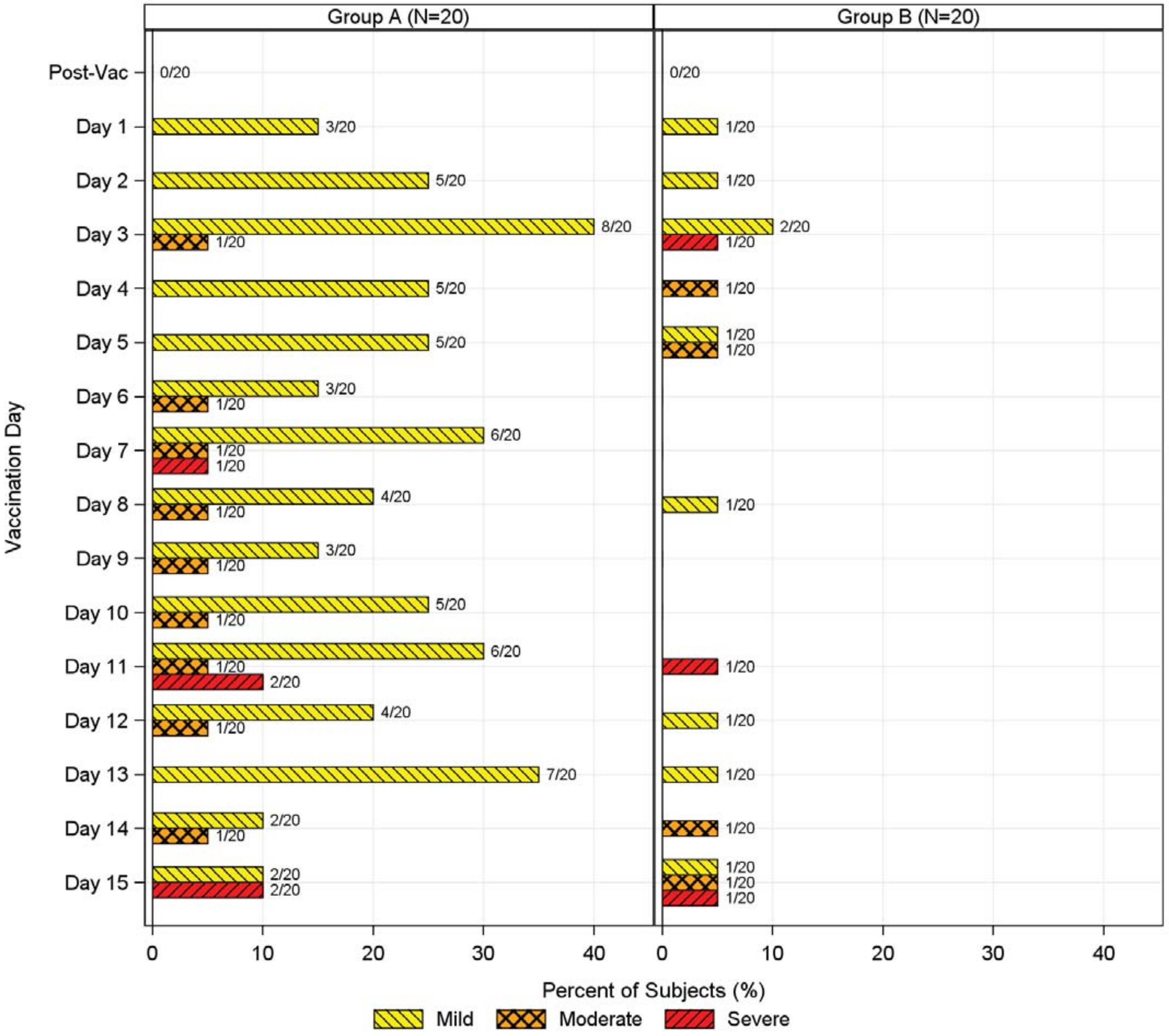

**Figure 14: Maximum Severity of Solicited Local Symptoms per Subject by Dose - Safety Population**

[Implementation Notes:

- Include a panel on the right for "All Subjects"
- Change the time points into "Dose" Post Dose 1, Post Dose 2, Post Dose 3]

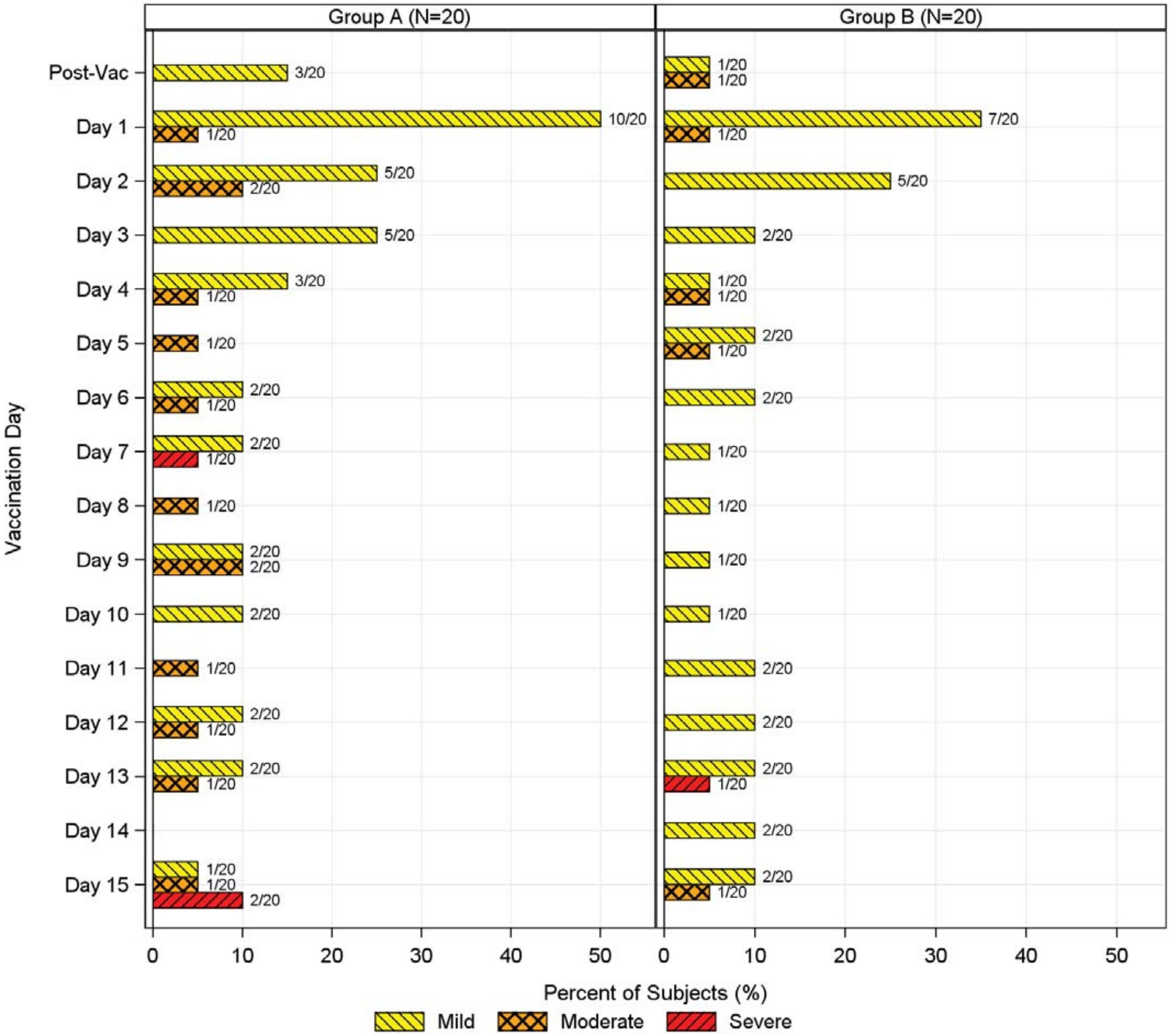

**Figure 15: Number of Related Unsolicited Adverse Events by MedDRA System Organ Class and Severity - Safety Population**

[Implementation Notes:

- Include a panel on the right for "All Subjects"]

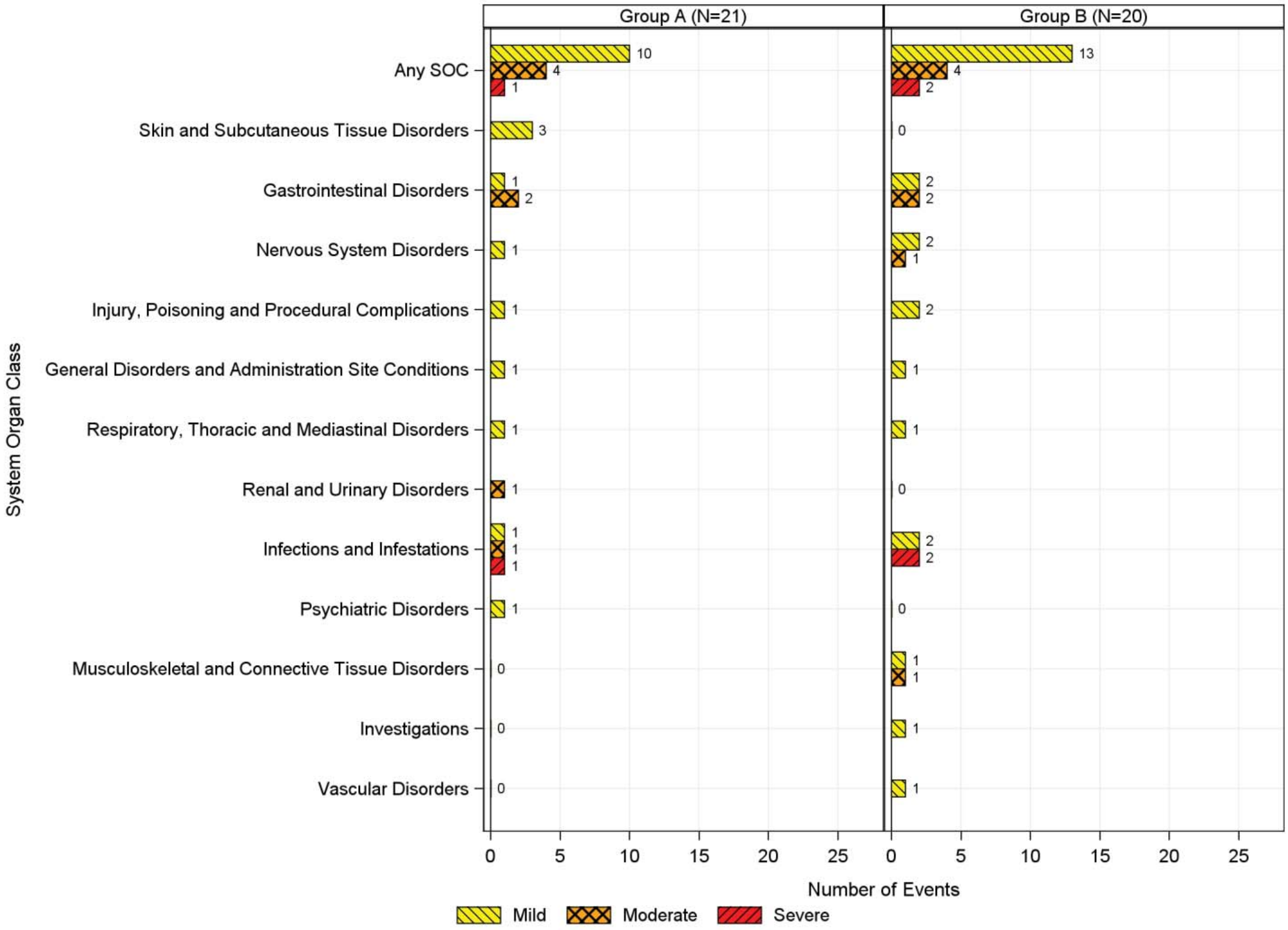

**Figure 16: Number and Percentage of Subjects Experiencing Related Unsolicited Adverse Events by MedDRA® System Organ Class and Maximum Severity - Safety Population**

[Implementation Notes:

- Include a panel on the right for "All Subjects"]

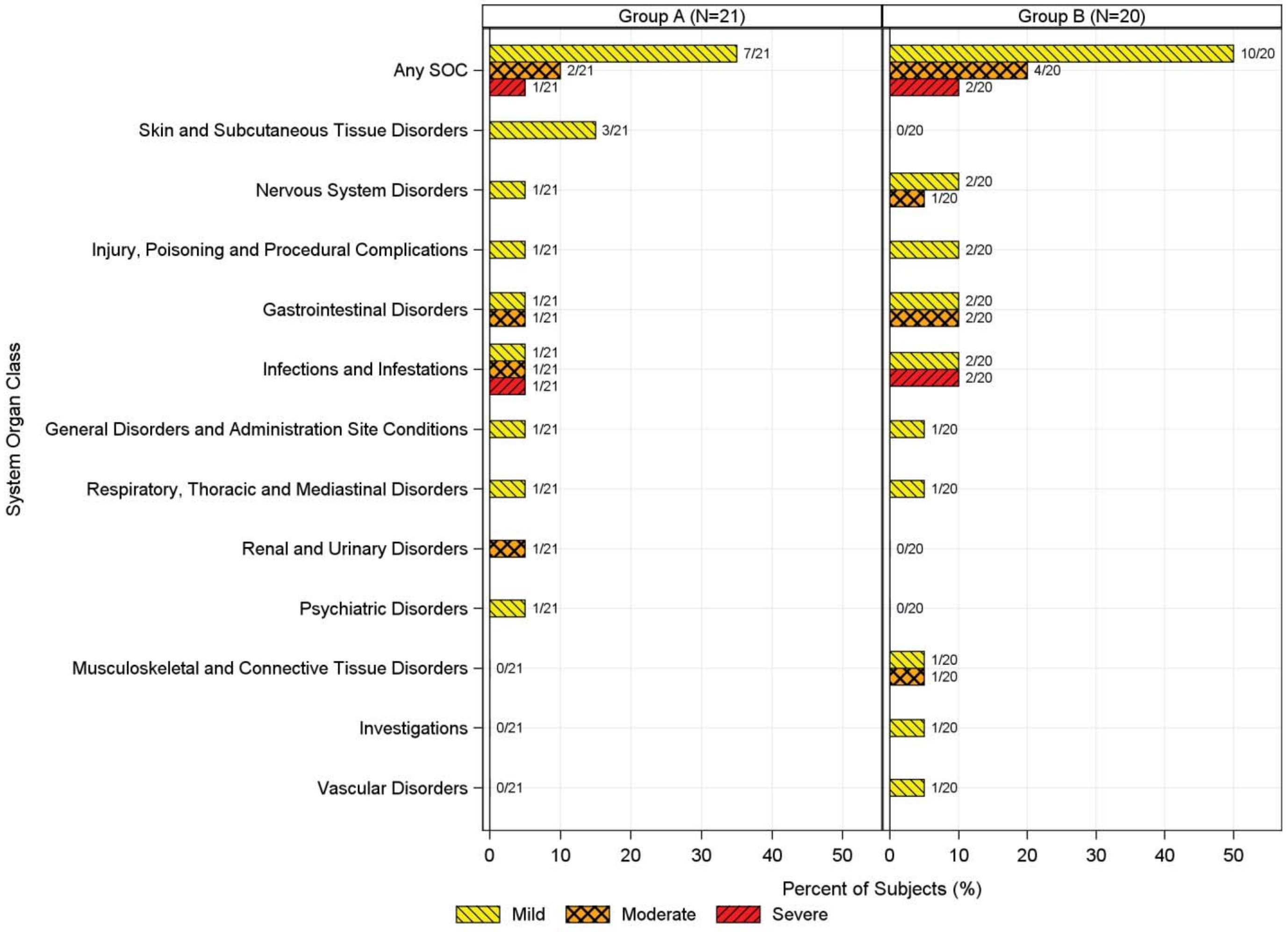

**Figure 17: Number of Unsolicited Adverse Events by MedDRA System Organ Class and Relationship to Treatment - Safety Population**

[Implementation Note:

- Include "Any SOC" bars
- Two treatment groups are One Dose BPG 2.4 MU and Three Doses BPG 2.4 MU
- Put "All Subjects" panel on the right]

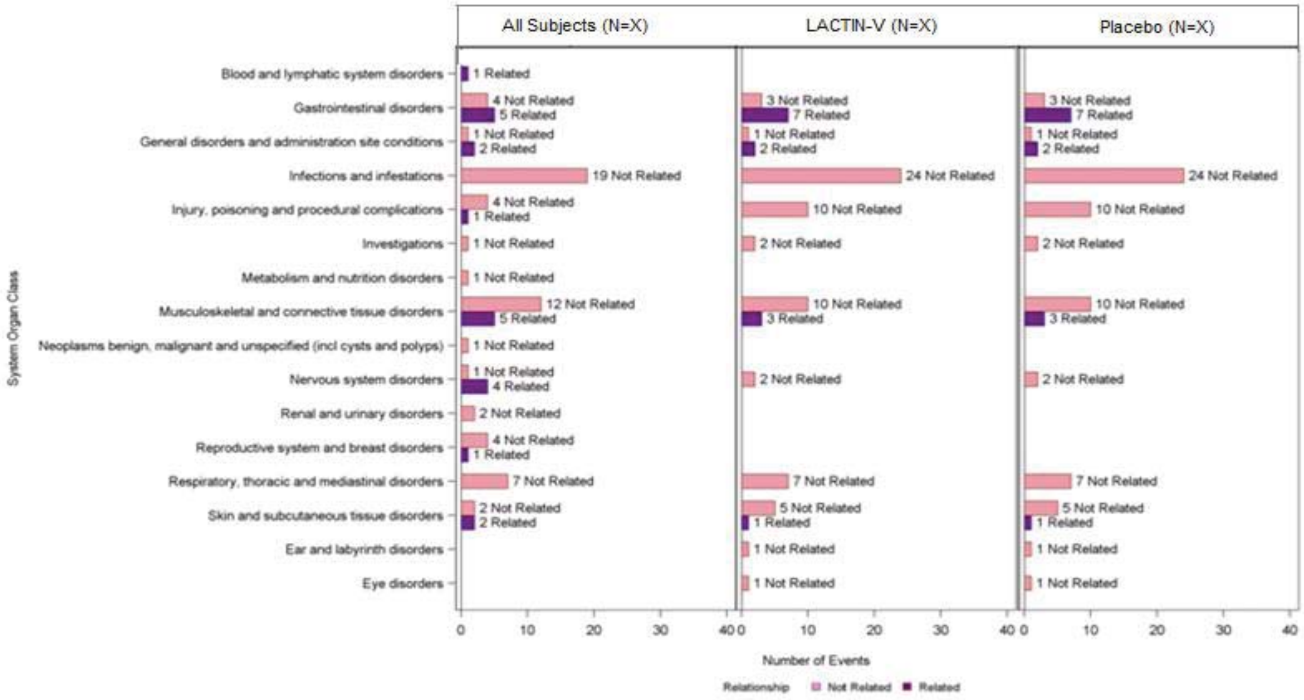

**Figure 18: Number and Percentage of Subjects Experiencing Unsolicited Adverse Events by MedDRA System Organ Class and Relationship to Treatment - Safety Population**

[Implementation Note:

- Include “Any SOC” bars
- Two treatment groups are One Dose BPG 2.4 MU and Three Doses BPG 2.4 MU
- Put “All Subjects” panel on the right
- Legend see Figure 5, number of subject/total]

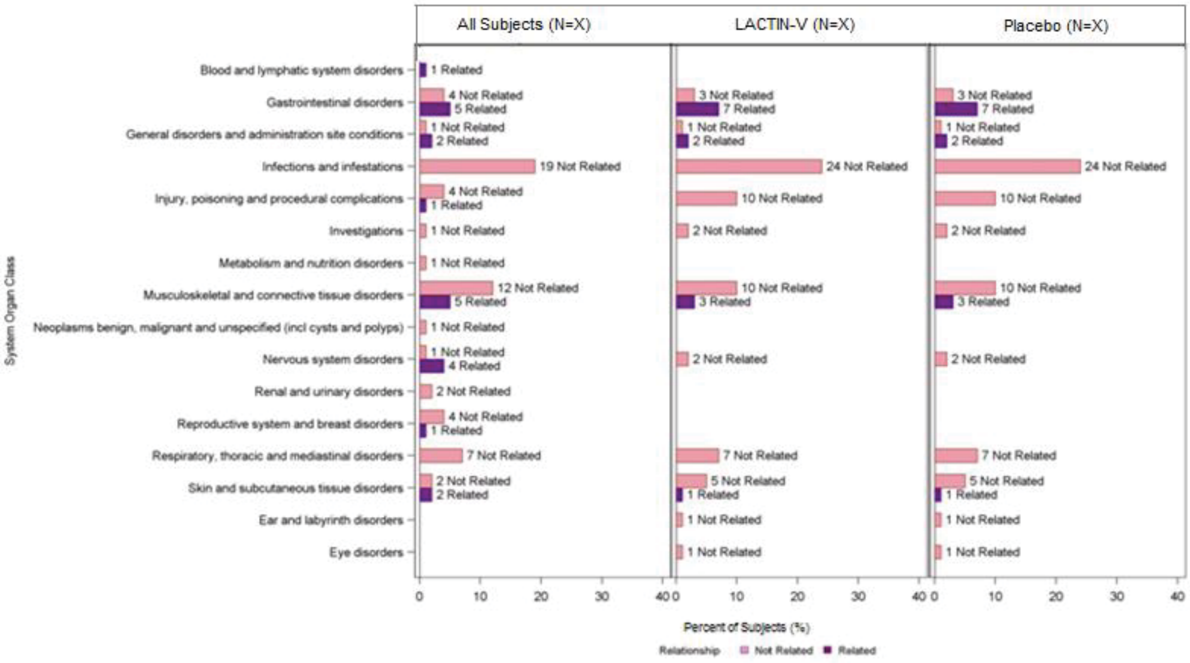



PRIVILEGED AND CONFIDENTIAL COMMUNICATION

# APPENDIX 3. LISTINGS MOCK-UPS

## LISTINGS

Listing 1: Subjects Excluded from Analysis Populations ....108
Listing 2: Early Terminations or Discontinued Subjects-All Enrolled Subjects ....109
Listing 3: Subject-Specific Protocol Deviations-All Enrolled Subjects ....110
Listing 4: Non-Subject-Specific Protocol Deviations ....111
Listing 5: Individual Efficacy Response Data - All Enrolled Subjects ....112
Listing 6: Compliance Data - All Enrolled Subjects ....113
Listing 7: Demographic and Other Baseline Characteristics - All Enrolled Subjects ....114
Listing 8: Sexual Behavior History Part I -All Enrolled Subjects ....115
Listing 9: Sexual Behavior History Part II - All Enrolled Subjects ....116
Listing 10: Sexual Behavior History Part III - Sexually Transmitted Disease Testing History at Baseline - All Enrolled Subjects ....117
Listing 11: Sexually Transmitted Infections (STI) Testing result ....118
Listing 12: Pre-Existing and Concurrent Medical Conditions ....119
Listing 13: Concomitant Medications ....120
Listing 14: Solicited Systemic Reaction Events – Safety Population ....121
Listing 15: Solicited Local Reaction Events – Safety Population ....122
Listing 16: Unsolicited Adverse Events - Safety Population ....124
Listing 17: Pregnancy Reports – Maternal Information ....125
Listing 18: Pregnancy Reports – Gravida and Para ....126
Listing 19: Pregnancy Reports – Live Birth Outcomes ....127
Listing 20: Pregnancy Reports – Still Birth Outcomes ....128
Listing 21: Pregnancy Reports – Spontaneous, Elective, or Therapeutic Abortion Outcomes ....129
Listing 22: Syphilis Clinical Assessment ....130
Listing 23: HIV Testing – All Enrolled Subjects ....131
Listing 24: TPPA Testing - All Enrolled Subjects ....132
Listing 25: Vital Signs – Safety Population ....133
Listing 26: Abnormal Physical Exam Findings ....134


PRIVILEGED AND CONFIDENTIAL COMMUNICATION

**Listing of Subjects Receiving Investigational Product**

(not included in SAP, but this is a placeholder for the CSR)

**Listing 1: Subjects Excluded from Analysis Populations**

Implementation Notes:

1. Sort order will be Treatment group, Subject ID

2. Reasons Subject Excluded 1) should be N/A if the subject was not excluded from any analysis population; 2) should match the same verbiage that is used on the Analysis population tables unless it's a special case recorded in the study plan with the reason confirmed by the PI before study lock.]

| Randomized Treatment Group | Subject ID | Analyses in which Subject is Included | Analyses from which Subject is Excluded | Results Available? | Reason(s) Subject Excluded |
|---|---|---|---|---|---|
| One Dose BPG 2.4 MU / Three Doses BPG 2.4 MU | xxxxxx | [e.g., Safety, ITT, mITT, Evaluable by Month 6, Evaluable by Month 12] | [e.g., Safety, ITT, mITT, Evaluable by Month 6, Evaluable by Month 12] | Yes/No | xxxxx/N/A |
| One Dose BPG 2.4 MU / Three Doses BPG 2.4 MU | xxxxxx | [e.g., Safety, ITT, mITT, Evaluable by Month 6, Evaluable by Month 12] | [e.g., Safety, ITT, mITT Evaluable by Month 6, Evaluable by Month 12] | Yes/No | xxxxx/N/A |
| One Dose BPG 2.4 MU / Three Doses BPG 2.4 MU | xxxxxx | [e.g., Safety, ITT, mITT, Evaluable by Month 6, Evaluable by Month 12] | [e.g., Safety, ITT, mITT Evaluable by Month 6, Evaluable by Month 12] | Yes/No | xxxxx/N/A |

Note: If the subject was excluded from any analysis population, "Yes" in the "Results Available" column indicates that available data were removed from the analysis. If the subject was not excluded from any analysis population, "Yes" in the "Results Available" column indicates that data was available for all analysis populations. "No" indicates that no data were available for inclusion in the analysis.

**Listing 2: Early Terminations or Discontinued Subjects-All Enrolled Subjects**

[Implementation Notes:

1. Sort order will be by Treatment Group, Subject ID, Category

2. Category will be "Early Termination" or "Treatment Discontinuation". If a subject discontinued treatment and terminated early, they will have two records.

3. In the “Reason” column, concatenate any “specify” fields, including AE number and DV number.]

| Randomized Treatment Group | Subject ID | Category | Study Day Corresponding to Early Termination/Treatment Discontinuation/Completion | Reason for Early Termination or Treatment Discontinuation |
|---|---|---|---|---|
| One Dose BPG 2.4 MU / Three Doses BPG 2.4 MU | xxxxxxx | Early Termination/Treatment Discontinuation | xx | xxxxxxxxxxxxxxxxx/N/A |
| One Dose BPG 2.4 MU / Three Doses BPG 2.4 MU | xxxxxxx | Early Termination/Treatment Discontinuation | xx | xxxxxxxxxxxxxxxxx/N/A |

Note. Subjects who had early terminated or discontinued due to Covid-19 are flagged.


PRIVILEGED AND CONFIDENTIAL COMMUNICATION

**Listing 3: Subject-Specific Protocol Deviations-All Enrolled Subjects**

[Implementation Notes:

1. Treatment group is the randomized treatment group.
2. Sort order will be by Treatment Group, Subject ID, Deviation Number.
3. Deviation description column will contain all subfields concatenated together.
4. In the Reason for Deviation column concatenate any specified fields.]

| Treatment Group | Subject ID | Deviation Number | Study Day | Deviation Description | Deviation Category | Reason for Deviation | Deviation Affected Product Stability? | Deviation Resulted in AE? | Deviation Resulted in Subject Termination? | Deviation Resolution | Comments |
|---|---|---|---|---|---|---|---|---|---|---|---|
| xxxx | xxxxx | xx | xx | xxxxxxx | xxxxxxxxxxx | xxxxxxx | Yes/No/ N/A | Yes/No | Yes/No | Yes/No | xxxxxxxxxxx |
| xxxx | xxxxx | xx | xx | xxxxxxx | xxxxxxxxxxx | xxxxxxx | Yes/No/ N/A | Yes/No | Yes/No | Yes/No | -- |

Note. Subjects who had deviation due to Covid-19 are flagged.

**Listing 4: Non-Subject-Specific Protocol Deviations**

[Implementation Notes:

1. Sort order will be by Site Name, Start Date

2. In the Deviation column concatenate any specified fields

3. In the Reason for Deviation column concatenate any specified fields.]

| Site | Start Day | Deviation | End Day | Reason for Deviation | Deviation Resulted in Subject Termination? | Deviation Affected Product Stability? | Deviation Category | Deviation Resolution | Comments |
|---|---|---|---|---|---|---|---|---|---|
| xxxx | xx | xxxx | xx | xxxx | Yes/No | Yes/No/N/A | xxxx | xxxx | Xxxx |
| xxxx | xx | xxxx | xx | xxxx | Yes/No | Yes/No/N/A | xxxx | xxxx | Xxxx |

Note. Deviation due to Covid-19 are flagged.


PRIVILEGED AND CONFIDENTIAL COMMUNICATION

## Listing 5: Individual Efficacy Response Data - All Enrolled Subjects

[Implementation Notes:

1. Sort order will be by Treatment Group and Subject ID, Planned Time Point. ]

| Randomized Treatment Group | Subject ID | Planned Time Point | Actual Testing Study Day | RPR Result | RPR Titer | Ratio to baseline | Serological Response to Therapy by Month 6 | Serological Response to Therapy by Month 12 | Serological Response to Therapy for Exploratory Efficacy Analysis |
|---|---|---|---|---|---|---|---|---|---|
| One Dose BPG 2.4 MU / Three Doses BPG 2.4 MU | xxxxx | Visit 1 | xxx | REACTIVE/NON-REACTIVE/INVALID | xxx | - | - | | - |
| One Dose BPG 2.4 MU / Three Doses BPG 2.4 MU | xxxxx | Visit 2 | xxx | REACTIVE/NON-REACTIVE/INVALID | xxx | xx | Response/<br>Non-Response | Response/<br>Non-Response | Response/<br>Non-Response/<br>Failure |
| One Dose BPG 2.4 MU / Three Doses BPG 2.4 MU | xxxxx | Visit 3 | xxx | REACTIVE/NON-REACTIVE/INVALID | xxx | xx | | | |
| One Dose BPG 2.4 MU / Three Doses BPG 2.4 MU | xxxxx | Visit 4 | xxx | REACTIVE/NON-REACTIVE/INVALID | xxx | xx | | | |
| One Dose BPG 2.4 MU / Three Doses BPG 2.4 MU | xxxxx | Visit 5 | xxx | REACTIVE/NON-REACTIVE/INVALID | xxx | xx | | | |
| One Dose BPG 2.4 MU / Three Doses BPG 2.4 MU | xxxxx | Visit 6 | xxx | REACTIVE/NON-REACTIVE/INVALID | xxx | xx | | | |
| One Dose BPG 2.4 MU / Three Doses BPG 2.4 MU | xxxxx | Early Termination prior to month 6 | xxx | REACTIVE/NON-REACTIVE/INVALID | xxx | xx | | | |
| One Dose BPG 2.4 MU / Three Doses BPG 2.4 MU | xxxxx | Visit 7 | xxx | REACTIVE/NON-REACTIVE/INVALID | xxx | xx | NA | | NA |
| One Dose BPG 2.4 MU / Three Doses BPG 2.4 MU | xxxxx | Visit 8 | xxx | REACTIVE/NON-REACTIVE/INVALID | xxx | xx | | | |
| One Dose BPG 2.4 MU / Three Doses BPG 2.4 MU | xxxxx | Early Termination after month 8 | xxx | REACTIVE/NON-REACTIVE/INVALID | xxx | xx | | | |

**Listing 6: Compliance Data - All Enrolled Subjects**

[Implementation Notes:

1. Sort order is Randomized treatment Group, Subject ID, Visit.]

| Treatment Group | Subject ID | Planned Time Point | Actual Study Day | Dose Administered within the Assigned Visit Window? | Compliance Status |
|---|---|---|---|---|---|
| One Dose BPG 2.4 MU | xxxxxx | Visit 1 | xx | Yes/No | Compliant/Non-compliant |
| Three Doses BPG 2.4 MU | xxxxxx | Visit 1 | xx | Yes/No | Compliant/Non-compliant |
| | | Visit 2 | xx | Yes/No | |
| | | Visit 3 | xx | Yes/No | |

**Listing 7: Demographic and Other Baseline Characteristics - All Enrolled Subjects**

[Implementation Notes:

1. Sort order will be by Treatment Group, Subject ID

2. For the Race column, if a subject is Multi-Racial, all races will be listed, separated by a comma]

| Randomized Treatment Group | Subject ID | Sex | Age at Enrollment (years) | Ethnicity | Race | Baseline HIV Status | Highest Level of Education the Subject Has Completed | Number of Years of Formal Education the Subject Completed |
|---|---|---|---|---|---|---|---|---|
| One Dose BPG 2.4 MU / Three Doses BPG 2.4 MU | xxxxxx | Male/Female | xx | xxxxxx | xxxxxx | HIV-infected/HIV-uninfected | xxx/xxx/xxx/xxx/xxx | xx |
| One Dose BPG 2.4 MU / Three Doses BPG 2.4 MU | xxxxxx | Male/Female | xx | xxxxxx | xxxxxx | HIV-infected/HIV-uninfected | xxx/xxx/xxx/xxx/xxx | xx |
| One Dose BPG 2.4 MU / Three Doses BPG 2.4 MU | xxxxxx | Male/Female | xx | xxxxxx | xxxxxx | HIV-infected/HIV-uninfected | xxx/xxx/xxx/xxx/xxx | xx |


PRIVILEGED AND CONFIDENTIAL COMMUNICATION

**Listing 8: Sexual Behavior History Part I -All Enrolled Subjects**

[Implementation Notes:

1. Sort order will be by Treatment Group, Subject ID

2. If the subject has used "other" gender identity or sexual orientation, list the specified field in the cell.

4. Missing response will be populated as "Not Reported".

5. For "Any known exposures to STD" variable, if response was Yes or Unknown, concatenate the SX2 Comments field.]

| Time Point | Subject's gender identity | Subject's sexual orientation | Subject's sexual partner | Treated or Require Retreatment for Syphilis[a] | How was retreatment need determined[b]? | Any known exposures to syphilis since the last study visit? | Any known exposures to STD[c]? |
|---|---|---|---|---|---|---|---|
| **Treatment Group: , Subject ID:** | | | | | | | |
| Visit 1 | Man / Woman / Trans Male / Trans Female / Genderqueer / Multiple / Non-binary / Other | Heterosexual / Homosexual / Bisexual / Other | xxx / xxx/… | N/A | N/A | N/A | Yes / No / Unknown |
| Visit 2 | N/A | N/A | N/A | Yes/No/Unknown | xxxx | Yes/No/Unknown | Yes / No / Unknown |
| Visit 3 | N/A | N/A | N/A | Yes/No/Unknown | xxxx | Yes/No/Unknown | Yes / No / Unknown |
| Visit 4 | N/A | N/A | N/A | Yes/No/Unknown | xxxx | Yes/No/Unknown | Yes / No / Unknown |
| Visit 5 | N/A | N/A | N/A | Yes/No/Unknown | xxxx | Yes/No/Unknown | Yes / No / Unknown |
| Visit 6 | N/A | N/A | N/A | Yes/No/Unknown | xxxx | Yes/No/Unknown | Yes / No / Unknown |
| Visit 7 | N/A | N/A | N/A | Yes/No/Unknown | xxxx | Yes/No/Unknown | Yes / No / Unknown |
| Visit 8 | N/A | N/A | N/A | Yes/No/Unknown | xxxx | Yes/No/Unknown | Yes / No / Unknown |

Notes: Subject's gender identity, subject's sexual orientation, and subject's sexual partner are baseline questions only. Treated or require retreatment for syphilis, how was retreatment determined, and any known exposures to syphilis since the last study visit are follow-up questions only.

[a] Was the subject treated or does the subject require retreatment for syphilis infection apart from study treatment received according to their randomization assignment?

[b] If the subject was treated or require retreatment for syphilis infection apart from study treatment received according to their randomization assignment, how was retreatment need determined?

[c] The question is "Did the subject have any known exposures to an STD in the past 60 days?" for Visit 1, and "Any known exposures to another STD since the last study visit?" for the follow-up visits.

**Listing 9: Sexual Behavior History Part II - All Enrolled Subjects**

[Implementation Notes:

1. Sort order will be by Treatment Group, Subject ID

3. Missing response will be populated as "Not Reported".

4. If the subject had sexual intercourse in the past 60 days or since the last visit, then the cells will be "N/A".]

| Time Point | Has the Subject Had Sexual Intercourse in the Specified Timeframe? | Days Since the Subject Last Had Sexual Intercourse in the Specified Timeframe | Sites of Exposure in the Specified Timeframe | How Often Was a Condom or Barrier for Protection Used in the Specified Timeframe? | Total Number of Sexual Partners in the Specified Timeframe | Number of New Partners in the Specified Timeframe | Number of Regular Partners in the Specified Timeframe | Number of Occasional Partners in the Specified Timeframe | Number of Male Partners in the Specified Timeframe | Number of Female Partners in the Specified Timeframe | Number of Continuing Partners in the Specified Timeframe |
|---|---|---|---|---|---|---|---|---|---|---|---|
| **Treatment Group: , Subject ID:** | | | | | | | | | | | |
| Visit 1 | Yes / No / Unknown | xx / N/A | Oral receptive / Oral active / Rectal receptive / Rectal insertive / Genital/vaginal / N/A | Always / Sometimes / Never / N/A | xx | xx | xx | xx | xx | xx | N/A |
| Visit 2 | Yes / No / Unknown | xx | Oral receptive / Oral active / Rectal receptive / Rectal insertive / Genital/vaginal / N/A | Always / Sometimes / Never / N/A | xx | xx | N/A | N/A | N/A | N/A | xx |
| Visit 3 | Yes / No / Unknown | xx | xxxx | xxxx | xx | xx | N/A | N/A | N/A | N/A | xx |
| Visit 4 | Yes / No / Unknown | xx | xxxx | xxxx | xx | xx | N/A | N/A | N/A | N/A | xx |
| Visit 5 | Yes / No / Unknown | xx | xxxx | xxxx | xx | xx | N/A | N/A | N/A | N/A | xx |
| Visit 6 | Yes / No / Unknown | xx | xxxx | xxxx | xx | xx | N/A | N/A | N/A | N/A | xx |
| Visit 7 | Yes / No / Unknown | xx | xxxx | xxxx | xx | xx | N/A | N/A | N/A | N/A | xx |
| Visit 8 | Yes / No / Unknown | xx | xxxx | xxxx | xx | xx | N/A | N/A | N/A | N/A | xx |

Notes: The specified timeframe for visit 1 is "in the past 60 days", and "since the last visit" for the follow-up visits.

For Visit 1, if subject had not had sexual intercourse in the past 60 days, response to the rest of the variables in this table will be N/A. For the follow-up visits, if subject had not had sexual intercourse since the previous visit, response to rest of the variables in this table will be N/A. Unscheduled visit assessments are included.

Number of continuing partners is a variable for follow-up visits only. Number of regular partners, number of occasional partners, number of male partners and number of female partners are for Visit 1 only.

**Listing 10: Sexual Behavior History Part III - Sexually Transmitted Disease Testing History at Baseline - All Enrolled Subjects**

[Implementation Notes:

1. Sort order will be by Actual Treatment Group, Subject ID

2. If all subjects received the correct treatment, only display a single "Treatment Group" column.]

| Treatment Group | Subject ID | Has been diagnosed with gonorrhea in the past 60 days? | Days since the subject last diagnosed with gonorrheal in the past 60 days | Has the subject been tested for gonorrhea in the past 14 days? | In the past 14 days, if the subject had been tested for gonorrhea, what were the test results? | Has been diagnosed with chlamydia in the past 60 days? | Days since the subject last diagnosed with chlamydia in the past 60 days | Has the subject been tested for chlamydia in the past 14 days? | In the past 14 days, if the subject had been tested for chlamydia, what were the test results? | Has been diagnosed with any other STI in the past 60 days? | Days since the subject last diagnosed with any other STI in the past 60 days |
|---|---|---|---|---|---|---|---|---|---|---|---|
| One Dose BPG 2.4 MU / Three Doses BPG 2.4 MU | xxxxxx | Yes / No | xx | Yes / No | Positive / Negative | Yes / No | xx | Yes / No | Positive / Negative | Yes / No | xx |
| One Dose BPG 2.4 MU / Three Doses BPG 2.4 MU | xxxxxx | Yes / No | xx | Yes / No | Positive / Negative | Yes / No | xx | Yes / No | Positive / Negative | Yes / No | xx |

**Listing 11: Sexually Transmitted Infections (STI) Testing result**

[Implementation Notes:

1. Sort order will be by Treatment Group, Subject ID, and time point]

| Treatment Group | Subject ID | Time Point | Was STI testing performed? | Test type | Was testing performed? | Specimen collection date | Site of specimen collection | Test Result |
|---|---|---|---|---|---|---|---|---|
| | xxxx | Visit 1 | Yes / No / NA | chlamydia | Yes / No | ddMMMyyyy | Genitourinary / Pharyngeal/ Rectal | Positive /negative |
| | | | | gonorrhea | Yes / No | ddMMMyyyy | Genitourinary / Pharyngeal/ Rectal | Positive /negative |
| | | | | Other STIs | Yes / No | ddMMMyyyy | Genitourinary / Pharyngeal/ Rectal | Positive /negative |
| | | | | specify STI | Yes / No | ddMMMyyyy | Genitourinary / Pharyngeal/ Rectal | Positive /negative |
| | | Visit 2 | Yes / No / NA | chlamydia | Yes / No | ddMMMyyyy | Genitourinary / Pharyngeal/ Rectal | Positive /negative |
| | | | | gonorrhea | Yes / No | ddMMMyyyy | Genitourinary / Pharyngeal/ Rectal | Positive /negative |
| | | | | Other STIs | Yes / No | ddMMMyyyy | Genitourinary / Pharyngeal/ Rectal | Positive /negative |
| | | | | specify STI | Yes / No | ddMMMyyyy | Genitourinary / Pharyngeal/ Rectal | Positive /negative |
| | | Visit 3 | Yes / No / NA | chlamydia | Yes / No | ddMMMyyyy | Genitourinary / Pharyngeal/ Rectal | Positive /negative |
| | | | | gonorrhea | Yes / No | ddMMMyyyy | Genitourinary / Pharyngeal/ Rectal | Positive /negative |
| | | | | Other STIs | Yes / No | ddMMMyyyy | Genitourinary / Pharyngeal/ Rectal | Positive /negative |
| | | | | specify STI | Yes / No | ddMMMyyyy | Genitourinary / Pharyngeal/ Rectal | Positive /negative |
| | | … | | | | | | |
| | | Visit 8 | Yes / No / NA | chlamydia | Yes / No | ddMMMyyyy | Genitourinary / Pharyngeal/ Rectal | Positive /negative |
| | | | | gonorrhea | Yes / No | ddMMMyyyy | Genitourinary / Pharyngeal/ Rectal | Positive /negative |
| | | | | Other STIs | Yes / No | ddMMMyyyy | Genitourinary / Pharyngeal/ Rectal | Positive /negative |
| | | | | specify STI | Yes / No | ddMMMyyyy | Genitourinary / Pharyngeal/ Rectal | Positive /negative |
| xxxx | xxxx | … | … | … | … | … | … | … |

**Listing 12: Pre-Existing and Concurrent Medical Conditions**

[Implementation Notes:

1. Sort order is Treatment Group, Subject ID, MH Number.

2. "Condition Start Day" and "Condition End Day" are relative to enrollment (which is Day 1, day before enrollment is Day -1). Display the study days for start day and end day that are during the study, as well as within the past 14 days prior to the study. For medical history start/end dates that are > 14 days prior to enrollment, rather than use exact study days, categorize as follows:

- > 5 years prior to enrollment

- 1-5 years prior to enrollment

-1-12 months prior to enrollment

-If Ongoing at the end of the study, display "Ongoing' in the "Condition End Day" column.

-If ending is unknown at the end of the study, display "Unknown" in the "Condition End Day" column.]

| Randomized Treatment Group | Subject ID | MH Number | Medical History Term | Condition Start Day | Condition End Day | MedDRA System Organ Class | MedDRA Preferred Term |
|---|---|---|---|---|---|---|---|
| One Dose BPG 2.4 MU / Three Doses BPG 2.4 MU | xxxxxx | xxx | xxxxxx | xx | xx | xxxxxx | xxxxxx |
| One Dose BPG 2.4 MU / Three Doses BPG 2.4 MU | xxxxxx | xxx | xxxxxx | xx | xx | xxxxxx | xxxxxx |

Note: This listing includes subjects who had pre-existing and/or concurrent medical conditions among all enrolled subjects.


PRIVILEGED AND CONFIDENTIAL COMMUNICATION

**Listing 13: Concomitant Medications**

[Implementation Notes:

1. Sort order is treatment group, Subject ID, concomitant medication number.

2. 'Medication Start Day' and 'Medication End Day' are relative to enrollment (which is Day 1, day before enrollment is Day -1). Display the study days for start day and end day that are during the study, as well as within 30 days prior to the study. For medication start/end dates that are > 30 days prior to enrollment, rather than use exact days, categorize as follows:

- > 5 years prior to enrollment

- 1- 5 years prior to enrollment

- 1-12 months prior to enrollment.

- For 'Medication End Day', if medication is Ongoing, display 'Ongoing' in the Medication End Day' column.

- For 'Medication End Day', if end of medication is unknown, display 'Unknown' in the 'Medication End Day' column.

3. If a Medication is taken for an AE, then concatenate the conmed with the Advere Events by AENUM and report the AETERM, plus the AE Number.

4. If a Medication is taken for an MH, then concatenate the conmed with the Medical History event by MHNUM and report the MHTERM, plus the MH Number.

5. Include the birth control information in this dataset. The birth control information is coming from the RP/SUPPRP or BC1 dataset.]

| Treatment Group | Subject ID | Concomitant Medication Number | Medication | Medication Start Day | Medication End Day | Indication | Taken for an AE? (AE Description; AE Number) | Taken for a condition on Medical History? (MH Description; MH Number) | ATC Level 1 (ATC Level 2) |
|---|---|---|---|---|---|---|---|---|---|
| One Dose BPG 2.4 MU / Three Doses BPG 2.4 MU | xxxxxx | xx | xxxxxx | xx | xx | xxxxxx | Yes/No<br>xxxxx; xx | Yes/No<br>xxxxx; xx | |
| One Dose BPG 2.4 MU / Three Doses BPG 2.4 MU | xxxxxx | xx | xxxxxx | xx | xx | xxxxxx | Yes/No<br>xxxxx; xx | Yes/No<br>xxxxx; xx | |

Note: This listing consists of subjects who had pre-existing and/or concurrent medical conditions among all enrolled subjects.

**Listing 14: Solicited Systemic Reaction Events – Safety Population**

[Implementation Notes:

1. Sort order is Treatment group, Subject ID, Study Day.]

| Randomized Treatment Group | Subject ID | Study Day of symptoms reviewed: | Reporting time (24-hr clock) | Jarisch-Herxheimer Reaction Symtom | Study Day of Symptom Start Date | Start Time (24-hr clock, hh:mm) | Study Day of Symptom Stop Date | Stop Time (24-hr clock, hh:mm) | Ongoing | Maximum Severity |
|---|---|---|---|---|---|---|---|---|---|---|
| | | | | Feverishness | | | | | | |
| | | | | Chills | | | | | | |
| | | | | Myalgia | | | | | | |
| | | | | Weakness | | | | | | |
| | | | | Flushing | | | | | | |
| | | | | Worsening of skin rash | | | | | | |
| | | | | Tachycardia | | | | | | |
| | | | | Heart palpitations | | | | | | |
| | | | | Generalized arthralgia | | | | | | |
| | | | | Nausea | | | | | | |
| | | | | Headache | | | | | | |
| | | | | Dizziness | | | | | | |

Notes: Subject who did not experience any symptoms of the Jarisch-Herxheimer Reaction in the 24-hours following Visit 1 will not be listed.
Symptoms that did not occur will not be listed.

### Listing 15: Solicited Local Reaction Events – Safety Population

[Implementation Notes:

1. Sort order is treatment group, Subject ID, Study Day.

2. Subjects in one-dose group will only have rows for dose 1. Subjects in 3-dose group each will have rows for all 3 doses.]

| Dose Number | Post Dose Day | Pain or Tenderness Severity / Study Day of Stop Date Post-dose[a] | Erythema or Redness Severity Diameter (mm) / Study Day of Stop Date Post-dose[a] | Induration or Swelling Severity Diameter (mm) / Study Day of Stop Date Post-dose[a] |
|---|---|---|---|---|
| **Treatment Group: Subject ID:** | | | | |
| 1 | 1 | None or Mild (x) or Moderate (x) or Severe (x) / Not Done | None or Mild (x) or Moderate (x) or Severe (x) / Not Done | None or Mild (x) or Moderate (x) or Severe (x) / Not Done |
| 1 | 2 | None or Mild (x) or Moderate (x) or Severe (x) / Not Done | None or Mild (x) or Moderate (x) or Severe (x) / Not Done | None or Mild (x) or Moderate (x) or Severe (x) / Not Done |
| 1 | 3 | None or Mild (x) or Moderate (x) or Severe (x) / Not Done | None or Mild (x) or Moderate (x) or Severe (x) / Not Done | None or Mild (x) or Moderate (x) or Severe (x) / Not Done |
| 1 | 4 | None or Mild (x) or Moderate (x) or Severe (x) / Not Done | None or Mild (x) or Moderate (x) or Severe (x) / Not Done | None or Mild (x) or Moderate (x) or Severe (x) / Not Done |
| 1 | 5 | None or Mild (x) or Moderate (x) or Severe (x) / Not Done | None or Mild (x) or Moderate (x) or Severe (x) / Not Done | None or Mild (x) or Moderate (x) or Severe (x) / Not Done |
| 1 | 6 | None or Mild (x) or Moderate (x) or Severe (x) / Not Done | None or Mild (x) or Moderate (x) or Severe (x) / Not Done | None or Mild (x) or Moderate (x) or Severe (x) / Not Done |
| 1 | >6 | Mild (x) or Moderate (x) or Severe (x) / x or N/A | Mild (x) or Moderate (x) or Severe (x) / x or N/A | Mild (x) or Moderate (x) or Severe (x) / x or N/A |
| 2 | 1 | None or Mild (x) or Moderate (x) or Severe (x) / Not Done | None or Mild (x) or Moderate (x) or Severe (x) / Not Done | None or Mild (x) or Moderate (x) or Severe (x) / Not Done |
| 2 | 2 | None or Mild (x) or Moderate (x) or Severe (x) / Not Done | None or Mild (x) or Moderate (x) or Severe (x) / Not Done | None or Mild (x) or Moderate (x) or Severe (x) / Not Done |
| 2 | 3 | None or Mild (x) or Moderate (x) or Severe (x) / Not Done | None or Mild (x) or Moderate (x) or Severe (x) / Not Done | None or Mild (x) or Moderate (x) or Severe (x) / Not Done |
| 2 | 4 | None or Mild (x) or Moderate (x) or Severe (x) / Not Done | None or Mild (x) or Moderate (x) or Severe (x) / Not Done | None or Mild (x) or Moderate (x) or Severe (x) / Not Done |
| 2 | 5 | None or Mild (x) or Moderate (x) or Severe (x) / Not Done | None or Mild (x) or Moderate (x) or Severe (x) / Not Done | None or Mild (x) or Moderate (x) or Severe (x) / Not Done |
| 2 | 6 | None or Mild (x) or Moderate (x) or Severe (x) / Not Done | None or Mild (x) or Moderate (x) or Severe (x) / Not Done | None or Mild (x) or Moderate (x) or Severe (x) / Not Done |
| 2 | >6 | Mild (x) or Moderate (x) or Severe (x) / x or N/A | Mild (x) or Moderate (x) or Severe (x) / x or N/A | Mild (x) or Moderate (x) or Severe (x) / x or N/A |
| 3 | 1 | None or Mild (x) or Moderate (x) or Severe (x) / Not Done | None or Mild (x) or Moderate (x) or Severe (x) / Not Done | None or Mild (x) or Moderate (x) or Severe (x) / Not Done |
| 3 | 2 | None or Mild (x) or Moderate (x) or Severe (x) / Not Done | None or Mild (x) or Moderate (x) or Severe (x) / Not Done | None or Mild (x) or Moderate (x) or Severe (x) / Not Done |
| 3 | 3 | None or Mild (x) or Moderate (x) or Severe (x) / Not Done | None or Mild (x) or Moderate (x) or Severe (x) / Not Done | None or Mild (x) or Moderate (x) or Severe (x) / Not Done |
| 3 | 4 | None or Mild (x) or Moderate (x) or Severe (x) / Not Done | None or Mild (x) or Moderate (x) or Severe (x) / Not Done | None or Mild (x) or Moderate (x) or Severe (x) / Not Done |
| 3 | 5 | None or Mild (x) or Moderate (x) or Severe (x) / Not Done | None or Mild (x) or Moderate (x) or Severe (x) / Not Done | None or Mild (x) or Moderate (x) or Severe (x) / Not Done |

| Dose Number | Post Dose Day | Pain or Tenderness Severity / Study Day of Stop Date Post-dose[a] | Erythema or Redness Severity Diameter (mm) / Study Day of Stop Date Post-dose[a] | Induration or Swelling Severity Diameter (mm) / Study Day of Stop Date Post-dose[a] |
|---|---|---|---|---|
| 3 | 6 | None or Mild (x) or Moderate (x) or Severe (x) / Not Done | None or Mild (x) or Moderate (x) or Severe (x) / Not Done | None or Mild (x) or Moderate (x) or Severe (x) / Not Done |
| 3 | >6 | Mild (x) or Moderate (x) or Severe (x) / x or N/A | Mild (x) or Moderate (x) or Severe (x) / x or N/A | Mild (x) or Moderate (x) or Severe (x) / x or N/A |

Notes: For Post Dose Day 1 to 6 rows, display the symptom diameters in parentheses next to the severity for Erythema/Redness and Iduration/Swelling; if the Severity of the symptom is None or Not Done, no diameter will be displayed.

[a] For Post Dose Day >6 rows, if the symptom was ongoing after day 6, Study Day of Stop Day Post-dose will be displayed under each symptom column after Severity separated by a '/'; if the symptom was not ongoing after day 6, N/A will be displayed under each symptom column.

**Listing 16: Unsolicited Adverse Events - Safety Population**

[Implementation Notes:

1. Sort order is treatment group, Subject ID, AE Number.]

| Adverse Event | Associated with Dose No. | No. of Days Post Associated Dose | Duration (Days) | Severity | SAE? | Relationship to Study Treatment | In Not Related, Alternative Etiology | Action Taken with Study Treatment | Subject Discontinued Due to AE | Outcome | MedDRA System Organ Class | MedDRA Preferred Term |
|---|---|---|---|---|---|---|---|---|---|---|---|---|
| **Treatment Group: , Subject ID: , AE Number:** | | | | | | | | | | | | |
| xxxxxxx | xx | x | x | xxxxxx | xxxxxx | xxxxxx | xxxxx | xxxxxx | Y/N | xxxxx | xxxxx | xxxxx |
| Comments: xxxxxxxxxxxxx | | | | | | | | | | | | |
| | | | | | | | | | | | | |
| **Treatment Group: , Subject ID: , AE Number:** | | | | | | | | | | | | |
| xxxxxxx | xx | x | x | xxxxxx | xxxxxx | xxxxxx | xxxxx | xxxxxx | Y/N | xxxxx | Xxxxx | xxxxx |
| Comments: xxxxxxxxxxxxx | | | | | | | | | | | | |
| | | | | | | | | | | | | |

Note: For listing of SAEs only, see Table 47.

**Listing 17: Pregnancy Reports – Maternal Information**

[Implementation Notes:

1. Sort order is treatment group, Subject ID]

| Randomized Treatment Group | Subject ID | Pregnancy Number | Study Day Corresponding to Estimated Date of Conception | Source of Maternal Information | Pregnancy Status | Mother's Pre-Pregnancy BMI | Mother's Weight Gain During Pregnancy | Tobacco, Alcohol, or Drug Use During Pregnancy? | Medications During Pregnancy? | Maternal Complications During Pregnancy? | Maternal Complications During Labor, Delivery, or Post-Partum? |
|---|---|---|---|---|---|---|---|---|---|---|---|
| One Dose BPG 2.4 MU / Three Doses BPG 2.4 MU | xxxxxx | xx | xx | xxx | xxx | xxx | xxx | Y/N | Y/N | Y/N | Y/N |
| One Dose BPG 2.4 MU / Three Doses BPG 2.4 MU | xxxxxx | xx | xx | xxx | xxx | xxx | xxx | Y/N | Y/N | Y/N | Y/N |

Note: Maternal Complications are included in the Adverse Event Listing. Medications taken during pregnancy are included in the Concomitant Medications Listing.

## Listing 18: Pregnancy Reports – Gravida and Para

| | | | Live Births | | | | | | | | | | | | |
|---|---|---|---|---|---|---|---|---|---|---|---|---|---|---|---|
| **Subject ID** | **Pregnancy Number** | **Gravida** | **Extremely Preterm Births** | **Very Preterm Births** | **Early Preterm Births** | **Late Preterm Births** | **Early Term Births** | **Full Term Births** | **Late Term Births** | **Post Term Births** | **Still Births** | **Spontaneous Abortion/ Miscarriage** | **Elective Abortions** | **Therapeutic Abortions** | **Major Congenital Anomaly with Previous Pregnancy?** |
| xxxxxx | xxx | xxx | xxx | xxx | xxx | xxx | xxx | xxx | xxx | xxx | xxx | xxx | xxx | Xxx | Y/N |
| xxxxxx | xxx | xxx | xxx | xxx | xxx | xxx | xxx | xxx | xxx | xxx | xxx | xxx | xxx | Xxx | Y/N |

Note: Gravida includes the current pregnancy, para events do not.

**Listing 19: Pregnancy Reports – Live Birth Outcomes**

| Subject ID | Pregnancy Number | Fetus Number | Pregnancy Outcome (for this Fetus) | Fetal Distress During Labor and Delivery? | Delivery Method | Gestational Age at Live Birth | Size for Gestational Age | Apgar Score, 1 minute | Apgar Score, 5 minutes | Cord pH | Congenital Anomalies? | Illnesses/ Hospitalizations within 1 Month of Birth? |
|---|---|---|---|---|---|---|---|---|---|---|---|---|
| xxxxxx | xxx | xxx | xxx | xxx | xxx | xxx | xxx | xxx | xxx | xxx | Y/N | xxx |
| xxxxxx | xxx | xxx | xxx | xxx | xxx | xxx | xxx | xxx | xxx | xxx | Y/N | xxx |

Note: Congenital Anomalies are included in the Adverse Event listing.

## Listing 20: Pregnancy Reports – Still Birth Outcomes

| Subject ID | Date of Initial Report | Fetus Number | Pregnancy Outcome (for this Fetus) | Fetal Distress During Labor and Delivery? | Delivery Method | Gestational Age at Still Birth | Size for Gestational Age | Cord pH | Congenital Anomalies? | Autopsy Performed? | If Autopsy, Etiology for Still Birth Identified? |
|---|---|---|---|---|---|---|---|---|---|---|---|
| xxxxxx | xx | xxx | xxxxxxx | Y/N | xxxxxxx | xxx | xxx | xx | Y/N | Y/N | xxxxxxx |

## Listing 21: Pregnancy Reports – Spontaneous, Elective, or Therapeutic Abortion Outcomes

| Subject ID | Date of Initial Report | Fetus Number | Pregnancy Outcome (for this Fetus) | Gestational Age at Termination | Abnormality in Product of Conception? | Reason for Therapeutic Abortion |
|---|---|---|---|---|---|---|
| xxxxxx | xx | xxx | xxxxx | xxxx | Y/N | Xxxxxxx |

**Listing 22: Syphilis Clinical Assessment**

[Implementation Note:

Sort order: Treatment Group, Subject ID, Clinical diagnosis of untreated syphilis at baseline, and Planned Time Point.

In the “If Yes, specify type of rash” column and “If present at follow-up visit, specify” column, concatenate “If Other, specify field” by “:”.

“If Yes, specify type of rash”= “N/A” and “If Yes, specify rash location”= “N/A” when “Rash (other than plantar/palmar) or lesion”= “No”.]

| Planned Time Point | Actual Study Day | Rash or lesion? If Yes, specify type of rash | If Rash or lesion was present, specify rash location | If Rash or lesion was present at follow-up visit, specify | Palmar/plantar rash? If Yes, specify type of rash | Condylomata lata | If Condylomata lata was present at follow-up visit, specify | Mucous patch | If Mucous patch was present at follow-up visit, specify | New or worsening signs? If Yes, specify |
|---|---|---|---|---|---|---|---|---|---|---|
| **Treatment Group: subject ID , Clinical diagnosis of untreated syphilis at baseline: Primary/ Secondary/ Early latent:** | | | | | | | | | | |
| Visit 1 | x | Yes/No, Macular/ Papular/ Maculo-papular/ Papulo-squamous/ Other:xx | Face and head/ Trunk/ Genital region/ Extremities | N/A | Yes/No, Macular/ Papular/ Maculo-papular/ Papulo-squamous/ Other:xx | Yes/No | N/A | Yes/No | N/A | N/A |
| Visit 2 | x | Yes/No, Macular/ Papular/ Maculo-papular/ Papulo-squamous/ Other:xx | Face and head/ Trunk/ Genital region/ Extremities | New sign/ Worsening/No change/Improving/ Other:xx | Yes/No, Macular/ Papular/ Maculo-papular/ Papulo-squamous/ Other:xx | Yes/No | New sign/ Worsening/No change/Improving/ Other:xx | Yes/No | New sign/ Worsening/ No change/ Improving/ Other:xx | Yes: xxx /No |
| Visit 3 | x | Yes/No, Macular/ Papular/ Maculo-papular/ Papulo-squamous/ Other:xx | Face and head/ Trunk/ Genital region/ Extremities | New sign/ Worsening/ No change/Improving/ Other:xx | Yes/No, Macular/ Papular/ Maculo-papular/ Papulo-squamous/ Other:xx | Yes/No | New sign/ Worsening/No change/Improving/ Other:xx | Yes/No | New sign/ Worsening/ No change/ Improving/ Other:xx | Yes: xxx /No |


PRIVILEGED AND CONFIDENTIAL COMMUNICATION

**Listing 23: HIV Testing – All Enrolled Subjects**

[Implementation Notes:

1.Sort order is Baseline HIV Status, treatment group, Subject ID, Study Visit.

2.If all subjects received the correct treatment, only display "Treatment Group".

3.If there are test results at interim visits, e.g., 06S, 06T, display the corresponding scheduled visit under "Study Visit" column, e.g., Visit 6.]

| Study Visit | Blood Collection Date for HIV CD4 Count | Blood Collection Date for Viral Load | Blood Collection Date for HIV Test | Had CD4 count record in the past 30 days? | If No CD4 record, was an HIV CD4 count performed | HIV CD4 Count (cells/mm3) | Had viral load record in the past 6 months[a]? | Was result detectable? | Viral Load Result (copies/mL) | Was an HIV test performed? | HIV Test Result |
|---|---|---|---|---|---|---|---|---|---|---|---|
| **Baseline HIV Status: Known Positive/ Unknown or Known Negative, Randomized Treatment Group: , Subject ID:** | | | | | | | | | | | |
| Visit 1 | dd/MMM/yyyy | dd/MMM/yyyy | dd/MMM/yyyy | Yes / No | Yes / No | xxxx | Yes / No | Yes / No | xxxxx or <xx | Yes / No | Negative/ Positive |
| Visit 2 | N/A | dd/MMM/yyyy | N/A | N/A | N/A | N/A | Yes / No / N/A | Yes / No | xxxxx or <xx | N/A | N/A |
| Visit 3 | N/A | dd/MMM/yyyy | N/A | N/A | N/A | N/A | Yes / No / N/A | Yes / No | xxxxx or <xx | N/A | N/A |
| Visit 4 | dd/MMM/yyyy | dd/MMM/yyyy | N/A | Yes / No / N/A | Yes / No | xxxx | Yes / No / N/A | Yes / No | xxxxx or <xx | N/A | N/A |
| Visit 5 | N/A | N/A | dd/MMM/yyyy | N/A | N/A | N/A | N/A | N/A | N/A | Yes / No | Negative/ Positive |
| Visit 6 | dd/MMM/yyyy | N/A | dd/MMM/yyyy | Yes / No / N/A | Yes / No | xxxx | N/A | N/A | N/A | Yes / No | Negative/ Positive |
| Visit 7 | N/A | N/A | dd/MMM/yyyy | N/A | N/A | N/A | N/A | N/A | N/A | Yes / No | Negative/ Positive |
| Visit 8 | dd/MMM/yyyy | N/A | dd/MMM/yyyy | Yes / No / N/A | Yes / No | xxxx | N/A | N/A | N/A | Yes / No | Negative/ Positive |

Notes: For the follow-up visits, HIV Testing could be done at Visits 5 – 8 Only; HIV CD4 Count could be done at Visits 4, 6, 8 Only; HIV Viral Load could be done at Visits 2, 3, 4 Only.
[a] For Visit 1, the question is "Do the subject's medical records include a viral load in the past 6 months?"; For follow-up visits, the question is "Do the subject's medical records include a viral load since the last visit?".

**Listing 24: TPPA Testing - All Enrolled Subjects**

[Implementation Notes:

1. Sort order will be by Actual Treatment Group and Subject ID, Planned Time Point.
2. Only show Visit 5 if V1 was non-reactive and a repeat testing was done for the subject. Remove data entry error where there are results at visits other than V1 after QC.]

| Randomized Treatment Group | Subject ID | Planned Time Point | Actual Study Day | TPPA Result |
|---|---|---|---|---|
| One Dose BPG 2.4 MU / Three Doses BPG 2.4 MU | xxxxx | Visit 1 | xxx | REACTIVE/NON-REACTIVE/INVALID |
| One Dose BPG 2.4 MU / Three Doses BPG 2.4 MU | xxxxx | Visit 5 | xxx | REACTIVE/NON-REACTIVE/INVALID |

**Listing 25: Vital Signs – Safety Population**

[Implementation Notes:

1. Sort order is treatment group, Subject ID, Study Day.]

| Randomized Treatment Group | Subject ID | Planned Time Point | Actual Study Day | Temperature (°F) | Systolic Blood Pressure (mmHg) | Diastolic Blood Pressure (mmHg) | Pulse (beats/min) | Respiratory Rate (breaths/min) |
|---|---|---|---|---|---|---|---|---|
| One Dose BPG 2.4 MU / Three Doses BPG 2.4 MU | xxxxxxx | Visit 1 (Day 1) | xx | xx | xx | xx | Xx | xx |
| One Dose BPG 2.4 MU / Three Doses BPG 2.4 MU | xxxxxxx | Visit 2, Week1 (Day 7 to 13) | xx | xx | xx | xx | Xx | xx |
| One Dose BPG 2.4 MU / Three Doses BPG 2.4 MU | xxxxxxx | Visit 3, Week 2 (6 to 12 days after Visit 2) | xx | xx | xx | xx | Xx | xx |
| One Dose BPG 2.4 MU / Three Doses BPG 2.4 MU | xxxxxxx | Visit 4, Month 1 (Day 30 ± 7 days) | xx | xx | xx | xx | Xx | xx |
| One Dose BPG 2.4 MU / Three Doses BPG 2.4 MU | xxxxxxx | Visit 5, Month 3 (Day 90 ± 21 days) | xx | xx | xx | xx | Xx | xx |
| One Dose BPG 2.4 MU / Three Doses BPG 2.4 MU | xxxxxxx | Visit 6, Month 6 (Day 180 ± 21 days) | xx | xx | xx | xx | Xx | xx |
| One Dose BPG 2.4 MU / Three Doses BPG 2.4 MU | xxxxxxx | Visit 7, Month 9 (Day 270 ± 28 days) | xx | xx | xx | xx | Xx | xx |
| One Dose BPG 2.4 MU / Three Doses BPG 2.4 MU | xxxxxxx | Visit 8, Week 12 (Day 360 ± 28 days) | xx | xx | xx | xx | Xx | xx |

**Listing 26: Abnormal Physical Exam Findings**

[Implementation Notes:

1.Sort order is actual treatment group, Subject ID, Study Day.

3.Only abnormal findings will be presented.

4.If the physical exam was reported as an AE, then concatenate the Physical exam with the Adverse Events by AENUM and report the AETERM, plus the AE Number

5. If a subject had more than one abnormal finding at a visit, list each abnormality on a separate row.]

| Actual Treatment Group | Subject ID | Planned Time Point | Actual Study Day | Body System | Abnormal Finding | Reported as an AE? (AE Description; AE Number) |
|---|---|---|---|---|---|---|
| One Dose BPG 2.4 MU / Three Doses BPG 2.4 MU | xxxxxxx | Visit 1 (Day 1) | xxx | xxxxxxxx | Genitourinary / Lymph nodes / Rectal / Skin / Oral | N/A |
| One Dose BPG 2.4 MU / Three Doses BPG 2.4 MU | xxxxxxx | Visit 2, Week1 (Day 7 to 13) | xxx | xxxxxxxx | Genitourinary / Lymph nodes / Rectal / Skin / Oral | No / Yes, Solicited AE / Yes, Unsolicited AE, xxxxxx; xx |
| One Dose BPG 2.4 MU / Three Doses BPG 2.4 MU | xxxxxxx | Visit 3, Week 2 (6 to 12 days after Visit 2) | xxx | xxxxxxxx | Genitourinary / Lymph nodes / Rectal / Skin / Oral | No / Yes, Solicited AE / Yes, Unsolicited AE, xxxxxx; xx |
| One Dose BPG 2.4 MU / Three Doses BPG 2.4 MU | xxxxxxx | Visit 4, Month 1 (Day 30 ± 7 days) | xxx | xxxxxxxx | Genitourinary / Lymph nodes / Rectal / Skin / Oral | No / Yes, Solicited AE / Yes, Unsolicited AE, xxxxxx; xx |
| One Dose BPG 2.4 MU / Three Doses BPG 2.4 MU | xxxxxxx | Visit 5, Month 3 (Day 90 ± 21 days) | xxx | xxxxxxxx | Genitourinary / Lymph nodes / Rectal / Skin / Oral | No / Yes, Solicited AE / Yes, Unsolicited AE, xxxxxx; xx |
| One Dose BPG 2.4 MU / Three Doses BPG 2.4 MU | xxxxxxx | Visit 6, Month 6 (Day 180 ± 21 days) | xxx | xxxxxxxx | Genitourinary / Lymph nodes / Rectal / Skin / Oral | No / Yes, Solicited AE / Yes, Unsolicited AE, xxxxxx; xx |
| One Dose BPG 2.4 MU / Three Doses BPG 2.4 MU | xxxxxxx | Visit 7, Month 9 (Day 270 ± 28 days) | xxx | xxxxxxxx | Genitourinary / Lymph nodes / Rectal / Skin / Oral | No / Yes, Solicited AE / Yes, Unsolicited AE, xxxxxx; xx |
| One Dose BPG 2.4 MU / Three Doses BPG 2.4 MU | xxxxxxx | Visit 8, Week 12 (Day 360 ± 28 days) | xxx | xxxxxxxx | Genitourinary / Lymph nodes / Rectal / Skin / Oral | No / Yes, Solicited AE / Yes, Unsolicited AE, xxxxxx; xx |

Note: This list consists of subjects who had abnormal findings.
