# Supplementary material for: Jarisch-Herxheimer Reaction After Benzathine Penicillin G Treatment in Adults With Early Syphilis: Secondary Analysis of a Randomized Clinical Trial
Source: JAMA Netw Open. 2025 Feb 13;8(2):e2459490. doi: 10.1001/jamanetworkopen.2024.59490 (PMC11826359; doi:10.1001/jamanetworkopen.2024.59490)
Supplement: Supplement 2. — Data Sharing Statement [file jamanetwopen-e2459490-s002.pdf]

Data Sharing Statement

Dionne. Jarisch-Herxheimer Reaction After Benzathine Penicillin G Treatment in Adults With Early Syphilis. *JAMA Netw Open*. Published February 13, 2025.
doi:10.1001/jamanetworkopen.2024.59490

Data
Additional Information: NCT03637660
Data available: Yes
Data types: Deidentified participant data
How to access data: jdionne@uabmc.edu
When available: beginning date: 12-31-2024

Supporting Documents
Document types: Other (please specify)
Additional Information: protocol
How to access documents: jdionne@uabmc.edu
When available: beginning date: 12-31-2024

Additional Information
Who can access the data: researchers whose proposed use of the data has been approved
Types of analyses: syphilis research
Mechanisms of data availability: with a signed DAA
